# Molecular analysis of *Culex quinquefasciatus* larvae responses to *Lysinibacillus sphaericus* Bin toxin

**DOI:** 10.1371/journal.pone.0175473

**Published:** 2017-04-13

**Authors:** Chontida Tangsongcharoen, Natapong Jupatanakul, Boonhiang Promdonkoy, George Dimopoulos, Panadda Boonserm

**Affiliations:** 1 Institute of Molecular Biosciences, Mahidol University, Salaya, Phuttamonthon, Nakhon Pathom, Thailand; 2 W. Harry Feinstone Department of Molecular Microbiology and Immunology, Bloomberg School of Public Health, Johns Hopkins University, Baltimore, Maryland, United States of America; 3 National Center for Genetic Engineering and Biotechnology, National Science and Technology Development Agency, Khlong Nueng, Khlong Luang, Pathum Thani, Thailand; Institute of Plant Physiology and Ecology Shanghai Institutes for Biological Sciences, CHINA

## Abstract

*Lysinibacillus sphaericus* produces the mosquito larvicidal binary toxin consisting of BinA and BinB, which are both required for toxicity against *Culex* and *Anopheles* larvae. The molecular mechanisms behind Bin toxin-induced damage remain unexplored. We used whole-genome microarray-based transcriptome analysis to better understand how *Culex* larvae respond to Bin toxin treatment at the molecular level. Our analyses of *Culex quinquefasciatus* larvae transcriptome changes at 6, 12, and 18 h after Bin toxin treatment revealed a wide range of transcript signatures, including genes linked to the cytoskeleton, metabolism, immunity, and cellular stress, with a greater number of down-regulated genes than up-regulated genes. Bin toxin appears to mainly repress the expression of genes involved in metabolism, the mitochondrial electron transport chain, and the protein transporter of the outer/inner mitochondrial membrane. The induced genes encode proteins linked to mitochondrial-mediated apoptosis and cellular detoxification including autophagic processes and lysosomal compartments. This study is, to our knowledge, the first microarray analysis of Bin toxin-induced transcriptional responses in *Culex* larvae, providing a basis for an in-depth understanding of the molecular nature of Bin toxin-induced damage.

## Introduction

Mosquito-borne diseases, such as West Nile fever, encephalitis, dengue, malaria, and lymphatic filariasis, are major public health concerns in tropical and subtropical climates [1]. Among the three mosquito genera, *Culex* is the most genetically diversified because of its wide geographic distribution [2]. Several strategies have been developed to control its vector populations and disease transmission in endemic areas. In the past, mosquito control had primarily been undertaken using chemical insecticides, which not only cause environmental problems but also lead to insecticide resistance [3, 4]. To tackle these problems, alternative mosquito control strategies have been developed, such as the use of microorganisms as biological insecticides. Bin toxin, derived from the bacterium *Lysinibacillus sphaericus* (*Ls*), is one of the insecticidal toxins that have been used to control *Culex* and *Anopheles* mosquitoes [5]. However, the increasing application of bioinsecticides in the field has recently led to various cases of insect resistance [6]. An understanding of Bin toxin’s mechanism of action and the mechanism of resistance to this toxin will help us develop a more effective toxin; however, research in this area is currently lacking and is urgently needed.

Bin toxin is a binary toxin composed of the BinA and BinB proteins, which are required in equimolar amounts for maximal toxicity against larvae [7]. After ingestion by susceptible larvae, these proteins are solubilized in the midgut under alkaline conditions and activated by gut proteases to form active toxins [8]. BinB has been shown to be involved in the initial receptor binding and interaction with BinA prior to internalization of the toxin complex [9]; the mechanism underlying this process is currently unknown. The receptor in *Culex pipiens* has been identified as the glycosylphosphatidylinositol (GPI)-anchored *C*. *pipiens* maltase 1 (Cpm1), which is present on the epithelial cells [10]. Many bacterial toxins such as VacA toxin, clostridial neurotoxins, and aerolysin interact with GPI-anchored proteins and enter cells through the endocytic pathway [11, 12]. *Culex* larvae lacking the Cpm1 receptor are resistant to the toxin, suggesting that the receptor recognition is crucial for mediating larval intoxication. Moreover, the specificity of the toxin for the receptor can also explain the differences in toxicity among different mosquito species [13]. Previous studies have reported that the deposition of the Bin complex on lipid membranes can promote conformational changes that facilitate the insertion of BinB into the lipid monolayer [14, 15]. Interestingly, we previously reported that the crystal structure of BinB is similar to that of the aerolysin type β-hairpin pore-forming toxins [16], suggesting the possibility that Bin toxin could be internalized through pore formation and subsequently induce its pathological effects on target cells, including mitochondrial swelling, endoplasmic reticulum breakdown, vacuole formation, and microvillar disruption [17]. A previous report has described a Bin toxin-induced autophagic response in mammalian cells [18]. Moreover, the mitochondrial pathway-mediated apoptosis in susceptible mosquito larvae that results from exposure to Bin has been suggested to contribute to larval death [19].

A previous whole-genome oligonucleotide microarray-based transcriptomic approach to characterize gene expression in the larval gut of *Ostrinia nubilalis* treated with *Bacillus thuringiensis* Cry1Ab toxin revealed several physiological changes in response to Cry1Ab intoxication [20]. In the present study, we have performed a comparative genome-wide microarray-based transcriptomic profiling of *C*. *quinquefasciatus* in response to Bin toxin exposure at different time points in order to better understand the results of toxin treatment, which could benefit the development of this potent insecticidal toxin. Our analyses have revealed the activation of apoptotic processes in the mosquito.

## Materials and methods

### Ethics statement

This study was carried out in strict accordance with the recommendations in the Guide for the Care and Use of Laboratory Animals of the National Institutes of Health. Mice were used only for mosquito rearing as a blood source, according to the approved protocol. The protocol was approved by the Animal Care and Use Committee of the Johns Hopkins University (Permit Number: M006H300).

### Larvae intoxication and RNA preparation

*Culex quinquefasciatus* (JHB strain) eggs were obtained from the Centers for Disease Control and Prevention (CDC, Atlanta, GA USA). *Culex* larvae were reared in distilled water at 28°C with 12 h light / dark cycles. Bin toxin was prepared from *Escherichia coli* BL21 (DE3) pLysS harboring the plasmid containing 6xHis-BinA and 6xHis-BinB, as described previously [21]. The third-instar *C*. *quinquefasciatus* larvae were starved for 3 h before feeding with the mixture of BinA and BinB at a 1:1 molar ratio. Four biological replicates of 20 third-instar *Culex* larvae were treated with an LC_90_ dose (80 ng/ml) of Bin toxin for 6, 12, or 18 h [19]; *Culex* larvae without Bin toxin treatment were used as negative controls. The treated and non-treated larvae that were still alive after a 6-, 12-, or 18-h exposure time were collected for transcriptomic analysis. Both control and toxin-treated *Culex* larvae were anesthetized on ice and then dissected at 4°C in RNAlater (Ambion). Their midguts were stored in RLT buffer (Qiagen) at -80°C until RNA extraction. For RNA extraction, the midguts were homogenized with a rotor-stator homogenizer (Next Advance Bullet Blender), and the RNA was extracted using an RNeasy Mini kit (Qiagen) according to the manufacturer’s protocol. RNA quantitation was measured on a NanoDrop 2000 spectrophotometer (Thermo Scientific), and RNA quality was determined using the Agilent 2200 TapeStation (Agilent Technologies).

### Microarray analysis

A Low Input Quick Amp labeling kit (Agilent Technologies) was used to synthesize negative control (Cy-3-labeled) or Bin toxin-treated (Cy-5-labeled) cRNA probes from 200 ng of total RNA per replicate. Labeled probes were hybridized on an Agilent custom microarray (4 x 44K platform) containing 42,990 probes to represent 22,913 different *C*. *quinquefasciatus* genes and 18,883 protein-coding genes from a NCBI database (Platform GPL10712) [2]. Hybridization intensities were determined using an Agilent SureScan Scanner. Image data were analyzed with Agilent’s Feature Extraction software. The linear models for microarray data (limma) package in R program was used to process the gene expression data from microarray [22]. The raw signal intensities of each array were corrected by backgroundCorrect function then normalized within array by loess method. Replicate spots in each array were averaged with avereps function. Linear model was generated with lmFit function then the statistical significance of differential expression was analyzed by empirical Bayes moderated t-statistics. Finally, the raw p value was adjusted with Benjamini-Hochberg (BH) method at a significance level of adjusted p<0.05 [23]. Self-self hybridization was used to determine the intensity-dependent cutoff value for the significance of differentially expressed genes on these types of microarrays as 0.75 in log_2_ scale, which corresponds to a 1.68 fold-change in regulation. Gene expression data were described in terms of function by homology to functionally annotated genes in *Culex* gene databases such as Gene Ontology [2]. Microarray data has been deposited to GEO NCBI with accession number GSE96838.

### Real-time PCR (qPCR)

Real-time PCR (qPCR) was used to validate the gene expression levels of cell death-related genes involved in the response to toxin in the immune system and redox, stress, and mitochondrion categories. One microgram of total RNA from either non-treated (control) or toxin-treated *Culex* larvae used for microarray analysis was treated with Turbo DNaseI (Ambion) and reverse-transcribed with M-MLV Reverse Transcriptase (Promega). Specific primers were designed and used for qPCR validation. cDNA quantification was performed in a StepOne Real-Time PCR System by using a SYBR Green PCR Kit (Applied Biosystems). The primer sequences were:

*Culex* ribosomal protein S7 (CPIJ006763 -RA):
  F: 5′AGAACCAGCAGACCACCATC-3′
  R: 5′-ACCCTCCCACTTCTCCATCT-3′
Caspase-3 (CPIJ009057-RA):
  F: 5′-GAAAACGGAGAACGGTACGA-3′
  R: 5′-CTCCAGTGTATCGGGAGCAT-3′
Caspase-1 (CPIJ008093 -RA):
  F: 5′-ATTATCTGATGGCGCAGGAC-3′
  R: 5′-TTGCCGGCCATAGAGTTAAG-3′
Cytochrome c (CPIJ019024-RA):
  F: 5′-AGGGTATCACGTGGAACGAG-3′
  R: 5′-TGCTGCAACACAGGTTTAGG-3′

Each transcript sample was normalized to the endogenous housekeeping gene, the *C*. *quinquefasciatus* ribosomal S7 gene. The -fold change in gene expression of each transcript in the toxin treatment samples was compared to the non-treated (control) value. Significantly different expression was defined by Student’s *t*-test as p<0.05.

## Results and discussion

### Bin toxin exposure induces diverse transcriptomic responses

**Fig 1:**
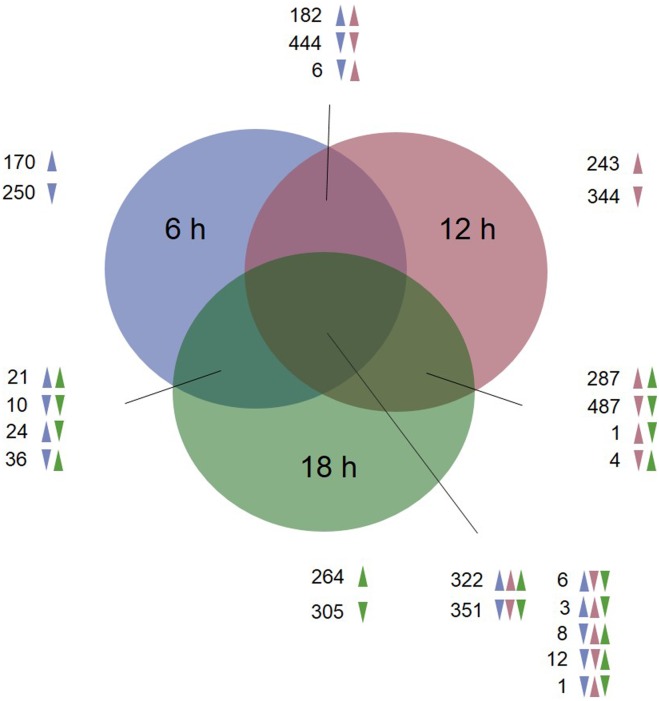
Venn diagram showing the numbers of unique and commonly regulated genes in the *C*. *quinquefasciatus* larval gut in response to LC_90_ dose of Bin toxin at different exposure times as compared to those for non-treated larvae. Data represent transcripts with ≥1.68-fold changed regulation at a significance level of p value<0.05 in *Culex* larvae upon Bin treatment. The overlapping regions represent genes regulated under two or three experimental conditions. The arrows indicate the direction of gene regulation.

**Fig 2:**
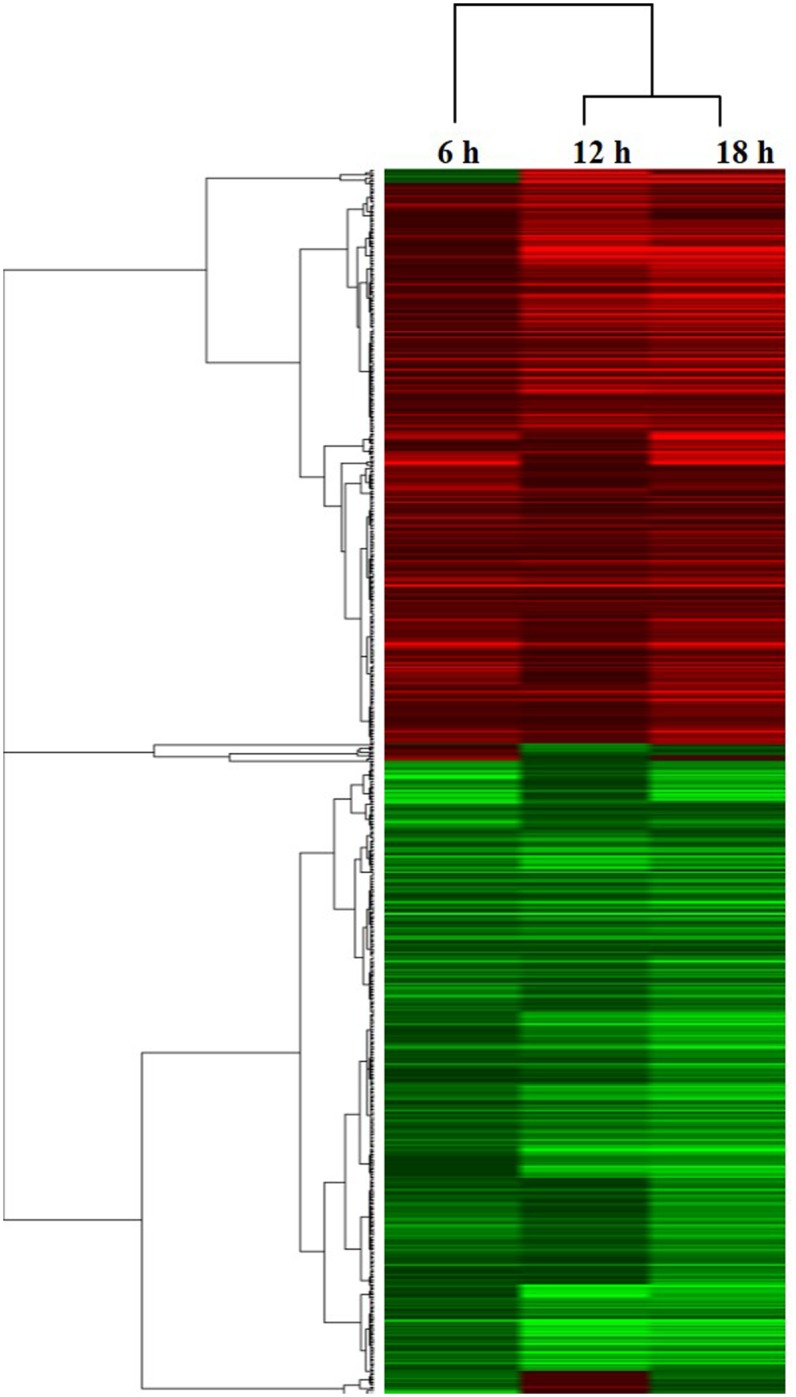
Differentially regulated genes of Bin toxin-treated (LC_90_ dose) *Culex* larvae compared to those of non-treated larvae. Hierarchical cluster analysis of differentially expressed genes in Bin toxin-treated *C*. *quinquefasciatus* larval guts as compared to those from non-treated *Culex* larvae. A total of 703 transcripts, which were significantly regulated at all three (6-, 12-, and 18-h) time points, is presented in S2 Table. Transcripts with at least a 0.75-fold regulation in log_2_-scale correspond to a > = 1.68-fold regulation and a p value<0.05. Genes with lowered transcript abundance are shown in green, and red indicates genes with a higher transcript abundance than the corresponding gene from the non-treated *C*. *quinquefasciatus* larval gut. More intense colors indicate higher levels of gene regulation.

To study the global gene expression changes elicited by the exposure of *Culex* larvae to Bin toxin, we used oligonucleotide microarray analysis to compare untreated and Bin-treated *Culex* larvae at 6, 12, and 18 h after toxin exposure. According to our recent publication, both cytopathological changes and biochemical evidence of apoptosis induced by Bin toxin were found in *Culex* larvae at these time points [19]. As many as 3,780 transcripts were differentially regulated upon Bin toxin exposure in at least one treatment group, representing 16.5% of the annotated *C*. *quinquefasciatus* transcriptome; this was a relatively large number when compared to previous transcriptional responses to pathogen treatment in lepidopterans, which typically range from 1–11% [24–26], with 7% for dipterans [27] and 1% for coleopterans [28]. A recent study has also revealed a large transcriptional impact of Vip3Aa toxin treatment of the lepidopteran *Spodoptera exigua*, comprising 19% of all unigenes [29]. Our results indicate that Bin toxin has a major physiological impact on *C*. *quinquefasciatus*. The numbers of significantly regulated genes at each time point were: 728 up-regulated and 1,118 down-regulated at 6 h; 1,053 up-regulated and 1,648 down-regulated at 12 h; and 954 up-regulated and 1,188 down-regulated at 18 h after exposure. Of the 3,780 regulated genes, the total number that was down-regulated was greater than the number up-regulated. It is notable that Bin toxin exposure mainly resulted in a decrease in gene expression in the *Culex* larvae. These results are in agreement with previous studies on coleopteran and lepidopteran insect exposure to *Bt* Cry toxins [28, 30]. The changes in transcriptional response of the toxin-treated *Culex* cells were dynamic throughout the time-course of Bin toxin exposure, with the greatest change at the 12-h time point. A total of 2,204 genes were differentially regulated at all three assayed time points (Fig 1), suggesting the involvement of major biological processes in the response to Bin intoxication. The genes that were significantly regulated (p<0.05) at all time points [6, 12, and 18 h], 703 of 22913 transcripts, were subjected to cluster analysis using a hierarchical clustering algorithm (Fig 2 and S1 Table). The clustering analysis revealed that genes regulated by 12 h of Bin exposure were closely related to those exposed for 18 h, whereas the expression profiles of those exposed for 6 h were less related.

### Transcriptional responses of *C*. *quinquefasciatus* larvae treated with Bin toxin

**Fig 3:**
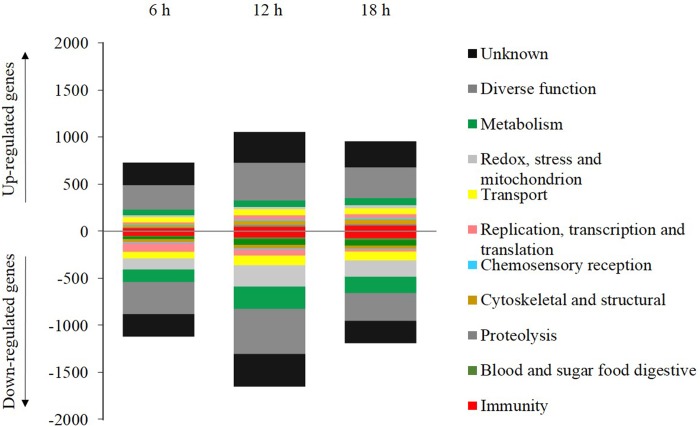
Functional classification of the Bin toxin intoxication transcriptome. The significantly changed transcripts in *Culex* after Bin toxin treatment for 6, 12, or 18 h were subsequently classified into 11 functional groups: 1) cytoskeletal and structural function (CST); 2) chemosensory reception (CSR); 3) blood and sugar food digestive (DIG); 4) diverse functions (DIV); 5) immunity (IMM); 6) metabolism (MET); 7) proteolysis (PRT); 8) redox, stress, and mitochondrion (RSM); 9) replication, transcription, and translation (RTT); 10) transport (TRP); and 11) unknown function (UNK). Bin toxin treatment responsive gene expression data is presented in S2 Table.

To better understand the biological processes and pathways in which the differentially regulated genes participate, we examined their functional classification (Fig 3), which revealed that the genes regulated by an LC_90_ dose of Bin toxin were associated with various biological processes in *Culex* larvae. As many as 2,830 regulated genes showed homology to genes of known function, representing 74.9% of all regulated genes, and the remaining 950 genes (25.1%) were of unknown function. With the exception of the genes with unknown function and those with diverse functions (32.3% of all regulated genes), the highest number of regulated genes from Bin-treated *Culex* larvae was associated with metabolism (10.4% of all regulated genes), and this class had about twice as many down-regulated as up-regulated genes. Similarly, other studies of *Ostrinia nubilalis* fed on Cry1Ab toxin and studies of *S*. *exigua* challenged with Vip3Aa toxin have also revealed predominant down-regulation of genes that were principally involved in metabolic processes [20, 29]. This down-regulation of metabolic processes is likely related to the loss of energy production, causing the slow movement of treated *Culex* larvae.

### Genes involved in Bin toxin entry and binding

A key role in toxicity is played by *Culex* receptor binding of the Bin toxin, which enables its entry into the mosquito [31]. We found that transcripts putatively involved in toxin binding and entry were differentially expressed in response to the ingestion of Bin toxin (Table 1). Transcripts involved in receptor binding, including α-glucosidases (CPIJ008904), α-amylases (CPIJ008079, CPIJ001464, CPIJ005060), and maltase1 (CPIJ013170) were down-regulated in response to Bin ingestion after 12 h of Bin treatment [32]. We hypothesize that the repressed expression of these proteins might act as defense against the effects of Bin by reducing toxin recognition and absorption of nutritive substrates. Toxin entry into cells by endocytosis may involve in the overexpression of Cdc-42 (CPIJ014902), a small GTPase of the Rho subfamily, during 12–18 h of Bin intoxication [33]. Moreover, transcripts related to intracellular membrane trafficking, including the small-GTP binding proteins Rab-5 (CPIJ001495), Rab-7 (CPIJ009089), Rab-8 (CPIJ006714), and Rab-10 (CPIJ005169), which are typical markers of early and late endosomes, were up-regulated after 6 h of Bin exposure, supporting the hypothesis that Bin toxin entry may involve the endocytic pathway. This observation is in agreement with a previous study on Bin internalization via receptor-mediated endocytosis [18].

**Table 1:** Log_2_-fold values and functional groups of transcripts that were enriched or depleted in *C*. *quinquefasciatus* gut larvae in response to Bin treatment (LC_90_ dose) at 6, 12, or 18 h: (blood and sugar food digestive [DIG]; diverse functions [DIV]; immunity [IMM]; proteolysis [PRT]; redox, stress, and mitochondrion [RSM]; and transport [TRP]).

**Gene ID**	**Functional group**	**Gene name**	**Log_2_ fold**		
			**6 h**	**12 h**	**18 h**
**Toxin binding and entry**					
CPIJ008904	DIG	alpha-glucosidase		-1.764	-3.091
CPIJ008079	DIG	alpha-amylase 1		-2.53	-3.071
CPIJ001464	DIG	alpha-amylase A			-1.4
CPIJ005060	DIG	alpha-amylase B			-1.326
CPIJ013170	DIG	maltase 1			-0.988
CPIJ014902	DIV	CDC42 homolog		2.514	2.886
CPIJ006714	DIV	ras-related protein Rab-8A		1.238	1.54
CPIJ009089	DIV	ras-related protein Rab-7	0.902	1.111	0.852
CPIJ005169	DIV	ras-related protein Rab-10			0.862
CPIJ012218	DIV	Rho-GTPase	1.981	1.719	
CPIJ001495	DIV	Rab-5		0.786	
**Immune response**					
CPIJ014718	IMM	serine protease inhibitor 4, serpin-4	1.266	2.704	2.604
CPIJ012013	IMM	serine protease inhibitor, serpin	0.831	2.865	1.92
CPIJ012016	IMM	serine protease inhibitor, serpin			0.9
CPIJ007019	IMM	serine protease inhibitor		0.842	
CPIJ003759	IMM	serine protease inhibitor			0.884
CPIJ016297	IMM	serine protease inhibitor A3K			0.84
CPIJ006515	IMM	Toll9	1.391	1.121	
CPIJ008547	IMM	myd88	0.793	0.758	
CPIJ018307	IMM	myd88	0.77	0.823	
CPIJ000574	IMM	cathepsin L	0.847	1.298	
CPIJ012236	IMM	nuclear factor NF-kappa-B p105 subunit	1.688		0.81
CPIJ006917	DIV	NFkappaB essential modulator	1.044	1.351	1.419
CPIJ001276	IMM	defensin-A	-1.808	-1.86	-2.2
CPIJ010699	IMM	cecropin A	-1.305	-1.149	-1.761
**Cell death**					
CPIJ003274	TRP	vacuolar proton translocating ATPase 116 kDa		-0.983	-0.797
CPIJ003418	TRP	V-type ATP synthase beta chain			-0.754
CPIJ007772	TRP	ATP synthase alpha subunit vacuolar			-0.77
CPIJ016432	TRP	vacuolar ATP synthase subunit F			-0.751
CPIJ002067	TRP	vacuolar ATP synthase subunit C		-0.863	
CPIJ008618	TRP	chloride channel protein 7		1.075	1.16
CPIJ003880	TRP	chloride channel protein 2	-0.751	-1.866	-1.68
CPIJ012964	DIV	autophagy protein 9	1.16	1.08	
CPIJ002100	DIV	autophagy-specific gene 12			1.043
CPIJ002931	DIV	ubiquitin protein ligase		1.257	1.15
CPIJ001423	DIV	ubiquitin-activating enzyme E1			0.858
CPIJ018658	DIV	ubiquitin-conjugating enzyme E2			0.77
CPIJ004272	DIV	ubiquitin-conjugating enzyme rad6			0.929
CPIJ000897	DIV	proteasome subunit alpha type 1	-1.445	-1.732	
CPIJ006946	DIV	proteasome subunit alpha type 2		-1.725	-1.085
CPIJ003586	DIV	proteasome subunit alpha type 3	-1.088	-1.716	
**Gene ID**	**Functional group**	**Gene name**	**Log fold**		
			**6 h**	**12 h**	**18 h**
**Cell death**					
CPIJ010893	DIV	proteasome subunit alpha type 4	-1.017	-1.952	
CPIJ001707	DIV	proteasome subunit alpha type 6		-1.4	-0.766
CPIJ010114	DIV	proteasome subunit alpha type	-0.899	-1.115	
CPIJ008264	PRT	proteasome subunit beta type 3	-1.558	-1.908	
CPIJ003987	PRT	proteasome subunit beta type 1	-1.006	-1.372	
CPIJ001361	PRT	proteasome subunit beta type 8	-0.858	-1.543	
CPIJ003351	DIV	26S proteasome non-ATPase regulatory subunit 6	-1.064	-1.171	
CPIJ004894	IMM	bax inhibitor	0.837	0.976	0.856
CPIJ002689	IMM	survivin	-0.827	-0.95	
CPIJ002102	IMM	apoptosis 1 inhibitor		1.136	1.066
CPIJ009056	IMM	caspase-3		1.803	1.594
CPIJ009057	IMM	caspase-3	0.807	1.743	1.979
CPIJ012580	IMM	caspase-3			1.293
CPIJ008252	IMM	caspase		1.169	1.529
CPIJ008093	IMM	caspase-1	-0.81		
CPIJ006908	RSM	carboxylesterase-6		-1.189	-2.523
CPIJ002384	RSM	endoplasmin	-1.469	-2.013	
CPIJ011727	DIV	translocon-associated protein, gamma subunit	-1.435	-2	
CPIJ004301	DIV	endoplasmic oxidoreductin-1	-1.081	-1.761	
CPIJ002043	DIV	translocon-associated protein, delta subunit	-1.798	-1.665	
CPIJ004778	DIV	ER protein reticulon	-2.107	-1.575	
CPIJ013335	DIV	ctg4a	-1.116	-1.558	
CPIJ013741	RSM	mitochondrial 39S ribosomal protein L27		-0.815	
CPIJ012217	TRP	inositol 1,4,5-triphosphate receptor	0.78	0.999	
CPIJ005134	TRP	calpain	1.001	0.837	
CPIJ007376	TRP	calreticulin	-2.35	-2.788	-1.136
CPIJ009164	DIV	calnexin	-0.89	-0.778	
CPIJ000967	TRP	voltage-dependent anion-selective channel		-1.016	-0.833
CPIJ007967	RSM	mitochondrial import receptor subunit tom20		-1.731	-1.21
CPIJ002542	RSM	mitochondrial import receptor subunit tom40	-0.763	-1.091	
CPIJ010382	RSM	mitochondrial import inner membrane translocase subunit Tim10	-1.308	-1.893	
CPIJ013823	RSM	mitochondrial import inner membrane translocase subunit Tim22	-1.245	-1.49	
CPIJ018054	RSM	mitochondrial import inner membrane translocase subunit Tim8A	-1.473	-1.833	
CPIJ018687	RSM	mitochondrial import inner membrane translocase subunit Tim9		-1.106	
CPIJ010413	RSM	mitochondrial import inner membrane translocase subunit Tim23	-0.837		
CPIJ018666	RSM	NADH dehydrogenase flavoprotein2, mitochondrial		-1.102	-1.21
CPIJ018667	RSM	NADH dehydrogenase flavoprotein2, mitochondrial		-1.298	
CPIJ003877	RSM	NADH-ubiquinone oxidoreductase subunit B14.5b		-0.934	
CPIJ017732	RSM	NADH dehydrogenase 1 alpha subcomplex subunit 12		-0.918	
CPIJ019817	RSM	cytochrome c oxidase assembly protein COX19	-1.261	-1.536	-1.067
CPIJ015280	RSM	cytochrome c oxidase assembly protein COX11	-1.2	-1.907	-1.44
CPIJ016252	RSM	cytochrome c oxidase assembly protein COX15	-0.952	-1.547	
**Gene ID**	**Functional group**	**Gene name**	**Log fold**		
			**6 h**	**12 h**	**18 h**
**Cell death**					
CPIJ017747	RSM	cytochrome c oxidase assembly protein COX15		-0.966	
CPIJ019024	RSM	cytochrome c	-2.279	-2.817	-0.993
CPIJ007010	RSM	peroxisomal membrane protein pmp34	-1.744	-1.935	-2.146
CPIJ002253	RSM	mitochondrial carnitine/acylcarnitine carrier protein		-1.549	-1.137
CPIJ017288	RSM	mitochondrial solute carrier protein			-1.142
CPIJ019834	RSM	mitochondrial carnitine/acylcarnitine carrier protein		-1.638	-1.27
CPIJ019838	RSM	mitochondrial oxodicarboxylate carrier	-0.838	-1.197	
CPIJ012682	RSM	mitochondrial dicarboxylate carrier		-1.276	
CPIJ006475	RSM	mitochondrial 2-oxoglutarate/malate carrier protein		-0.946	
CPIJ016780	RSM	mitochondrial 2-oxoglutarate/malate carrier protein		-0.872	
CPIJ004719	IMM	superoxide dismutase 3.4, mitochondrial	-0.894	-2.095	-1.314

### Bin toxin induces *Culex* immune responses in the gut

Since the Bin toxin is bacteria-derived, it is likely that the toxin can elicit an immune response in the mosquitoes. Both increased and decreased expression of genes involved in the insect immune system were observed in response to treatment with Bin toxin (Table 1). Serine protease inhibitors (serpins), which were up-regulated by Bin intoxication, regulate several immunity-related serine proteases in a number of insects [34, 35]. Transcripts related to antimicrobial peptides, including defensin (CPIJ001276) and cecropin (CPIJ010699), were down-regulated during 6–18 h of Bin exposure. It is possible that the innate immune response in *Culex* larvae is activated before 6 h of Bin treatment and then repressed after 6 h as a consequence of larval death. However, immune-related transcripts such as Toll9 (CPIJ006515), myd88 (CPIJ018307, CPIJ008547) and a lysosomal protease, cathepsin L (CPIJ000574), were enriched after 6–12 h of Bin exposure. Toll9 signaling likely activates nuclear factor NF-kappa-B (CPIJ012236) by up-regulation after 6–18 h of Bin treatment. It is possible that the overexpression of Toll9, myd88, cathepsin L, and nuclear factor NF-kappa-B indicates Toll pathway activation in response to a decreased level of AMPs, since the Toll pathway is important for maintaining the basal level of AMPs [36].

### Bin toxin regulates autophagic processes

We found that cell death-related genes belonging to the immunity (IMM); proteolysis (PRT); redox, stress, and mitochondrion (RSM); transport (TRP); and diverse functions (DIV) were differentially expressed in Bin toxin-exposed *Culex* larvae and to controls (Table 1). The vacuolar-type H^+^-ATPase (V-ATPase) uses energy from ATP hydrolysis to acidify the endosome compartment [37]. During 12–18 h of Bin exposure, the transcript abundance of genes involved in proton transport, including the vacuolar proton translocating ATPase (CPIJ003274) and the vacuolar ATP synthase (CPIJ003418, CPIJ007772, CPIJ016432, CPIJ002067), was decreased, possibly because of the low ATP concentration in the Bin-exposed larval cells. This finding is in contrast to the vacuole formation of *H*. *pylori* VacA toxin, which is accomplished by increasing the V-ATPase activity [38, 39]. Our results support a TEM-based cytopathological study that showed the presence of vacuoles in the cells of Bin-treated mosquitoes, probably as a result of cytoplasmic organelle breakdown and not V-ATPase activity [19]. Moreover, the organelle swelling of cells treated with Bin from 6–18 h may have been caused by the down-regulation of transcripts encoding the chloride channel 2 protein (CPIJ003880), which is a ubiquitous chloride channel abundant in the plasma membrane that is involved in the regulation of cell volume [40]. Interestingly, the transcript level of the chloride channel 7 gene (CPIJ008618), which drives lysosomal acidification in endosomal/lysosomal compartments, was up-regulated after 12–18 h of Bin exposure [41, 42]; this increase in acidification may result in excessive autophagy. The up-regulation of the autophagy-specific genes Atg-9 (CPIJ012964) and Atg-12 (CPIJ002100) suggests that autophagy is activated in Bin-treated cells, resulting in the appearance of autophagic vacuolation. This finding agrees with a previous report showing that Bin-induced vacuole formation originates from autophagic processes [18]. Thus, Bin toxin may induce cellular detoxification by autophagic process as a defensive response to remove damaged cellular components during Bin exposure.

Our transcriptomic data also suggest that Bin toxin may induce the degradation of misfolded proteins and damaged organelles as well as the ubiquitin-proteasome system. At 6–12 h of treatment with Bin toxin, lysosomal cathepsin L (CPIJ000574) and the autophagy protein 9 (CPIJ012964) were up-regulated. Later, at 18 h, autophagy-specific gene 12 (CPIJ002100) was also up-regulated. These data suggest that Bin toxin causes cell death via protein degradation and autophagy.

Ubiquitination is another mechanism necessary for protein degradation via the ubiquitin-proteasome pathway. Our transcriptomic data indicated an up-regulation of Atg 12, one of the ubiquitin-like proteins that has been shown to contribute to proteasome inhibition and mitochondrial apoptosis [43, 44]. Here, protein degradation by ubiquitin-proteasome system was indicated by the overexpression of ubiquitin enzymes including ubiquitin-activating enzyme E1 (CPIJ001423), ubiquitin-conjugating enzyme E2 (CPIJ018658), and ubiquitin-protein ligase (CPIJ002931) at 18 h of Bin exposure; in contrast, transcripts encoding proteasome subunits (CPIJ000897, CPIJ006946, CPIJ003586, CPIJ010893, CPIJ001707, CPIJ010114) were down-regulated after 6 h of Bin treatment. These results suggest that Bin exposure results in a lower expression of proteasome subunits at 6 h, which could result in the aggregation of misfolded proteins, potentially leading to the induction of ubiquitination-related transcripts related to the degradation of these misfolded proteins. Also, these misfolded proteins potentially induce apoptosis, as shown by repressed expression of survivin (CPIJ002689) during 6–12 h of Bin exposure [45–47]. In contrast, transcripts encoding apoptosis inhibitor (CPIJ002102) were up-regulated after 12 h of Bin treatment.

### Bin toxin alters ER-associated genes

Our transcriptomic data further revealed that transcripts associated with the ER, such as carboxylesterase-6 (CPIJ006908), endoplasmin (CPIJ002384), translocon-associated proteins (CPIJ011727, CPIJ002043), endoplasmic oxidoreductin-1 (CPIJ004301), and the ER protein reticulin (CPIJ004778) were down-regulated after 6 h of Bin exposure. These changes in ER-associated transcripts could alter the balance of protein synthesis and the accumulation of misfolded proteins in the endoplasmic reticulum, causing ER stress [19, 48]. The occurrence of ER stress induced by Bin toxin could also affect Ca^2+^ release from ER lumen into the cytosol, as shown by the increased transcript abundance of inositol trisphosphate receptor (InsP3R) (CPIJ012217) and calpain (CPIJ005134) during 6–12 h of Bin treatment as well as the decreased transcript abundance of calreticulin (CPIJ007376) and calnexin (CPIJ009164) [49, 50]. An increased cytosolic Ca^2+^ concentration triggers the translocation of inactive calpain from cytosol to membrane and the activation of calpain [51, 52]. Activated calpain can in turn activate the proapoptotic protein BAX which causes potential mitochondrial membrane loss, resulting in the swelling of the mitochondria and the release of apoptogenic proteins from them [53, 54]. Interestingly, our results indicated that transcripts encoding the Bax inhibitor (CPIJ004894), which regulates ER stress-associated mitochondrial Ca^2+^ accumulation, were up-regulated during 6–18 h of Bin treatment [55].

### Bin toxin induces the degradation of mitochondria

Previous investigation of Bin toxin-induced cellular pathology using TEM have found the occurrence of endoplasmic reticulum (ER) and mitochondrial breakdown after 6 and 18 h of Bin treatment [19]. An increased cytosolic Ca^2+^ concentration can trigger Ca^2+^ flux into the mitochondria through voltage-dependent anion-selective channels (VDAC) in closed configuration, leading to outer mitochondrial membrane permeabilization and apoptogenic release [56, 57]. Furthermore, the down-regulation of transcripts associated with mitochondrial protein transporters including the outer/inner membrane translocase complex (TOM CPIJ007967, CPIJ002542 and TIM CPIJ010382, CPIJ013823, CPIJ018054, CPIJ018687, CPIJ010413) after 6 h of Bin treatment may lead to the reduced translocation of mitochondrial proteins into mitochondria and a decreased outer/inner mitochondrial membrane potential, resulting in the degradation of the mitochondrial membrane [58]. Also, transcripts encoding VDAC (CPIJ000967) on the outer mitochondrial membrane, which regulates the transport of ATP, ADP, Ca^2+^, and metabolites, were down-regulated during 12–18 h of Bin exposure, suggesting that mitochondrial energy production and metabolism should be disrupted [59]. A disruption in ATP/ADP exchange across the mitochondrial membrane occurs as a result of the decrease in the rate of the mitochondrial electron transport chain caused by the suppressed expression of NADH dehydrogenases (CPIJ018666, CPIJ018667, CPIJ003877, CPIJ017732) and cytochrome c oxidase (COX) assembly proteins (CPIJ019817, CPIJ015280, CPIJ016252, CPIJ017747), including cytochrome c (CPIJ019024), during 6–18 h of Bin treatment, leading to the loss of ATP synthesis. Moreover, transcripts related to mitochondrial carrier proteins on the inner mitochondrial membrane (CPIJ002253, CPIJ017288, CPIJ019834, CPIJ006475, CPIJ016780) were down-regulated after 12 h of Bin exposure and probably caused mitochondrial inner membrane hyperpolarization and matrix swelling [60]. Alternatively, the down-regulation of the mitochondrial electron transport chain may have caused the generation of reactive oxygen species (ROS) because the mitochondrial electron transport chain is a major site of oxygen metabolism and ROS production, which are important in the pathogenesis and triggering of cell death [61]. Moreover, a repressed transcription of superoxide dismutase (CPIJ004719) after 6 h of Bin treatment would also increase the production of ROS. These results suggest that alterations in the ER and mitochondria produced by Bin toxin exposure can trigger oxidative damage, accumulation of mitochondrial ROS, and mitochondrial membrane permeabilization, inducing apoptosis via the increased transcription of caspase-3 (CPIJ009056, CPIJ009057 and CPIJ012580) as shown the overexpression after 6–18 h of Bin exposure. Whereas transcript encoding caspase-8 (CPIJ014995) was not significantly changed from that of non-treated cells, indicating that damage from Bin exposure may not involve the death receptor or the extrinsic pathway. These results suggest that Bin intoxication of *Culex* larvae induces mitochondrial degradation, followed by the induction of apoptosis.

### Real-time qPCR confirmation of cell death-related genes

**Fig 4:**
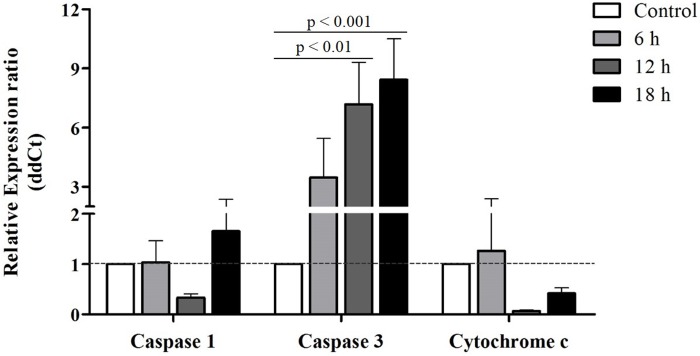
Real-time qPCR confirmation of cell death-related genes in *Culex* treated with Bin toxin at 6, 12, or 18 h. Bar charts represent -fold changes in caspase-1, caspase-3, and cytochrome c gene expression levels relative to levels in the non-treated *C*. *quinquefasciatus* larval gut. Error bars indicate the standard error of the mean from four biological replicates by Student’s *t*-test. Numeric data is presented in S3 Table.

Real-time qPCR was used to validate the microarray expression data of cell death-related genes, including caspase-1, caspase-3, and cytochrome c (Fig 4). Consistent with the increased caspase-3 activity shown in our recent report, we found that both microarray and qPCR analyses showed that transcripts encoding caspase-3 (CPIJ009057) were significantly up-regulated after 12–18 h of Bin exposure [19]. In addition, the expression of cytochrome c, which is involved in the mitochondrial apoptotic pathway, was also repressed [62]. Both microarray and qPCR analyses showed that transcripts encoding caspase-1 (CPIJ008093) were down-regulated after 6 h of Bin exposure. These results support that pathway involved in mitochondria-mediated apoptosis, rather than necrosis, is induced in Bin toxin-treated *Culex* larvae, leading to larval death.

## Conclusion

In summary, our transcriptomic analysis of the *C*. *quinquefasciatus* gut after Bin toxin treatment has revealed a wide range of transcriptional responses, including toxin binding, metabolism, immunity, cellular stress, and cell death. Especially, cellular detoxification by autophagic pathway and mitochondria-mediated apoptosis are involved in Bin intoxication of *Culex* larvae. After the entry of Bin toxin into the cells via receptor-mediated endocytosis, it apparently exerts its cytotoxicity through a variety of intracellular targets. The mitochondria are one of its intracellular targets, as indicated by the decreased in transcript abundance of mitochondrial genes, leading to a reduction in cellular ATP synthesis, followed by outer/inner mitochondrial membrane permeabilization and apoptotic cell death regulated by caspase-3. Transcripts related to protein degradation are involved in autophagy and ubiquitin-proteasome degradation during Bin intoxication. Bin toxin also disrupts the ER through an alteration of Ca^2+^ homeostasis and increased cytosolic Ca^2+^, possibly caused by the loss of mitochondrial membrane potential. Taken together, our data provide insights into the transcriptomic responses in the gut of *C*. *quinquefasciatus* larvae during Bin toxin exposure. In addition to confirming the cellular pathology previously investigated by TEM, our study provides an additional dataset and proposed hypotheses for further characterization of the results of Bin exposure at the molecular level.

## Supporting information

S1 Table Hierarchical cluster analysis of 703 transcripts that were significantly regulated at all three (6-, 12-, and 18-h) time points of Bin toxin-treated *C*. *quinquefasciatus* larvae. (DOCX)

S2 Table The functional groups of the total 3,781 transcripts were enriched or depleted in *C*. *quinquefasciatus* gut larvae in response to LC_90_ dose of Bin toxin at 6, 12, or 18 h. Functional group abbreviations: CST, cytoskeletal and structural function; CSR, chemosensory reception; DIG, blood and sugar food digestive; DIV, diverse functions; IMM, immunity; MET, metabolism; PRT, proteolysis; RSM, redox, stress, and mitochondrion; RTT, replication, transcription, and translation; TRP, transport; UNK, unknown function.
(DOCX)

S3 Table Averaged data from four biological replicate Real-time qPCR assays of caspase-1, caspase-3, and cytochrome c gene expression after 6, 12, or 18 h of Bin exposure. The fold change in gene expression levels relative to levels in the non-treated *C*. *quinquefasciatus* larval gut (control) is shown. p-values are for a Student’s *t*-test comparing fold change in gene expression upon Bin intoxicated cells. *, p<0.05; SEM, standard error of the mean.
(DOCX)
