# Supplementary material for: Molecular analysis of Culex quinquefasciatus larvae responses to Lysinibacillus sphaericus Bin toxin
Source: PLoS One. 2017 Apr 13;12(4):e0175473. doi: 10.1371/journal.pone.0175473 (PMC5391067; doi:10.1371/journal.pone.0175473)
Supplement: S1 Table — (DOCX) [file pone.0175473.s001.docx]

**Table S1:** Hierarchical cluster analysis of 703 transcripts that were significantly regulated at all three (6-, 12-, and 18-h) time points of Bin toxin-treated (LC_90_ dose) *C. quinquefasciatus* larvae.

| **GENE ID** | **NAME** | **FUNCTIONAL GROUP** | **Log_2_ fold** | | |
| --- | --- | --- | --- | --- | --- |
|  |  |  | **6 h** | **12 h** | **18 h** |
| CPIJ001870 | predicted protein | CSR | -1.759 | -2.065 | -2.905 |
| CPIJ001869 | hypothetical protein | CSR | -1.75 | -2.348 | -2.966 |
| CPIJ002605 | serine/threonine kinase | CSR | -1.69 | -2.384 | -2.153 |
| CPIJ017326 | general odorant-binding protein 99a | CSR | -1.464 | -3.863 | -5.333 |
| CPIJ003583 | conserved hypothetical protein | CSR | 0.767 | 1.896 | 2.108 |
| CPIJ004634 | odorant-binding protein | CSR | 0.835 | 1.653 | 1.687 |
| CPIJ002629 | sensory appendage protein | CSR | 1.071 | 1.281 | 2.695 |
| CPIJ002611 | sensory appendage protein | CSR | 1.529 | 3.029 | 2.848 |
| CPIJ019986 | serine/threonine kinase | CSR | 1.887 | 3.18 | 3.396 |
| CPIJ019985 | sensory appendage protein | CSR | 1.905 | 3.122 | 3.597 |
| CPIJ008374 | arrestin domain-containing protein 2 | CSR | 1.956 | 2.565 | 2.195 |
| CPIJ002609 | serine/threonine kinase | CSR | 2.009 | 1.698 | 1.891 |
| CPIJ012934 | conserved hypothetical protein | CST | -3.985 | -3.889 | -1.67 |
| CPIJ001078 | Pupal cuticle protein | CST | -2.091 | -1.856 | -0.965 |
| CPIJ016326 | conserved hypothetical protein | CST | -1.091 | -2.747 | -0.92 |
| CPIJ012928 | tubulin alpha-1 chain | CST | -0.957 | -1.529 | -0.797 |
| CPIJ003900 | bifunctional aminoacyl-tRNA synthetase | CST | 0.813 | 1.334 | 1.194 |
| CPIJ003871 | paxillin | CST | 0.817 | 1.431 | 0.981 |
| CPIJ013337 | tubulin-specific chaperone e | CST | 0.859 | 1.151 | 0.826 |
| CPIJ008005 | annexin x | CST | 0.975 | 1.31 | 0.753 |
| CPIJ014101 | conserved hypothetical protein | CST | 1.017 | 2.059 | 0.784 |
| CPIJ015899 | myosin heavy chain | CST | 1.024 | 1.881 | 1.516 |
| CPIJ008557 | conserved hypothetical protein | CST | 1.289 | 0.925 | 0.757 |
| CPIJ002897 | conserved hypothetical protein | CST | 1.302 | 1.447 | 0.966 |
| CPIJ004628 | Gelsolin | CST | 1.335 | 2.434 | 2.627 |
| CPIJ006518 | hypothetical protein | CST | 1.735 | 3.871 | 3.041 |
| CPIJ013616 | trypsin 5 | DIG | -2.458 | -2.449 | -2.543 |
| CPIJ019598 | basic endochitinase CHB4 | DIG | -2.367 | -1.96 | -0.983 |
| CPIJ004400 | ornithine aminotransferase, mitochondrial | DIV | -3.48 | -3.516 | -3.131 |
| CPIJ016642 | predicted protein | DIV | -3.172 | -2.268 | -1.552 |
| CPIJ004401 | ornithine aminotransferase, mitochondrial | DIV | -3.136 | -2.896 | -1.33 |
| CPIJ007628 | Juvenile hormone-inducible protein | DIV | -3.006 | -3.889 | -2.727 |
| CPIJ006617 | conserved hypothetical protein | DIV | -2.913 | -3.786 | -4.282 |
| CPIJ004019 | saccharopine dehydrogenase domain-containing protein | DIV | -2.782 | -3.965 | -2.314 |
| CPIJ002835 | serine-pyruvate aminotransferase | DIV | -2.265 | -2.852 | -3.553 |
| CPIJ003807 | allantoinase | DIV | -2.263 | -3.305 | -3.922 |
| CPIJ001265 | alkaline phosphatase | DIV | -2.128 | -1.845 | -2.186 |
| CPIJ002322 | conserved hypothetical protein | DIV | -2.046 | -1.96 | -2.549 |
| CPIJ001264 | alkaline phosphatase | DIV | -1.905 | -2.658 | -2.274 |
| CPIJ005558 | conserved hypothetical protein | DIV | -1.858 | -2.223 | -1.264 |
| CPIJ015241 | alkaline phosphatase | DIV | -1.809 | -3.601 | -3.781 |
| CPIJ015060 | conserved hypothetical protein | DIV | -1.77 | -2.263 | -2.036 |
| CPIJ015130 | beta-1,3-galactosyltransferase | DIV | -1.743 | -2.19 | -1.129 |
| CPIJ005644 | lethal | DIV | -1.74 | 5.033 | 3.373 |
| CPIJ016992 | conserved hypothetical protein | DIV | -1.722 | -2.057 | -1.087 |
| CPIJ005515 | conserved hypothetical protein | DIV | -1.71 | -2.107 | -1.534 |
| CPIJ004310 | enolase | DIV | -1.697 | -2.002 | -1.057 |
| CPIJ017111 | conserved hypothetical protein | DIV | -1.696 | -1.93 | -1.259 |
| CPIJ015282 | dopamine N acetyltransferase | DIV | -1.686 | -2.246 | -1.181 |
| CPIJ002430 | cytidine deaminase | DIV | -1.641 | -2.807 | -1.538 |
| CPIJ003550 | 78 kDa glucose-regulated protein | DIV | -1.63 | -1.429 | 2.066 |
| CPIJ016991 | conserved hypothetical protein | DIV | -1.616 | -1.923 | -1.132 |
| CPIJ007938 | asparagine synthetase B | DIV | -1.602 | -1.493 | -0.819 |
| CPIJ000948 | enolase | DIV | -1.599 | -2.058 | -0.774 |
| CPIJ011702 | enolase | DIV | -1.595 | -1.836 | -0.962 |
| CPIJ011350 | conserved hypothetical protein | DIV | -1.535 | -1.973 | -1.03 |
| CPIJ005519 | enolase | DIV | -1.473 | -1.721 | -0.793 |
| CPIJ012990 | allantoicase | DIV | -1.398 | -2.917 | -3.082 |
| CPIJ003610 | conserved hypothetical protein | DIV | -1.35 | -1.804 | -1.168 |
| CPIJ010375 | conserved hypothetical protein | DIV | -1.34 | -1.809 | -1.154 |
| CPIJ004284 | sulfide quinone reductase | DIV | -1.329 | -2.401 | -1.286 |
| CPIJ000277 | conserved hypothetical protein | DIV | -1.328 | -2.041 | -2.238 |
| CPIJ013577 | selenium-binding protein 2 | DIV | -1.303 | -3.304 | -2.651 |
| CPIJ015893 | disulfide isomerase | DIV | -1.297 | -2.119 | -0.936 |
| CPIJ013881 | conserved hypothetical protein | DIV | -1.286 | -2.048 | -1.843 |
| CPIJ010151 | dihydropyrimidinase | DIV | -1.284 | -1.658 | -1.645 |
| CPIJ002256 | spermine synthase | DIV | -1.274 | -2.48 | -1.653 |
| CPIJ004088 | guanylyl cyclase receptor | DIV | -1.271 | -2.327 | -2.939 |
| CPIJ019318 | conserved hypothetical protein | DIV | -1.25 | -1.695 | -0.954 |
| CPIJ007516 | thiamine transporter 1 | DIV | -1.226 | -1.52 | -1.981 |
| CPIJ005468 | adenosine diphosphatase | DIV | -1.224 | -0.835 | -0.782 |
| CPIJ009744 | conserved hypothetical protein | DIV | -1.165 | -2.347 | -2.591 |
| CPIJ004454 | conserved hypothetical protein | DIV | -1.163 | -0.875 | 0.754 |
| CPIJ015662 | ecto ADP-ribosylhydrolase | DIV | -1.133 | -2.246 | -1.372 |
| CPIJ013731 | conserved hypothetical protein | DIV | -1.126 | -1.882 | -1.353 |
| CPIJ005907 | conserved hypothetical protein | DIV | -1.104 | -0.907 | -1.415 |
| CPIJ005258 | conserved hypothetical protein | DIV | -1.097 | -0.816 | -0.792 |
| CPIJ003586 | proteasome subunit alpha type 3 | DIV | -1.088 | -1.716 | -0.841 |
| CPIJ005657 | phospholipid scramblase 1 | DIV | -1.086 | -1.751 | -1.581 |
| CPIJ013737 | conserved hypothetical protein | DIV | -1.076 | -2.194 | -2.346 |
| CPIJ006031 | conserved hypothetical protein | DIV | -1.072 | -1.656 | -1.766 |
| CPIJ004455 | sulfite reductase | DIV | -1.06 | -1.747 | -1.687 |
| CPIJ010893 | proteasome subunit alpha type 4 | DIV | -1.017 | -1.952 | -0.851 |
| CPIJ015587 | oligosaccharyl transferase | DIV | -1.005 | -1.438 | -0.793 |
| CPIJ004924 | LRR Toll | DIV | -0.992 | -1.096 | -1.307 |
| CPIJ002047 | dystrobrevin | DIV | -0.983 | -1.111 | -1.376 |
| CPIJ016908 | conserved hypothetical protein | DIV | -0.954 | -0.832 | -0.874 |
| CPIJ000840 | conserved hypothetical protein | DIV | -0.953 | -0.909 | -0.982 |
| CPIJ011177 | GPI mannosyltransferase 1 | DIV | -0.914 | -1.788 | -0.962 |
| CPIJ008512 | conserved hypothetical protein | DIV | -0.913 | -1.373 | -0.873 |
| CPIJ003397 | aldo-keto reductase | DIV | -0.907 | -1.911 | -1.199 |
| CPIJ014532 | 2-oxoisovalerate dehydrogenase subunit beta, mitochondrial | DIV | -0.895 | -1.509 | -0.855 |
| CPIJ008531 | lactase-phlorizin hydrolase | DIV | -0.891 | -2.444 | -3.052 |
| CPIJ017624 | glyoxylase | DIV | -0.886 | -1.954 | -2.101 |
| CPIJ004020 | saccharopine dehydrogenase domain-containing protein | DIV | -0.884 | -1.885 | -1.857 |
| CPIJ010531 | ABC1 family protein | DIV | -0.866 | 1.049 | 1.989 |
| CPIJ013882 | heat shock protein 67B2 | DIV | -0.862 | -1.896 | -1.453 |
| CPIJ017079 | conserved hypothetical protein | DIV | -0.85 | -1.289 | -0.853 |
| CPIJ008352 | conserved hypothetical protein | DIV | -0.82 | -1.811 | -1.662 |
| CPIJ011711 | conserved hypothetical protein | DIV | -0.817 | -1.022 | -0.948 |
| CPIJ001740 | mannose-P-dolichol utilization defect 1 protein | DIV | -0.814 | -1.742 | -0.962 |
| CPIJ011388 | diazepam binding inhibitor | DIV | -0.811 | -1.964 | -1.774 |
| CPIJ019133 | quinone oxidoreductase | DIV | -0.778 | -1.236 | -1.058 |
| CPIJ018121 | membrane-bound alkaline phosphatase | DIV | -0.769 | -2.636 | -2.149 |
| CPIJ019557 | conserved hypothetical protein | DIV | -0.756 | -0.807 | -0.781 |
| CPIJ002913 | conserved hypothetical protein | DIV | 0.761 | 1.177 | 1.174 |
| CPIJ013950 | conserved hypothetical protein | DIV | 0.771 | 1.203 | 1.145 |
| CPIJ013746 | kinase | DIV | 0.785 | 0.911 | 1.2 |
| CPIJ014022 | coronin | DIV | 0.791 | 1.659 | 1.389 |
| CPIJ003158 | G protein-coupled receptor | DIV | 0.8 | 0.971 | 1.423 |
| CPIJ004200 | serine/threonine-protein kinase 40 | DIV | 0.808 | 1.459 | 2.117 |
| CPIJ006689 | conserved hypothetical protein | DIV | 0.813 | 0.997 | 0.815 |
| CPIJ002003 | conserved hypothetical protein | DIV | 0.819 | 1.258 | 1.017 |
| CPIJ017084 | conserved hypothetical protein | DIV | 0.819 | 1.021 | 1.235 |
| CPIJ011659 | conserved hypothetical protein | DIV | 0.833 | 1.018 | 1.018 |
| CPIJ013723 | dimethylaniline monooxygenase | DIV | 0.844 | 1.485 | 1.161 |
| CPIJ000182 | N-acetyl neuraminate lyase | DIV | 0.858 | -1.144 | -1.644 |
| CPIJ006276 | F-box/WD repeat protein 5 | DIV | 0.866 | 0.807 | 0.755 |
| CPIJ005048 | conserved hypothetical protein | DIV | 0.87 | 1.06 | 1.011 |
| CPIJ007773 | conserved hypothetical protein | DIV | 0.877 | 1.717 | 1.967 |
| CPIJ017966 | conserved hypothetical protein | DIV | 0.881 | 0.771 | 0.841 |
| CPIJ009089 | ras-related protein Rab-7 | DIV | 0.902 | 1.111 | 0.852 |
| CPIJ007837 | zinc finger protein | DIV | 0.918 | 2.376 | 1.019 |
| CPIJ009050 | lkb1 interacting protein | DIV | 0.92 | 0.903 | 0.861 |
| CPIJ009404 | trehalose-6-phosphate synthase | DIV | 0.924 | -1.299 | -1.164 |
| CPIJ017297 | quinone oxidoreductase | DIV | 0.93 | 2.19 | 1.872 |
| CPIJ015482 | conserved hypothetical protein | DIV | 0.938 | 0.914 | 1.667 |
| CPIJ000921 | menin | DIV | 0.947 | 1.231 | 0.926 |
| CPIJ003516 | signal transducing adapter molecule 1 | DIV | 0.961 | 1.343 | 1.542 |
| CPIJ016906 | conserved hypothetical protein | DIV | 0.963 | 1.618 | 1.466 |
| CPIJ002340 | conserved hypothetical protein | DIV | 0.965 | 1.075 | 0.974 |
| CPIJ004017 | cyclin l | DIV | 0.969 | 1.297 | 1.156 |
| CPIJ002519 | conserved hypothetical protein | DIV | 0.978 | 2.371 | 1.931 |
| CPIJ007154 | conserved hypothetical protein | DIV | 0.993 | 1.898 | 1.474 |
| CPIJ008259 | hypothetical protein | DIV | 0.994 | 1.144 | 0.899 |
| CPIJ019184 | conserved hypothetical protein | DIV | 1.046 | 1.231 | 0.848 |
| CPIJ005646 | alphaA-crystallin | DIV | 1.052 | 4.245 | 3.782 |
| CPIJ015901 | conserved hypothetical protein | DIV | 1.061 | 1.738 | 1.445 |
| CPIJ002707 | zinc finger protein | DIV | 1.065 | 1.588 | 0.988 |
| CPIJ000895 | conserved hypothetical protein | DIV | 1.073 | 1.267 | 1.339 |
| CPIJ015947 | NULL | DIV | 1.109 | 1.06 | 0.822 |
| CPIJ003793 | BTB/POZ domain-containing protein 9 | DIV | 1.111 | 1.392 | 1.178 |
| CPIJ002448 | lipoma preferred partner/lpp | DIV | 1.116 | 1.54 | 1.496 |
| CPIJ001300 | zinc finger protein 780B | DIV | 1.131 | 1.143 | 0.915 |
| CPIJ011609 | conserved hypothetical protein | DIV | 1.152 | 1.79 | 2.055 |
| CPIJ008919 | tyrosine phosphatase n9 | DIV | 1.168 | 1.698 | 1.324 |
| CPIJ000493 | conserved hypothetical protein | DIV | 1.176 | 1.738 | 1.798 |
| CPIJ019535 | imaginal discs arrested | DIV | 1.186 | 1.223 | 0.884 |
| CPIJ016941 | conserved hypothetical protein | DIV | 1.19 | 1.217 | 1.023 |
| CPIJ017857 | ATPase WRNIP1 | DIV | 1.204 | 1.082 | 0.814 |
| CPIJ008851 | maltose phosphorylase | DIV | 1.209 | 1.963 | 1.535 |
| CPIJ000181 | schnurri | DIV | 1.211 | 1.464 | 1.155 |
| CPIJ003582 | TATA-binding protein-associated factor 172 | DIV | 1.211 | 1.684 | 2.344 |
| CPIJ000595 | conserved hypothetical protein | DIV | 1.238 | 1.412 | 1.01 |
| CPIJ008853 | maltose phosphorylase | DIV | 1.255 | 3.049 | 2.574 |
| CPIJ006113 | internalin A | DIV | 1.257 | 1.621 | 1.867 |
| CPIJ002498 | lysosomal thioesterase PPT2 | DIV | 1.262 | 1.123 | 1.046 |
| CPIJ002978 | aminotransferase | DIV | 1.264 | 1.688 | 1.227 |
| CPIJ009633 | conserved hypothetical protein | DIV | 1.277 | 1.354 | 1.179 |
| CPIJ008182 | L47123_1 ferritin | DIV | 1.33 | 1.823 | 1.56 |
| CPIJ012373 | virus-induced RNA | DIV | 1.335 | 1.468 | 2.126 |
| CPIJ005661 | leucine rich protein | DIV | 1.352 | 2.951 | 1.441 |
| CPIJ016890 | sans | DIV | 1.362 | 1.56 | 1.849 |
| CPIJ016766 | conserved hypothetical protein | DIV | 1.368 | 1.57 | 1.167 |
| CPIJ003242 | conserved hypothetical protein | DIV | 1.392 | 2.566 | 2.339 |
| CPIJ006963 | conserved hypothetical protein | DIV | 1.442 | 1.939 | 2.129 |
| CPIJ007658 | werner syndrome helicase | DIV | 1.442 | 1.819 | 1.063 |
| CPIJ016927 | conserved hypothetical protein | DIV | 1.443 | 1.33 | 0.79 |
| CPIJ007754 | rac serine/threonine kinase | DIV | 1.464 | 1.716 | 1.589 |
| CPIJ008852 | maltose phosphorylase | DIV | 1.486 | 2.124 | 1.545 |
| CPIJ007659 | conserved hypothetical protein | DIV | 1.495 | 1.523 | 0.817 |
| CPIJ018976 | 1-acylglycerol-3-phosphate acyltransferase | DIV | 1.503 | 1.938 | 1.488 |
| CPIJ010173 | testis-specific protein pbs13 | DIV | 1.526 | 1.929 | 1.697 |
| CPIJ005745 | c3f | DIV | 1.53 | 1.191 | 1.045 |
| CPIJ012839 | 1-acylglycerol-3-phosphate acyltransferase | DIV | 1.572 | 1.385 | 1.001 |
| CPIJ014100 | conserved hypothetical protein | DIV | 1.586 | 2.238 | 1.561 |
| CPIJ015211 | imaginal discs arrested | DIV | 1.619 | 1.892 | 1.492 |
| CPIJ003456 | uricase | DIV | 1.652 | 2.073 | 2.641 |
| CPIJ005640 | heat shock protein 26 | DIV | 1.682 | 3.654 | 3.283 |
| CPIJ002117 | conserved hypothetical protein | DIV | 1.704 | 1.733 | 1.787 |
| CPIJ005118 | fasciclin | DIV | 1.705 | 2.123 | 1.573 |
| CPIJ007747 | T01G9.2 | DIV | 1.711 | 1.826 | 0.962 |
| CPIJ015931 | regulator of g protein signaling | DIV | 1.717 | 1.398 | 0.757 |
| CPIJ009709 | conserved hypothetical protein | DIV | 1.723 | 3.287 | 2.006 |
| CPIJ005772 | trithorax protein ash2 | DIV | 1.804 | 1.439 | 0.901 |
| CPIJ000291 | conserved hypothetical protein | DIV | 1.826 | 1.86 | 0.803 |
| CPIJ001938 | BTG1 protein | DIV | 1.906 | 1.784 | 0.851 |
| CPIJ010972 | zinc finger protein | DIV | 2.026 | 1.237 | -0.774 |
| CPIJ006312 | conserved hypothetical protein | DIV | 2.146 | 2.423 | 1.928 |
| CPIJ007984 | f-box/lrr protein | DIV | 2.318 | 2.732 | 1.69 |
| CPIJ015408 | branched-chain amino acid aminotransferase | DIV | 2.35 | 3.462 | 2.552 |
| CPIJ008106 | conserved hypothetical protein | DIV | 2.362 | 0.778 | -0.931 |
| CPIJ003202 | conserved hypothetical protein | DIV | 2.391 | 2.329 | 3.136 |
| CPIJ014573 | phenylalanyl-tRNA synthetase beta chain | DIV | 2.57 | 3.012 | 1.065 |
| CPIJ011365 | E3 ubiquitin-protein ligase mib1 | DIV | 2.729 | 1.864 | 1.186 |
| CPIJ013070 | phenylalanyl-tRNA synthetase beta chain | DIV | 2.73 | 3.131 | 1.096 |
| CPIJ008571 | UDP-glucuronosyltransferase | DIV | 3.077 | 2.918 | 2.211 |
| CPIJ013215 | conserved hypothetical protein | DIV | 3.429 | 3.133 | 1.763 |
| CPIJ000210 | cysteine-rich venom protein | DIV | 4.471 | 4.706 | 3.898 |
| CPIJ009451 | conserved hypothetical protein | DIV | 4.471 | 4.336 | 2.8 |
| CPIJ014453 | conserved hypothetical protein | DIV, CSR | 1.145 | 1.547 | 1.054 |
| CPIJ018042 | conserved hypothetical protein | DIV, CST | 0.882 | 2.011 | 1.398 |
| CPIJ006917 | NFkappaB essential modulator | DIV, IMM | 1.044 | 1.351 | 1.419 |
| CPIJ004285 | neural/ectodermal development factor IMP-L2 | DIV, IMM | 1.788 | 3.996 | 4.078 |
| CPIJ007064 | conserved hypothetical protein | DIV, MET | -0.79 | -1.663 | -1.135 |
| CPIJ007385 | serine-type enodpeptidase | DIV, PRT, MET, IMM, DIG | -3.048 | -3.381 | -1.202 |
| CPIJ007384 | azurocidin | DIV, PRT, MET, IMM, DIG, TRP | -1.786 | -2.294 | -1.451 |
| CPIJ018034 | brachyurin | DIV, PRT, MET, IMM, DIG, TRP | -0.804 | -1.643 | -0.972 |
| CPIJ009112 | plasminogen | DIV, PRT, MET, IMM, DIG, TRP | 1.357 | 1.029 | 1.095 |
| CPIJ005271 | trypsin 2 | DIV, PRT, MET, IMM, DIG, TRP | 2.56 | 3.426 | 1.809 |
| CPIJ003393 | aldose reductase | DIV, RSM | -1.59 | -2.601 | -1.982 |
| CPIJ017844 | Tetratricopeptide repeat protein | DIV, RSM | -1.519 | -1.492 | 1.089 |
| CPIJ019794 | prov protein | DIV, RSM | -1.126 | -1.077 | 0.849 |
| CPIJ000098 | electron transfer flavoprotein-ubiquinone oxidoreductase | DIV, RSM | -0.79 | -1.848 | -0.936 |
| CPIJ009607 | malic enzyme | DIV, RSM | 1.74 | 1.596 | 0.77 |
| CPIJ018127 | conserved hypothetical protein | DIV, RTT | 0.761 | 0.998 | 0.787 |
| CPIJ007769 | testis development protein prtd | DIV, RTT | 0.824 | 0.844 | 0.848 |
| CPIJ000084 | jun | DIV, RTT | 0.981 | 1.449 | 1.042 |
| CPIJ011082 | heat shock protein 70 B2 | DIV, RTT | 1.293 | 3.434 | 3.217 |
| CPIJ011725 | conserved hypothetical protein | DIV, RTT | 2.611 | 2.452 | 1.437 |
| CPIJ006361 | equilibrative nucleoside transporter | DIV, TRP | -1.081 | -1.56 | -1.276 |
| CPIJ011542 | synaptic vesicle protein | DIV, TRP | -0.888 | -1.511 | -1.32 |
| CPIJ004127 | macroglobulin/complement | IMM | -2.228 | -3.349 | -2.566 |
| CPIJ009032 | larval serum protein 2 | IMM | -2.176 | -3.039 | -3.473 |
| CPIJ008450 | peroxiredoxin-6 | IMM | -2.161 | -4.72 | -3.555 |
| CPIJ009506 | hexamerin 2 beta | IMM | -1.931 | -3.591 | -4.574 |
| CPIJ001276 | defensin-A | IMM | -1.808 | -1.86 | -2.2 |
| CPIJ004325 | gram-negative bacteria binding protein | IMM | -1.79 | -1.728 | -1.287 |
| CPIJ019787 | ficolin-3 | IMM | -1.747 | -1.962 | -1.466 |
| CPIJ003103 | croquemort | IMM | -1.694 | -1.646 | -2.108 |
| CPIJ006392 | fibrinogen and fibronectin | IMM | -1.562 | -1.587 | -1.1 |
| CPIJ010699 | cecropin A | IMM | -1.305 | -1.149 | -1.761 |
| CPIJ001239 | cathepsin B | IMM | -1.274 | -3.74 | -2.951 |
| CPIJ017039 | cd36 antigen | IMM | -1.218 | -1.026 | -0.962 |
| CPIJ000195 | conserved hypothetical protein | IMM | -1.213 | -1.397 | -0.889 |
| CPIJ006716 | peptidoglycan recognition protein sb2 | IMM | -1.189 | 1.488 | 2.207 |
| CPIJ007579 | peroxidase | IMM | -1.099 | -1.94 | -1.528 |
| CPIJ017588 | peroxidase | IMM | -1.066 | -1.91 | -1.359 |
| CPIJ006014 | serine protease | IMM | -1.055 | -0.988 | -1.036 |
| CPIJ004320 | gram-negative bacteria-binding protein 1 | IMM | -1.053 | -2.814 | -3.981 |
| CPIJ012830 | fibrinogen and fibronectin | IMM | -1.038 | 2.351 | -2.545 |
| CPIJ010092 | ficolin-3 | IMM | -0.964 | -1.477 | -1.226 |
| CPIJ004947 | leucine-rich repeat-containing protein 1 | IMM | -0.908 | -2.155 | -1.408 |
| CPIJ004719 | superoxide dismutase 3.4, mitochondrial | IMM | -0.894 | -2.095 | -1.314 |
| CPIJ008014 | oxidase/peroxidase | IMM | -0.827 | -1.607 | -0.773 |
| CPIJ002382 | Misexpression suppressor of KSR | IMM | -0.755 | -0.936 | -1.008 |
| CPIJ012013 | serine protease inhibitor, serpin | IMM | 0.831 | 2.865 | 1.92 |
| CPIJ004894 | bax inhibitor | IMM | 0.837 | 0.976 | 0.856 |
| CPIJ003380 | suppressorsof cytokine signalling | IMM | 0.845 | 1.31 | 1.396 |
| CPIJ008999 | leucine-rich transmembrane protein | IMM | 0.977 | 1.025 | 1.078 |
| CPIJ002658 | clip-domain serine protease | IMM | 0.986 | 0.792 | 1.175 |
| CPIJ013339 | transferrin | IMM | 1.03 | 0.983 | 1.355 |
| CPIJ013424 | PIWI | IMM | 1.054 | 1.99 | 1.654 |
| CPIJ014718 | serine protease inhibitor 4, serpin-4 | IMM | 1.266 | 2.704 | 2.604 |
| CPIJ007783 | arylphorin subunit alpha | IMM | 1.312 | -1.19 | -1.091 |
| CPIJ015701 | 32 kDa beta-galactoside-binding lectin lec-3 | IMM | 1.606 | 1.573 | 1.435 |
| CPIJ007535 | adhesive serine protease | IMM | 1.921 | 1.863 | 2.664 |
| CPIJ006471 | cathepsin l | IMM | 1.925 | 1.79 | 1.166 |
| CPIJ018481 | yellow | IMM | 2.274 | 1.224 | 1.84 |
| CPIJ001385 | glutamate decarboxylase | IMM, MET | -1.376 | -2.069 | -1.771 |
| CPIJ009057 | caspase-3 | IMM, MET | 0.807 | 1.743 | 1.979 |
| CPIJ007035 | lipase | MET | -4.413 | -4.576 | -1.174 |
| CPIJ004373 | UDP-glucuronosyltransferase | MET | -2.874 | -3.369 | -1.887 |
| CPIJ001556 | acyl-coenzyme A oxidase 3 | MET | -2.45 | -3.944 | -3.673 |
| CPIJ010973 | glutamine synthetase 1, mitochondrial | MET | -2.395 | -2.297 | -1.087 |
| CPIJ019917 | triacylglycerol lipase | MET | -2.352 | -2.257 | -1.498 |
| CPIJ011600 | long-chain-fatty-acid coa ligase | MET | -2.306 | -2.341 | -1.413 |
| CPIJ012752 | cysteine synthase | MET | -2.175 | -2.509 | -1.343 |
| CPIJ019470 | 3-demethylubiquinone-9 3-methyltransferase | MET | -2.159 | -2.698 | -2.008 |
| CPIJ020058 | pyridoxal kinase | MET | -2.157 | -2.604 | -1.481 |
| CPIJ002354 | triosephosphate isomerase | MET | -2.119 | -2.513 | -1.348 |
| CPIJ009280 | phosphoglycerate kinase | MET | -2.112 | -1.809 | -0.95 |
| CPIJ000226 | glucosyl/glucuronosyl transferase | MET | -2.061 | -1.374 | -1.406 |
| CPIJ004138 | 1-acyl-sn-glycerol-3-phosphate acyltransferase | MET | -1.957 | -2.624 | -1.703 |
| CPIJ001564 | selenide | MET | -1.589 | -1.914 | -1.49 |
| CPIJ003185 | gamma-glutamyltranspeptidase 1 | MET | -1.56 | -1.979 | -2.632 |
| CPIJ003693 | glucosyl/glucuronosyl transferase | MET | -1.551 | -2.821 | -2.466 |
| CPIJ003695 | glucosyl/glucuronosyl transferase | MET | -1.545 | -3.22 | -3.644 |
| CPIJ006245 | coproporphyrinogen III oxidase | MET | -1.528 | -2.58 | -1.459 |
| CPIJ000372 | L-xylulose reductase | MET | -1.518 | -2.94 | -2.249 |
| CPIJ000225 | UDP-glucuronosyltransferase R-21 | MET | -1.506 | -1.96 | -1.183 |
| CPIJ010716 | luciferin 4-monooxygenase | MET | -1.437 | -2.747 | -2.378 |
| CPIJ001998 | 1-acyl-sn-glycerol-3-phosphate acyltransferase | MET | -1.383 | -2.087 | -1.352 |
| CPIJ010456 | cysteine dioxygenase | MET | -1.377 | -1.924 | -1.144 |
| CPIJ009094 | ornithine decarboxylase 1 | MET | -1.376 | -3.737 | -3.588 |
| CPIJ003841 | methylcrotonoyl-CoA carboxylase alpha chain, mitochondrial | MET | -1.371 | -1.926 | -0.867 |
| CPIJ000351 | UDP-glucuronosyltransferase 2B28 | MET | -1.351 | -1.859 | -0.792 |
| CPIJ002148 | phenylalanine-4-hydroxylase | MET | -1.346 | 2.673 | 3.107 |
| CPIJ006068 | utp-glucose-1-phosphate uridylyltransferase 2 | MET | -1.319 | -1.436 | -1.151 |
| CPIJ008729 | 4-aminobutyrate aminotransferase, mitochondrial | MET | -1.28 | -2.199 | -1.816 |
| CPIJ006187 | acyl-CoA oxidase | MET | -1.263 | -2.045 | -2.236 |
| CPIJ007302 | long-chain fatty acid transport protein 4 | MET | -1.254 | -1.522 | -1.456 |
| CPIJ000128 | galactokinase | MET | -1.224 | -1.921 | -1.391 |
| CPIJ006323 | xaa-Pro aminopeptidase 1 | MET | -1.195 | -1.999 | -1.69 |
| CPIJ003432 | aldehyde dehydrogenase | MET | -1.194 | -2.765 | -2.723 |
| CPIJ015182 | conserved hypothetical protein | MET | -1.186 | -1.337 | -1.245 |
| CPIJ009093 | ornithine decarboxylase | MET | -1.184 | -3.049 | -2.169 |
| CPIJ004141 | 1-acyl-sn-glycerol-3-phosphate acyltransferase beta | MET | -1.141 | -1.615 | -1.369 |
| CPIJ002383 | bifunctional purine biosynthesis protein PURH | MET | -1.124 | -1.645 | -0.818 |
| CPIJ004319 | molybdenum cofactor synthesis protein cinnamon | MET | -1.107 | -1.638 | -0.873 |
| CPIJ015209 | 3-hydroxyisobutyrate dehydrogenase | MET | -1.076 | -2.485 | -1.843 |
| CPIJ009438 | aldehyde dehydrogenase | MET | -1.061 | -2.55 | -1.591 |
| CPIJ011465 | C-4 methylsterol oxidase | MET | -1.051 | -1.748 | -1.498 |
| CPIJ003061 | acyl-CoA oxidase | MET | -1.02 | -1.829 | -1.782 |
| CPIJ007527 | carbonic anhydrase | MET | -1.017 | -1.401 | -0.906 |
| CPIJ001655 | aldose 1-epimerase | MET | -0.986 | -1.284 | -1.332 |
| CPIJ002685 | enoyl-CoA hydratase ECHA12 | MET | -0.968 | -2.343 | -1.781 |
| CPIJ008783 | alcohol dehydrogenase class 3 | MET | -0.959 | -1.894 | -1.225 |
| CPIJ006508 | UDP-glucuronosyltransferase 2B4 | MET | -0.931 | -1.445 | -2.203 |
| CPIJ007660 | pyrroline-5-carboxylate dehydrogenase | MET | -0.91 | -1.363 | -1.185 |
| CPIJ009697 | glutathione synthetase | MET | -0.887 | -1.221 | -0.951 |
| CPIJ009306 | neutral alpha-glucosidase ab | MET | -0.886 | -3.387 | -2.904 |
| CPIJ000992 | juvenile hormone epoxide hydrolase 1 | MET | -0.86 | -2.714 | -2.802 |
| CPIJ020056 | cysteine dioxygenase | MET | -0.849 | -1.592 | -1.846 |
| CPIJ015409 | cleavage and polyadenylation specificity factor | MET | -0.825 | -1.068 | -0.905 |
| CPIJ011631 | short-chain specific acyl-CoA dehydrogenase, mitochondrial | MET | -0.801 | -1.498 | -1.127 |
| CPIJ012030 | 3-hydroxyisobutyryl-coenzyme A hydrolase | MET | -0.79 | -1.403 | -0.849 |
| CPIJ015417 | xylulose kinase | MET | -0.778 | -1.888 | -1.731 |
| CPIJ010515 | phosphoenolpyruvate carboxykinase | MET | -0.772 | 1.969 | 1.62 |
| CPIJ014557 | nitrilase and fragile histidine triad fusion protein NitFhit | MET | -0.772 | -1.038 | -1.007 |
| CPIJ011877 | NULL | MET | -0.765 | -1.885 | -1.671 |
| CPIJ002911 | retinoid-inducible serine carboxypeptidase | MET | -0.751 | -2.391 | -3.592 |
| CPIJ002958 | sterol o-acyltransferase | MET | 0.751 | 1.065 | 0.898 |
| CPIJ011996 | 10-formyltetrahydrofolate dehydrogenase | MET | 0.77 | 1.424 | 2.168 |
| CPIJ001337 | uridine 5'-monophosphate synthase | MET | 0.808 | 1.185 | 1.03 |
| CPIJ006770 | glucosamine-fructose-6-phosphate aminotransferase 2 | MET | 0.827 | 1.632 | 1.968 |
| CPIJ012841 | valacyclovir hydrolase | MET | 0.838 | 1.126 | 1.599 |
| CPIJ018167 | sterol regulatory element-binding protein 1 | MET | 0.851 | 1.394 | 1.559 |
| CPIJ005214 | ceramide glucosyltransferase | MET | 0.901 | 1.1 | 1.534 |
| CPIJ012679 | adam | MET | 0.931 | 2.576 | 2.833 |
| CPIJ011260 | phosphoglucomutase | MET | 0.933 | 1.231 | 1.026 |
| CPIJ006769 | glucosamine-fructose-6-phosphate aminotransferase 2 | MET | 0.963 | 1.755 | 2.267 |
| CPIJ013862 | phosphoglucomutase | MET | 0.966 | 1.354 | 0.891 |
| CPIJ019694 | ceramide glucosyltransferase | MET | 1.007 | 1.304 | 1.043 |
| CPIJ017812 | hexokinase | MET | 1.091 | 0.843 | 0.956 |
| CPIJ006164 | serine palmitoyltransferase 1 | MET | 1.111 | 1.173 | 0.756 |
| CPIJ008049 | hexokinase | MET | 1.151 | 1.092 | 0.864 |
| CPIJ008181 | inosine-uridine preferring nucleoside hydrolase | MET | 1.256 | 2.353 | 3.231 |
| CPIJ019120 | chitotriosidase-1 | MET | 1.363 | 1.083 | 0.821 |
| CPIJ017745 | sterol desaturase | MET | 1.443 | 0.948 | 1.194 |
| CPIJ003090 | cullin | MET | 1.551 | 1.99 | 1.214 |
| CPIJ008074 | glucosamine-6-phosphate isomerase | MET | 1.568 | 0.82 | 0.83 |
| CPIJ004967 | uracil phosphoribosyltransferase | MET | 1.602 | 1.978 | 1.542 |
| CPIJ016639 | acetyl-CoA synthetase | MET | 1.662 | 1.931 | 2.59 |
| CPIJ005543 | phosphatidylinositol 3-kinase 1 | MET | 1.747 | 2.068 | 1.094 |
| CPIJ018208 | cholinephosphate cytidylyl transferase B2 | MET | 2.047 | 2.549 | 1.337 |
| CPIJ013573 | N-acetyl galactosaminyl transferase 6 | MET | 2.066 | 2.34 | 0.842 |
| CPIJ005231 | N-acetyl galactosaminyl transferase 6 | MET | 2.301 | 2.572 | 1.013 |
| CPIJ002959 | sterol O-acyltransferase 2 | MET | 2.379 | 2.153 | 1.688 |
| CPIJ015715 | AMP dependent ligase | MET | 2.438 | 3.933 | 3.155 |
| CPIJ005348 | lipase 3 | MET | 2.573 | 3.598 | 3.116 |
| CPIJ003304 | isocitrate dehydrogenase | MET, DIV | -0.83 | -2.882 | -2.723 |
| CPIJ010432 | acyl-coenzyme A thioesterase 9 | MET, DIV | 0.86 | 0.873 | 1.728 |
| CPIJ013377 | sorbitol dehydrogenase | MET, DIV | 1.452 | -0.755 | -1.754 |
| CPIJ008878 | prolylcarboxypeptidase | MET, PRT | -3.9 | -3.218 | -1.034 |
| CPIJ008716 | tubulointerstitial nephritis antigen | MET, PRT | 1.782 | 1.983 | 2.268 |
| CPIJ007204 | apolipophorins | MET, TRP | -1.902 | -2.746 | -2.325 |
| CPIJ019029 | metalloproteinase | PRT | -1.997 | -1.397 | -1.344 |
| CPIJ008873 | prolylcarboxypeptidase | PRT | -1.879 | -3.401 | -2.623 |
| CPIJ013671 | aminoacylase | PRT | -1.544 | -0.884 | -1.687 |
| CPIJ013669 | aminoacylase | PRT | -1.506 | -1.68 | -1.56 |
| CPIJ001240 | cathepsin B-like thiol protease | PRT | -1.452 | -3.876 | -3.431 |
| CPIJ008379 | conserved hypothetical protein | PRT | -1.183 | -1.753 | -2.741 |
| CPIJ008387 | aminopeptidase N | PRT | -1.159 | -2.168 | -1.359 |
| CPIJ004060 | aminopeptidase N | PRT | -1.053 | -2.59 | -2.512 |
| CPIJ008264 | proteasome subunit beta type 7 | PRT | -0.953 | -1.433 | -0.863 |
| CPIJ013670 | aminoacylase | PRT | -0.949 | -1.017 | -0.816 |
| CPIJ002133 | trypsin epsilon | PRT | -0.892 | -2.737 | -3.182 |
| CPIJ015253 | zinc carboxypeptidase A 1 | PRT | -0.866 | -2.117 | -1.447 |
| CPIJ001743 | carboxypeptidase A2 | PRT | -0.766 | -2.291 | -3.767 |
| CPIJ007722 | hypothetical protein | PRT | 1.499 | 0.822 | -0.89 |
| CPIJ003994 | serine collagenase 1 | PRT | 1.549 | 2.246 | 1.651 |
| CPIJ006544 | chymotrypsinogen 2 | PRT | 5.478 | 4.719 | 0.799 |
| CPIJ008031 | conserved hypothetical protein | PRT, DIV | 1.829 | 2.053 | 1.342 |
| CPIJ002136 | serine protease1/2 | PRT, IMM, MET | -1.344 | -1.933 | -0.903 |
| CPIJ012743 | 60S ribosomal protein L7 | PRT, RTT | 0.945 | 1.085 | 0.894 |
| CPIJ000318 | cytochrome b5 | RSM | -2.959 | -3.198 | -3.479 |
| CPIJ019024 | cytochrome c | RSM | -2.279 | -2.817 | -0.993 |
| CPIJ015075 | heat shock protein 83 | RSM | -2.203 | -1.732 | 1.417 |
| CPIJ001380 | cytochrome P450 | RSM | -2.135 | -1.589 | -1.301 |
| CPIJ004599 | 3-oxoacyl-[acyl-carrier-protein] reductase | RSM | -2.125 | -3.115 | -2.718 |
| CPIJ005899 | cytochrome P450 6A1 | RSM | -2.018 | -3.008 | -2.919 |
| CPIJ009415 | cytochrome P450 4g15 | RSM | -2.01 | -2.714 | -1.465 |
| CPIJ010544 | cytochrome P450 9b2 | RSM | -2.007 | -3.336 | -4.037 |
| CPIJ010543 | cytochrome P450 17A1 | RSM | -1.998 | -2.31 | -1.61 |
| CPIJ016922 | serine 3-dehydrogenase | RSM | -1.988 | -2.902 | -2.66 |
| CPIJ005655 | oxidoreductase | RSM | -1.967 | -3.469 | -3.808 |
| CPIJ002537 | cytochrome P450 26B1 | RSM | -1.951 | -4.23 | -4.902 |
| CPIJ019572 | glutathione transferase AtGST | RSM | -1.861 | -2.301 | -1.794 |
| CPIJ010542 | cytochrome P450 1A1 | RSM | -1.804 | -3 | -2.761 |
| CPIJ013917 | esterase B1 | RSM | -1.804 | -2.705 | -2.169 |
| CPIJ009186 | complement component | RSM | -1.803 | -2.36 | -0.915 |
| CPIJ007010 | peroxisomal membrane protein pmp34 | RSM | -1.744 | -1.935 | -2.146 |
| CPIJ001758 | cytochrome P450 4d1 | RSM | -1.719 | -3.335 | -3.259 |
| CPIJ010858 | cytochrome P450 6a22 | RSM | -1.691 | -3.644 | -4.01 |
| CPIJ016921 | 3-oxoacyl-[acyl-carrier-protein] reductase 1 | RSM | -1.64 | -1.753 | -1.102 |
| CPIJ000670 | 24-dehydrocholesterol reductase | RSM | -1.596 | -2.586 | -2.095 |
| CPIJ016857 | cytochrome P450 | RSM | -1.523 | -3.03 | -2.511 |
| CPIJ004600 | oxidoreductase | RSM | -1.495 | -2.524 | -2.686 |
| CPIJ011246 | heat shock protein 83 | RSM | -1.495 | -1.429 | 1.004 |
| CPIJ020199 | cytochrome P450 | RSM | -1.465 | -2.04 | -1.693 |
| CPIJ001886 | cytochrome P450 4C1 | RSM | -1.411 | -2.384 | -2.546 |
| CPIJ005957 | cytochrome P450 | RSM | -1.404 | -3.188 | -2.359 |
| CPIJ011127 | cytochrome P450 4d1 | RSM | -1.391 | -1.078 | -1.551 |
| CPIJ004365 | xanthine dehydrogenase | RSM | -1.357 | -2.354 | -2.231 |
| CPIJ008445 | amine oxidase | RSM | -1.351 | -1.801 | -0.885 |
| CPIJ018624 | glutathione-s-transferase theta, gst | RSM | -1.341 | -1.669 | -1.139 |
| CPIJ011244 | heat shock protein 83 | RSM | -1.329 | -1.303 | 0.849 |
| CPIJ007829 | alpha-esterase | RSM | -1.316 | -1.702 | -0.958 |
| CPIJ007943 | peroxiredoxin 5, prdx5 | RSM | -1.297 | -2.032 | -1.663 |
| CPIJ010904 | conserved hypothetical protein | RSM | -1.295 | -2.428 | -1.239 |
| CPIJ016855 | cytochrome P450 4A6 | RSM | -1.282 | -2 | -1.907 |
| CPIJ016341 | alpha-esterase | RSM | -1.28 | -1.642 | -1.272 |
| CPIJ018633 | glutathione-s-transferase theta | RSM | -1.273 | -2.04 | -1.107 |
| CPIJ004637 | glutactin | RSM | -1.262 | -2.096 | -1.907 |
| CPIJ019817 | cytochrome c oxidase assembly protein COX19 | RSM | -1.261 | -1.536 | -1.067 |
| CPIJ006159 | glutathione-requiring prostaglandin D synthase | RSM | -1.251 | -1.868 | -2.174 |
| CPIJ005953 | cytochrome P450 CYP6BB1v2 | RSM | -1.209 | -2.341 | -2.129 |
| CPIJ015280 | cytochrome c oxidase assembly protein COX11, mitochondrial | RSM | -1.2 | -1.907 | -1.44 |
| CPIJ015053 | mitochondrial 28S ribosomal protein S25 | RSM | -1.169 | -1.581 | -0.987 |
| CPIJ009733 | glucose 1-dehydrogenase 2 | RSM | -1.168 | -2.773 | -2.27 |
| CPIJ018241 | microsomal glutathione S-transferase 1 | RSM | -1.122 | -1.476 | -1.38 |
| CPIJ011017 | heat shock protein | RSM | -1.121 | -1.973 | -0.9 |
| CPIJ010545 | cytochrome P450 9b1 | RSM | -1.1 | -2.768 | -3.382 |
| CPIJ005531 | conserved hypothetical protein | RSM | -1.022 | -1.9 | -2.141 |
| CPIJ016356 | cytochrome P450 6a9 | RSM | -0.993 | -1.545 | -1.805 |
| CPIJ004595 | cytochrome b5 | RSM | -0.966 | -1.184 | -0.878 |
| CPIJ001759 | cytochrome P450 4c21 | RSM | -0.964 | -2.357 | -1.758 |
| CPIJ008190 | 39S ribosomal protein L14, mitochondrial | RSM | -0.958 | -1.305 | -1.054 |
| CPIJ014579 | cytochrome P450 4d1 | RSM | -0.927 | -1.557 | -1.074 |
| CPIJ019179 | ninjurin a | RSM | -0.915 | -2.162 | -1.749 |
| CPIJ001757 | cytochrome P450 4d1 | RSM | -0.895 | -2.139 | -1.992 |
| CPIJ010075 | cytochrome P450 4c3 | RSM | -0.894 | -1.521 | -1.264 |
| CPIJ019705 | cytochrome P450 6a22 | RSM | -0.829 | -1.534 | -1.239 |
| CPIJ016854 | cytochrome P450 | RSM | -0.812 | -2.348 | -1.899 |
| CPIJ010934 | conserved hypothetical protein | RSM | -0.807 | -1.07 | -1.093 |
| CPIJ007225 | 3-ketodihydrosphingosine reductase | RSM | -0.78 | -1.107 | -0.866 |
| CPIJ003709 | thioredoxin, mitochondrial | RSM | -0.775 | -1.424 | -0.925 |
| CPIJ016339 | liver carboxylesterase 1 | RSM | -0.771 | -0.814 | -0.775 |
| CPIJ018494 | cytochrome P450 6B1 | RSM | -0.753 | -1.686 | -1.055 |
| CPIJ014219 | cytochrome P450 | RSM | 0.751 | 0.856 | 1.484 |
| CPIJ016849 | cytochrome P450 71B36 | RSM | 0.794 | 2.312 | 1.119 |
| CPIJ011081 | heat shock protein 70 B2 | RSM | 0.93 | 4.011 | 4.177 |
| CPIJ014220 | cytochrome P450 52D1 | RSM | 0.98 | 2.92 | 2.518 |
| CPIJ008261 | hypothetical protein | RSM | 1.012 | 3.312 | 3.462 |
| CPIJ002683 | glutathione S-transferase 1-1 | RSM | 1.053 | 2.055 | 1.804 |
| CPIJ018407 | cytochrome b561 | RSM | 1.337 | 1.287 | 1.039 |
| CPIJ007418 | cytochrome b561 | RSM | 1.687 | 1.405 | 1.348 |
| CPIJ010175 | cytochrome P450 9b1 | RSM | 2.232 | 2.628 | 1.6 |
| CPIJ010536 | cytochrome P450 9b2 | RSM | 2.402 | 2.318 | 1.971 |
| CPIJ007629 | splicing factor | RTT | -1.161 | -0.867 | -0.809 |
| CPIJ008664 | conserved hypothetical protein | RTT | -1.152 | 4.149 | 4.396 |
| CPIJ002050 | homeobox protein | RTT | -1.085 | -1.492 | -1.218 |
| CPIJ001440 | eukaryotic translation initiation factor 3 subunit | RTT | -1.008 | -1.183 | -0.798 |
| CPIJ004247 | transcriptional activator protein Pur-alpha | RTT | -0.815 | -0.963 | -0.79 |
| CPIJ012380 | histone H2A type 1-C | RTT | -0.774 | -1.141 | -1.219 |
| CPIJ003074 | conserved hypothetical protein | RTT | 0.829 | 0.845 | 0.774 |
| CPIJ009446 | DEAD box ATP-dependent RNA helicase | RTT | 0.931 | 1.605 | 1.131 |
| CPIJ003266 | CCAAT/enhancer-binding protein | RTT | 0.944 | 1.57 | 1.614 |
| CPIJ018256 | NULL | RTT | 1.077 | 1.607 | 1.712 |
| CPIJ005884 | eukaryotic translation initiation factor 2-alpha kinase 1 | RTT | 1.194 | 1.597 | 1.119 |
| CPIJ012801 | conserved hypothetical protein | RTT | 1.292 | 1.953 | 1.306 |
| CPIJ014791 | eukaryotic translation initiation factor 2C 2 | RTT | 1.361 | 1.68 | 1.474 |
| CPIJ003181 | RNAse H | RTT | 1.947 | 2.823 | 2.449 |
| CPIJ013993 | amino acid transporter | TRP | -2.457 | -1.857 | -1.291 |
| CPIJ007376 | calreticulin | TRP | -2.35 | -2.788 | -1.136 |
| CPIJ016887 | multidrug resistance protein 2 | TRP | -2.016 | -1.991 | -1.552 |
| CPIJ014926 | conserved hypothetical protein | TRP | -1.992 | -2.395 | -2.685 |
| CPIJ010101 | cation efflux protein/ zinc transporter | TRP | -1.982 | -2.557 | -2.307 |
| CPIJ005186 | organic anion transporter | TRP | -1.78 | -1.268 | -1.282 |
| CPIJ020291 | sodium-dependent multivitamin transporter | TRP | -1.555 | -1.318 | -1.084 |
| CPIJ001812 | sugar transporter | TRP | -1.482 | -1.234 | -0.851 |
| CPIJ013697 | tricarboxylate transport protein, mitochondrial | TRP | -1.472 | -1.835 | -1.225 |
| CPIJ004817 | sodium-dependent phosphate transporter | TRP | -1.449 | -1.866 | -1.68 |
| CPIJ004760 | low-Mr GTP-binding protein Rab31 | TRP | -1.407 | -1.705 | -1.283 |
| CPIJ010187 | sodium/solute symporter | TRP | -1.391 | -0.874 | -0.971 |
| CPIJ000242 | monocarboxylate transporter | TRP | -1.358 | -2.986 | -3.554 |
| CPIJ017878 | permease | TRP | -1.292 | -1.664 | -1.4 |
| CPIJ012068 | sucrose transport protein | TRP | -1.29 | -1.363 | -2.382 |
| CPIJ012675 | sugar transporter | TRP | -1.287 | -1.92 | -1.818 |
| CPIJ015637 | antioxidant enzyme | TRP | -1.203 | -2.257 | -1.909 |
| CPIJ008928 | cationic amino acid transporter | TRP | -1.193 | -1.191 | -0.753 |
| CPIJ019446 | copper transport protein | TRP | -1.178 | -2.286 | -1.933 |
| CPIJ014443 | ATP-binding cassette sub-family G member 4 | TRP | -1.174 | -1.52 | -0.985 |
| CPIJ007621 | choline dehydrogenase | TRP | -1.085 | -2.825 | -2.64 |
| CPIJ010409 | fructose-1,6-bisphosphatase 1 | TRP | -1.068 | -2.004 | -2.072 |
| CPIJ003167 | zinc/iron transporter | TRP | -0.988 | -2.056 | -2.472 |
| CPIJ004646 | UDP-galactose transporter | TRP | -0.956 | -1.366 | -0.833 |
| CPIJ019349 | malonyl CoA-acyl carrier protein transacylase | TRP | -0.901 | -1.603 | -0.864 |
| CPIJ010134 | sodium-dependent phosphate transporter | TRP | -0.88 | -2.446 | -1.649 |
| CPIJ015850 | d-amino acid oxidase | TRP | -0.876 | -1.437 | -0.825 |
| CPIJ000191 | sugar transporter | TRP | -0.8 | -1.147 | -1.228 |
| CPIJ017146 | 2-acylglycerol O-acyltransferase 2-A | TRP | -0.77 | -1.494 | -1.516 |
| CPIJ003880 | chloride channel protein 2 | TRP | -0.751 | -0.902 | -0.843 |
| CPIJ009224 | nodulin-26 | TRP | 0.834 | -1.408 | -2.027 |
| CPIJ010164 | katanin p60 ATPase-containing subunit | TRP | 0.866 | 1.003 | 0.81 |
| CPIJ007997 | sodium-dependent serotonin transporter | TRP | 0.891 | 1.22 | 0.77 |
| CPIJ009335 | ATPase n2b | TRP | 0.95 | 1.869 | 2.286 |
| CPIJ004221 | monocarboxylate transporter | TRP | 0.961 | 1.364 | 1.087 |
| CPIJ005816 | CRAL/TRIO domain-containing protein | TRP | 0.968 | 1.413 | 0.976 |
| CPIJ012563 | transmembrane protein 77 | TRP | 0.97 | 1.412 | 1.112 |
| CPIJ015036 | syntaxin | TRP | 0.972 | 1.243 | 1.178 |
| CPIJ008456 | cystinosin | TRP | 1.118 | 1.775 | 1.185 |
| CPIJ019820 | sugar transporter | TRP | 1.175 | 1.366 | 1.148 |
| CPIJ011949 | potassium-dependent sodium-calcium exchanger | TRP | 1.272 | 0.812 | 0.778 |
| CPIJ019526 | conserved hypothetical protein | TRP | 1.331 | 0.863 | 1.15 |
| CPIJ006785 | pickpocket | TRP | 1.416 | 1.95 | 0.942 |
| CPIJ002659 | transmembrane protease | TRP | 1.484 | 0.955 | 1.402 |
| CPIJ002443 | sodium-dependent phosphate transporter | TRP | 1.508 | 1.325 | 0.764 |
| CPIJ000674 | excitatory amino acid transporter 3 | TRP | 1.616 | 1.735 | 1.424 |
| CPIJ005368 | lysosomal trafficking regulator | TRP | 1.763 | 1.387 | 1.187 |
| CPIJ015359 | glucose transport protein | TRP | 1.94 | 2.285 | 1.204 |
| CPIJ012066 | sodium/Chloride dependent amino acid transporter | TRP | 2.139 | 3.213 | 1.178 |
| CPIJ012067 | conserved hypothetical protein | TRP | 2.486 | 3.862 | 1.053 |
| CPIJ000673 | glutamate transporter | TRP | 3.282 | 3.15 | 2.077 |
| CPIJ004492 | sodium/potassium/calcium exchanger 3 | TRP | 3.66 | 3.192 | 1.963 |
| CPIJ002361 | sodium/solute symporter | TRP | 4.597 | 4.905 | 1.271 |
| CPIJ007243 | conserved hypothetical protein | TRP, DIV | 1.862 | 1.327 | 1.172 |
| CPIJ014224 | conserved hypothetical protein | UNK | -3.684 | -5.054 | -4.904 |
| CPIJ002679 | glutathione S-transferase theta-2 | UNK | -3.598 | -2.541 | -1.082 |
| CPIJ016195 | hypothetical protein | UNK | -3.589 | -1.942 | -1.77 |
| CPIJ010758 | conserved hypothetical protein | UNK | -3.551 | -3.921 | -1.917 |
| CPIJ008809 | hypothetical protein | UNK | -3.234 | -1.51 | -1.634 |
| CPIJ010315 | Juvenile hormone-inducible protein | UNK | -3.058 | -4.095 | -3.314 |
| CPIJ008976 | 4-nitrophenylphosphatase | UNK | -2.972 | -1.466 | 0.828 |
| CPIJ011496 | hypothetical protein | UNK | -2.944 | -3.251 | -0.76 |
| CPIJ004733 | predicted protein | UNK | -2.856 | -2.802 | -2.177 |
| CPIJ004602 | serine 3-dehydrogenase | UNK | -2.749 | -3.333 | -2.873 |
| CPIJ009371 | conserved hypothetical protein | UNK | -2.692 | -3.614 | -3.116 |
| CPIJ010761 | conserved hypothetical protein | UNK | -2.482 | -2.609 | -0.906 |
| CPIJ001035 | conserved hypothetical protein | UNK | -2.414 | -3.633 | -2.771 |
| CPIJ010828 | conserved hypothetical protein | UNK | -2.351 | -2.013 | -1.233 |
| CPIJ004815 | uricase | UNK | -2.317 | -2.457 | -2.492 |
| CPIJ008859 | conserved hypothetical protein | UNK | -2.262 | -3.331 | -2.312 |
| CPIJ000150 | conserved hypothetical protein | UNK | -2.196 | -2.267 | -1.864 |
| CPIJ012069 | sucrose transport protein | UNK | -2.191 | -2.569 | -2.939 |
| CPIJ000061 | conserved hypothetical protein | UNK | -2.145 | -2.28 | -1.133 |
| CPIJ016920 | NADP-dependent L-serine/L-allo-threonine dehydrogenase ydfG | UNK | -2.139 | -2.67 | -2.809 |
| CPIJ017188 | conserved hypothetical protein | UNK | -2.094 | -2.103 | -0.916 |
| CPIJ001262 | alkaline phosphatase | UNK | -1.991 | -1.203 | -1.685 |
| CPIJ020028 | multicopper oxidase | UNK | -1.944 | -1.486 | -1.282 |
| CPIJ008582 | hypothetical protein | UNK | -1.907 | -1.668 | -1.149 |
| CPIJ016491 | 4-coumarate-CoA ligase 1 | UNK | -1.843 | -2.528 | -1.218 |
| CPIJ005087 | cell wall cysteine-rich protein | UNK | -1.758 | -1.488 | 1.239 |
| CPIJ015669 | AMP dependent ligase | UNK | -1.737 | -2.148 | -1.843 |
| CPIJ010320 | conserved hypothetical protein | UNK | -1.702 | -2.632 | -2.18 |
| CPIJ010311 | conserved hypothetical protein | UNK | -1.691 | -2.654 | -0.95 |
| CPIJ004598 | Acetoin | UNK | -1.66 | -1.799 | -1.038 |
| CPIJ008097 | conserved hypothetical protein | UNK | -1.657 | -2.128 | -0.802 |
| CPIJ001052 | aminopeptidase 2, mitochondrial | UNK | -1.604 | -3.234 | -2.874 |
| CPIJ018629 | glutathione-s-transferase theta, gst | UNK | -1.523 | -2.665 | -2.455 |
| CPIJ018625 | glutathione-s-transferase theta, gst | UNK | -1.517 | -1.705 | -1.244 |
| CPIJ017829 | predicted protein | UNK | -1.489 | -1.628 | 0.94 |
| CPIJ005200 | conserved hypothetical protein | UNK | -1.468 | -0.882 | 0.882 |
| CPIJ017722 | gamma glutamyl transpeptidase | UNK | -1.446 | -1.415 | -1.569 |
| CPIJ010324 | conserved hypothetical protein | UNK | -1.435 | -2.513 | -1.673 |
| CPIJ002675 | glutathione S-transferase 1 | UNK | -1.38 | -2.479 | -2.372 |
| CPIJ010903 | conserved hypothetical protein | UNK | -1.373 | -1.738 | -1.22 |
| CPIJ014161 | conserved hypothetical protein | UNK | -1.372 | -1.425 | -1.055 |
| CPIJ015920 | conserved hypothetical protein | UNK | -1.358 | -2.807 | -1.329 |
| CPIJ006221 | conserved hypothetical protein | UNK | -1.339 | -1.711 | -0.801 |
| CPIJ006693 | conserved hypothetical protein | UNK | -1.337 | -1.996 | -1.57 |
| CPIJ019996 | conserved hypothetical protein | UNK | -1.321 | -1.354 | -0.929 |
| CPIJ012786 | predicted protein | UNK | -1.295 | -1.508 | -1.452 |
| CPIJ015243 | hypothetical protein | UNK | -1.249 | 1.636 | 4.297 |
| CPIJ019683 | conserved hypothetical protein | UNK | -1.248 | -1.831 | -1.195 |
| CPIJ017746 | conserved hypothetical protein | UNK | -1.24 | -1.91 | -1.647 |
| CPIJ018834 | conserved hypothetical protein | UNK | -1.232 | -1.894 | -0.768 |
| CPIJ015296 | retinol-binding protein | UNK | -1.166 | -2.498 | -2.059 |
| CPIJ014162 | BTB/POZ domain containing protein | UNK | -1.162 | -1.087 | -1.178 |
| CPIJ018314 | conserved hypothetical protein | UNK | -1.157 | -1.192 | -1.735 |
| CPIJ007196 | conserved hypothetical protein | UNK | -1.155 | -0.888 | 1.214 |
| CPIJ004603 | oxidoreductase | UNK | -1.131 | -2.124 | -2.422 |
| CPIJ018116 | conserved hypothetical protein | UNK | -1.122 | -1.359 | -0.788 |
| CPIJ004283 | conserved hypothetical protein | UNK | -1.112 | -2.831 | -2.328 |
| CPIJ011592 | hypothetical protein | UNK | -1.107 | -1.731 | -1.621 |
| CPIJ012298 | conserved hypothetical protein | UNK | -1.098 | -1.139 | -1.416 |
| CPIJ012036 | aminopeptidase N | UNK | -1.046 | -1.726 | -2.12 |
| CPIJ015536 | outer mitochondrial translocase subunit | UNK | -1.006 | -1.432 | -0.811 |
| CPIJ012899 | secreted protein | UNK | -0.956 | -1.196 | -0.893 |
| CPIJ016492 | long-chain-fatty-acid-CoA ligase 1 | UNK | -0.953 | -1.621 | -0.789 |
| CPIJ016280 | conserved hypothetical protein | UNK | -0.937 | -1.028 | -0.869 |
| CPIJ011512 | conserved hypothetical protein | UNK | -0.931 | -0.784 | -1.264 |
| CPIJ005300 | sugar transporter | UNK | -0.895 | -1.883 | -2.012 |
| CPIJ019227 | pancreatic triacylglycerol lipase | UNK | -0.854 | 1.547 | 1.712 |
| CPIJ000559 | conserved hypothetical protein | UNK | -0.847 | -2.127 | -1.638 |
| CPIJ012907 | luciferin 4-monooxygenase | UNK | -0.839 | -3.579 | -3.295 |
| CPIJ004943 | hypothetical protein | UNK | -0.814 | -1.949 | -2.61 |
| CPIJ019565 | glucose-6-phosphate isomerase | UNK | -0.799 | -1.674 | -0.774 |
| CPIJ005369 | conserved hypothetical protein | UNK | -0.789 | -1.691 | -1.098 |
| CPIJ016792 | hypothetical protein | UNK | 0.76 | 1.658 | 2.424 |
| CPIJ005302 | conserved hypothetical protein | UNK | 0.775 | 1.655 | 1.178 |
| CPIJ002272 | conserved hypothetical protein | UNK | 0.791 | 1.704 | 1.232 |
| CPIJ010490 | conserved hypothetical protein | UNK | 0.793 | 1.22 | 1.012 |
| CPIJ007369 | conserved hypothetical protein | UNK | 0.794 | 1.624 | 1.518 |
| CPIJ016399 | conserved hypothetical protein | UNK | 0.795 | 2.481 | 1.829 |
| CPIJ015898 | conserved hypothetical protein | UNK | 0.796 | 1.555 | 0.873 |
| CPIJ007584 | conserved hypothetical protein | UNK | 0.803 | 1.82 | 1.699 |
| CPIJ012076 | conserved hypothetical protein | UNK | 0.807 | 1.655 | 1.953 |
| CPIJ014903 | zinc metalloprotease | UNK | 0.808 | 1.055 | 1.046 |
| CPIJ000942 | Misexpression suppressor of ras | UNK | 0.818 | 1.337 | 1.26 |
| CPIJ010077 | enhancer of polycomb | UNK | 0.818 | 1.647 | 1.583 |
| CPIJ002695 | conserved hypothetical protein | UNK | 0.823 | 0.799 | 1.58 |
| CPIJ002572 | conserved hypothetical protein | UNK | 0.833 | 1.905 | 1.791 |
| CPIJ003663 | conserved hypothetical protein | UNK | 0.833 | 0.907 | 1.194 |
| CPIJ000492 | conserved hypothetical protein | UNK | 0.837 | 1.059 | 0.867 |
| CPIJ017460 | luciferin 4-monooxygenase | UNK | 0.844 | 2.041 | 2.006 |
| CPIJ001667 | conserved hypothetical protein | UNK | 0.858 | 1.029 | 1.163 |
| CPIJ009976 | conserved hypothetical protein | UNK | 0.89 | 0.771 | 0.842 |
| CPIJ019637 | conserved hypothetical protein | UNK | 0.892 | 1.493 | 1.424 |
| CPIJ005325 | hypothetical protein | UNK | 0.896 | -0.819 | -1.687 |
| CPIJ010400 | conserved hypothetical protein | UNK | 0.896 | 1.383 | 1.088 |
| CPIJ018194 | conserved hypothetical protein | UNK | 0.897 | 1.152 | 0.802 |
| CPIJ017319 | hypothetical protein | UNK | 0.906 | 1.825 | 1.727 |
| CPIJ003963 | conserved hypothetical protein | UNK | 0.915 | 0.814 | 0.766 |
| CPIJ019628 | conserved hypothetical protein | UNK | 0.918 | 1.538 | 0.992 |
| CPIJ006112 | conserved hypothetical protein | UNK | 0.925 | 1.96 | 1.352 |
| CPIJ002957 | sugar transporter | UNK | 0.944 | 0.859 | 0.771 |
| CPIJ003366 | conserved hypothetical protein | UNK | 0.952 | 2.085 | 1.824 |
| CPIJ011964 | conserved hypothetical protein | UNK | 0.97 | 1.299 | 1.153 |
| CPIJ003180 | WS beta-transducin repeats protein | UNK | 0.996 | 1.418 | 2.027 |
| CPIJ020309 | hypothetical protein | UNK | 0.996 | 1.353 | 0.884 |
| CPIJ001345 | conserved hypothetical protein | UNK | 1.001 | 1.165 | 0.765 |
| CPIJ002091 | conserved hypothetical protein | UNK | 1.01 | 1.671 | 1.399 |
| CPIJ014044 | conserved hypothetical protein | UNK | 1.018 | 1.514 | 1.204 |
| CPIJ005544 | nuclear pore complex protein nup93 | UNK | 1.022 | 1.843 | 0.819 |
| CPIJ000490 | conserved hypothetical protein | UNK | 1.027 | 1.434 | 0.899 |
| CPIJ009630 | transcription factor IIIA | UNK | 1.036 | 1.074 | 1.02 |
| CPIJ008302 | conserved hypothetical protein | UNK | 1.041 | 1.083 | 0.752 |
| CPIJ001177 | transcription factor | UNK | 1.065 | 1.266 | 0.985 |
| CPIJ007283 | conserved hypothetical protein | UNK | 1.073 | 1.568 | 0.993 |
| CPIJ000343 | valacyclovir hydrolase | UNK | 1.075 | 1.26 | 1.345 |
| CPIJ007966 | conserved hypothetical protein | UNK | 1.086 | 1 | 0.98 |
| CPIJ001424 | conserved hypothetical protein | UNK | 1.087 | 2.46 | 1.656 |
| CPIJ020237 | conserved hypothetical protein | UNK | 1.088 | 1.834 | 1.951 |
| CPIJ017295 | NULL | UNK | 1.093 | 2.653 | 1.839 |
| CPIJ016094 | conserved hypothetical protein | UNK | 1.098 | 1.687 | 1.567 |
| CPIJ004346 | conserved hypothetical protein | UNK | 1.108 | 1.606 | 1.575 |
| CPIJ012374 | conserved hypothetical protein | UNK | 1.112 | 1.753 | 1.236 |
| CPIJ016398 | GTP-binding protein | UNK | 1.139 | 1.566 | 1.078 |
| CPIJ011414 | conserved hypothetical protein | UNK | 1.142 | 1.297 | 1.369 |
| CPIJ009740 | LIM protein pin-2 | UNK | 1.181 | 1.406 | 0.831 |
| CPIJ018923 | conserved hypothetical protein | UNK | 1.189 | 2.184 | 1.592 |
| CPIJ000676 | conserved hypothetical protein | UNK | 1.206 | 1.688 | 1.855 |
| CPIJ016555 | conserved hypothetical protein | UNK | 1.208 | 2.468 | 1.786 |
| CPIJ002777 | conserved hypothetical protein | UNK | 1.209 | 1.625 | 1.592 |
| CPIJ007206 | conserved hypothetical protein | UNK | 1.21 | 1.122 | 0.862 |
| CPIJ001996 | conserved hypothetical protein | UNK | 1.215 | 1.614 | 1.153 |
| CPIJ003536 | conserved hypothetical protein | UNK | 1.223 | 2.845 | 2.819 |
| CPIJ007760 | hypothetical protein | UNK | 1.223 | 1.6 | 1.629 |
| CPIJ009726 | conserved hypothetical protein | UNK | 1.223 | 2.493 | 1.999 |
| CPIJ011133 | predicted protein | UNK | 1.239 | 1.197 | 1.216 |
| CPIJ015367 | conserved hypothetical protein | UNK | 1.246 | 1.339 | 1.299 |
| CPIJ002274 | conserved hypothetical protein | UNK | 1.251 | 1.439 | 1.047 |
| CPIJ014088 | conserved hypothetical protein | UNK | 1.277 | 2.187 | 1.991 |
| CPIJ001282 | conserved hypothetical protein | UNK | 1.289 | 1.878 | 1.053 |
| CPIJ019789 | hypothetical protein | UNK | 1.317 | 1.348 | 0.965 |
| CPIJ012507 | conserved hypothetical protein | UNK | 1.326 | 1.36 | 0.756 |
| CPIJ011664 | conserved hypothetical protein | UNK | 1.327 | 1.37 | 0.946 |
| CPIJ016767 | conserved hypothetical protein | UNK | 1.357 | 1.42 | 0.901 |
| CPIJ012802 | conserved hypothetical protein | UNK | 1.364 | 1.556 | 1.26 |
| CPIJ009505 | conserved hypothetical protein | UNK | 1.389 | 1.611 | 1.102 |
| CPIJ014495 | transient receptor potential cation channel protein painless | UNK | 1.389 | 1.521 | 1.373 |
| CPIJ001940 | hypothetical protein | UNK | 1.391 | 3.014 | 0.898 |
| CPIJ012948 | conserved hypothetical protein | UNK | 1.392 | 1.607 | 0.97 |
| CPIJ013009 | c3f | UNK | 1.397 | 1.199 | 1.065 |
| CPIJ009229 | conserved hypothetical protein | UNK | 1.416 | 0.932 | 0.811 |
| CPIJ003708 | conserved hypothetical protein | UNK | 1.43 | 1.675 | 1.023 |
| CPIJ001281 | conserved hypothetical protein | UNK | 1.456 | 2.074 | 1.056 |
| CPIJ013194 | conserved hypothetical protein | UNK | 1.501 | 1.579 | 1.108 |
| CPIJ002468 | embryonic polarity dorsal | UNK | 1.502 | 1.395 | 0.958 |
| CPIJ011770 | hypothetical protein | UNK | 1.506 | 2.407 | 2.582 |
| CPIJ002673 | conserved hypothetical protein | UNK | 1.539 | 1.487 | 1.902 |
| CPIJ011663 | conserved hypothetical protein | UNK | 1.577 | 1.608 | 1.508 |
| CPIJ018717 | conserved hypothetical protein | UNK | 1.591 | 2.526 | 3.106 |
| CPIJ000466 | conserved hypothetical protein | UNK | 1.614 | 1.205 | 0.921 |
| CPIJ000211 | cysteine-rich secretory protein-2 | UNK | 1.629 | 2.391 | 1.986 |
| CPIJ011376 | hypothetical protein | UNK | 1.663 | 1.943 | 1.176 |
| CPIJ003938 | conserved hypothetical protein | UNK | 1.704 | 1.84 | 1.512 |
| CPIJ006519 | hypothetical protein | UNK | 1.771 | 3.92 | 3.106 |
| CPIJ007645 | conserved hypothetical protein | UNK | 1.809 | 3.942 | 3.691 |
| CPIJ006120 | microfibril-associated glycoprotein 4 | UNK | 1.83 | 2.072 | 2.7 |
| CPIJ014493 | transient receptor potential cation channel protein painless | UNK | 1.859 | 3.358 | 1.768 |
| CPIJ002628 | sensory appendage protein | UNK | 1.88 | 1.666 | 1.808 |
| CPIJ003907 | conserved hypothetical protein | UNK | 1.973 | 2.087 | 1.38 |
| CPIJ000495 | conserved hypothetical protein | UNK | 1.981 | 3.25 | 2.016 |
| CPIJ018014 | cell division protein ftsj | UNK | 1.981 | 0.906 | 0.998 |
| CPIJ018567 | hypothetical protein | UNK | 1.992 | 2.458 | 2.92 |
| CPIJ000500 | conserved hypothetical protein | UNK | 2.049 | 3.691 | 2.999 |
| CPIJ004065 | conserved hypothetical protein | UNK | 2.06 | 2.103 | 1.67 |
| CPIJ000496 | conserved hypothetical protein | UNK | 2.097 | 2.172 | 1.379 |
| CPIJ012254 | conserved hypothetical protein | UNK | 2.142 | 3.021 | 1.288 |
| CPIJ015534 | ER lumen protein retaining receptor | UNK | 2.204 | 2.18 | 1.829 |
| CPIJ000494 | conserved hypothetical protein | UNK | 2.218 | 2.464 | 1.149 |
| CPIJ005269 | conserved hypothetical protein | UNK | 2.218 | 1.585 | 0.944 |
| CPIJ007305 | conserved hypothetical protein | UNK | 2.288 | 3.49 | 3.398 |
| CPIJ002612 | sensory appendage protein | UNK | 2.333 | 3.461 | 3.637 |
| CPIJ000531 | hypothetical protein | UNK | 2.498 | 4.289 | 4.062 |
| CPIJ005157 | hypothetical protein | UNK | 2.519 | 2.206 | 1.882 |
| CPIJ011318 | conserved hypothetical protein | UNK | 2.532 | 4.618 | 1.399 |
| CPIJ003787 | conserved hypothetical protein | UNK | 2.754 | 2.715 | 1.683 |
| CPIJ014496 | conserved hypothetical protein | UNK | 2.768 | 3.142 | 3.573 |
| CPIJ003381 | SERAC1 | UNK | 2.979 | 4.609 | 3.727 |
