# Supplementary material for: Molecular analysis of Culex quinquefasciatus larvae responses to Lysinibacillus sphaericus Bin toxin
Source: PLoS One. 2017 Apr 13;12(4):e0175473. doi: 10.1371/journal.pone.0175473 (PMC5391067; doi:10.1371/journal.pone.0175473)
Supplement: S2 Table — Functional group abbreviations: CST, cytoskeletal and structural function; CSR, chemosensory reception; DIG, blood and sugar food digestive; DIV, diverse functions; IMM, immunity; MET, metabolism; PRT, proteolysis; RSM, redox, stress, and mitochondrion; RTT, replication, transcription, and translation; TRP, transport; UNK, unknown function. (DOCX) [file pone.0175473.s002.docx]

**Table S2** The functional groups of the total 3,781 transcripts were enriched or depleted in *C. quinquefasciatus* gut larvae in response to LC_90_ dose Bin treatment at 6, 12, or 18 h. Functional group abbreviations: CST, cytoskeletal and structural function; CSR, chemosensory reception; DIG, blood and sugar food digestive; DIV, diverse functions; IMM, immunity; MET, metabolism; PRT, proteolysis; RSM, redox, stress, and mitochondrion; RTT, replication, transcription, and translation; TRP, transport; UNK, unknown function.

| **GENE ID** | **NAME** | **FUNTIONAL GROUP** | **Log_2_ fold** | | |
| --- | --- | --- | --- | --- | --- |
|  |  |  | **6 h** | **12 h** | **18 h** |
| CPIJ019985 | sensory appendage protein | CSR | 1.905 | 3.122 | 3.597 |
| CPIJ019986 | serine/threonine kinase | CSR | 1.887 | 3.18 | 3.396 |
| CPIJ002611 | sensory appendage protein | CSR | 1.529 | 3.029 | 2.848 |
| CPIJ002629 | sensory appendage protein | CSR | 1.071 | 1.281 | 2.695 |
| CPIJ008374 | arrestin domain-containing protein 2 | CSR | 1.956 | 2.565 | 2.195 |
| CPIJ016967 | Odorant-binding protein 56e | CSR |  | 1.542 | 1.525 |
| CPIJ003583 | conserved hypothetical protein | CSR | 0.767 | 1.896 | 2.108 |
| CPIJ002609 | serine/threonine kinase | CSR | 2.009 | 1.698 | 1.891 |
| CPIJ008373 | conserved hypothetical protein | CSR |  | 0.846 | 1.005 |
| CPIJ008372 | arrestin domain containing 4 | CSR |  |  | 1.213 |
| CPIJ012718 | Odorant-binding protein 56e | CSR | -1.399 |  | 0.809 |
| CPIJ004634 | odorant-binding protein | CSR | 0.835 | 1.653 | 1.687 |
| CPIJ018957 | Odorant-binding protein 56e | CSR | -1.455 |  |  |
| CPIJ003584 | conserved hypothetical protein | CSR |  | 1.429 | 1.344 |
| CPIJ014525 | Odorant-binding protein 56a | CSR |  |  | 0.813 |
| CPIJ004130 | Odorant receptor 13a | CSR |  | -1.107 | -0.752 |
| CPIJ013952 | Odorant receptor 85d | CSR |  | -1.07 | -1.153 |
| CPIJ013946 | odorant receptor | CSR |  | -1.425 | -1.674 |
| CPIJ002605 | serine/threonine kinase | CSR | -1.69 | -2.384 | -2.153 |
| CPIJ001870 | predicted protein | CSR | -1.759 | -2.065 | -2.905 |
| CPIJ001869 | hypothetical protein | CSR | -1.75 | -2.348 | -2.966 |
| CPIJ017326 | general odorant-binding protein 99a | CSR | -1.464 | -3.863 | -5.333 |
| CPIJ001868 | predicted protein | CSR | -1.249 | -1.315 |  |
| CPIJ017524 | conserved hypothetical protein | CSR | -1.136 | -1.034 |  |
| CPIJ001867 | hypothetical protein | CSR |  | -0.998 |  |
| CPIJ005891 | conserved hypothetical protein | CSR |  | 0.884 |  |
| CPIJ006518 | hypothetical protein | CST | 1.735 | 3.871 | 3.041 |
| CPIJ004628 | Gelsolin | CST | 1.335 | 2.434 | 2.627 |
| CPIJ009326 | endocuticle structural glycoprotein SgAbd-4 | CST |  |  | 1.731 |
| CPIJ015734 | conserved hypothetical protein | CST |  | 2.377 | 2.761 |
| CPIJ008003 | annexin x | CST |  | 1.651 | 1.813 |
| CPIJ011729 | myosin light chain 1 | CST |  | 1.738 | 1.841 |
| CPIJ016709 | bifunctional aminoacyl-tRNA synthetase | CST |  |  | 1.588 |
| CPIJ015899 | myosin heavy chain | CST | 1.024 | 1.881 | 1.516 |
| CPIJ006350 | cortactin | CST |  | 1.028 | 1.494 |
| CPIJ006739 | dynein light chain | CST |  | 0.844 | 1.354 |
| CPIJ009151 | paramyosin | CST |  |  | 1.398 |
| CPIJ003487 | hypothetical protein | CST | -0.776 |  | 0.763 |
| CPIJ016344 | conserved hypothetical protein | CST |  | 1.319 | 1.433 |
| CPIJ009793 | glioma tumor suppressor candidate region gene 2 protein | CST | -0.788 |  | 0.828 |
| CPIJ016342 | conserved hypothetical protein | CST |  | 1.36 | 1.578 |
| CPIJ005875 | dynactin subunit 2 | CST |  |  | 1.1 |
| CPIJ010369 | conserved hypothetical protein | CST |  | 1.704 | 1.412 |
| CPIJ008831 | Pupal cuticle protein | CST | -1.347 |  | 0.938 |
| CPIJ003900 | bifunctional aminoacyl-tRNA synthetase | CST | 0.813 | 1.334 | 1.194 |
| CPIJ002897 | conserved hypothetical protein | CST | 1.302 | 1.447 | 0.966 |
| CPIJ008558 | conserved hypothetical protein | CST |  | 0.944 | 0.94 |
| CPIJ014101 | conserved hypothetical protein | CST | 1.017 | 2.059 | 0.784 |
| CPIJ003952 | conserved hypothetical protein | CST |  | 1.422 | 1.113 |
| CPIJ008183 | kinesin-associated protein | CST |  |  | 0.955 |
| CPIJ008974 | cuticle protein | CST |  | 0.98 | 0.852 |
| CPIJ008922 | septin | CST |  | 0.925 | 1.041 |
| CPIJ014790 | spectrin alpha chain | CST |  |  | 0.985 |
| CPIJ003871 | paxillin | CST | 0.817 | 1.431 | 0.981 |
| CPIJ003742 | beta-parvin | CST |  | 0.862 | 0.858 |
| CPIJ017208 | BMP-binding endothelial regulator protein | CST, DIV |  | 1.072 | 0.795 |
| CPIJ000682 | conserved hypothetical protein | CST |  |  | 1.057 |
| CPIJ003488 | hypothetical protein | CST | -0.849 |  |  |
| CPIJ009300 | myosin-Va | CST |  |  | 0.803 |
| CPIJ012935 | thymosin 1 | CST |  |  | 0.763 |
| CPIJ000681 | obstractor B | CST |  |  | 0.86 |
| CPIJ015771 | kinesin light chain 1 and | CST |  |  | 0.878 |
| CPIJ010989 | paxillin | CST |  | 1.225 | 0.954 |
| CPIJ008540 | muscle-specific protein 20 | CST |  |  | 0.928 |
| CPIJ015035 | myosin-IB | CST |  |  | -1.039 |
| CPIJ006528 | anion exchange protein 2, slc4a2 | CST |  | -0.843 | -0.997 |
| CPIJ013626 | conserved hypothetical protein | CST |  |  | -0.966 |
| CPIJ019804 | pupal cuticle protein | CST |  |  | -0.966 |
| CPIJ003836 | integrin beta-nu | CST |  |  | -1.02 |
| CPIJ003960 | conserved hypothetical protein | CST | 0.937 |  | -0.784 |
| CPIJ016750 | conserved hypothetical protein | CST |  | -0.838 | -1.079 |
| CPIJ007903 | peritrophin-1 | CST |  |  | -1.465 |
| CPIJ007898 | conserved hypothetical protein | CST |  |  | -1.976 |
| CPIJ007909 | conserved hypothetical protein | CST |  |  | -1.992 |
| CPIJ007902 | conserved hypothetical protein | CST |  |  | -1.493 |
| CPIJ012138 | conserved hypothetical protein | CST |  |  | -1.259 |
| CPIJ018642 | pupal cuticle protein | CST |  |  | -1.222 |
| CPIJ007906 | conserved hypothetical protein | CST |  |  | -1.441 |
| CPIJ018641 | pupal cuticle protein | CST |  |  | -1.179 |
| CPIJ014269 | chitin synthase 1 | CST |  |  | -1.986 |
| CPIJ007904 | conserved hypothetical protein | CST |  |  | -1.934 |
| CPIJ004287 | conserved hypothetical protein | CST |  | -1.129 | -1.511 |
| CPIJ007900 | conserved hypothetical protein | CST |  |  | -2.192 |
| CPIJ007956 | conserved hypothetical protein | CST |  | -1.424 | -1.855 |
| CPIJ007300 | conserved hypothetical protein | CST |  | -1.818 | -2.07 |
| CPIJ011304 | conserved hypothetical protein | CST | 1.108 |  | -2.219 |
| CPIJ012928 | tubulin alpha-1 chain | CST | -0.957 | -1.529 | -0.797 |
| CPIJ016326 | conserved hypothetical protein | CST | -1.091 | -2.747 | -0.92 |
| CPIJ012934 | conserved hypothetical protein | CST | -3.985 | -3.889 | -1.67 |
| CPIJ001078 | Pupal cuticle protein | CST | -2.091 | -1.856 | -0.965 |
| CPIJ008005 | annexin x | CST | 0.975 | 1.31 | 0.753 |
| CPIJ008557 | conserved hypothetical protein | CST | 1.289 | 0.925 | 0.757 |
| CPIJ013337 | tubulin-specific chaperone e | CST | 0.859 | 1.151 | 0.826 |
| CPIJ012220 | microtubule binding protein D-CLIP-190 | CST |  | 0.961 | 0.953 |
| CPIJ013834 | ARP2/3 complex 21 kDa subunit | CST |  | -0.783 | -0.863 |
| CPIJ019577 | alpha-actinin | CST |  | -1.284 | -0.954 |
| CPIJ003954 | conserved hypothetical protein | CST |  |  | 0.798 |
| CPIJ001684 | filamin | CST |  |  | 0.754 |
| CPIJ003953 | conserved hypothetical protein | CST |  |  | -1.139 |
| CPIJ007441 | conserved hypothetical protein | CST |  |  | 0.779 |
| CPIJ007899 | conserved hypothetical protein | CST |  |  | -1.325 |
| CPIJ008405 | plekhh1 | CST |  |  | 0.812 |
| CPIJ009226 | groucho protein | CST |  |  | -0.762 |
| CPIJ009333 | cuticle protein | CST |  |  | 1.152 |
| CPIJ011272 | conserved hypothetical protein | CST |  |  | 0.755 |
| CPIJ015328 | nesprin | CST |  |  | 0.753 |
| CPIJ016644 | integrin-linked protein kinase | CST |  |  | 0.83 |
| CPIJ019541 | conserved hypothetical protein | CST |  |  | 0.824 |
| CPIJ003966 | conserved hypothetical protein | CST | 1.569 | 2.293 |  |
| CPIJ016325 | conserved hypothetical protein | CST | -1.948 | -2.609 |  |
| CPIJ004731 | conserved hypothetical protein | CST | 1.307 | 1.031 |  |
| CPIJ003967 | conserved hypothetical protein | CST | 2.398 | 1.443 |  |
| CPIJ004730 | conserved hypothetical protein | CST | 1.431 | 1.836 |  |
| CPIJ006777 | conserved hypothetical protein | CST | -0.943 | -1.374 |  |
| CPIJ011935 | conserved hypothetical protein | CST | -1.684 | -2.171 |  |
| CPIJ000679 | conserved hypothetical protein | CST | -1.048 | -1.135 |  |
| CPIJ011550 | tubulin alpha-2 chain | CST | -0.788 | -1.119 |  |
| CPIJ007603 | conserved hypothetical protein | CST | -1.014 | -1.249 |  |
| CPIJ001741 | myosin-VIIa | CST | 0.866 | 0.931 |  |
| CPIJ001834 | cuticle protein | CST | -0.936 | 0.762 |  |
| CPIJ002097 | rho/rac/cdc GTPase-activating protein | CST | 1.033 | 0.821 |  |
| CPIJ003446 | cuticle protein | CST | -1.133 | -0.921 |  |
| CPIJ003447 | cuticle protein | CST | -1.128 | -0.96 |  |
| CPIJ003448 | cuticle protein | CST | -1.013 | -0.771 |  |
| CPIJ003449 | cuticle protein | CST | -1.288 | -0.876 |  |
| CPIJ003450 | cuticle protein | CST | -1.171 | -0.876 |  |
| CPIJ003453 | cuticle protein | CST | -1.139 | -0.762 |  |
| CPIJ003479 | cuticle protein | CST | -0.963 | -0.772 |  |
| CPIJ008214 | testin | CST | 0.937 | 1.016 |  |
| CPIJ010204 | groucho protein | CST | -0.925 | -0.788 |  |
| CPIJ012653 | septin | CST | 1.469 | 1.005 |  |
| CPIJ012877 | innexin inx3 | CST | 0.959 | 0.762 |  |
| CPIJ017383 | tubulin alpha-1 chain | CST | -0.798 | -1.017 |  |
| CPIJ017970 | conserved hypothetical protein | CST | -0.85 | -0.881 |  |
| CPIJ012466 | pupal cuticle protein 78E | CST |  | 1.007 |  |
| CPIJ016316 | pupal cuticle protein 78E | CST |  | -0.85 |  |
| CPIJ000085 | conserved hypothetical protein | CST |  | -1.232 |  |
| CPIJ001786 | collagen alpha chain | CST |  | -0.901 |  |
| CPIJ003961 | conserved hypothetical protein | CST |  | 1.493 |  |
| CPIJ016323 | cuticle protein CP14.6 | CST |  | -1.94 |  |
| CPIJ000680 | conserved hypothetical protein | CST |  | -0.774 |  |
| CPIJ003635 | tubulin beta-1 chain | CST |  | -1.732 |  |
| CPIJ006133 | conserved hypothetical protein | CST |  | -1.075 |  |
| CPIJ007018 | conserved hypothetical protein | CST |  | -1.465 |  |
| CPIJ007897 | conserved hypothetical protein | CST |  | 0.836 |  |
| CPIJ013883 | angiomotin | CST |  | 0.761 |  |
| CPIJ013963 | dynein light chain | CST |  | -0.88 |  |
| CPIJ020138 | conserved hypothetical protein | CST |  | 1.484 |  |
| CPIJ003581 | conserved hypothetical protein | CST | 0.828 |  |  |
| CPIJ001833 | cuticle protein | CST | -0.819 |  |  |
| CPIJ001835 | cuticle protein | CST | -0.926 |  |  |
| CPIJ003226 | actin | CST | 0.792 |  |  |
| CPIJ003476 | cuticle protein | CST | -1.11 |  |  |
| CPIJ003477 | cuticle protein | CST | -1.15 |  |  |
| CPIJ004460 | tubulin-specific chaperone | CST | 0.981 |  |  |
| CPIJ012574 | actin | CST | -0.84 |  |  |
| CPIJ013361 | tropomyosin-1 | CST | -0.885 |  |  |
| CPIJ002148 | phenylalanine-4-hydroxylase | MET | -1.346 | 2.673 | 3.107 |
| CPIJ003279 | tryptophanyl-tRNA synthetase | MET |  | 2.594 | 3.423 |
| CPIJ015715 | AMP dependent ligase | MET | 2.438 | 3.933 | 3.155 |
| CPIJ005348 | lipase 3 | MET | 2.573 | 3.598 | 3.116 |
| CPIJ008181 | inosine-uridine preferring nucleoside hydrolase | MET | 1.256 | 2.353 | 3.231 |
| CPIJ018191 | GDP-mannose 4,6 dehydratase | MET |  |  | 2.496 |
| CPIJ012679 | adam | MET | 0.931 | 2.576 | 2.833 |
| CPIJ016639 | acetyl-CoA synthetase | MET | 1.662 | 1.931 | 2.59 |
| CPIJ006769 | glucosamine-fructose-6-phosphate aminotransferase 2 | MET | 0.963 | 1.755 | 2.267 |
| CPIJ008716 | tubulointerstitial nephritis antigen | MET, PRT | 1.782 | 1.983 | 2.268 |
| CPIJ002959 | sterol O-acyltransferase 2 | MET | 2.379 | 2.153 | 1.688 |
| CPIJ012919 | GDP-mannose 4,6 dehydratase | MET |  |  | 1.495 |
| CPIJ007034 | lipase | MET | -3.871 | -3.582 |  |
| CPIJ007035 | lipase | MET | -4.413 | -4.576 | -1.174 |
| CPIJ003367 | lumbrokinase-3 | MET |  | 1.949 | 2.059 |
| CPIJ012841 | valacyclovir hydrolase | MET | 0.838 | 1.126 | 1.599 |
| CPIJ004780 | serine palmitoyltransferase | MET |  | 1.453 | 1.797 |
| CPIJ010432 | acyl-coenzyme A thioesterase 9 | MET, DIV | 0.86 | 0.873 | 1.728 |
| CPIJ006770 | glucosamine-fructose-6-phosphate aminotransferase 2 | MET | 0.827 | 1.632 | 1.968 |
| CPIJ011996 | 10-formyltetrahydrofolate dehydrogenase | MET | 0.77 | 1.424 | 2.168 |
| CPIJ016131 | l-lactate dehydrogenase | MET | -0.937 |  | 1.52 |
| CPIJ017452 | lysyl-tRNA synthetase | MET |  | 1.163 | 1.786 |
| CPIJ007036 | phospholipase A1 1 | MET | -2.148 | -1.826 |  |
| CPIJ012753 | cysteine synthase | MET |  | 1.122 | 1.371 |
| CPIJ014454 | l-lactate dehydrogenase | MET | -0.97 |  | 1.343 |
| CPIJ018208 | cholinephosphate cytidylyl transferase B2 | MET | 2.047 | 2.549 | 1.337 |
| CPIJ004564 | brain chitinase and chia | MET |  |  | 1.096 |
| CPIJ004029 | venom allergen 5 | MET, DIV | -1.806 | -1.607 |  |
| CPIJ001337 | uridine 5'-monophosphate synthase | MET | 0.808 | 1.185 | 1.03 |
| CPIJ004967 | uracil phosphoribosyltransferase | MET | 1.602 | 1.978 | 1.542 |
| CPIJ017812 | hexokinase | MET | 1.091 | 0.843 | 0.956 |
| CPIJ000008 | chitotriosidase-1 | MET | -2.234 | -2.19 |  |
| CPIJ005214 | ceramide glucosyltransferase | MET | 0.901 | 1.1 | 1.534 |
| CPIJ002689 | survivin | MET, IMM |  | 1.227 | 1.262 |
| CPIJ008049 | hexokinase | MET | 1.151 | 1.092 | 0.864 |
| CPIJ008607 | sterol regulatory element-binding protein 1 | MET |  | 1.474 | 1.491 |
| CPIJ019159 | elongase | MET |  | 0.912 | 1.145 |
| CPIJ015088 | 4-coumarate-CoA ligase 1 | MET | 1.462 |  |  |
| CPIJ017745 | sterol desaturase | MET | 1.443 | 0.948 | 1.194 |
| CPIJ008348 | GATA transcription factor GATAd | MET, RTT |  | 0.907 | 1.427 |
| CPIJ001663 | survivin | MET, IMM |  | 1.446 | 1.142 |
| CPIJ003090 | cullin | MET | 1.551 | 1.99 | 1.214 |
| CPIJ018167 | sterol regulatory element-binding protein 1 | MET | 0.851 | 1.394 | 1.559 |
| CPIJ007406 | thymus-specific serine protease | MET |  |  | 1.061 |
| CPIJ004709 | bifunctional protein folD | MET |  | 1.555 | 1.295 |
| CPIJ010718 | conserved hypothetical protein | MET |  | 1.404 | 0.987 |
| CPIJ002278 | S-methyl-5-thioadenosine phosphorylase | MET | -1.082 | -1.015 |  |
| CPIJ011260 | phosphoglucomutase | MET | 0.933 | 1.231 | 1.026 |
| CPIJ002032 | conserved hypothetical protein | MET |  |  | 1.099 |
| CPIJ012747 | uridine cytidine kinase i | MET |  |  | 1.078 |
| CPIJ001676 | CDP-diacylglycerol-glycerol-3-phosphate 3-phosphatidyltransferase | MET |  |  | 0.971 |
| CPIJ001555 | choline/ethanolamine kinase | MET |  | 1.287 | 1.229 |
| CPIJ017396 | AMP dependent ligase | MET |  |  | 1.153 |
| CPIJ011301 | achelase-2 | MET | 2.034 | 0.977 |  |
| CPIJ002958 | sterol o-acyltransferase | MET | 0.751 | 1.065 | 0.898 |
| CPIJ006325 | choline/ethanolamine kinase | MET | 1.404 |  | 1.267 |
| CPIJ019694 | ceramide glucosyltransferase | MET | 1.007 | 1.304 | 1.043 |
| CPIJ004417 | 4-hydroxyphenylpyruvate dioxygenase | MET |  |  | 1.605 |
| CPIJ014202 | dopamine beta hydroxylase | MET |  | 1.099 | 0.862 |
| CPIJ013862 | phosphoglucomutase | MET | 0.966 | 1.354 | 0.891 |
| CPIJ003276 | deoxynucleoside kinase | MET | -0.951 |  |  |
| CPIJ002952 | conserved hypothetical protein | MET | -1.231 |  |  |
| CPIJ005462 | pancreatic triacylglycerol lipase | MET |  | -1.159 |  |
| CPIJ015259 | nucleosome assembly protein | MET |  | 0.88 |  |
| CPIJ002923 | caspase-1 | MET, IMM |  | 0.977 | 1.102 |
| CPIJ016885 | pre-mRNA-splicing factor prp1 | MET |  | 0.907 | 0.76 |
| CPIJ020069 | methionine aminopeptidase 2 | MET |  |  | 0.751 |
| CPIJ005543 | phosphatidylinositol 3-kinase 1 | MET | 1.747 | 2.068 | 1.094 |
| CPIJ012003 | conserved hypothetical protein | MET |  |  | 0.871 |
| CPIJ017511 | chitotriosidase-1 | MET |  |  | 1.513 |
| CPIJ008532 | glycoside hydrolase | MET |  |  | 0.889 |
| CPIJ006326 | dihydrolipoamide branched chain transacylase E2 | MET |  | -1.359 |  |
| CPIJ004319 | molybdenum cofactor synthesis protein cinnamon | MET | -1.107 | -1.638 | -0.873 |
| CPIJ019861 | citrate synthase, mitochondrial | MET | -1.034 | -1.643 |  |
| CPIJ003841 | methylcrotonoyl-CoA carboxylase alpha chain, mitochondrial | MET | -1.371 | -1.926 | -0.867 |
| CPIJ009697 | glutathione synthetase | MET | -0.887 | -1.221 | -0.951 |
| CPIJ006248 | D-arabinose 1-dehydrogenase | MET |  | -1.121 |  |
| CPIJ019501 | hydroxyacylglutathione hydrolase | MET | -0.81 | -1.276 |  |
| CPIJ008959 | ribulokinase 2 | MET |  | -1.085 | -0.896 |
| CPIJ006453 | trans-2-enoyl-CoA reductase, mitochondrial | MET, DIV |  | -1.102 | -0.829 |
| CPIJ015656 | lysosomal alpha-mannosidase | MET |  |  | -0.838 |
| CPIJ006124 | prolylcarboxypeptidase | MET, PRT |  |  | -0.992 |
| CPIJ006619 | cystathionine gamma-lyase | MET |  | -1.231 | -1.247 |
| CPIJ015655 | lysosomal alpha-mannosidase | MET |  |  | -0.915 |
| CPIJ005246 | abhydrolase domain-containing protein 7 | MET |  | -1.189 |  |
| CPIJ000863 | 6-pyruvoyl tetrahydrobiopterin synthase | MET |  |  | -1.024 |
| CPIJ012752 | cysteine synthase | MET | -2.175 | -2.509 | -1.343 |
| CPIJ006299 | glyoxalase domain-containing protein 4 | MET |  | -1.262 | -1.03 |
| CPIJ010605 | conserved hypothetical protein | MET | -1.813 | -2.004 |  |
| CPIJ004847 | alpha methylacyl-coa racemase | MET |  | -1.091 | -1.075 |
| CPIJ008878 | prolylcarboxypeptidase | MET, PRT | -3.9 | -3.218 | -1.034 |
| CPIJ000425 | short-chain-fatty-acid-CoA ligase | MET |  | -0.944 | -0.986 |
| CPIJ010621 | mannosyl-oligosaccharide glucosidase | MET |  | -0.937 | -1.055 |
| CPIJ006455 | enoyl-CoA hydratase, mitochondrial | MET |  | -0.818 | -0.984 |
| CPIJ007527 | carbonic anhydrase | MET | -1.017 | -1.401 | -0.906 |
| CPIJ014783 | isovaleryl-CoA dehydrogenase, mitochondrial | MET |  | -1.653 | -1.131 |
| CPIJ002460 | malate dehydrogenase | MET |  |  | -1.193 |
| CPIJ015671 | glyoxylate reductase/hydroxypyruvate reductase | MET |  |  | -0.916 |
| CPIJ002285 | conserved hypothetical protein | MET |  | -0.856 | -0.875 |
| CPIJ010973 | glutamine synthetase 1, mitochondrial | MET | -2.395 | -2.297 | -1.087 |
| CPIJ005324 | UDP-glucuronosyltransferase 2B18 | MET |  |  | -0.832 |
| CPIJ002383 | bifunctional purine biosynthesis protein PURH | MET | -1.124 | -1.645 | -0.818 |
| CPIJ011631 | short-chain specific acyl-CoA dehydrogenase, mitochondrial | MET | -0.801 | -1.498 | -1.127 |
| CPIJ013748 | acyl-CoA desaturase 1 | MET | -1.146 |  |  |
| CPIJ016353 | elongase | MET |  | -1.153 | -1.24 |
| CPIJ009498 | adrenodoxin | MET |  | -1.133 | -0.996 |
| CPIJ009280 | phosphoglycerate kinase | MET | -2.112 | -1.809 | -0.95 |
| CPIJ017646 | prostatic acid phosphatase | MET |  |  | -0.863 |
| CPIJ007198 | aldehyde dehydrogenase, mitochondrial | MET |  |  | -1.015 |
| CPIJ011103 | glutamyl aminopeptidase | MET, PRT |  |  | -1.18 |
| CPIJ006430 | oviductin | MET, IMM |  | -0.932 | -1.136 |
| CPIJ009966 | sphingosine kinase a, b | MET |  | -0.788 | -0.864 |
| CPIJ007302 | long-chain fatty acid transport protein 4 | MET | -1.254 | -1.522 | -1.456 |
| CPIJ005698 | N-acetyl galactosaminyl transferase | MET |  |  | -1.242 |
| CPIJ002514 | pyruvate carboxylase, mitochondrial | MET |  | -1.155 | -1.229 |
| CPIJ006068 | utp-glucose-1-phosphate uridylyltransferase 2 | MET | -1.319 | -1.436 | -1.151 |
| CPIJ020263 | fatty acid oxidation complex subunit alpha | MET |  | -2.138 | -1.299 |
| CPIJ010979 | phosphatidylcholine-sterol acyltransferase | MET | 2.397 |  | -0.846 |
| CPIJ000351 | UDP-glucuronosyltransferase 2B28 | MET | -1.351 | -1.859 | -0.792 |
| CPIJ001655 | aldose 1-epimerase | MET | -0.986 | -1.284 | -1.332 |
| CPIJ000225 | UDP-glucuronosyltransferase R-21 | MET | -1.506 | -1.96 | -1.183 |
| CPIJ006761 | glucose-6-phosphate 1-dehydrogenase | MET |  |  | -0.977 |
| CPIJ008783 | alcohol dehydrogenase class 3 | MET | -0.959 | -1.894 | -1.225 |
| CPIJ003692 | glucosyl/glucuronosyl transferase | MET |  | -1.751 |  |
| CPIJ013380 | sorbitol dehydrogenase | MET, DIV | 0.772 |  | -1.008 |
| CPIJ011317 | lysosomal acid phosphatase | MET |  | -1.481 | -1.427 |
| CPIJ009756 | DNA topoisomerase 3-beta | MET |  |  | -0.918 |
| CPIJ001670 | glycerol-3-phosphate acyltransferase | MET |  | -1.112 | -0.16 |
| CPIJ006141 | pantothenate kinase | MET |  | -1.172 | -1.391 |
| CPIJ006245 | coproporphyrinogen III oxidase | MET | -1.528 | -2.58 | -1.459 |
| CPIJ011495 | chitotriosidase-1 | MET |  |  | -1.818 |
| CPIJ010743 | 4-nitrophenylphosphatase | MET |  | -0.804 | -1.18 |
| CPIJ001998 | 1-acyl-sn-glycerol-3-phosphate acyltransferase | MET | -1.383 | -2.087 | -1.352 |
| CPIJ000032 | glucosyl/glucuronosyl transferase | MET |  |  | -0.901 |
| CPIJ003183 | N | MET |  | -1.585 | -0.283 |
| CPIJ007660 | pyrroline-5-carboxylate dehydrogenase | MET | -0.91 | -1.363 | -1.185 |
| CPIJ008635 | lysosomal acid phosphatase | MET |  | -1.247 | -1.307 |
| CPIJ006323 | xaa-Pro aminopeptidase 1 | MET | -1.195 | -1.999 | -1.69 |
| CPIJ000207 | glycerol kinase | MET |  |  | -1.44 |
| CPIJ011600 | long-chain-fatty-acid coa ligase | MET | -2.306 | -2.341 | -1.413 |
| CPIJ005914 | sterol o-acyltransferase | MET |  | -1.139 | -1.33 |
| CPIJ000226 | glucosyl/glucuronosyl transferase | MET | -2.061 | -1.374 | -1.406 |
| CPIJ000005 | malate synthase | MET |  | -0.861 | -1.733 |
| CPIJ000128 | galactokinase | MET | -1.224 | -1.921 | -1.391 |
| CPIJ010817 | ribulose-phosphate 3-epimerase | MET |  | -1.175 | -1.481 |
| CPIJ014058 | NADP-dependent leukotriene B4 12-hydroxydehydrogenase | MET, RSM |  | -1.423 | -1.48 |
| CPIJ009148 | acyl-CoA dehydrogenase | MET |  |  | -1.48 |
| CPIJ005435 | peroxisomal 3,2-trans-enoyl-CoA isomerase | MET |  | -0.96 | -1.26 |
| CPIJ008366 | mitochondrial malate dehydrogenase 2 | MET |  | -1.202 | -1.586 |
| CPIJ005697 | N-acetylgalactosaminyltransferase | MET |  | -1.165 | -1.427 |
| CPIJ001352 | N-myc downstream regulated | MET |  |  | -1.2 |
| CPIJ008217 | acyl-coa dehydrogenase | MET |  | -1.278 | -1.498 |
| CPIJ019138 | fumarylacetoacetate hydrolase domain-containing protein 1 | MET |  | -1.588 | -1.543 |
| CPIJ008729 | 4-aminobutyrate aminotransferase, mitochondrial | MET | -1.28 | -2.199 | -1.816 |
| CPIJ015417 | xylulose kinase | MET | -0.778 | -1.888 | -1.731 |
| CPIJ015186 | galactokinase | MET |  |  | -1.517 |
| CPIJ002354 | triosephosphate isomerase | MET | -2.119 | -2.513 | -1.348 |
| CPIJ014580 | dimeric dihydrodiol dehydrogenase | MET |  | -1.374 | -1.778 |
| CPIJ004373 | UDP-glucuronosyltransferase | MET | -2.874 | -3.369 | -1.887 |
| CPIJ005491 | dipeptidyl peptidase 4 | MET |  |  | -1.837 |
| CPIJ009981 | conserved hypothetical protein | MET |  | -0.867 | -1.526 |
| CPIJ005681 | phosphoglucomutase | MET |  | -1.738 | -1.462 |
| CPIJ003061 | acyl-CoA oxidase | MET | -1.02 | -1.829 | -1.782 |
| CPIJ011465 | C-4 methylsterol oxidase | MET | -1.051 | -1.748 | -1.489 |
| CPIJ001129 | valacyclovir hydrolase | MET |  |  | -1.567 |
| CPIJ013377 | sorbitol dehydrogenase | MET, DIV | 1.452 | -0.755 | -1.754 |
| CPIJ004224 | pancreatic triacylglycerol lipase | MET |  | -1.185 | -1.883 |
| CPIJ001564 | selenide | MET | -1.589 | -1.914 | -1.49 |
| CPIJ004141 | 1-acyl-sn-glycerol-3-phosphate acyltransferase beta | MET | -1.141 | -1.615 | -1.369 |
| CPIJ015739 | long-chain-fatty-acid coa ligase | MET |  | -0.916 | -1.578 |
| CPIJ004138 | 1-acyl-sn-glycerol-3-phosphate acyltransferase | MET | -1.957 | -2.624 | -1.703 |
| CPIJ016732 | malate dehydrogenase | MET |  |  | -1.512 |
| CPIJ018230 | sphingosine-1-phosphate lyase | MET |  | -1.518 | -1.801 |
| CPIJ000039 | glucosyl/glucuronosyl transferase | MET |  | -1.031 | -1.52 |
| CPIJ010456 | cysteine dioxygenase | MET | -1.377 | -1.924 | -1.144 |
| CPIJ006930 | gamma-glutamyl hydrolase | MET |  |  | -1.65 |
| CPIJ009438 | aldehyde dehydrogenase | MET | -1.061 | -2.55 | -1.591 |
| CPIJ004228 | lipase | MET |  |  | -1.591 |
| CPIJ011877 | NULL | MET | -0.765 | -1.885 | -1.671 |
| CPIJ018047 | 3-hydroxyacyl-coa dehyrogenase | MET, DIV |  | -1.337 | -1.766 |
| CPIJ006166 | deltamethrin resistance-associated NYD-GBE | MET |  | -1.917 | -1.971 |
| CPIJ019378 | long-chain-fatty-acid coa ligase | MET |  | -1.003 | -1.702 |
| CPIJ018004 | selenide | MET |  | -1.261 | -1.803 |
| CPIJ012157 | polyserase-2 | MET |  | -1.295 | -1.218 |
| CPIJ010683 | transaldolase | MET |  | -2.004 | -1.895 |
| CPIJ000349 | UDP-glucuronosyltransferase 1-1 | MET |  | -1.356 | -2.195 |
| CPIJ006775 | arylsulfatase b | MET |  | -1.037 | -1.751 |
| CPIJ008287 | phosphoserine aminotransferase | MET |  | -1.732 | -2.025 |
| CPIJ000037 | UDP-glucuronosyltransferase 2B20 | MET |  |  | -1.411 |
| CPIJ003656 | luciferin 4-monooxygenase | MET |  | -0.926 | -1.822 |
| CPIJ018325 | lipase | MET |  | -1.057 | -1.61 |
| CPIJ002685 | enoyl-CoA hydratase ECHA12 | MET | -0.968 | -2.343 | -1.781 |
| CPIJ008869 | homocysteine S-methyltransferase | MET |  | -1.051 | -1.292 |
| CPIJ020058 | pyridoxal kinase | MET | -2.157 | -2.604 | -1.481 |
| CPIJ019470 | 3-demethylubiquinone-9 3-methyltransferase | MET | -2.159 | -2.698 | -2.008 |
| CPIJ015209 | 3-hydroxyisobutyrate dehydrogenase | MET | -1.076 | -2.485 | -1.843 |
| CPIJ019115 | lambda-crystallin | MET, DIV |  | -1.653 | -2.013 |
| CPIJ014581 | aldehyde dehydrogenase | MET |  | -0.796 | -1.615 |
| CPIJ009095 | luciferin 4-monooxygenase | MET |  | -2.125 | -2.211 |
| CPIJ020056 | cysteine dioxygenase | MET | -0.849 | -1.592 | -1.846 |
| CPIJ012160 | sphingomyelin phosphodiesterase | MET |  | -1.178 | -2.13 |
| CPIJ011533 | carbonic anhydrase | MET |  | -1.337 | -1.7 |
| CPIJ015124 | sterol carrier protein-2 | MET |  |  | -2.071 |
| CPIJ017705 | 2-hydroxyphytanoyl-coa lyase | MET |  | -1.843 | -2.02 |
| CPIJ009093 | ornithine decarboxylase | MET | -1.184 | -3.049 | -2.169 |
| CPIJ004370 | nonspecific lipid-transfer protein | MET |  | -0.953 | -1.96 |
| CPIJ006187 | acyl-CoA oxidase | MET | -1.263 | -2.045 | -2.236 |
| CPIJ014172 | 2-hydroxyacyl-CoA lyase 1 | MET |  | -1.911 | -2.184 |
| CPIJ007204 | apolipophorins | MET, TRP | -1.902 | -2.746 | -2.325 |
| CPIJ014059 | NADP-dependent leukotriene B4 12-hydroxydehydrogenase | MET, RSM |  | -1.975 | -2.167 |
| CPIJ003693 | glucosyl/glucuronosyl transferase | MET | -1.551 | -2.821 | -2.466 |
| CPIJ012159 | sphingomyelin phosphodiesterase | MET |  | -1.378 | -2.466 |
| CPIJ015407 | xaa-Pro aminopeptidase 1 | MET |  | -1.115 | -2.438 |
| CPIJ000372 | L-xylulose reductase | MET | -1.518 | -2.94 | -2.249 |
| CPIJ006508 | UDP-glucuronosyltransferase 2B4 | MET | -0.931 | -1.445 | -2.203 |
| CPIJ000994 | juvenile hormone epoxide hydrolase 1 | MET |  | -1.496 | -2.119 |
| CPIJ003184 | l-asparaginase | MET |  |  | -2.051 |
| CPIJ015654 | lysosomal alpha-mannosidase | MET |  | -1.388 | -2.625 |
| CPIJ011630 | C-4 methylsterol oxidase | MET |  | -0.833 | -2.28 |
| CPIJ003185 | gamma-glutamyltranspeptidase 1 | MET | -1.56 | -1.979 | -2.632 |
| CPIJ010716 | luciferin 4-monooxygenase | MET | -1.437 | -2.747 | -2.378 |
| CPIJ003059 | acyl-CoA oxidase | MET |  | -2.551 | -2.537 |
| CPIJ011629 | sterol desaturase | MET |  | -1.123 | -2.251 |
| CPIJ002104 | plasma alpha-L-fucosidase | MET |  | -2.3 | -2.234 |
| CPIJ002718 | lipase 3 | MET |  | -1.104 | -2.489 |
| CPIJ002726 | lipase 3 | MET |  | -1.103 | -2.629 |
| CPIJ003304 | isocitrate dehydrogenase | MET, DIV | -0.83 | -2.882 | -2.723 |
| CPIJ003432 | aldehyde dehydrogenase | MET | -1.194 | -2.765 | -2.723 |
| CPIJ009306 | neutral alpha-glucosidase ab | MET | -0.886 | -3.387 | -2.904 |
| CPIJ014281 | carbonic anhydrase | MET |  | -1.901 | -2.573 |
| CPIJ000992 | juvenile hormone epoxide hydrolase 1 | MET | -0.86 | -2.714 | -2.802 |
| CPIJ003695 | glucosyl/glucuronosyl transferase | MET | -1.545 | -3.22 | -3.644 |
| CPIJ002911 | retinoid-inducible serine carboxypeptidase | MET | -0.751 | -2.391 | -3.592 |
| CPIJ009094 | ornithine decarboxylase 1 | MET | -1.376 | -3.737 | -3.588 |
| CPIJ001556 | acyl-coenzyme A oxidase 3 | MET | -2.45 | -3.944 | -3.673 |
| CPIJ018060 | thymus-specific serine protease | MET, IMM |  | -2.225 | -3.794 |
| CPIJ005231 | N-acetyl galactosaminyl transferase 6 | MET | 2.301 | 2.572 | 1.013 |
| CPIJ013573 | N-acetyl galactosaminyl transferase 6 | MET | 2.066 | 2.34 | 0.842 |
| CPIJ010515 | phosphoenolpyruvate carboxykinase | MET | -0.772 | 1.969 | 1.62 |
| CPIJ008074 | glucosamine-6-phosphate isomerase | MET | 1.568 | 0.82 | 0.83 |
| CPIJ019917 | triacylglycerol lipase | MET | -2.352 | -2.257 | -1.498 |
| CPIJ012030 | 3-hydroxyisobutyryl-coenzyme A hydrolase | MET | -0.79 | -1.403 | -0.849 |
| CPIJ015182 | conserved hypothetical protein | MET | -1.186 | -1.337 | -1.245 |
| CPIJ019120 | chitotriosidase-1 | MET | 1.363 | 1.083 | 0.821 |
| CPIJ006164 | serine palmitoyltransferase 1 | MET | 1.111 | 1.173 | 0.756 |
| CPIJ014557 | nitrilase and fragile histidine triad fusion protein NitFhit | MET | -0.772 | -1.038 | -1.007 |
| CPIJ015409 | cleavage and polyadenylation specificity factor | MET | -0.825 | -1.068 | -0.905 |
| CPIJ003886 | 4-Hydroxybutyrate CoA-transferase | MET |  | -0.9 | -0.84 |
| CPIJ009362 | phosphatidyltransferase | MET |  | -1.22 | -0.958 |
| CPIJ013747 | enoyl-CoA hydratase, mitochondrial | MET |  | -0.768 | -0.78 |
| CPIJ014333 | glucosyl/glucuronosyl transferase | MET |  | -0.835 | -1.056 |
| CPIJ019160 | elongase | MET |  | 0.836 | 0.951 |
| CPIJ015310 | AMP dependent ligase | MET | 0.849 |  | 0.868 |
| CPIJ013307 | aromatic-L-amino-acid decarboxylase | MET | 1.028 |  | -1.236 |
| CPIJ004216 | lipase member I | MET |  |  | -0.917 |
| CPIJ007697 | testicular acid phosphatase | MET |  |  | 1.28 |
| CPIJ000105 | elongase | MET |  |  | 0.851 |
| CPIJ000377 | homogentisate 1,2-dioxygenase | MET |  |  | 0.778 |
| CPIJ001195 | glyoxylate reductase/hydroxypyruvate reductase | MET |  |  | -0.776 |
| CPIJ003544 | phosphatidylinositol synthase | MET |  |  | -0.784 |
| CPIJ007895 | mitochondrial dimethyladenosine transferase 1 | MET |  |  | 1.042 |
| CPIJ011025 | elongation of very long chain fatty acids protein 4 | MET |  |  | 0.817 |
| CPIJ012481 | proacrosin | MET |  |  | 0.929 |
| CPIJ014005 | elongation of very long chain fatty acids protein 1 | MET |  |  | 0.793 |
| CPIJ019193 | dihydroceramide delta | MET |  |  | 0.838 |
| CPIJ002715 | lipase 3 | MET | 2.351 | 3.835 |  |
| CPIJ003148 | cystathionine beta-lyase | MET | 1.071 | 1.064 |  |
| CPIJ005082 | pyrroline-5-carboxylate reductase | MET | 2.076 | 1.354 |  |
| CPIJ000424 | AMP dependent coa ligase | MET | 1.897 | 1.117 |  |
| CPIJ000734 | glutaminyl-tRNA synthetase | MET | -1.111 | -1.201 |  |
| CPIJ001127 | valacyclovir hydrolase | MET | -1.934 | -1.776 |  |
| CPIJ002337 | ribonucleoside-diphosphate reductase small chain | MET | -1.269 | -1.363 |  |
| CPIJ002523 | spermidine synthase | MET | -1.847 | -1.269 |  |
| CPIJ004143 | 1-acylglycerol-3-phosphate O-acyltransferase | MET | 0.887 | 1.043 |  |
| CPIJ005616 | deoxyuridine 5'-triphosphate nucleotidohydrolase | MET | -1.791 | -1.755 |  |
| CPIJ006768 | phosphomannomutase | MET | -2.15 | -2.218 |  |
| CPIJ006772 | 4-nitrophenylphosphatase | MET | -1.21 | -1.138 |  |
| CPIJ008309 | para-hydroxybenzoate-polyprenyltransferase, mitochondrial | MET | -1.283 | -1.403 |  |
| CPIJ008347 | phosphatidylserine decarboxylase | MET | -1.589 | -1.652 |  |
| CPIJ008432 | 1-acylglycerol-3-phosphate acyltransferase | MET | -1.567 | -1.136 |  |
| CPIJ009201 | N-myc downstream regulated | MET | 1.629 | 0.99 |  |
| CPIJ010888 | origin recognition complex subunit 1 | MET | -1.845 | -1.051 |  |
| CPIJ011181 | oligoribonuclease | MET | -1.47 | -1.935 |  |
| CPIJ011415 | chromatin assembly factor 1 subunit B | MET | -1.422 | -0.933 |  |
| CPIJ015136 | dodo | MET | -1.182 | -1.572 |  |
| CPIJ000981 | prohibitin-2 | MET | -0.778 | -1.774 |  |
| CPIJ001725 | NAD dependent epimerase/dehydratase | MET | -0.846 | -0.865 |  |
| CPIJ001828 | papilin | MET | -1.042 | -1.559 |  |
| CPIJ002065 | hydroxymethylglutaryl-CoA synthase 1 | MET | -1.173 | -0.786 |  |
| CPIJ003608 | glycerol-3-phosphate dehydrogenase, mitochondrial | MET | 0.875 | 0.875 |  |
| CPIJ006375 | mannose-6-phosphate isomerase | MET | -0.802 | -1.206 |  |
| CPIJ006486 | glucose-6-phosphate isomerase | MET | -0.815 | -1.724 |  |
| CPIJ012300 | nonsense-mediated mRNA decay protein 1 | MET | 0.786 | 0.969 |  |
| CPIJ013923 | glucosyl/glucuronosyl transferase | MET | -0.844 | -0.832 |  |
| CPIJ014604 | NAD dependent epimerase/dehydratase | MET | -0.765 | -0.885 |  |
| CPIJ002176 | phosphatidylserine synthase | MET | -1.313 | -1.298 |  |
| CPIJ004369 | glucosyl transferase | MET | -1.139 | -0.801 |  |
| CPIJ005288 | SM protein G | MET | -1.388 | -0.801 |  |
| CPIJ013352 | prefoldin subunit 3 | MET | -0.78 | -0.776 |  |
| CPIJ015389 | proliferation-associated 2g4 | MET | -1.25 | -0.83 |  |
| CPIJ000363 | conserved hypothetical protein | MET | -1.229 | -1.106 |  |
| CPIJ005699 | N-acetyl galactosaminyl transferase | MET | -0.958 | -0.772 |  |
| CPIJ006003 | delta-aminolevulinic acid dehydratase | MET | -0.853 | -0.845 |  |
| CPIJ006313 | lipoate-protein ligase | MET | -1.034 | -1.163 |  |
| CPIJ009461 | proline oxidase | MET | -0.806 | -1.094 |  |
| CPIJ010493 | conserved hypothetical protein | MET | -1.393 | -1.478 |  |
| CPIJ011777 | FK506-binding protein 2 | MET | -0.879 | -1.22 |  |
| CPIJ013384 | histone deacetylase 3 | MET | -0.751 | -1.085 |  |
| CPIJ013596 | glycogenin | MET | -1.369 | -1.619 |  |
| CPIJ014099 | methyl enetetrahydrofolate dehydrogenase | MET | -0.864 | -1.022 |  |
| CPIJ014559 | hydrolase | MET | -1.128 | -1.16 |  |
| CPIJ014691 | FK506-binding protein 59 | MET | -1.183 | -1.354 |  |
| CPIJ016638 | glycine cleavage system h protein | MET | -1.094 | -1.665 |  |
| CPIJ017458 | 4-coumarate-CoA ligase 1 | MET | -1.219 | -1.668 |  |
| CPIJ017610 | glucose-6-phosphate isomerase | MET | -0.936 | -1.421 |  |
| CPIJ019627 | F-box/LRR-repeat protein 14 | MET | -0.931 | -0.995 |  |
| CPIJ020026 | glycogen debranching enzyme | MET | -0.945 | -1.113 |  |
| CPIJ002014 | carbonic anhydrase | MET |  | -0.809 |  |
| CPIJ000106 | elongase, puatative | MET |  | -0.872 |  |
| CPIJ000415 | phosphatidyltransferase | MET |  | -1.047 |  |
| CPIJ002859 | isocitrate dehydrogenase | MET |  | -1.217 |  |
| CPIJ005779 | succinyl-CoA ligase beta-chain, mitochondrial | MET |  | -1.212 |  |
| CPIJ006767 | methylglutaconyl-CoA hydratase, mitochondrial | MET |  | -1.116 |  |
| CPIJ008006 | ferrochelatase, mitochondrial | MET |  | -1.137 |  |
| CPIJ009181 | polypeptide N-acetylgalactosaminyltransferase 2 | MET |  | -0.866 |  |
| CPIJ010693 | uroporphyrinogen decarboxylase | MET |  | -0.986 |  |
| CPIJ010991 | neural stem cell-derived dendrite regulator | MET |  | -1.025 |  |
| CPIJ011359 | thymidylate synthase | MET |  | -1.036 |  |
| CPIJ013067 | nitrilase and fragile histidine triad fusion protein NitFhit | MET |  | -1.337 |  |
| CPIJ013145 | prolyl-tRNA synthetase | MET |  | -1.089 |  |
| CPIJ015323 | XTP3-transactivated gene B protein | MET |  | -1.019 |  |
| CPIJ020264 | UDP-glucuronosyltransferase 2B15 | MET |  | -0.791 |  |
| CPIJ000038 | UDP-glucuronosyltransferase 1-3 | MET |  | 2.068 |  |
| CPIJ000239 | taz protein | MET |  | -0.966 |  |
| CPIJ000918 | glucosaminephosphotransferase | MET |  | -0.761 |  |
| CPIJ002114 | elongation of very long chain fatty acids protein 4 | MET |  | -0.933 |  |
| CPIJ002286 | conserved hypothetical protein | MET |  | -0.796 |  |
| CPIJ003282 | metalloproteinase inhibitor 3 | MET |  | 0.818 |  |
| CPIJ003494 | fatty acid synthase S-acetyltransferase | MET |  | -0.958 |  |
| CPIJ004258 | ribose-5-phosphate isomerase | MET |  | -0.76 |  |
| CPIJ004308 | 3-oxoacyl-[acyl-carrier-protein] synthase, mitochondrial | MET |  | -0.855 |  |
| CPIJ004528 | conserved hypothetical protein | MET |  | -0.848 |  |
| CPIJ004539 | adrenodoxin | MET |  | -0.977 |  |
| CPIJ005463 | salivary lipase | MET |  | -0.885 |  |
| CPIJ005550 | centaurin-beta 2 | MET |  | 0.782 |  |
| CPIJ005635 | c-AMP specific cyclic nucleotide phosphodiesterase | MET |  | 1.138 |  |
| CPIJ006300 | alpha | MET |  | 0.766 |  |
| CPIJ008367 | fatty acid synthase S-acetyl transferase | MET |  | -1.022 |  |
| CPIJ008608 | vitellogenic carboxypeptidase | MET |  | -0.812 |  |
| CPIJ010212 | inositol polyphosphate 5-phosphatase | MET |  | 0.918 |  |
| CPIJ010291 | citrate synthase, mitochondrial | MET |  | -1.112 |  |
| CPIJ011392 | serine/threonine-protein kinase BUD32 | MET |  | -1.111 |  |
| CPIJ011633 | short-chain specific acyl-CoA dehydrogenase, mitochondrial | MET |  | -0.881 |  |
| CPIJ012766 | acetyl-coa synthetase | MET |  | -0.869 |  |
| CPIJ012943 | UDP-glucuronosyltransferase 2C1 | MET |  | -0.801 |  |
| CPIJ013034 | pantothenate kinase 4 | MET |  | -0.799 |  |
| CPIJ013435 | molybdenum cofactor synthesis protein 2 large subunit | MET |  | -0.826 |  |
| CPIJ015711 | elongase | MET |  | 0.787 |  |
| CPIJ015716 | 4-coumarate-CoA ligase 1 | MET |  | 0.768 |  |
| CPIJ017721 | gamma-glutamyltranspeptidase | MET |  | 0.805 |  |
| CPIJ018235 | prefoldin subunit 5 | MET |  | -0.991 |  |
| CPIJ011302 | trypsin | MET | 2.09 |  |  |
| CPIJ000427 | AMP dependent coa ligase | MET | 2.2 |  |  |
| CPIJ010518 | phosphoenolpyruvate carboxykinase | MET | -2.085 |  |  |
| CPIJ000297 | 5,10-methylenetetrahydrofolate reductase | MET | 1.217 |  |  |
| CPIJ000426 | acetyl-coenzyme A synthetase | MET | 1.612 |  |  |
| CPIJ002867 | AMP dependent coa ligase | MET | 1.8 |  |  |
| CPIJ010016 | Hsp70-interacting protein | MET | 1.477 |  |  |
| CPIJ012815 | mannose-1-phosphate guanyltransferase | MET | -0.795 |  |  |
| CPIJ009664 | purple acid phosphatase | MET | 0.763 |  |  |
| CPIJ000040 | UDP-glucuronosyltransferase 2B1 | MET | -0.832 |  |  |
| CPIJ004710 | bifunctional protein folD | MET | -0.843 |  |  |
| CPIJ004863 | deoxycytidylate deaminase | MET | -0.785 |  |  |
| CPIJ005343 | glucosylceramidase | MET | 1.711 |  |  |
| CPIJ006210 | FK506-binding protein 6 | MET | -1.161 |  |  |
| CPIJ008090 | trans-prenyltransferase | MET | -0.803 |  |  |
| CPIJ010516 | phosphoenolpyruvate carboxykinase | MET | -1.229 |  |  |
| CPIJ010526 | cysteinyl-tRNA synthetase | MET | -0.85 |  |  |
| CPIJ011300 | lumbrokinase-3 | MET | 0.817 |  |  |
| CPIJ013321 | vitellogenin | MET | 0.757 |  |  |
| CPIJ016440 | dihydroceramide delta | MET | 1.055 |  |  |
| CPIJ017305 | sterol desaturase | MET | 0.763 |  |  |
| CPIJ014619 | zinc binding dehydrogenase | MET, DIV |  | -1.211 | -0.903 |
| CPIJ016451 | crotonobetainyl-CoA dehydrogenase | MET, DIV |  | -2.076 |  |
| CPIJ007255 | cytidylate kinase | MET, DIV |  | -1.408 |  |
| CPIJ006569 | glutaminase | MET, DIV | 1.235 |  |  |
| CPIJ008877 | prolylcarboxypeptidase | MET, PRT | -1.154 | -1.062 |  |
| CPIJ003801 | NADP-dependent leukotriene B4 12-hydroxydehydrogenase | MET, RSM |  |  | -0.933 |
| CPIJ013219 | 3-hydroxybutyrate dehydrogenase type 2 | MET, RSM | -1.163 | -1.451 |  |
| CPIJ006447 | alcohol dehydrogenase | MET, RSM |  | -0.829 |  |
| CPIJ001771 | 3-oxoacyl-[acyl-carrier-protein] reductase | MET, RSM |  | -1.495 |  |
| CPIJ017424 | small nuclear ribonucleoprotein E | MET, RTT | -1.174 |  |  |
| CPIJ013616 | trypsin 5 | DIG | -2.458 | -2.449 | -2.543 |
| CPIJ008904 | alpha-glucosidase | DIG |  | -1.764 | -3.091 |
| CPIJ004339 | salivary C-type lectin | DIG |  | -1.018 | -1.829 |
| CPIJ002066 | alpha-galactosidase A | DIG |  | -2.216 | -1.08 |
| CPIJ008079 | alpha-amylase 1 | DIG |  | -2.53 | -3.071 |
| CPIJ006074 | trypsin-epsilon | DIG |  | -1.717 | -1.691 |
| CPIJ013172 | alpha-glucosidase | DIG |  | -1.185 | -2.906 |
| CPIJ005600 | alpha-N-acetylgalactosaminidase | DIG |  | -1.278 | -1.785 |
| CPIJ012204 | alpha-glucosidase | DIG |  |  | -2.642 |
| CPIJ001464 | alpha-amylase A | DIG |  |  | -1.4 |
| CPIJ005060 | alpha-amylase B | DIG |  |  | -1.326 |
| CPIJ005061 | alpha-amylase B | DIG |  |  | -0.813 |
| CPIJ005725 | alpha-amylase A | DIG |  |  | -2.196 |
| CPIJ006081 | trypsin alpha | DIG |  |  | -1.593 |
| CPIJ013170 | maltase 1 | DIG |  |  | -0.988 |
| CPIJ013171 | alpha-glucosidase | DIG |  |  | -1.397 |
| CPIJ013173 | neutral and basic amino acid transport protein rBAT | DIG |  |  | -1.209 |
| CPIJ018222 | alpha-amylase B | DIG |  |  | -0.941 |
| CPIJ006568 | chymotrypsin 1 | DIG | -0.847 | -1.469 |  |
| CPIJ018802 | endochitinase A | DIG | -2.342 | -1.76 |  |
| CPIJ013165 | 16 kDa salivary peptide | DIG | -1.225 | -1.479 |  |
| CPIJ010945 | acidic mammalian chitinase | DIG |  | -0.872 |  |
| CPIJ017657 | salivary secreted angiopoietin | DIG |  | 1.553 |  |
| CPIJ005065 | alpha-amylase 2 | DIG | -1.012 |  |  |
| CPIJ019598 | basic endochitinase CHB4 | DIG | -2.367 | -1.96 | -0.983 |
| CPIJ003718 | vitamin K-dependent protein C | DIG |  | 0.902 | 1.204 |
| CPIJ015107 | myeloblastin | DIG |  | 1.121 | 0.807 |
| CPIJ002937 | trypsin delta/gamma | DIG |  |  | -1.308 |
| CPIJ005064 | alpha-amylase | DIG |  |  | 0.979 |
| CPIJ018118 | amidase | DIG | -1.206 | -1.662 |  |
| CPIJ002703 | amidase | DIG |  | -1.145 |  |
| CPIJ005160 | serine protease htra2 | DIG |  | -0.806 |  |
| CPIJ019291 | serine protease htra2 | DIG |  | -1.021 |  |
| CPIJ016291 | 16.8 kDa salivary protein | DIG |  | 0.801 |  |
| CPIJ016619 | trypsin-1 | DIG |  | 0.787 |  |
| CPIJ019363 | chitinase domain-containing protein 1 | DIG |  | -0.883 |  |
| CPIJ006073 | trypsin | DIG | 0.795 |  |  |
| CPIJ013082 | chitinase | DIG, MET |  | -1.468 | -1.385 |
| CPIJ002939 | metalloproteinase | PRT |  | 2.39 | 2.445 |
| CPIJ015719 | interstitial collagenase | PRT |  | 1.193 | 1.592 |
| CPIJ003994 | serine collagenase 1 | PRT | 1.549 | 2.246 | 1.651 |
| CPIJ010224 | metalloproteinase | PRT | -3.539 | -4.09 |  |
| CPIJ019029 | metalloproteinase | PRT | -1.997 | -1.397 | -1.344 |
| CPIJ008031 | conserved hypothetical protein | PRT, DIV | 1.829 | 2.053 | 1.342 |
| CPIJ013317 | metalloproteinase | PRT | -1.311 | -1.433 |  |
| CPIJ000593 | coagulation factor XI | PRT |  |  | 1.333 |
| CPIJ013319 | metalloproteinase | PRT | -2.231 | -2.102 |  |
| CPIJ003906 | hedgehog | PRT |  | 1.451 | 1.22 |
| CPIJ006295 | protease m1 zinc metalloprotease | PRT |  | 1.032 | 1.133 |
| CPIJ006578 | paraplegin | PRT |  |  | 1.016 |
| CPIJ012743 | 60S ribosomal protein L7 | PRT, RTT | 0.945 | 1.085 | 0.894 |
| CPIJ010278 | calpain | PRT |  |  | 0.962 |
| CPIJ002647 | cell division protease ftsH | PRT |  |  | 0.872 |
| CPIJ004203 | proteasome subunit beta type 6 | PRT |  | -1.123 |  |
| CPIJ001700 | matrix metalloproteinase | PRT |  | -0.85 | -1.196 |
| CPIJ002841 | leucine aminopeptidase | PRT |  |  | -0.953 |
| CPIJ008387 | aminopeptidase N | PRT | -1.159 | -2.168 | -1.359 |
| CPIJ014109 | conserved hypothetical protein | PRT |  | -0.91 | -1.015 |
| CPIJ009825 | leucyl aminopeptidase | PRT |  | -1.386 | -1.024 |
| CPIJ002136 | serine protease1/2 | PRT, IMM, MET | -1.344 | -1.933 | -0.903 |
| CPIJ019165 | tryptase-2 | PRT, IMM |  |  | -1.836 |
| CPIJ013670 | aminoacylase | PRT | -0.949 | -1.017 | -0.816 |
| CPIJ011997 | zinc carboxypeptidase A 1 | PRT |  | -1.287 | -0.854 |
| CPIJ016973 | conserved hypothetical protein | PRT |  |  | -1.108 |
| CPIJ002944 | meprin A beta-subunit | PRT |  |  | -1.307 |
| CPIJ015253 | zinc carboxypeptidase A 1 | PRT | -0.866 | -2.117 | -1.447 |
| CPIJ002943 | conserved hypothetical protein | PRT |  | -2.161 | -2.229 |
| CPIJ004093 | coagulation factor XI | PRT |  |  | -0.972 |
| CPIJ002135 | trypsin alpha-4 | PRT, IMM |  |  | -2.203 |
| CPIJ009394 | zinc carboxypeptidase | PRT |  | -1.832 | -1.593 |
| CPIJ010137 | zinc carboxypeptidase A 1 | PRT |  | -1.015 | -2.379 |
| CPIJ013669 | aminoacylase | PRT | -1.506 | -1.68 | -1.56 |
| CPIJ002941 | high choriolytic enzyme 1 | PRT |  | -1.266 | -1.644 |
| CPIJ008379 | conserved hypothetical protein | PRT | -1.183 | -1.753 | -2.741 |
| CPIJ011102 | conserved hypothetical protein | PRT |  |  | -1.692 |
| CPIJ009804 | aminoacylase | PRT |  | -1.932 | -1.677 |
| CPIJ010138 | zinc carboxypeptidase A 1 | PRT |  | -2.432 | -2.472 |
| CPIJ008388 | aminopeptidase N | PRT |  | -1.063 | -1.512 |
| CPIJ013671 | aminoacylase | PRT | -1.544 | -0.884 | -1.687 |
| CPIJ015252 | zinc carboxypeptidase | PRT |  | -1.705 | -2.265 |
| CPIJ005457 | neurotrypsin | PRT |  | -0.966 | -0.948 |
| CPIJ013740 | conserved hypothetical protein | PRT |  | -1.565 | -1.96 |
| CPIJ008876 | lysosomal pro-X carboxypeptidase | PRT |  |  | -1.514 |
| CPIJ010803 | zinc-carboxypeptidase | PRT |  |  | -1.651 |
| CPIJ008337 | N | PRT |  |  | -1.822 |
| CPIJ001173 | plasma glutamate carboxypeptidase | PRT |  | -1.104 | -1.979 |
| CPIJ008277 | conserved hypothetical protein | PRT |  | -1.213 | -1.966 |
| CPIJ011433 | brachyurin | PRT, IMM |  |  | -1.779 |
| CPIJ001046 | protease m1 zinc metalloprotease | PRT |  | -1.553 | -2.468 |
| CPIJ004060 | aminopeptidase N | PRT | -1.053 | -2.59 | -2.512 |
| CPIJ008873 | prolylcarboxypeptidase | PRT | -1.879 | -3.401 | -2.623 |
| CPIJ002156 | chymotrypsin BI | PRT, IMM | -1.36 | -2.459 |  |
| CPIJ009106 | angiotensin-converting enzyme | PRT |  |  | -2.053 |
| CPIJ002138 | chymotrypsinogen | PRT |  | -2.192 | -2.573 |
| CPIJ002942 | zinc metalloproteinase nas-12 | PRT |  | -1.679 | -3.41 |
| CPIJ001745 | zinc carboxypeptidase | PRT |  | -1.735 | -3.035 |
| CPIJ002945 | zinc metalloproteinase dpy-31 | PRT |  |  | -2.564 |
| CPIJ001744 | zinc carboxypeptidase | PRT |  | -1.991 | -3.081 |
| CPIJ002133 | trypsin epsilon | PRT | -0.892 | -2.737 | -3.182 |
| CPIJ009738 | conserved hypothetical protein | PRT |  | -1.27 | -2.927 |
| CPIJ010805 | carboxypeptidase A1 | PRT |  | -0.951 | -2.956 |
| CPIJ011383 | serine-type enodpeptidase | PRT, DIG |  | -1.988 | -3.415 |
| CPIJ001240 | cathepsin B-like thiol protease | PRT | -1.452 | -3.876 | -3.431 |
| CPIJ008874 | prolylcarboxypeptidase | PRT |  | -1.999 | -3.32 |
| CPIJ001743 | carboxypeptidase A2 | PRT | -0.766 | -2.291 | -3.767 |
| CPIJ006544 | chymotrypsinogen 2 | PRT | 5.478 | 4.719 | 0.799 |
| CPIJ002126 | chymotrypsin-1 | PRT |  | -3.382 | -4.934 |
| CPIJ007722 | hypothetical protein | PRT | 1.499 | 0.822 | -0.89 |
| CPIJ008264 | proteasome subunit beta type 7 | PRT | -0.953 | -1.433 | -0.863 |
| CPIJ009640 | cytosol aminopeptidase | PRT |  | 0.808 | 0.781 |
| CPIJ018380 | spermatogenesis-associated protein 20 | PRT |  | -0.776 | -0.754 |
| CPIJ002130 | kallikrein-7 | PRT |  |  | -0.784 |
| CPIJ002946 | flavastacin | PRT |  |  | 0.983 |
| CPIJ011626 | signal peptide peptidase | PRT |  |  | -0.795 |
| CPIJ001361 | proteasome subunit beta type 3 | PRT | -1.558 | -1.908 |  |
| CPIJ003987 | proteasome subunit beta type 1 | PRT | -1.006 | -1.372 |  |
| CPIJ008800 | ATP-dependent protease La | PRT | -1.049 | -1.412 |  |
| CPIJ010223 | zinc metalloproteinase nas-8 | PRT | -2.901 | -2.266 |  |
| CPIJ017386 | proteasome subunit beta type 8 | PRT | -0.858 | -1.543 |  |
| CPIJ004903 | presequence protease, mitochondrial | PRT | -0.975 | -0.827 |  |
| CPIJ009721 | ubiquitin carboxyl-terminal hydrolase 34 | PRT | -0.764 | -0.994 |  |
| CPIJ005134 | calpain | PRT | 1.001 | 0.837 |  |
| CPIJ012217 | inositol 1,4,5-trisphosphate receptor | PRT | 0.78 | 0.999 |  |
| CPIJ000894 | proteasome subunit beta type 2 | PRT |  | -1.14 |  |
| CPIJ007721 | hypothetical protein | PRT |  | 1.16 |  |
| CPIJ016441 | ubiquitin carboxyl-terminal hydrolase | PRT |  | -1.159 |  |
| CPIJ016997 | proteasome subunit beta type 5,8 | PRT |  | -1.076 |  |
| CPIJ002112 | serine protease nudel | PRT |  | 0.871 |  |
| CPIJ004086 | angiotensin-converting enzyme | PRT |  | -0.881 |  |
| CPIJ007009 | conserved hypothetical protein | PRT |  | 0.772 |  |
| CPIJ007755 | CAAX prenyl protease 1 | PRT |  | -0.884 |  |
| CPIJ010802 | carboxypeptidase A1 | PRT |  | 0.811 |  |
| CPIJ011309 | conserved hypothetical protein | PRT |  | 0.769 |  |
| CPIJ011448 | leukotriene A-4 hydrolase | PRT |  | -0.953 |  |
| CPIJ013685 | signal peptide peptidase | PRT |  | -0.754 |  |
| CPIJ009142 | coagulation factor VII | PRT | 1.01 |  |  |
| CPIJ002938 | meprin A subunit beta | PRT | 0.836 |  |  |
| CPIJ013625 | conserved hypothetical protein | PRT | -0.909 |  |  |
| CPIJ002772 | conserved hypothetical protein | PRT, DIV | 0.896 | 1.142 |  |
| CPIJ002134 | gamma-renin | PRT, IMM |  |  | -0.861 |
| CPIJ011296 | peroxiredoxin 6 | IMM | 1.227 |  | 2.576 |
| CPIJ012894 | membrane glycoprotein LIG-1 | IMM |  | 2.164 | 2.323 |
| CPIJ014718 | serine protease inhibitor 4, serpin-4 | IMM | 1.266 | 2.704 | 2.604 |
| CPIJ018481 | yellow | IMM | 2.274 | 1.224 | 1.84 |
| CPIJ009057 | caspase-3 | IMM, MET | 0.807 | 1.743 | 1.979 |
| CPIJ007535 | adhesive serine protease | IMM | 1.921 | 1.863 | 2.664 |
| CPIJ012013 | serine protease inhibitor, serpin | IMM | 0.831 | 2.865 | 1.92 |
| CPIJ006716 | peptidoglycan recognition protein sb2 | IMM | -1.189 | 1.488 | 2.207 |
| CPIJ013339 | transferrin | IMM | 1.03 | 0.983 | 1.355 |
| CPIJ019641 | leucine-rich repeat-containing protein 47 | IMM |  | 0.886 | 1.465 |
| CPIJ008252 | caspase | IMM, MET |  | 1.169 | 1.529 |
| CPIJ012579 | Caspase-3 | IMM |  |  | 2.523 |
| CPIJ013424 | PIWI | IMM | 1.054 | 1.99 | 1.654 |
| CPIJ012017 | serine protease | IMM |  |  | 1.145 |
| CPIJ009056 | caspase-3 | IMM |  | 1.803 | 1.594 |
| CPIJ006918 | conserved hypothetical protein | IMM |  | 3.637 | 3.464 |
| CPIJ008581 | sorcin | IMM |  | 0.983 | 1.491 |
| CPIJ001059 | serine protease | IMM |  | 1.908 | 1.434 |
| CPIJ012713 | tryptase gamma | IMM |  |  | 0.879 |
| CPIJ010085 | angiopoietin-1 | IMM |  |  | 1.035 |
| CPIJ007589 | leucine-rich repeat-containing protein 47 | IMM |  |  | 1.191 |
| CPIJ002658 | clip-domain serine protease | IMM | 0.986 | 0.792 | 1.175 |
| CPIJ011775 | serpin B6 | IMM |  | 0.755 | 1.127 |
| CPIJ017792 | serine protease | IMM | 1.485 |  | 1.108 |
| CPIJ018882 | fibrinogen gamma-B chain | IMM |  | 0.776 |  |
| CPIJ003380 | suppressorsof cytokine signalling | IMM | 0.845 | 1.31 | 1.396 |
| CPIJ001546 | profilin | IMM, DIV |  |  | 1.042 |
| CPIJ006471 | cathepsin l | IMM | 1.925 | 1.79 | 1.166 |
| CPIJ004919 | conserved hypothetical protein | IMM |  | 1.147 | 1.3 |
| CPIJ015701 | 32 kDa beta-galactoside-binding lectin lec-3 | IMM | 1.606 | 1.573 | 1.435 |
| CPIJ012580 | Caspase-3 | IMM |  |  | 1.293 |
| CPIJ010731 | lysozyme c-4 | IMM |  | 1.052 | 0.909 |
| CPIJ011423 | yellow | IMM |  |  | 1.128 |
| CPIJ019277 | ficolin-2 | IMM |  |  | 1.083 |
| CPIJ001060 | coagulation factor XI | IMM |  | 0.97 | 0.989 |
| CPIJ007745 | leucine-rich repeat-containing protein 15 | IMM |  |  | 0.97 |
| CPIJ012016 | serine protease inhibitor, serpin | IMM |  |  | 0.9 |
| CPIJ008999 | leucine-rich transmembrane protein | IMM | 0.977 | 1.025 | 1.078 |
| CPIJ013049 | conserved hypothetical protein | IMM |  |  | 0.853 |
| CPIJ010576 | endopin-1 | IMM | -0.895 |  | 0.759 |
| CPIJ013048 | conserved hypothetical protein | IMM |  | 0.95 | 1.085 |
| CPIJ017793 | elegaxobin-2 | IMM |  |  | 0.846 |
| CPIJ007729 | techylectin-5B | IMM |  | 1.239 | 1.259 |
| CPIJ007019 | serine proteinase inhibitor | IMM |  | 0.842 |  |
| CPIJ007730 | techylectin-5A | IMM |  | 0.877 | 1.174 |
| CPIJ012236 | nuclear factor NF-kappa-B p105 subunit | IMM | 1.688 |  | 0.81 |
| CPIJ003759 | serine protease inhibitor | IMM |  |  | 0.884 |
| CPIJ004893 | bax inhibitor | IMM |  |  | 0.975 |
| CPIJ008093 | caspase-1 | IMM | -0.81 |  |  |
| CPIJ008700 | yellow | IMM | 0.977 | 0.95 |  |
| CPIJ016297 | serine protease inhibitor A3K | IMM |  |  | 0.84 |
| CPIJ007498 | leucine-rich transmembrane protein | IMM |  | 0.9 | 1.179 |
| CPIJ010562 | aromatic amino acid decarboxylase | IMM, MET |  | 1.433 | 0.854 |
| CPIJ002442 | mitochondrial precursor protein import receptor | IMM, RSM |  | -0.836 | -0.901 |
| CPIJ006041 | angiopoietin-2 | IMM |  |  | -0.832 |
| CPIJ007579 | peroxidase | IMM | -1.099 | -1.94 | -1.528 |
| CPIJ016031 | superoxide dismutase | IMM |  |  | -0.807 |
| CPIJ010495 | carboxypeptidase N subunit 2 | IMM |  | -0.967 |  |
| CPIJ017039 | cd36 antigen | IMM | -1.218 | -1.026 | -0.962 |
| CPIJ019787 | ficolin-3 | IMM | -1.747 | -1.962 | -1.466 |
| CPIJ015786 | programmed cell death | IMM, DIV |  |  | -1.089 |
| CPIJ010730 | lysozyme P | IMM |  |  | -0.95 |
| CPIJ000194 | conserved hypothetical protein | IMM |  | -1.279 | -0.86 |
| CPIJ016771 | peptidoglycan recognition protein | IMM |  |  | -1.195 |
| CPIJ000195 | conserved hypothetical protein | IMM | -1.213 | -1.397 | -0.889 |
| CPIJ002382 | Misexpression suppressor of KSR | IMM | -0.755 | -0.936 | -1.008 |
| CPIJ008014 | oxidase/peroxidase | IMM | -0.827 | -1.607 | -0.773 |
| CPIJ016566 | phenoloxidase subunit 1 | IMM |  |  | -0.943 |
| CPIJ013538 | ficolin-1 | IMM |  | -1.283 |  |
| CPIJ002098 | chymotrypsin BI | IMM |  | -1.247 |  |
| CPIJ004325 | gram-negative bacteria binding protein | IMM | -1.79 | -1.728 | -1.287 |
| CPIJ007783 | arylphorin subunit alpha | IMM | 1.312 | -1.19 | -1.091 |
| CPIJ000937 | techylectin-5B | IMM |  | -1.291 | -1.722 |
| CPIJ004229 | gram negative bacteria binding protein 2 | IMM |  | -2.023 | -1.346 |
| CPIJ015785 | transferrin | IMM |  | -0.962 |  |
| CPIJ011413 | peroxiredoxin-4 | IMM |  |  | -1.316 |
| CPIJ004719 | superoxide dismutase 3.4, mitochondrial | IMM | -0.894 | -2.095 | -1.314 |
| CPIJ012252 | croquemort | IMM |  |  | -1.648 |
| CPIJ002747 | conserved hypothetical protein | IMM |  | -0.958 | -1.317 |
| CPIJ016551 | caspase-1 | IMM |  |  | -1.348 |
| CPIJ012151 | trypsin | IMM, PRT |  |  | -1.617 |
| CPIJ002132 | serine-type enodpeptidase | IMM, MET, PRT |  |  | -1.645 |
| CPIJ006811 | salivary C-type lectin | IMM |  |  | -2.464 |
| CPIJ012829 | fibrinogen and fibronectin | IMM |  |  | -1.132 |
| CPIJ011918 | conserved hypothetical protein | IMM |  |  | -1.661 |
| CPIJ001385 | glutamate decarboxylase | IMM, MET | -1.376 | -2.069 | -1.771 |
| CPIJ004127 | macroglobulin/complement | IMM | -2.228 | -3.349 | -2.566 |
| CPIJ010092 | ficolin-3 | IMM | -0.964 | -1.477 | -1.226 |
| CPIJ012833 | ficolin-1 | IMM |  |  | -2.164 |
| CPIJ001276 | defensin-A | IMM | -1.808 | -1.86 | -2.2 |
| CPIJ004947 | leucine-rich repeat-containing protein 1 | IMM | -0.908 | -2.155 | -1.408 |
| CPIJ011617 | chymotrypsinogen | IMM, PRT |  |  | -1.434 |
| CPIJ003613 | beta-1,3-glucan-binding protein | IMM |  | -0.892 | -1.698 |
| CPIJ011917 | conserved hypothetical protein | IMM |  | -1.318 | -1.754 |
| CPIJ000577 | cathepsin B | IMM, PRT |  |  | -1.205 |
| CPIJ004095 | serine protease | IMM |  | -1.203 | -2.031 |
| CPIJ012831 | fibrinogen and fibronectin | IMM | -2.434 |  | -2.705 |
| CPIJ004323 | gram-negative bacteria binding protein | IMM |  | -0.916 | -2.031 |
| CPIJ009033 | arylphorin subunit C223 | IMM |  | -1.127 | -1.643 |
| CPIJ002128 | mast cell protease 2 | IMM |  | -1.47 | -2.324 |
| CPIJ003103 | croquemort | IMM | -1.694 | -1.646 | -2.108 |
| CPIJ002131 | urokinase-type plasminogen activator | IMM, MET, PRT |  | -1.918 | -2.681 |
| CPIJ008450 | peroxiredoxin-6 | IMM | -2.161 | -4.72 | -3.555 |
| CPIJ004321 | gram-negative bacteria binding protein | IMM |  |  | -2.114 |
| CPIJ004324 | gram-negative bacteria binding protein | IMM |  | -1.579 | -2.773 |
| CPIJ002129 | trypsin alpha-4 | IMM, PRT |  | -0.941 | -2.505 |
| CPIJ018824 | larval serum protein 1 beta chain | IMM |  | -2.115 | -3.029 |
| CPIJ006538 | larval serum protein 1 beta chain | IMM |  | -2.276 | -3.361 |
| CPIJ000056 | larval serum protein 1 beta chain | IMM |  | -1.152 | -2.977 |
| CPIJ001820 | larval serum protein 2 | IMM |  | -1.953 | -3.169 |
| CPIJ007044 | catalase | IMM |  | -2.127 | -3.208 |
| CPIJ009032 | larval serum protein 2 | IMM | -2.176 | -3.039 | -3.473 |
| CPIJ000434 | microfibril-associated glycoprotein 4 | IMM | -2.026 |  | -3.922 |
| CPIJ002737 | MPA2 allergen | IMM | 1.486 |  | -1.424 |
| CPIJ001822 | larval serum protein 2 | IMM |  | -2.722 | -3.816 |
| CPIJ001239 | cathepsin B | IMM | -1.274 | -3.74 | -2.951 |
| CPIJ004320 | gram-negative bacteria-binding protein 1 | IMM | -1.053 | -2.814 | -3.981 |
| CPIJ009506 | hexamerin 2 beta | IMM | -1.931 | -3.591 | -4.574 |
| CPIJ017588 | peroxidase | IMM | -1.066 | -1.91 | -1.359 |
| CPIJ012830 | fibrinogen and fibronectin | IMM | -1.038 | 2.351 | -2.545 |
| CPIJ004894 | bax inhibitor | IMM | 0.837 | 0.976 | 0.856 |
| CPIJ010699 | cecropin A | IMM | -1.305 | -1.149 | -1.761 |
| CPIJ006014 | serine protease | IMM | -1.055 | -0.988 | -1.036 |
| CPIJ006392 | fibrinogen and fibronectin | IMM | -1.562 | -1.587 | -1.1 |
| CPIJ002102 | apoptosis 1 inhibitor | IMM |  | 1.136 | 1.066 |
| CPIJ002393 | dopachrome-conversion enzyme | IMM |  | -1.003 | -1.25 |
| CPIJ007868 | antifreeze protein | IMM |  | 1.145 | 0.837 |
| CPIJ015640 | ficolin-3 | IMM |  | -0.899 | -0.903 |
| CPIJ020296 | microfibril-associated glycoprotein 4 | IMM |  | 0.753 | 0.88 |
| CPIJ008656 | angiopoietin-1 | IMM | 1.127 |  | 0.847 |
| CPIJ005451 | lysozyme | IMM | -0.798 |  | -0.849 |
| CPIJ006919 | predicted protein | IMM | -1.094 |  | 0.916 |
| CPIJ020116 | conserved hypothetical protein | IMM |  |  | -0.879 |
| CPIJ002395 | dopachrome conversion enzyme | IMM |  |  | 1.103 |
| CPIJ019595 | fibrinogen and fibronectin | IMM |  |  | 1.006 |
| CPIJ000938 | techylectin-5B | IMM |  |  | -1.6 |
| CPIJ006092 | conserved hypothetical protein | IMM |  |  | 0.881 |
| CPIJ007869 | galactose-specific C-type lectin | IMM |  |  | 1.521 |
| CPIJ008065 | caspase-1 | IMM |  |  | -0.882 |
| CPIJ016012 | tryptase-2 | IMM |  |  | -2.24 |
| CPIJ000574 | cathepsin L | IMM | 0.847 | 1.298 |  |
| CPIJ008547 | myd88 | IMM | 0.793 | 0.758 |  |
| CPIJ009679 | peroxiredoxin-2 | IMM | -0.904 | -1.806 |  |
| CPIJ018307 | myd88 | IMM | 0.77 | 0.823 |  |
| CPIJ012722 | leucine-rich transmembrane protein | IMM | -0.894 | -1.242 |  |
| CPIJ000443 | galactose-specific C-type lectin | IMM | -0.751 | -0.907 |  |
| CPIJ000665 | galectin | IMM | -0.877 | -1.067 |  |
| CPIJ002531 | serine protease | IMM | -1.042 | -1.032 |  |
| CPIJ003399 | peroxiredoxins, prx-1, prx-2, prx-3 | IMM | -0.85 | -0.888 |  |
| CPIJ004042 | plasma kallikrein | IMM | -0.801 | -0.851 |  |
| CPIJ006047 | secreted glutathione peroxidase | IMM | -1.038 | -1.305 |  |
| CPIJ006515 | Toll9 | IMM | 1.391 | 1.121 |  |
| CPIJ006694 | survivin | IMM | -0.827 | -0.95 |  |
| CPIJ004652 | conserved hypothetical protein | IMM |  | 0.887 |  |
| CPIJ005274 | piwi | IMM |  | 1.026 |  |
| CPIJ007727 | fibrinogen and fibronectin | IMM |  | 0.868 |  |
| CPIJ002281 | sptzle 6 | IMM |  | -0.97 |  |
| CPIJ009877 | phenoloxidase subunit 1 | IMM |  | -0.916 |  |
| CPIJ012187 | conserved hypothetical protein | IMM |  | -0.778 |  |
| CPIJ015742 | conserved hypothetical protein | IMM |  | -1.031 |  |
| CPIJ018036 | serine protease | IMM |  | -0.784 |  |
| CPIJ000146 | superoxide dismutase 2 | IMM | 0.755 |  |  |
| CPIJ016084 | gambicin | IMM | -2.747 |  |  |
| CPIJ005191 | leucine-rich repeat-containing protein 15 | IMM | 0.856 |  |  |
| CPIJ007509 | mitotic protein phosphatase 1 regulator | IMM | -0.772 |  |  |
| CPIJ006561 | peptidoglycan recognition protein-lc | IMM | 1.111 |  |  |
| CPIJ001747 | tep3 | IMM | 0.785 |  |  |
| CPIJ003717 | vitamin K-dependent protein C | IMM | -1.124 |  |  |
| CPIJ003752 | ficolin-2 | IMM | -0.838 |  |  |
| CPIJ005192 | conserved hypothetical protein | IMM | -0.986 |  |  |
| CPIJ006150 | Toll9 | IMM | 0.853 |  |  |
| CPIJ008807 | ficolin-1 | IMM | -0.963 |  |  |
| CPIJ009480 | tryptase gamma | IMM | -0.927 |  |  |
| CPIJ010701 | cecropin | IMM | -0.801 |  |  |
| CPIJ012040 | ficolin-1 | IMM | 1.353 |  |  |
| CPIJ001764 | chorion peroxidase | IMM, DIV | 2.032 | 0.842 |  |
| CPIJ007672 | leucine-rich repeat-containing protein 4B | IMM, DIV |  | 0.892 |  |
| CPIJ010925 | heat shock protein 70 | IMM, DIV |  | -0.848 |  |
| CPIJ002603 | bacteria responsive protein 1 | IMM, DIV | -1.016 |  |  |
| CPIJ002518 | chymotrypsin 1 | IMM, PRT | 1.276 |  |  |
| CPIJ008261 | hypothetical protein | RSM | 1.012 | 3.312 | 3.462 |
| CPIJ001156 | mitogen-activated protein kinase 8 | RSM |  | 2.551 | 2.361 |
| CPIJ014221 | cytochrome P450 3A19 | RSM |  | 2.531 | 2.412 |
| CPIJ014220 | cytochrome P450 52D1 | RSM | 0.98 | 2.92 | 2.518 |
| CPIJ010536 | cytochrome P450 9b2 | RSM | 2.402 | 2.318 | 1.971 |
| CPIJ010175 | cytochrome P450 9b1 | RSM | 2.232 | 2.628 | 1.6 |
| CPIJ001157 | jnk | RSM |  | 2.313 | 1.946 |
| CPIJ011081 | heat shock protein 70 B2 | RSM | 0.93 | 4.011 | 4.177 |
| CPIJ002683 | glutathione S-transferase 1-1 | RSM | 1.053 | 2.055 | 1.804 |
| CPIJ015075 | heat shock protein 83 | RSM | -2.203 | -1.732 | 1.417 |
| CPIJ001738 | 39S ribosomal protein L28, mitochondrial | RSM |  |  | 1.77 |
| CPIJ000418 | conserved hypothetical protein | RSM | -1.216 |  | 1.792 |
| CPIJ007418 | cytochrome b561 | RSM | 1.687 | 1.405 | 1.348 |
| CPIJ016849 | cytochrome P450 71B36 | RSM | 0.794 | 2.312 | 1.119 |
| CPIJ019950 | mitochondrial ribosomal protein, L28 | RSM |  |  | 1.52 |
| CPIJ011246 | heat shock protein 83 | RSM | -1.495 | -1.429 | 1.004 |
| CPIJ011244 | heat shock protein 83 | RSM | -1.329 | -1.303 | 0.849 |
| CPIJ007338 | conserved hypothetical protein | RSM |  | 1.152 | 1.058 |
| CPIJ014219 | cytochrome P450 | RSM | 0.751 | 0.856 | 1.484 |
| CPIJ003064 | ribosomal protein S6 kinase | RSM |  |  | 1.173 |
| CPIJ006043 | methionine synthase reductase, mitochondrial | RSM |  | 0.838 | 1.025 |
| CPIJ018407 | cytochrome b561 | RSM | 1.337 | 1.287 | 1.039 |
| CPIJ013919 | xanthine dehydrogenase/oxidase | RSM |  | -0.783 | 0.757 |
| CPIJ007424 | juvenile hormone esterase | RSM |  |  | 0.752 |
| CPIJ017734 | cryptochrome-1 | RSM |  |  | 1.11 |
| CPIJ018318 | short-chain dehydrogenase | RSM |  | 0.76 | 1.028 |
| CPIJ006950 | cytochrome P450 | RSM |  | 1.259 |  |
| CPIJ012754 | microsomal glutathione transferase GSTMIC2 | RSM |  |  | 0.794 |
| CPIJ011247 | heat shock protein 82 | RSM | -1.855 | -1.386 |  |
| CPIJ014336 | cytochrome P450 | RSM |  |  | 1.265 |
| CPIJ002566 | mitochondrial import inner membrane translocase subunit TIM16 | RSM |  |  | 0.775 |
| CPIJ008936 | cytochrome P450 4V3 | RSM |  |  | -0.893 |
| CPIJ011017 | heat shock protein | RSM | -1.121 | -1.973 | -0.9 |
| CPIJ018667 | NADH dehydrogenase flavoprotein 2, mitochondrial | RSM |  | -1.298 |  |
| CPIJ015685 | 3-oxoacyl-[acyl-carrier-protein] reductase | RSM |  |  | -0.963 |
| CPIJ003394 | aldose reductase | RSM |  | -1.215 | -0.84 |
| CPIJ018624 | glutathione-s-transferase theta, gst | RSM | -1.341 | -1.669 | -1.139 |
| CPIJ009476 | cytochrome P450 | RSM |  | -0.772 | -0.863 |
| CPIJ007225 | 3-ketodihydrosphingosine reductase | RSM | -0.78 | -1.107 | -0.866 |
| CPIJ008190 | 39S ribosomal protein L14, mitochondrial | RSM | -0.958 | -1.305 | -1.054 |
| CPIJ017840 | 39S ribosomal protein L12, mitochondrial | RSM |  | -1.322 | -1.042 |
| CPIJ002676 | glutathione S-transferase D7 | RSM |  | -1.125 | -1.151 |
| CPIJ002342 | 3-ketoacyl-CoA thiolase | RSM |  | -0.982 | -0.857 |
| CPIJ019242 | mitochondrial carrier | RSM |  | -1.061 | -1.107 |
| CPIJ003709 | thioredoxin, mitochondrial | RSM | -0.775 | -1.424 | -0.925 |
| CPIJ005135 | peroxisome assembly factor 1 | RSM |  | -1.351 | -1.025 |
| CPIJ010814 | glutathione S-transferase 1-5 | RSM |  | -1.396 | -1.117 |
| CPIJ004342 | mitochondrial uncoupling protein | RSM |  |  | -0.898 |
| CPIJ008445 | amine oxidase | RSM | -1.351 | -1.801 | -0.885 |
| CPIJ007829 | alpha-esterase | RSM | -1.316 | -1.702 | -0.958 |
| CPIJ001372 | bphl protein | RSM |  | -1.198 | -1.073 |
| CPIJ010934 | conserved hypothetical protein | RSM | -0.807 | -1.07 | -1.093 |
| CPIJ019024 | cytochrome c | RSM | -2.279 | -2.817 | -0.993 |
| CPIJ016850 | cytochrome P450 6a9 | RSM |  | -1.177 | -0.844 |
| CPIJ009186 | complement component | RSM | -1.803 | -2.36 | -0.915 |
| CPIJ016336 | esterase B1 | RSM |  | -0.793 | -0.932 |
| CPIJ007967 | mitochondrial import receptor subunit tom20 | RSM |  | -1.731 | -1.21 |
| CPIJ015686 | cyclopentanol dehydrogenase | RSM |  |  | -1.036 |
| CPIJ007824 | esterase B1 | RSM |  |  | -0.929 |
| CPIJ014785 | mitochondrial ribosomal protein, L46 | RSM |  | -1.352 | -1.062 |
| CPIJ013714 | peroxisomal | RSM |  | -1.12 | -0.929 |
| CPIJ002536 | cytochrome P450 6j1 | RSM |  |  | -1.172 |
| CPIJ000298 | cytochrome P450 6B7 | RSM |  |  | -0.834 |
| CPIJ003722 | aldo-keto reductase | RSM |  |  | -1.027 |
| CPIJ017014 | cytochrome P450 6A1 | RSM |  | -1.858 | -1.3 |
| CPIJ019673 | cytochrome P450 6A1 | RSM |  | -1.535 | -1.266 |
| CPIJ018494 | cytochrome P450 6B1 | RSM | -0.753 | -1.686 | -1.055 |
| CPIJ018666 | NADH dehydrogenase flavoprotein 2, mitochondrial | RSM |  | -1.102 | -1.045 |
| CPIJ019817 | cytochrome c oxidase assembly protein COX19 | RSM | -1.261 | -1.536 | -1.067 |
| CPIJ020082 | cytochrome P450 | RSM |  |  | -0.817 |
| CPIJ008447 | amine oxidase | RSM |  | -0.756 |  |
| CPIJ005959 | cytochrome P450 93A3 | RSM |  | -1.347 | -1.195 |
| CPIJ005551 | short-chain dehydrogenase | RSM |  | -1.696 | -2.327 |
| CPIJ001755 | cytochrome P450 4p1 | RSM |  | -1.083 | -1.308 |
| CPIJ015280 | cytochrome c oxidase assembly protein COX11, mitochondrial | RSM | -1.2 | -1.907 | -1.44 |
| CPIJ004595 | cytochrome b5 | RSM | -0.966 | -1.184 | -0.878 |
| CPIJ015681 | cytochrome P450 4d1 | RSM | 0.923 |  | -0.802 |
| CPIJ018241 | microsomal glutathione S-transferase 1 | RSM | -1.122 | -1.476 | -1.38 |
| CPIJ019705 | cytochrome P450 6a22 | RSM | -0.829 | -1.534 | -1.239 |
| CPIJ010539 | cytochrome P450 52A5 | RSM |  |  | -0.972 |
| CPIJ010225 | cytochrome P450 12b1, mitochondrial | RSM |  | -1.549 | -1.141 |
| CPIJ018633 | glutathione-s-transferase theta | RSM | -1.273 | -2.04 | -1.107 |
| CPIJ003377 | cytochrome P450 | RSM |  | -0.919 | -1.079 |
| CPIJ010543 | cytochrome P450 17A1 | RSM | -1.998 | -2.31 | -1.61 |
| CPIJ010075 | cytochrome P450 4c3 | RSM | -0.894 | -1.521 | -1.264 |
| CPIJ016341 | alpha-esterase | RSM | -1.28 | -1.642 | -1.272 |
| CPIJ002253 | mitochondrial carnitine/acylcarnitine carrier protein | RSM |  | -1.549 | -1.137 |
| CPIJ013918 | esterase B1 | RSM |  | -1.382 | -1.324 |
| CPIJ010826 | cytochrome P450 | RSM |  |  | -1.104 |
| CPIJ014579 | cytochrome P450 4d1 | RSM | -0.927 | -1.557 | -1.074 |
| CPIJ016921 | 3-oxoacyl-[acyl-carrier-protein] reductase 1 | RSM | -1.64 | -1.753 | -1.102 |
| CPIJ016212 | glutathione S-transferase 1 | RSM |  | -1.231 | -1.433 |
| CPIJ017462 | cytochrome P450 6A1 | RSM |  | -0.952 | -1.903 |
| CPIJ005955 | cytochrome P450 11A1, mitochondrial | RSM |  | -1.687 | -1.371 |
| CPIJ010548 | cytochrome P450 3A19 | RSM |  | -1.875 | -1.411 |
| CPIJ010227 | cytochrome P450 12b1, mitochondrial | RSM |  | -1.496 | -1.24 |
| CPIJ012932 | spermine oxidase | RSM |  | -1.495 | -1.297 |
| CPIJ016926 | cytochrome B5 | RSM |  | -0.897 | -1.42 |
| CPIJ003082 | cytochrome P450 9b1 | RSM |  | -0.815 | -1.587 |
| CPIJ020018 | cytochrome P450 6d3 | RSM |  | -0.803 | -1.242 |
| CPIJ017288 | mitochondrial solute carrier protein | RSM |  |  | -1.142 |
| CPIJ019834 | mitochondrial carnitine/acylcarnitine carrier protein | RSM |  | -1.638 | -1.27 |
| CPIJ016851 | cytochrome P450 6a9 | RSM |  | -2.132 | -1.463 |
| CPIJ001380 | cytochrome P450 | RSM | -2.135 | -1.589 | -1.301 |
| CPIJ014125 | short-chain dehydrogenase | RSM |  | -1.039 | -1.328 |
| CPIJ012909 | 3-oxoacyl-[acyl-carrier-protein] reductase | RSM |  | -1.658 | -1.312 |
| CPIJ016340 | esterase 6 | RSM |  | -1.698 | -1.401 |
| CPIJ003361 | cytochrome P450 6a8 | RSM |  |  | -1.353 |
| CPIJ007943 | peroxiredoxin 5, prdx5 | RSM | -1.297 | -2.032 | -1.663 |
| CPIJ012756 | microsomal glutathione s-transferase | RSM |  | -2.003 | -1.678 |
| CPIJ000047 | carboxylesterase | RSM |  | -1.136 | -1.55 |
| CPIJ016763 | short-chain dehydrogenase | RSM |  |  | -1.665 |
| CPIJ013920 | aldehyde oxidase | RSM |  | -1.753 | -1.657 |
| CPIJ010904 | conserved hypothetical protein | RSM | -1.295 | -2.428 | -1.239 |
| CPIJ001759 | cytochrome P450 4c21 | RSM | -0.964 | -2.357 | -1.758 |
| CPIJ001121 | kynurenine formamidase | RSM |  | -1.642 | -1.815 |
| CPIJ003677 | NADPH FAD oxidoreductase | RSM |  | -1.435 | -0.898 |
| CPIJ013796 | 3,2-trans-enoyl-CoA isomerase, mitochondrial | RSM |  | -1.815 | -1.721 |
| CPIJ010480 | cytochrome P450 4C1 | RSM |  | -1.634 | -1.569 |
| CPIJ013934 | xanthine dehydrogenase/oxidase | RSM |  | -1.585 | -1.693 |
| CPIJ002538 | cytochrome p450 family protein 44A1 | RSM |  | -0.786 | -1.284 |
| CPIJ006160 | glutathione s-transferase | RSM |  | -0.978 | -1.668 |
| CPIJ017085 | 2-oxoglutarate dehydrogenase E1 component | RSM |  | -1.118 | -1.604 |
| CPIJ005956 | cytochrome P450 | RSM |  | -0.828 | -1.358 |
| CPIJ011127 | cytochrome P450 4d1 | RSM | -1.391 | -1.078 | -1.551 |
| CPIJ003305 | NAD | RSM |  | -2.089 | -1.843 |
| CPIJ019179 | ninjurin a | RSM | -0.915 | -2.162 | -1.749 |
| CPIJ000284 | peroxisomal membrane protein | RSM |  | -1.663 | -1.929 |
| CPIJ020199 | cytochrome P450 | RSM | -1.465 | -2.04 | -1.693 |
| CPIJ016356 | cytochrome P450 6a9 | RSM | -0.993 | -1.545 | -1.805 |
| CPIJ016854 | cytochrome P450 | RSM | -0.812 | -2.348 | -1.899 |
| CPIJ014776 | peroxisomal targeting signal 2 receptor | RSM |  |  | -1.76 |
| CPIJ016855 | cytochrome P450 4A6 | RSM | -1.282 | -2 | -1.907 |
| CPIJ011768 | short-chain dehydrogenase | RSM |  | -1.155 | -1.854 |
| CPIJ006159 | glutathione-requiring prostaglandin D synthase | RSM | -1.251 | -1.868 | -2.174 |
| CPIJ007828 | bile salt-activated lipase | RSM |  | -2.039 | -1.827 |
| CPIJ007825 | para-nitrobenzyl esterase | RSM |  | -1.757 | -1.639 |
| CPIJ012142 | peroxisomal targeting signal 2 receptor | RSM |  |  | -1.811 |
| CPIJ005954 | cytochrome P450 71B38 | RSM |  | -1.445 | -1.62 |
| CPIJ003376 | cytochrome P450 71D6 | RSM |  | -1.812 | -1.977 |
| CPIJ001757 | cytochrome P450 4d1 | RSM | -0.895 | -2.139 | -1.992 |
| CPIJ012470 | cytochrome P450 9b2 | RSM |  | -1.682 | -2.313 |
| CPIJ007010 | peroxisomal membrane protein pmp34 | RSM | -1.744 | -1.935 | -2.146 |
| CPIJ015428 | cytochrome P450 3A19 | RSM |  | -1.326 | -1.846 |
| CPIJ009474 | cytochrome P450 4d10 | RSM | 1.308 |  | -1.414 |
| CPIJ004411 | cytochrome P450 6d3 | RSM |  | -1.701 | -1.853 |
| CPIJ015429 | conserved hypothetical protein | RSM |  | -1.594 | -1.865 |
| CPIJ019572 | glutathione transferase AtGST | RSM | -1.861 | -2.301 | -1.794 |
| CPIJ005957 | cytochrome P450 | RSM | -1.404 | -3.188 | -2.359 |
| CPIJ013917 | esterase B1 | RSM | -1.804 | -2.705 | -2.169 |
| CPIJ000993 | juvenile hormone epoxide hydrolase 1 | RSM |  | -1.686 | -2.047 |
| CPIJ000503 | mitochondrial ATPase inhibitor | RSM |  | -1.247 | -2.118 |
| CPIJ000670 | 24-dehydrocholesterol reductase | RSM | -1.596 | -2.586 | -2.095 |
| CPIJ018627 | glutathione S-transferase 1-1 | RSM |  | -2.105 | -2.276 |
| CPIJ005958 | cytochrome P450 82A2 | RSM |  | -1.713 | -2.078 |
| CPIJ004365 | xanthine dehydrogenase | RSM | -1.357 | -2.354 | -2.231 |
| CPIJ004637 | glutactin | RSM | -1.262 | -2.096 | -1.907 |
| CPIJ018632 | glutathione-s-transferase theta, gst | RSM |  | -1.226 | -1.987 |
| CPIJ005531 | conserved hypothetical protein | RSM | -1.022 | -1.9 | -2.141 |
| CPIJ014052 | glutathione-s-transferase theta, gst | RSM |  | -1.464 | -2.528 |
| CPIJ009733 | glucose 1-dehydrogenase 2 | RSM | -1.168 | -2.773 | -2.27 |
| CPIJ014051 | glutathione-s-transferase theta, gst | RSM |  | -1.99 | -2.115 |
| CPIJ016857 | cytochrome P450 | RSM | -1.523 | -3.03 | -2.511 |
| CPIJ010542 | cytochrome P450 1A1 | RSM | -1.804 | -3 | -2.761 |
| CPIJ020229 | cytochrome P450 4d8 | RSM |  | -1.684 | -2.256 |
| CPIJ005953 | cytochrome P450 CYP6BB1v2 | RSM | -1.209 | -2.341 | -2.129 |
| CPIJ004600 | oxidoreductase | RSM | -1.495 | -2.524 | -2.686 |
| CPIJ006908 | carboxylesterase-6 | RSM |  | -1.189 | -2.523 |
| CPIJ009473 | cytochrome P450 4d10 | RSM |  | -0.963 | -2.213 |
| CPIJ004636 | para-nitrobenzyl esterase | RSM | 1.709 |  | -2.729 |
| CPIJ003375 | cytochrome P450 | RSM |  | -1.601 | -2.505 |
| CPIJ000299 | cytochrome P450 6B6 | RSM |  | -1.993 | -3.018 |
| CPIJ001886 | cytochrome P450 4C1 | RSM | -1.411 | -2.384 | -2.546 |
| CPIJ009478 | cytochrome P450 | RSM |  | -2.125 | -2.834 |
| CPIJ005899 | cytochrome P450 6A1 | RSM | -2.018 | -3.008 | -2.919 |
| CPIJ016922 | serine 3-dehydrogenase | RSM | -1.988 | -2.902 | -2.66 |
| CPIJ010547 | cytochrome P450 9b2 | RSM |  | -2.723 | -2.92 |
| CPIJ016284 | cytochrome P450 4C1 | RSM |  | -2.038 | -2.721 |
| CPIJ000318 | cytochrome b5 | RSM | -2.959 | -3.198 | -3.479 |
| CPIJ004599 | 3-oxoacyl-[acyl-carrier-protein] reductase | RSM | -2.125 | -3.115 | -2.718 |
| CPIJ005332 | cytochrome P450 9b1 | RSM |  | -2.243 | -2.675 |
| CPIJ007188 | cytochrome P450 4d8 | RSM |  | -2.006 | -2.992 |
| CPIJ001758 | cytochrome P450 4d1 | RSM | -1.719 | -3.335 | -3.259 |
| CPIJ010545 | cytochrome P450 9b1 | RSM | -1.1 | -2.768 | -3.382 |
| CPIJ018231 | carboxylesterase | RSM |  | -2.365 | -3.743 |
| CPIJ010544 | cytochrome P450 9b2 | RSM | -2.007 | -3.336 | -4.037 |
| CPIJ005655 | oxidoreductase | RSM | -1.967 | -3.469 | -3.808 |
| CPIJ010858 | cytochrome P450 6a22 | RSM | -1.691 | -3.644 | -4.01 |
| CPIJ002537 | cytochrome P450 26B1 | RSM | -1.951 | -4.23 | -4.902 |
| CPIJ009415 | cytochrome P450 4g15 | RSM | -2.01 | -2.714 | -1.465 |
| CPIJ015053 | mitochondrial 28S ribosomal protein S25 | RSM | -1.169 | -1.581 | -0.987 |
| CPIJ016339 | liver carboxylesterase 1 | RSM | -0.771 | -0.814 | -0.775 |
| CPIJ016852 | cytochrome P450 6B5 | RSM |  | -0.875 | -0.843 |
| CPIJ019704 | cytochrome P450 6A1 | RSM |  | -0.79 | -1.045 |
| CPIJ004752 | esterase B1 | RSM |  | -0.972 | -0.754 |
| CPIJ007370 | aldose reductase | RSM |  | -0.872 | -0.915 |
| CPIJ011250 | mitochondrial carrier | RSM |  | -1.067 | -0.897 |
| CPIJ016847 | cytochrome P450 71B11 | RSM |  | -0.935 | -0.891 |
| CPIJ017813 | spermine oxidase | RSM |  | -0.836 | -0.843 |
| CPIJ012226 | augmenter of liver regeneration | RSM | -0.949 |  | 0.991 |
| CPIJ016853 | cytochrome P450 4F12 | RSM | -1.299 |  | -0.811 |
| CPIJ010810 | cytochrome P450 4F14 | RSM |  |  | -1.03 |
| CPIJ013684 | Mitochondrial glutamate carrier | RSM |  |  | 0.807 |
| CPIJ019765 | cytochrome P450 9c1 | RSM |  |  | 0.863 |
| CPIJ003396 | 2,5-diketo-D-gluconic acid reductase B | RSM |  |  | -0.773 |
| CPIJ008492 | 39S ribosomal protein L40, mitochondrial | RSM |  |  | -0.888 |
| CPIJ010226 | cytochrome P450 CYP12A2 | RSM |  |  | 0.995 |
| CPIJ011502 | short-chain dehydrogenase | RSM |  |  | -0.955 |
| CPIJ017991 | peroxisomal n1-acetyl-spermine/spermidine oxidase | RSM |  |  | -0.835 |
| CPIJ017243 | cytochrome P450 4c21 | RSM | 2.199 | 1.68 |  |
| CPIJ002073 | juvenile hormone esterase | RSM | -0.971 | -1.56 |  |
| CPIJ002785 | carboxylesterase | RSM | -1.397 | -2.016 |  |
| CPIJ012011 | conserved hypothetical protein | RSM | -0.78 | -1.385 |  |
| CPIJ000655 | cytochrome P450 9b2 | RSM | -1.664 | -1.7 |  |
| CPIJ000729 | mitochondrial ribosomal protein, L17 | RSM | -1.225 | -1.135 |  |
| CPIJ002384 | endoplasmin | RSM | -1.469 | -2.013 |  |
| CPIJ002591 | mitochondrial solute carrier | RSM | -1.094 | -1.276 |  |
| CPIJ004795 | mitochondrial carrier protein | RSM | 0.765 | 0.779 |  |
| CPIJ005508 | DNA-J/hsp40 | RSM | -1.402 | -1.921 |  |
| CPIJ005552 | thioredoxin reductase 1, mitochondrial | RSM | -1.149 | -1.089 |  |
| CPIJ005656 | oxidoreductase | RSM | -1.9 | -2.374 |  |
| CPIJ007228 | heat shock protein | RSM | -2.763 | -2.01 |  |
| CPIJ007314 | mitochondrial 18 kDa protein | RSM | -1.442 | -2.011 |  |
| CPIJ009912 | mitochondrial ribosomal protein S34 | RSM | -1.298 | -1.397 |  |
| CPIJ010382 | mitochondrial import inner membrane translocase subunit Tim10 | RSM | -1.308 | -1.893 |  |
| CPIJ011366 | mitochondrial ribosomal protein S31 | RSM | -1.28 | -1.37 |  |
| CPIJ013823 | mitochondrial import inner membrane translocase subunit Tim22 | RSM | -1.245 | -1.49 |  |
| CPIJ017625 | conserved hypothetical protein | RSM | -0.989 | -1.077 |  |
| CPIJ018053 | mitochondrial inner membrane protein translocase, 8kD-subunit | RSM | -1.399 | -1.862 |  |
| CPIJ018054 | mitochondrial import inner membrane translocase subunit Tim8 A | RSM | -1.473 | -1.833 |  |
| CPIJ018211 | mitochondrial inner membrane protein translocase, 13kD-subunit | RSM | -1.176 | -1.571 |  |
| CPIJ018255 | mitochondrial 28S ribosomal protein S33 | RSM | -1.709 | -2.047 |  |
| CPIJ018406 | cytochrome B561 | RSM | -1.496 | -1.351 |  |
| CPIJ020006 | zinc finger protein 569 | RSM | 1.177 | 0.97 |  |
| CPIJ001392 | conserved hypothetical protein | RSM | -0.953 | -1.092 |  |
| CPIJ001842 | translation initiation factor IF-2, mitochondrial | RSM | -0.869 | -1.112 |  |
| CPIJ002377 | cytochrome c-type heme lyase | RSM | -0.811 | -0.88 |  |
| CPIJ002387 | RPII140-upstream gene protein | RSM | -0.967 | -1.045 |  |
| CPIJ003110 | 39S ribosomal protein L10, mitochondrial | RSM | -0.84 | -1.005 |  |
| CPIJ004545 | ribosome recycling factor, mitochondrial | RSM | -0.879 | -1.339 |  |
| CPIJ007862 | mitochondrial ribosomal protein L50 | RSM | -0.843 | -1.413 |  |
| CPIJ011273 | 39S ribosomal protein 54, mitochondrial | RSM | -0.872 | -1.01 |  |
| CPIJ011578 | mitochondrial ribosomal protein L30 | RSM | -0.879 | -1.336 |  |
| CPIJ016252 | cytochrome c oxidase assembly protein cox15 | RSM | -0.952 | -1.547 |  |
| CPIJ018626 | glutathione-s-transferase theta, gst | RSM | -1.051 | -1.357 |  |
| CPIJ019088 | NFAT | RSM | 1.204 | 1.128 |  |
| CPIJ019838 | mitochondrial oxodicarboxylate carrier | RSM | -0.838 | -1.197 |  |
| CPIJ007420 | cytochrome B561 | RSM | -1.543 | -1.177 |  |
| CPIJ008935 | 39S ribosomal protein L17, mitochondrial | RSM | -0.866 | -0.918 |  |
| CPIJ012685 | cytochrome | RSM | 1.387 | 0.79 |  |
| CPIJ002542 | mitochondrial import receptor subunit tom40 | RSM | -0.763 | -1.091 |  |
| CPIJ004601 | 3-oxoacyl-[acyl-carrier-protein] reductase 1 | RSM | -1.057 | -1.029 |  |
| CPIJ006417 | insect replication protein a | RSM | -0.811 | -0.877 |  |
| CPIJ010540 | cytochrome P450 9b2 | RSM | -1.021 | -1.551 |  |
| CPIJ012840 | 39S ribosomal protein L24, mitochondrial | RSM | -0.776 | -1.153 |  |
| CPIJ016856 | cytochrome P450 | RSM | -1.064 | -0.965 |  |
| CPIJ002063 | mitochondrial ribosomal protein L42 | RSM |  | -1.013 |  |
| CPIJ006475 | mitochondrial 2-oxoglutarate/malate carrier protein | RSM |  | -0.946 |  |
| CPIJ006691 | insect replication protein a | RSM |  | -0.979 |  |
| CPIJ008368 | 39S ribosomal protein L44 | RSM |  | -1.142 |  |
| CPIJ009434 | glutathione S-transferase 1 | RSM |  | -1.134 |  |
| CPIJ010328 | 39S ribosomal protein L51, mitochondrial | RSM |  | -0.992 |  |
| CPIJ011521 | mitochondrial 28S ribosomal protein S29 | RSM |  | -1.008 |  |
| CPIJ012682 | mitochondrial dicarboxylate carrier | RSM |  | -1.276 |  |
| CPIJ016740 | conserved hypothetical protein | RSM |  | -0.909 |  |
| CPIJ017747 | cytochrome c oxidase assembly protein COX15 | RSM |  | -0.966 |  |
| CPIJ018687 | mitochondrial import inner membrane translocase subunit Tim9 | RSM |  | -1.106 |  |
| CPIJ000344 | 39S ribosomal protein L24, mitochondrial | RSM |  | -1.131 |  |
| CPIJ001620 | 50S ribosomal protein L1 | RSM |  | -0.777 |  |
| CPIJ002680 | glutathione S-transferase | RSM |  | -0.978 |  |
| CPIJ002682 | GSTD2 protein | RSM |  | 1.314 |  |
| CPIJ003877 | NADH-ubiquinone oxidoreductase subunit B14.5b | RSM |  | -0.934 |  |
| CPIJ007771 | mitochondrial ribosomal protein S26 | RSM |  | -0.875 |  |
| CPIJ007827 | carboxylesterase 2 | RSM |  | -0.809 |  |
| CPIJ008457 | 39S ribosomal protein L55, mitochondrial | RSM |  | -0.812 |  |
| CPIJ008826 | bifunctional coenzyme A synthase | RSM |  | -1.116 |  |
| CPIJ009477 | cytochrome P450 4d10 | RSM |  | -0.792 |  |
| CPIJ010573 | 28S ribosomal protein S9, mitochondrial | RSM |  | -1.039 |  |
| CPIJ011026 | dehydrogenase/reductase SDR family member 7 | RSM |  | -1.129 |  |
| CPIJ013282 | 39S ribosomal protein L9, mitochondrial | RSM |  | -0.882 |  |
| CPIJ013741 | mitochondrial 39S ribosomal protein L27 | RSM |  | -0.815 |  |
| CPIJ015929 | mitochondrial ribosomal protein S18A | RSM |  | -0.84 |  |
| CPIJ016780 | mitochondrial 2-oxoglutarate/malate carrier protein | RSM |  | -0.872 |  |
| CPIJ016888 | aldehyde oxidase 2 | RSM |  | 0.822 |  |
| CPIJ017407 | mitochondrial 39S ribosomal protein L3 | RSM |  | -0.759 |  |
| CPIJ017732 | NADH dehydrogenase 1 alpha subcomplex subunit 12 | RSM |  | -0.918 |  |
| CPIJ019136 | 39S ribosomal protein L35, mitochondrial | RSM |  | -0.841 |  |
| CPIJ019848 | mitochondrial ribosomal protein, S26 | RSM |  | -1.034 |  |
| CPIJ017242 | cytochrome P450 | RSM | 0.907 |  |  |
| CPIJ002663 | glutathione S-transferase 1-1 | RSM | 1.176 |  |  |
| CPIJ006139 | mitochondrial ribosomal protein, L53 | RSM | -0.848 |  |  |
| CPIJ010982 | heat shock factor binding protein | RSM | -0.969 |  |  |
| CPIJ016846 | cytochrome P450 6a8 | RSM | -0.898 |  |  |
| CPIJ010413 | mitochondrial import inner membrane translocase subunit Tim23 | RSM | -0.837 |  |  |
| CPIJ007135 | juvenile hormone esterase | RSM | -0.783 |  |  |
| CPIJ007923 | conserved hypothetical protein | RSM | -0.774 |  |  |
| CPIJ009569 | cytochrome P450 4V2 | RSM | 1.207 |  |  |
| CPIJ011501 | short-chain dehydrogenase | RSM | -0.759 |  |  |
| CPIJ008665 | mitochondrial ATP synthase coupling factor 6 | RSM, TRP |  |  | -0.872 |
| CPIJ000673 | glutamate transporter | TRP | 3.282 | 3.15 | 2.077 |
| CPIJ002361 | sodium/solute symporter | TRP | 4.597 | 4.905 | 1.271 |
| CPIJ012067 | conserved hypothetical protein | TRP | 2.486 | 3.862 | 1.053 |
| CPIJ009335 | ATPase n2b | TRP | 0.95 | 1.869 | 2.286 |
| CPIJ012066 | sodium/Chloride dependent amino acid transporter | TRP | 2.139 | 3.213 | 1.178 |
| CPIJ007432 | sialin | TRP |  | 0.928 | 0.939 |
| CPIJ013203 | soluble NSF attachment protein | TRP |  | 0.775 | 1.729 |
| CPIJ009137 | transmembrane and coiled-coil domains protein 1 | TRP |  | 0.783 | 1.877 |
| CPIJ012296 | glucose dehydrogenase | TRP | 0.9 |  | 1.479 |
| CPIJ002659 | transmembrane protease | TRP | 1.484 | 0.955 | 1.402 |
| CPIJ014225 | CRAL/TRIO domain-containing protein | TRP | 1.29 |  | 1.142 |
| CPIJ005816 | CRAL/TRIO domain-containing protein | TRP | 0.968 | 1.413 | 0.976 |
| CPIJ015359 | glucose transport protein | TRP | 1.94 | 2.285 | 1.204 |
| CPIJ004517 | sugar transporter | TRP |  |  | 1.273 |
| CPIJ013090 | sialin | TRP |  | 1.716 | 1.354 |
| CPIJ000674 | excitatory amino acid transporter 3 | TRP | 1.616 | 1.735 | 1.424 |
| CPIJ018475 | synaptobrevin | TRP |  | 1.123 | 1.244 |
| CPIJ004516 | sugar transporter | TRP |  | 0.974 | 1.433 |
| CPIJ001600 | cytohesin 1, 2, 3, 4 | TRP |  | 1.25 | 1.367 |
| CPIJ012065 | tryptophan transporter | TRP | 3.414 | 3.134 |  |
| CPIJ008618 | chloride channel protein 7 | TRP |  | 1.075 | 1.16 |
| CPIJ013674 | conserved hypothetical protein | TRP |  | 0.822 | 0.811 |
| CPIJ008456 | cystinosin | TRP | 1.118 | 1.775 | 1.185 |
| CPIJ010069 | ATP-binding cassette sub-family A member 3 | TRP |  | 1.099 | 1.186 |
| CPIJ003950 | conserved hypothetical protein | TRP |  | 0.935 | 1.161 |
| CPIJ011963 | multidrug resistance-associated protein 1 | TRP | -1.44 |  | 0.832 |
| CPIJ009442 | prenylated Rab acceptor protein 1 | TRP |  |  | 1.132 |
| CPIJ001942 | sodium-dependent phosphate transporter | TRP |  | 1.301 | 1.272 |
| CPIJ002096 | importin-7 | TRP |  |  | 0.807 |
| CPIJ012563 | transmembrane protein 77 | TRP | 0.97 | 1.412 | 1.112 |
| CPIJ007889 | abc transporter | TRP |  |  | 0.976 |
| CPIJ003323 | synaptosomal associated protein | TRP |  |  | 0.977 |
| CPIJ005854 | abc transporter | TRP |  |  | 0.752 |
| CPIJ020068 | peptidyl-prolyl cis-trans isomerase cyp8 | TRP, DIV |  |  | 0.917 |
| CPIJ002443 | sodium-dependent phosphate transporter | TRP | 1.508 | 1.325 | 0.764 |
| CPIJ017814 | transport protein SEC22 | TRP |  | 0.848 | 0.978 |
| CPIJ014882 | clathrin heavy chain | TRP |  | 0.949 | 0.972 |
| CPIJ015036 | syntaxin | TRP | 0.972 | 1.243 | 1.178 |
| CPIJ015651 | cation-transporting ATPase | TRP |  |  | 0.851 |
| CPIJ007243 | conserved hypothetical protein | TRP, DIV | 1.862 | 1.327 | 1.172 |
| CPIJ001599 | cytohesin 1, 2, 3, 4 | TRP |  | 0.92 | 1.026 |
| CPIJ003611 | sugar transporter | TRP |  | 1.358 | 1.46 |
| CPIJ004492 | sodium/potassium/calcium exchanger 3 | TRP | 3.66 | 3.192 | 1.963 |
| CPIJ010164 | katanin p60 ATPase-containing subunit | TRP | 0.866 | 1.003 | 0.81 |
| CPIJ004221 | monocarboxylate transporter | TRP | 0.961 | 1.364 | 1.087 |
| CPIJ006785 | pickpocket | TRP | 1.416 | 1.95 | 0.942 |
| CPIJ005368 | lysosomal trafficking regulator | TRP | 1.763 | 1.387 | 1.187 |
| CPIJ006913 | tumor susceptibility gene 101 protein | TRP |  | 0.868 | 0.789 |
| CPIJ019820 | sugar transporter | TRP | 1.175 | 1.366 | 1.148 |
| CPIJ002543 | phosphate transporter | TRP |  | 1.437 | 0.82 |
| CPIJ005331 | sulfate transporter | TRP |  |  | 0.794 |
| CPIJ001310 | canalicular multispecific organic anion transporter 2 | TRP | -1.186 |  |  |
| CPIJ001623 | sorting nexin | TRP |  | 1.108 |  |
| CPIJ010329 | importin beta-3 | TRP |  | 1.006 |  |
| CPIJ016577 | cation chloride cotransporter | TRP | 0.884 | 0.778 |  |
| CPIJ000905 | tetraspanin | TRP | 0.752 |  |  |
| CPIJ020291 | sodium-dependent multivitamin transporter | TRP | -1.555 | -1.318 | -1.084 |
| CPIJ003897 | transmembrane protein | TRP |  | -1.134 | -0.921 |
| CPIJ012147 | sulfate transporter | TRP | 1.706 |  |  |
| CPIJ001520 | multidrug resistance-associated protein 1 | TRP | -0.896 |  | -0.998 |
| CPIJ000967 | voltage-dependent anion-selective channel | TRP |  | -1.016 | -0.833 |
| CPIJ004646 | UDP-galactose transporter | TRP | -0.956 | -1.366 | -0.833 |
| CPIJ009357 | alkali metal ion/proton exchanger 3 | TRP | 1.184 | 1.216 |  |
| CPIJ014223 | cellular retinaldehyde-binding protein | TRP, DIV |  | -0.899 | -0.762 |
| CPIJ001521 | multidrug resistance-associated protein 1 | TRP |  |  | -0.881 |
| CPIJ012779 | potassium/chloride symporter | TRP |  | -0.918 | -0.934 |
| CPIJ002534 | sodium/iodide cotransporter | TRP | -0.861 | -1.124 |  |
| CPIJ015251 | solute carrier family 2 | TRP |  | -0.872 | -1.322 |
| CPIJ009770 | conserved hypothetical protein | TRP | 1.368 |  |  |
| CPIJ020148 | monocarboxylate transporter | TRP |  |  | -0.902 |
| CPIJ019020 | creatine transporter | TRP |  | -1.083 |  |
| CPIJ008812 | sodium-dependent phosphate transporter | TRP |  | -0.786 | -0.958 |
| CPIJ013697 | tricarboxylate transport protein, mitochondrial | TRP | -1.472 | -1.835 | -1.225 |
| CPIJ001812 | sugar transporter | TRP | -1.482 | -1.234 | -0.851 |
| CPIJ007376 | calreticulin | TRP | -2.35 | -2.788 | -1.136 |
| CPIJ014443 | ATP-binding cassette sub-family G member 4 | TRP | -1.174 | -1.52 | -0.985 |
| CPIJ011516 | surfeit locus protein 4 | TRP |  |  | -1.087 |
| CPIJ017740 | potassium/chloride symporter | TRP |  | -0.86 | -0.883 |
| CPIJ000602 | sulfate transporter | TRP |  | -1.331 | -1.013 |
| CPIJ016918 | canalicular multispecific organic anion transporter 1 | TRP | -1.082 |  | -1.223 |
| CPIJ014208 | monocarboxylate transporter | TRP |  |  | -0.909 |
| CPIJ017440 | mfs transporter | TRP |  |  | -0.983 |
| CPIJ007436 | Sialin, Sodium/sialic acid cotransporter | TRP |  | -1.354 | -0.751 |
| CPIJ017743 | tetraspanin | TRP |  |  | -0.985 |
| CPIJ000613 | sulfate transporter | TRP |  |  | -1.228 |
| CPIJ014936 | cationic amino acid transporter | TRP |  | -0.98 | -1.146 |
| CPIJ008945 | sugar transporter | TRP | 1.066 |  | -1.005 |
| CPIJ004604 | multidrug resistance-associated protein 1 | TRP | -1.116 |  | -1.29 |
| CPIJ001134 | excitatory amino acid transporter 3 | TRP |  | -0.968 | -1.153 |
| CPIJ001523 | TRPgamma cation channel | TRP |  |  | -0.871 |
| CPIJ011438 | amino acid transporter | TRP |  |  | -0.972 |
| CPIJ007428 | Sialin, Sodium/sialic acid cotransporter | TRP | 1.128 |  | -1.085 |
| CPIJ009956 | d-amino acid oxidase | TRP |  | -1.187 |  |
| CPIJ014550 | long form D7Bclu1 salivary protein | TRP |  |  | -0.752 |
| CPIJ008284 | canalicular multispecific organic anion transporter 1 | TRP |  | -0.753 | -1.222 |
| CPIJ012543 | pickpocket | TRP |  |  | -1.012 |
| CPIJ017095 | sulfate transporter | TRP |  | -1.372 | -0.817 |
| CPIJ018186 | phosphatidylinositol transfer protein SEC14 | TRP, DIV |  |  | -1.327 |
| CPIJ008651 | solute carrier family 41 | TRP |  | -1.336 | -1.309 |
| CPIJ005130 | pickpocket | TRP |  |  | -0.828 |
| CPIJ012368 | lipoprotein-releasing system ATP-binding protein lolD | TRP |  | -1.295 | -1.067 |
| CPIJ019232 | acetyl-CoA acetyltransferase, mitochondrial | TRP, DIV |  |  | -1.318 |
| CPIJ015850 | d-amino acid oxidase | TRP | -0.876 | -1.437 | -0.825 |
| CPIJ004760 | low-Mr GTP-binding protein Rab31 | TRP | -1.407 | -1.705 | -1.283 |
| CPIJ017140 | inwardly rectifying k+ channel | TRP | 2.024 |  | -1.069 |
| CPIJ003880 | chloride channel protein 2 | TRP | -0.751 | -0.902 | -0.843 |
| CPIJ005183 | organic anion transporter | TRP | 0.917 | 0.78 |  |
| CPIJ004817 | sodium-dependent phosphate transporter | TRP | -1.449 | -1.866 | -1.68 |
| CPIJ013992 | amino acid transporter | TRP |  | -1.661 | -1.513 |
| CPIJ013993 | amino acid transporter | TRP | -2.457 | -1.857 | -1.291 |
| CPIJ012677 | sugar transporter | TRP |  |  | -1.074 |
| CPIJ000191 | sugar transporter | TRP | -0.8 | -1.147 | -1.228 |
| CPIJ016887 | multidrug resistance protein 2 | TRP | -2.016 | -1.991 | -1.552 |
| CPIJ009225 | aquaporin | TRP |  | -1.554 | -1.784 |
| CPIJ002532 | sodium-dependent multivitamin transporter | TRP |  | -1.147 | -1.003 |
| CPIJ017146 | 2-acylglycerol O-acyltransferase 2-A | TRP | -0.77 | -1.494 | -1.516 |
| CPIJ010750 | tetraspanin | TRP, DIV |  | -1.364 | -1.342 |
| CPIJ012207 | ammonium transporter 1 | TRP |  | -1.572 | -1.484 |
| CPIJ008943 | sugar transporter | TRP |  | -1.947 | -1.528 |
| CPIJ011039 | amino acid transporter | TRP |  | -1.237 | -1.348 |
| CPIJ009224 | nodulin-26 | TRP | 0.834 | -1.408 | -2.027 |
| CPIJ008941 | sugar transporter | TRP |  | -1.609 | -1.717 |
| CPIJ012675 | sugar transporter | TRP | -1.287 | -1.92 | -1.818 |
| CPIJ007435 | conserved hypothetical protein | TRP |  | -0.814 | -1.811 |
| CPIJ007434 | sodium/phosphate cotransporter | TRP |  |  | -1.92 |
| CPIJ010101 | cation efflux protein/ zinc transporter | TRP | -1.982 | -2.557 | -2.307 |
| CPIJ002533 | sodium/solute symporter | TRP |  |  | -1.052 |
| CPIJ010134 | sodium-dependent phosphate transporter | TRP | -0.88 | -2.446 | -1.649 |
| CPIJ015637 | antioxidant enzyme | TRP | -1.203 | -2.257 | -1.909 |
| CPIJ010409 | fructose-1,6-bisphosphatase 1 | TRP | -1.068 | -2.004 | -2.072 |
| CPIJ017878 | permease | TRP | -1.292 | -1.664 | -1.4 |
| CPIJ019446 | copper transport protein | TRP | -1.178 | -2.286 | -1.933 |
| CPIJ005186 | organic anion transporter | TRP | -1.78 | -1.268 | -1.282 |
| CPIJ001859 | sodium/solute symporter | TRP |  | -1.862 | -1.912 |
| CPIJ007620 | choline dehydrogenase | TRP |  | -1.743 | -2.106 |
| CPIJ012068 | sucrose transport protein | TRP | -1.29 | -1.363 | -2.382 |
| CPIJ004489 | potassium-dependent sodium-calcium exchanger | TRP |  | -1.62 | -2.104 |
| CPIJ000961 | G protein-activated inward rectifier potassium channel 1 | TRP |  | -1.547 | -1.584 |
| CPIJ003167 | zinc/iron transporter | TRP | -0.988 | -2.056 | -2.472 |
| CPIJ004491 | sodium/potassium/calcium exchanger 3 | TRP |  | -1.081 | -2.549 |
| CPIJ007621 | choline dehydrogenase | TRP | -1.085 | -2.825 | -2.64 |
| CPIJ014926 | conserved hypothetical protein | TRP | -1.992 | -2.395 | -2.685 |
| CPIJ000242 | monocarboxylate transporter | TRP | -1.358 | -2.986 | -3.554 |
| CPIJ007622 | glucose dehydrogenase | TRP |  | -2.246 | -3.159 |
| CPIJ019526 | conserved hypothetical protein | TRP | 1.331 | 0.863 | 1.15 |
| CPIJ008928 | cationic amino acid transporter | TRP | -1.193 | -1.191 | -0.753 |
| CPIJ019349 | malonyl CoA-acyl carrier protein transacylase | TRP | -0.901 | -1.603 | -0.864 |
| CPIJ007997 | sodium-dependent serotonin transporter | TRP | 0.891 | 1.22 | 0.77 |
| CPIJ010187 | sodium/solute symporter | TRP | -1.391 | -0.874 | -0.971 |
| CPIJ011949 | potassium-dependent sodium-calcium exchanger | TRP | 1.272 | 0.812 | 0.778 |
| CPIJ003267 | sodium/Chloride dependent amino acid transporter | TRP |  | 0.775 | 1.006 |
| CPIJ003274 | vacuolar proton translocating ATPase 116 kDa subunit a 1 | TRP |  | -0.983 | -0.797 |
| CPIJ001861 | sodium/solute symporter | TRP |  | 0.838 | 0.771 |
| CPIJ007069 | SNARE Vti1a protein | TRP |  | 0.933 | 0.759 |
| CPIJ008274 | monocarboxylate transporter 3 | TRP |  | 1.079 | 1.088 |
| CPIJ015151 | adenylate cyclase | TRP | 1.522 |  | 1.036 |
| CPIJ002058 | conserved hypothetical protein | TRP | -1.126 |  | 0.886 |
| CPIJ000611 | sulfate transporter 1.2 | TRP |  |  | -1.123 |
| CPIJ008117 | monocarboxylate transporter | TRP |  |  | -0.945 |
| CPIJ003418 | V-type ATP synthase beta chain | TRP |  |  | -0.754 |
| CPIJ006275 | organic anion transporter | TRP |  |  | 0.791 |
| CPIJ007115 | sodium/potassium-dependent ATPase beta-2 subunit | TRP |  |  | 1.233 |
| CPIJ007772 | ATP synthase alpha subunit vacuolar | TRP |  |  | -0.77 |
| CPIJ008118 | monocarboxylate transporter | TRP |  |  | -0.879 |
| CPIJ008774 | transmembrane protein 135 | TRP |  |  | -0.911 |
| CPIJ014699 | vacuolar ATP synthase subunit B | TRP |  |  | -0.762 |
| CPIJ016432 | vacuolar ATP synthase subunit F | TRP |  |  | -0.751 |
| CPIJ018600 | conserved hypothetical protein | TRP |  |  | 0.817 |
| CPIJ018677 | trafficking protein particle complex subunit 2 | TRP |  |  | -0.773 |
| CPIJ000151 | sodium/solute symporter | TRP | -1.34 | -1.167 |  |
| CPIJ002353 | cystinosin | TRP | -1.496 | -1.336 |  |
| CPIJ002838 | THO complex 7 | TRP | -1.044 | -1.067 |  |
| CPIJ004839 | transmembrane and coiled-coil domain-containing protein 1 | TRP | -1.125 | -0.979 |  |
| CPIJ005257 | transmembrane protein 85 | TRP | -0.979 | -0.926 |  |
| CPIJ006367 | transmembrane protein 93 | TRP | -0.931 | -0.925 |  |
| CPIJ007043 | transmembrane protein 70 | TRP | -1.241 | -1.316 |  |
| CPIJ007054 | alpha-tocopherol transfer protein | TRP | 1.203 | 0.8 |  |
| CPIJ007723 | transport protein Sec61 subunit alpha 2 | TRP | -0.818 | -1.253 |  |
| CPIJ009025 | hypothetical protein | TRP | -1.488 | -1.656 |  |
| CPIJ010068 | ATP-binding cassette sub-family A member 7 | TRP | 1.992 | 1.564 |  |
| CPIJ010921 | monocarboxylate transporter | TRP | -1.25 | -1.088 |  |
| CPIJ018212 | abc transporter | TRP | 1.113 | 1.037 |  |
| CPIJ005682 | translocon-associated protein subunit beta | TRP | -0.765 | -0.966 |  |
| CPIJ006132 | importin alpha | TRP | -0.871 | -1.223 |  |
| CPIJ014934 | malonyl CoA-acyl carrier protein transacylase | TRP | -0.919 | -1.591 |  |
| CPIJ007395 | conserved hypothetical protein | TRP | 0.79 | 0.777 |  |
| CPIJ000931 | conserved hypothetical protein | TRP | -0.797 | -1.085 |  |
| CPIJ004980 | ATP-binding cassette sub-family A member 3 | TRP | 1.466 | 1.207 |  |
| CPIJ005830 | glutaredoxin | TRP | -0.798 | -0.96 |  |
| CPIJ006002 | high affinity copper transporter | TRP | -0.854 | -0.762 |  |
| CPIJ006230 | phospholipid-transporting ATPase 1 | TRP | 0.806 | 0.964 |  |
| CPIJ006431 | cation-transporting ATPase | TRP | 0.91 | 0.99 |  |
| CPIJ009698 | conserved hypothetical protein | TRP | 0.787 | 1.267 |  |
| CPIJ010587 | WD40-repeat protein | TRP | -0.94 | -0.958 |  |
| CPIJ011439 | proton-coupled amino acid transporter 1 | TRP | 0.753 | 1.022 |  |
| CPIJ002868 | ATP-binding cassette sub-family B member 10, mitochondrial | TRP |  | -0.935 |  |
| CPIJ003005 | coatomer subunit zeta-1 | TRP |  | -0.973 |  |
| CPIJ006257 | solute carrier family 35 member B1 | TRP |  | -0.784 |  |
| CPIJ008001 | transmembrane protein 93 | TRP |  | -0.82 |  |
| CPIJ012070 | sucrose transport protein | TRP |  | -0.752 |  |
| CPIJ012523 | coatomer subunit gamma | TRP |  | -0.751 |  |
| CPIJ000184 | syntaxin | TRP |  | 0.758 |  |
| CPIJ001335 | serine threonine-protein kinase | TRP |  | 0.772 |  |
| CPIJ002067 | vacuolar ATP synthase subunit C | TRP |  | -0.863 |  |
| CPIJ002817 | d-amino acid oxidase | TRP |  | -1.033 |  |
| CPIJ003441 | electron transfer flavoprotein subunit alpha, mitochondrial | TRP |  | -1.118 |  |
| CPIJ003914 | transmembrane protein 177 | TRP |  | -0.752 |  |
| CPIJ007272 | d-amino acid oxidase | TRP |  | -1.162 |  |
| CPIJ007273 | d-amino acid oxidase | TRP |  | -0.907 |  |
| CPIJ008281 | multidrug resistance-associated protein 1 | TRP |  | 0.955 |  |
| CPIJ008283 | multidrug resistance protein 2 | TRP |  | -0.752 |  |
| CPIJ008515 | cellular retinaldehyde binding protein | TRP |  | -0.946 |  |
| CPIJ008942 | sugar transporter | TRP |  | -1.005 |  |
| CPIJ009452 | monocarboxylate transporter | TRP |  | -0.821 |  |
| CPIJ010259 | voltage and ligand gated potassium channel | TRP |  | -0.876 |  |
| CPIJ010473 | transmembrane protein 19 | TRP |  | -0.817 |  |
| CPIJ013204 | peroxisomal biogenesis factor 3 | TRP |  | -0.806 |  |
| CPIJ013655 | transmembrane protein 38A | TRP |  | -0.786 |  |
| CPIJ017442 | Exportin | TRP |  | 0.756 |  |
| CPIJ019347 | copper-transporting ATPase 1 | TRP |  | -0.766 |  |
| CPIJ008946 | sugar transporter | TRP | 1.867 |  |  |
| CPIJ010067 | ATP-binding cassette sub-family A member 3 | TRP | 1.27 |  |  |
| CPIJ000906 | tetraspanin | TRP | 0.821 |  |  |
| CPIJ006091 | zinc/iron transporter | TRP | 1.086 |  |  |
| CPIJ009454 | phosphatidylinositol transfer protein/retinal degeneration b protein | TRP | -0.944 |  |  |
| CPIJ014925 | solute carrier family 2 | TRP | -1.461 |  |  |
| CPIJ005015 | ICLn protein | TRP | -1.06 |  |  |
| CPIJ012737 | nucleoporin 50kDa | TRP | -1.11 |  |  |
| CPIJ002831 | oligopeptide transporter | TRP | -1.334 |  |  |
| CPIJ002845 | monocarboxylate transporter | TRP | -0.94 |  |  |
| CPIJ004244 | cationic amino acid transporter | TRP | 0.876 |  |  |
| CPIJ004333 | multidrug resistance-associated protein | TRP | 0.964 |  |  |
| CPIJ005690 | arsenical pump-driving ATPase | TRP | -0.781 |  |  |
| CPIJ006416 | conserved hypothetical protein | TRP | -0.79 |  |  |
| CPIJ007114 | sodium/potassium-dependent ATPase beta-2 subunit | TRP | -1.08 |  |  |
| CPIJ009504 | clathrin coat assembly protein AP17 | TRP | -0.8 |  |  |
| CPIJ010829 | sodium/nucleoside cotransporter 1 | TRP | -1.155 |  |  |
| CPIJ011041 | amino acid transporter | TRP | 1.105 |  |  |
| CPIJ012120 | NF-X1-type zinc finger protein NFXL1 | TRP | -0.869 |  |  |
| CPIJ012539 | organic cation transporter protein | TRP | -0.83 |  |  |
| CPIJ016917 | transport and Golgi organization | TRP | -0.756 |  |  |
| CPIJ017138 | inwardly rectifying k+ channel | TRP | 1.319 |  |  |
| CPIJ017473 | organic cation transporter | TRP | 0.758 |  |  |
| CPIJ020206 | 2-acylglycerol O-acyltransferase 2-A | TRP | -1.027 |  |  |
| CPIJ014670 | endothelin B receptor | TRP, DIV |  |  | 0.843 |
| CPIJ016900 | ns1 binding protein | TRP, DIV |  | 1.004 |  |
| CPIJ008639 | past-1 | TRP, DIV | -0.798 |  |  |
| CPIJ008664 | conserved hypothetical protein | RTT | -1.152 | 4.149 | 4.396 |
| CPIJ006534 | conserved hypothetical protein | RTT |  | 1.27 | 2.377 |
| CPIJ015813 | conserved hypothetical protein | RTT |  | 1.735 | 1.627 |
| CPIJ018256 | NULL | RTT | 1.077 | 1.607 | 1.712 |
| CPIJ018848 | mitochondrial protein import protein MAS5 | RTT, RSM |  | -0.837 | 1.835 |
| CPIJ009235 | RNA 3'-terminal phosphate cyclase | RTT | -1.055 |  | 1.499 |
| CPIJ009234 | RNA 3'-terminal phosphate cyclase | RTT | -1.12 |  | 1.551 |
| CPIJ003266 | CCAAT/enhancer-binding protein | RTT | 0.944 | 1.57 | 1.614 |
| CPIJ015642 | ATP-dependent RNA helicase Ddx1 | RTT |  |  | 1.551 |
| CPIJ014791 | eukaryotic translation initiation factor 2C 2 | RTT | 1.361 | 1.68 | 1.474 |
| CPIJ002798 | cuticle protein | RTT | -1.101 |  |  |
| CPIJ003181 | RNAse H | RTT | 1.947 | 2.823 | 2.449 |
| CPIJ001988 | ATP-dependent RNA helicase A | RTT |  | 0.976 | 1.383 |
| CPIJ001612 | heterogeneous nuclear ribonucleoprotein | RTT |  |  | 1.002 |
| CPIJ039626 | U4 spliceosomal RNA [Source: RFAM 8.0] | RTT |  | 1.515 | 1.17 |
| CPIJ012801 | conserved hypothetical protein | RTT | 1.292 | 1.953 | 1.306 |
| CPIJ039616 | U4 spliceosomal RNA [Source: RFAM 8.0] | RTT |  | 1.399 | 1.053 |
| CPIJ010951 | RNA polymerase I-specific transcription initiation factor RRN3 | RTT |  | 0.817 |  |
| CPIJ014935 | DEAD box ATP-dependent RNA helicase | RTT |  | 0.847 | 0.765 |
| CPIJ017391 | ATP-dependent RNA helicase DHX8 | RTT |  |  | 1.014 |
| CPIJ039789 | U4 spliceosomal RNA [Source: RFAM 8.0] | RTT |  | 1.591 | 0.908 |
| CPIJ006749 | translation initiation factor 5C | RTT |  |  | 1.056 |
| CPIJ005884 | eukaryotic translation initiation factor 2-alpha kinase 1 | RTT | 1.194 | 1.597 | 1.119 |
| CPIJ005588 | small nuclear ribonucleoprotein SM D3 | RTT |  |  | 0.887 |
| CPIJ014361 | conserved hypothetical protein | RTT |  |  | 0.869 |
| CPIJ013324 | polyubiquitin | RTT |  |  | 1.098 |
| CPIJ012510 | ATP-dependent RNA helicase DDX24 | RTT | -0.793 |  |  |
| CPIJ012377 | histone H1 | RTT | 1.176 | 1.53 |  |
| CPIJ011705 | myelin expression factor 2 | RTT |  |  | 0.839 |
| CPIJ010599 | exonuclease nef-sp | RTT, DIV |  | 0.951 | 0.848 |
| CPIJ009446 | DEAD box ATP-dependent RNA helicase | RTT | 0.931 | 1.605 | 1.131 |
| CPIJ039777 | U5 spliceosomal RNA [Source: RFAM 8.0] | RTT |  | 0.795 |  |
| CPIJ021729 | Arg tRNA | RTT | -1.096 |  |  |
| CPIJ014048 | artemis protein | RTT |  |  | 0.807 |
| CPIJ005868 | multisynthetase complex auxiliary component p43 | RTT |  | -0.849 |  |
| CPIJ011778 | transcription initiation factor TFIID subunit 12 | RTT |  |  | 0.836 |
| CPIJ011259 | eukaryotic translation initiation factor | RTT | -0.86 |  |  |
| CPIJ003270 | broad-complex core-protein | RTT |  |  | -0.781 |
| CPIJ004247 | transcriptional activator protein Pur-alpha | RTT | -0.815 | -0.963 | -0.79 |
| CPIJ003382 | SERAC1 | RTT | -1.424 | -0.753 |  |
| CPIJ015469 | translation initiation factor eif-2b | RTT |  | -0.758 | -0.852 |
| CPIJ002050 | homeobox protein | RTT | -1.085 | -1.492 | -1.218 |
| CPIJ015889 | homeobox protein | RTT, DIV |  | -1.397 | -1.504 |
| CPIJ009710 | conserved hypothetical protein | RTT |  |  | -1.581 |
| CPIJ014845 | 40S ribosomal protein S20 | RTT |  |  | -1.598 |
| CPIJ008661 | conserved hypothetical protein | RTT |  |  | -2.116 |
| CPIJ003074 | conserved hypothetical protein | RTT | 0.829 | 0.845 | 0.774 |
| CPIJ012380 | histone H2A type 1-C | RTT | -0.774 | -1.141 | -1.219 |
| CPIJ007629 | splicing factor | RTT | -1.161 | -0.867 | -0.809 |
| CPIJ001440 | eukaryotic translation initiation factor 3 subunit | RTT | -1.008 | -1.183 | -0.798 |
| CPIJ010112 | 60S ribosomal protein L5 | RTT |  | 0.939 | 0.815 |
| CPIJ012390 | late histone H2A.3 | RTT |  | -0.999 | -1.245 |
| CPIJ017451 | SOF1 | RTT |  | 1.019 | 0.782 |
| CPIJ011083 | heat shock protein 70 B2 | RTT |  | 2.758 | 2.8 |
| CPIJ005825 | conserved hypothetical protein | RTT |  | 0.937 | 0.787 |
| CPIJ019323 | histone-lysine n-methyltransferase | RTT |  | 1.301 | 0.766 |
| CPIJ001008 | homeotic antennapedia protein | RTT |  | -0.771 | -0.761 |
| CPIJ002918 | eukaryotic translation initiation factor 4 gamma | RTT |  | 1.157 | 0.847 |
| CPIJ011709 | conserved hypothetical protein | RTT | 0.861 |  | 0.918 |
| CPIJ019325 | conserved hypothetical protein | RTT |  |  | 0.753 |
| CPIJ039609 | U2 spliceosomal RNA [Source: RFAM 8.0] | RTT |  |  | -0.934 |
| CPIJ002728 | splicing factor 3A subunit 3 | RTT |  |  | 0.836 |
| CPIJ003592 | DNA-repair protein complementing XP-C cells | RTT |  |  | 0.832 |
| CPIJ021707 | Thr tRNA | RTT |  |  | -0.999 |
| CPIJ027176 | Thr tRNA | RTT |  |  | -1.007 |
| CPIJ027886 | Thr tRNA | RTT |  |  | -0.96 |
| CPIJ035058 | Thr tRNA | RTT |  |  | -0.975 |
| CPIJ037678 | Thr tRNA | RTT |  |  | -1.025 |
| CPIJ039590 | U2 spliceosomal RNA [Source: RFAM 8.0] | RTT |  |  | -0.912 |
| CPIJ039665 | Eukaryotic type signal recognition particle RNA [Source: RFAM 8.0] | RTT |  |  | -0.98 |
| CPIJ039758 | U2 spliceosomal RNA [Source: RFAM 8.0] | RTT |  |  | -0.899 |
| CPIJ039791 | Eukaryotic type signal recognition particle RNA [Source: RFAM 8.0] | RTT |  |  | -1.024 |
| CPIJ039796 | Eukaryotic type signal recognition particle RNA [Source: RFAM 8.0] | RTT |  |  | -0.998 |
| CPIJ008189 | ATP-dependent RNA helicase DBP9 | RTT | 0.979 | 0.963 |  |
| CPIJ000325 | DNA primase small subunit | RTT | -1.244 | -1.16 |  |
| CPIJ002279 | elongation factor Tu, mitochondrial | RTT | -1.278 | -1.403 |  |
| CPIJ004698 | elongation factor ts | RTT | -0.8 | -1.086 |  |
| CPIJ004845 | 40S ribosomal protein S3a | RTT | 1.201 | 0.995 |  |
| CPIJ004899 | eukaryotic translation initiation factor 3 subunit 1 | RTT | -1.201 | -0.902 |  |
| CPIJ005091 | DNA replication licensing factor MCM8 | RTT | 1.052 | 0.898 |  |
| CPIJ005421 | signal recognition particle receptor subunit alpha | RTT | -0.944 | -1.113 |  |
| CPIJ008397 | signal recognition particle 68 kDa protein | RTT | -0.865 | -0.946 |  |
| CPIJ009047 | DNA replication licensing factor Mcm6 | RTT | -1.731 | -1.306 |  |
| CPIJ009962 | elongation factor Tu | RTT | -0.877 | -1.229 |  |
| CPIJ009979 | eukaryotic translation initiation factor 4 gamma 3 | RTT | -1.138 | -0.979 |  |
| CPIJ010887 | DNA replication licensing factor MCM4 | RTT | -1.896 | -1.377 |  |
| CPIJ010897 | DNA polymerase epsilon subunit 4 | RTT | -1.037 | -0.984 |  |
| CPIJ010917 | ribonuclease H2 subunit A | RTT | -1.218 | -0.909 |  |
| CPIJ012309 | DNA replication licensing factor MCM3 | RTT | -1.607 | -1.108 |  |
| CPIJ012387 | histone H1 | RTT | 0.955 | 1.219 |  |
| CPIJ013501 | Lsm5 protein | RTT | -1.097 | -1.209 |  |
| CPIJ013588 | mitochondrial 28S ribosomal protein S2 | RTT | -1.157 | -1.145 |  |
| CPIJ015538 | endonuclease G, mitochondrial | RTT | -1.584 | -1.637 |  |
| CPIJ000160 | DNA replication licensing factor MCM7 | RTT | -1.012 | -0.879 |  |
| CPIJ001441 | eukaryotic translation initiation factor 3 subunit | RTT | -1.037 | -1.213 |  |
| CPIJ003743 | DEAD-box helicase Dbp80 | RTT | -0.97 | -1.01 |  |
| CPIJ007476 | DNA repair/transcription protein met18/mms19 | RTT | -0.829 | -0.816 |  |
| CPIJ008667 | DNA polymerase epsilon subunit 2 | RTT | -1.307 | -0.922 |  |
| CPIJ010000 | 39S ribosomal protein L20, mitochondrial | RTT | -0.894 | -1.297 |  |
| CPIJ015005 | late histone H2B.L4 | RTT | -0.962 | -0.972 |  |
| CPIJ001219 | DNA-directed RNA polymerase II 19 kDa polypeptide | RTT | -1.244 | -1.011 |  |
| CPIJ005206 | DNA-directed RNA polymeraseI | RTT | -1.01 | -0.864 |  |
| CPIJ016511 | small nuclear ribonucleoprotein Sm D1 | RTT | -1.415 | -0.801 |  |
| CPIJ001442 | eukaryotic translation initiation factor 3 subunit | RTT | -0.754 | -0.967 |  |
| CPIJ001443 | eukaryotic translation initiation factor 3 subunit | RTT | -1.028 | -1.223 |  |
| CPIJ001445 | eukaryotic translation initiation factor 3 subunit | RTT | -0.939 | -0.787 |  |
| CPIJ001937 | chromodomain helicase DNA binding protein | RTT | -0.809 | -0.921 |  |
| CPIJ003289 | pontin | RTT | -0.973 | -0.777 |  |
| CPIJ008262 | 39S ribosomal protein L15, mitochondrial | RTT | -0.783 | -1.131 |  |
| CPIJ008915 | heat shock 70 kDa protein cognate 4 | RTT | -1.23 | -0.775 |  |
| CPIJ018062 | histone H1 | RTT | -1.159 | -0.842 |  |
| CPIJ019444 | 39S ribosomal protein L21, mitochondrial | RTT | -0.875 | -0.861 |  |
| CPIJ035694 | Ser tRNA | RTT | -0.866 | -0.785 |  |
| CPIJ035696 | Ser tRNA | RTT | -0.858 | -0.803 |  |
| CPIJ007050 | N | RTT |  | 0.815 |  |
| CPIJ010674 | proliferating-cell nucleolar antigen p120 | RTT |  | 0.804 |  |
| CPIJ039761 | 5.8S ribosomal RNA [Source: RFAM 8.0] | RTT |  | 1.069 |  |
| CPIJ001214 | ribosome biogenesis protein RLP24 | RTT |  | 0.933 |  |
| CPIJ002310 | 28S ribosomal protein S15, mitochondrial | RTT |  | -1.112 |  |
| CPIJ003098 | conserved hypothetical protein | RTT |  | 1.173 |  |
| CPIJ007070 | conserved hypothetical protein | RTT |  | 0.913 |  |
| CPIJ008817 | ribosome biogenesis protein RLP24 | RTT |  | 0.849 |  |
| CPIJ039654 | U5 spliceosomal RNA [Source: RFAM 8.0] | RTT |  | 0.955 |  |
| CPIJ039678 | U5 spliceosomal RNA [Source: RFAM 8.0] | RTT |  | 0.956 |  |
| CPIJ039705 | U5 spliceosomal RNA [Source: RFAM 8.0] | RTT |  | 0.773 |  |
| CPIJ039780 | U5 spliceosomal RNA [Source: RFAM 8.0] | RTT |  | 0.889 |  |
| CPIJ000880 | RNA-binding post-transcriptional regulator csx1 | RTT |  | -0.763 |  |
| CPIJ002045 | Poly | RTT |  | 0.834 |  |
| CPIJ002300 | hypothetical protein | RTT |  | 0.796 |  |
| CPIJ002368 | polyA-binding protein interacting protein | RTT |  | 1.155 |  |
| CPIJ002427 | mitochondrial 39S ribosomal protein L4 | RTT |  | -0.758 |  |
| CPIJ003976 | gar2 | RTT |  | -1.001 |  |
| CPIJ004407 | elongation factor 1-alpha | RTT |  | -0.861 |  |
| CPIJ005704 | U11/U12 snRNP 35K | RTT |  | -0.845 |  |
| CPIJ012400 | histone H1 | RTT |  | 0.824 |  |
| CPIJ013504 | DHX9 protein | RTT |  | -0.81 |  |
| CPIJ015119 | mitochondrial 28S ribosomal protein S5 | RTT |  | -0.917 |  |
| CPIJ015639 | 39S ribosomal protein L21, mitochondrial | RTT |  | -0.859 |  |
| CPIJ003555 | nuclear cap-binding protein subunit 2 | RTT | -0.825 |  |  |
| CPIJ005256 | TFIIH basal transcription factor complex p52 subunit | RTT | 0.972 |  |  |
| CPIJ016756 | 60S acidic ribosomal protein P2 | RTT | -1.111 |  |  |
| CPIJ001399 | replication factor C subunit 4 | RTT | -1.504 |  |  |
| CPIJ005330 | 60S ribosome subunit biogenesis protein NIP7 | RTT | -1.671 |  |  |
| CPIJ006616 | small nuclear ribonucleoprotein-associated protein B | RTT | -1.143 |  |  |
| CPIJ007887 | DNA-directed RNA polymerase III 25 kDa polypeptide | RTT | -1.19 |  |  |
| CPIJ007987 | ATP-dependent RNA helicase DRS1 | RTT | -1.137 |  |  |
| CPIJ009694 | tRNA methyltransferase | RTT | -1.126 |  |  |
| CPIJ010410 | tRNA-dihydrouridine synthase | RTT | -1.081 |  |  |
| CPIJ010488 | DEAD box ATP-dependent RNA helicase | RTT | -1.113 |  |  |
| CPIJ015157 | Lsm6 protein | RTT | -0.999 |  |  |
| CPIJ017520 | U3 small nucleolar ribonucleoprotein protein IMP3 | RTT | -1.1 |  |  |
| CPIJ035661 | Arg tRNA | RTT | -1.106 |  |  |
| CPIJ000301 | histone-fold protein CHRAC subunit | RTT | -0.865 |  |  |
| CPIJ000302 | replication factor C subunit 2 | RTT | -1.131 |  |  |
| CPIJ000689 | eukaryotic translation initiation factor 5 | RTT | -1.293 |  |  |
| CPIJ000904 | conserved hypothetical protein | RTT | -0.786 |  |  |
| CPIJ000979 | DNA-directed RNA polymerase I subunit D | RTT | -0.773 |  |  |
| CPIJ002554 | TFIIA | RTT | 0.89 |  |  |
| CPIJ002555 | DNA replication licensing factor Mcm2 | RTT | -1.002 |  |  |
| CPIJ003854 | conserved hypothetical protein | RTT | -0.908 |  |  |
| CPIJ004849 | eukaryotic translation initiation factor 4 gamma | RTT | -0.849 |  |  |
| CPIJ005545 | conserved hypothetical protein | RTT | -1.237 |  |  |
| CPIJ007556 | regulator of ribosome biosynthesis | RTT | -0.826 |  |  |
| CPIJ007894 | conserved hypothetical protein | RTT | -0.776 |  |  |
| CPIJ008494 | histone h2a | RTT | -0.804 |  |  |
| CPIJ009286 | ATP-dependent RNA helicase vasa | RTT | -0.795 |  |  |
| CPIJ009916 | brix domain-containing protein 2 | RTT | -0.844 |  |  |
| CPIJ010363 | histone H1 | RTT | 0.814 |  |  |
| CPIJ012113 | brix domain-containing protein 2 | RTT | -0.842 |  |  |
| CPIJ012140 | conserved hypothetical protein | RTT | -0.791 |  |  |
| CPIJ013860 | eukaryotic translation initiation factor 2 subunit beta | RTT | -0.791 |  |  |
| CPIJ018005 | helicase conserved C-terminal domain containing protein | RTT | 0.85 |  |  |
| CPIJ018444 | DNA-directed RNA polymeraseI | RTT | -0.841 |  |  |
| CPIJ018569 | DNA-directed RNA polymeraseI | RTT | -0.87 |  |  |
| CPIJ019134 | U1 small nuclear ribonucleoprotein A | RTT | -0.827 |  |  |
| CPIJ020000 | DNA polymerase epsilon subunit 3 | RTT | -0.773 |  |  |
| CPIJ021733 | Ser tRNA | RTT | -0.872 |  |  |
| CPIJ021741 | Ser tRNA | RTT | -0.786 |  |  |
| CPIJ026105 | Ser tRNA | RTT | -0.925 |  |  |
| CPIJ011772 | homeobox protein | RTT, DIV |  |  | -0.776 |
| CPIJ006712 | conserved hypothetical protein | RTT, DIV | -0.868 | -0.991 |  |
| CPIJ009445 | DEAD box ATP-dependent RNA helicase | RTT, DIV |  | 1.5 |  |
| CPIJ014707 | conserved hypothetical protein | RTT, DIV |  | -0.999 |  |
| CPIJ005218 | RNA m5u methyltransferase | RTT, DIV | -0.84 |  |  |
| CPIJ013558 | 23S rRNA methyltransferase | RTT, DIV | -0.863 |  |  |
| CPIJ013673 | 39S ribosomal protein L11, mitochondrial | RTT, RSM | -1.458 | -1.316 |  |
| CPIJ018777 | guanine nucleotide binding protein | RTT, RSM | -0.911 | -1.246 |  |
| CPIJ018940 | guanine nucleotide binding protein | RTT, RSM | -1.05 | -1.479 |  |
| CPIJ019257 | 39S ribosomal protein L19, mitochondrial | RTT, RSM | -0.816 | -1.168 |  |
| CPIJ006060 | 39S ribosomal protein L47, mitochondrial | RTT, RSM |  | -0.783 |  |
| CPIJ011449 | 39S ribosomal protein L13 | RTT, RSM |  | -0.808 |  |
| CPIJ007189 | DNA-J | RTT, RSM | -0.775 |  |  |
| CPIJ013130 | 39S ribosomal protein L19, mitochondrial | RTT, RSM | -0.809 |  |  |
| CPIJ003889 | breast carcinoma amplified sequence 2 | RTT, TRP | -1.314 | -1.517 |  |
| CPIJ004285 | neural/ectodermal development factor IMP-L2 | DIV, IMM | 1.788 | 3.996 | 4.078 |
| CPIJ000210 | cysteine-rich venom protein | DIV | 4.471 | 4.706 | 3.898 |
| CPIJ009451 | conserved hypothetical protein | DIV | 4.471 | 4.336 | 2.8 |
| CPIJ005640 | heat shock protein 26 | DIV | 1.682 | 3.654 | 3.283 |
| CPIJ015476 | aspartyl-tRNA synthetase | DIV |  | 1.464 | 3.05 |
| CPIJ001748 | conserved hypothetical protein | DIV |  | 3.013 | 3.396 |
| CPIJ008853 | maltose phosphorylase | DIV | 1.255 | 3.049 | 2.574 |
| CPIJ007536 | hypothetical protein | DIV |  | 1.585 | 2.579 |
| CPIJ014902 | CDC42_ANOGA CDC42 homolog | DIV |  | 2.514 | 2.886 |
| CPIJ003202 | conserved hypothetical protein | DIV | 2.391 | 2.329 | 3.136 |
| CPIJ000134 | phosphatidate phosphatase | DIV |  | 1.942 | 2.602 |
| CPIJ007775 | apolipophorin-III | DIV |  |  | 1.783 |
| CPIJ000755 | conserved hypothetical protein | DIV |  | 2.064 | 2.651 |
| CPIJ003456 | uricase | DIV | 1.652 | 2.073 | 2.641 |
| CPIJ003582 | TATA-binding protein-associated factor 172 | DIV | 1.211 | 1.684 | 2.344 |
| CPIJ005644 | lethal | DIV | -1.74 | 5.033 | 3.373 |
| CPIJ015408 | branched-chain amino acid aminotransferase | DIV | 2.35 | 3.462 | 2.552 |
| CPIJ001263 | membrane-bound alkaline phosphatase | DIV |  | 2.838 | 2.244 |
| CPIJ002519 | conserved hypothetical protein | DIV | 0.978 | 2.371 | 1.931 |
| CPIJ011609 | conserved hypothetical protein | DIV | 1.152 | 1.79 | 2.055 |
| CPIJ017909 | tankyrase | DIV |  | 1.748 | 2.299 |
| CPIJ012748 | conserved hypothetical protein | DIV |  | 2.201 | 2.178 |
| CPIJ012373 | virus-induced RNA | DIV | 1.335 | 1.468 | 2.126 |
| CPIJ005645 | heat shock protein 22 | DIV |  | 5.365 | 4.807 |
| CPIJ009030 | glutaredoxin 2 | DIV, RSM |  | 1.641 | 2.063 |
| CPIJ016642 | predicted protein | DIV | -3.172 | -2.268 | -1.552 |
| CPIJ006320 | rieske-domain protein Neverland | DIV |  | 0.983 | 1.539 |
| CPIJ003945 | three prime repair exonuclease 1 | DIV |  | 1.599 | 1.86 |
| CPIJ005639 | lethal | DIV |  | 1.524 | 2.026 |
| CPIJ008851 | maltose phosphorylase | DIV | 1.209 | 1.963 | 1.535 |
| CPIJ017297 | quinone oxidoreductase | DIV | 0.93 | 2.19 | 1.872 |
| CPIJ003242 | conserved hypothetical protein | DIV | 1.392 | 2.566 | 2.339 |
| CPIJ002602 | chitotriosidase-1 | DIV, IMM |  |  | 1.628 |
| CPIJ016643 | predicted protein | DIV | -1.395 | -0.994 |  |
| CPIJ004200 | serine/threonine-protein kinase 40 | DIV | 0.808 | 1.459 | 2.117 |
| CPIJ016890 | sans | DIV | 1.362 | 1.56 | 1.849 |
| CPIJ017898 | phosphatidic acid phosphatase | DIV |  | 1.201 | 1.686 |
| CPIJ002510 | conserved hypothetical protein | DIV |  | 1.029 | 1.352 |
| CPIJ007773 | conserved hypothetical protein | DIV | 0.877 | 1.717 | 1.967 |
| CPIJ006312 | conserved hypothetical protein | DIV | 2.146 | 2.423 | 1.928 |
| CPIJ010531 | ABC1 family protein | DIV | -0.866 | 1.049 | 1.989 |
| CPIJ009320 | ethanolamine-phosphate cytidylyltransferase | DIV |  | 1.072 | 1.511 |
| CPIJ013749 | delta | DIV |  | 0.956 | 1.722 |
| CPIJ018145 | swiprosin | DIV |  | 1.666 | 1.678 |
| CPIJ003550 | 78 kDa glucose-regulated protein | DIV | -1.63 | -1.429 | 2.066 |
| CPIJ005642 | heat shock protein 27 | DIV |  | 5.305 | 4.856 |
| CPIJ008164 | conserved hypothetical protein | DIV |  | 1.326 | 1.409 |
| CPIJ005643 | lethal | DIV |  | 1.38 | 1.738 |
| CPIJ000014 | conserved hypothetical protein | DIV |  | 0.901 | 1.881 |
| CPIJ000493 | conserved hypothetical protein | DIV | 1.176 | 1.738 | 1.798 |
| CPIJ006963 | conserved hypothetical protein | DIV | 1.442 | 1.939 | 2.129 |
| CPIJ013725 | dimethylaniline monooxygenase | DIV, TRP |  | 1.367 | 2.545 |
| CPIJ016525 | histidyl-tRNA synthetase | DIV |  | 1.149 | 1.884 |
| CPIJ015211 | imaginal discs arrested | DIV | 1.619 | 1.892 | 1.492 |
| CPIJ005049 | conserved hypothetical protein | DIV |  | 0.878 | 1.824 |
| CPIJ014100 | conserved hypothetical protein | DIV | 1.586 | 2.238 | 1.561 |
| CPIJ003158 | G protein-coupled receptor | DIV | 0.8 | 0.971 | 1.423 |
| CPIJ009709 | conserved hypothetical protein | DIV | 1.723 | 3.287 | 2.006 |
| CPIJ018205 | chymotrypsin-2 | DIV, PRT, MET, IMM, DIG, TRP |  | 1.673 | 2.328 |
| CPIJ004859 | growth hormone-inducible transmembrane protein | DIV |  |  | 1.87 |
| CPIJ018976 | 1-acylglycerol-3-phosphate acyltransferase | DIV | 1.503 | 1.938 | 1.488 |
| CPIJ014970 | niemann-Pick C1 protein | DIV |  | 1.403 | 1.466 |
| CPIJ018104 | zinc finger and SCAN domain-containing protein 12 | DIV |  |  | 1.698 |
| CPIJ011681 | synaptosomal-associated protein 29 | DIV |  |  | 1.488 |
| CPIJ013746 | kinase | DIV | 0.785 | 0.911 | 1.2 |
| CPIJ017084 | conserved hypothetical protein | DIV | 0.819 | 1.021 | 1.235 |
| CPIJ002117 | conserved hypothetical protein | DIV | 1.704 | 1.733 | 1.787 |
| CPIJ006714 | ras-related protein Rab-8A | DIV |  | 1.238 | 1.54 |
| CPIJ008852 | maltose phosphorylase | DIV | 1.486 | 2.124 | 1.545 |
| CPIJ002448 | lipoma preferred partner/lpp | DIV | 1.116 | 1.54 | 1.496 |
| CPIJ004450 | GTP-binding protein yptV3 | DIV |  | 1.63 | 1.562 |
| CPIJ007838 | chymotrypsin-2 | DIV, PRT, MET, IMM, DIG, TRP |  | 1.584 | 2.078 |
| CPIJ005118 | fasciclin | DIV | 1.705 | 2.123 | 1.573 |
| CPIJ007837 | zinc finger protein | DIV | 0.918 | 2.376 | 1.019 |
| CPIJ006113 | internalin A | DIV | 1.257 | 1.621 | 1.867 |
| CPIJ012947 | conserved hypothetical protein | DIV |  | 1.201 | 1.7 |
| CPIJ012355 | regulator of chromosome condensation | DIV |  |  | 1.209 |
| CPIJ003523 | glutamine gamma-glutamyltransferase | DIV |  | 1.065 | 1.145 |
| CPIJ001290 | kappa bs | DIV |  | 1.694 | 1.524 |
| CPIJ008182 | L47123_1 ferritin | DIV | 1.33 | 1.823 | 1.56 |
| CPIJ008018 | dual specificity protein phosphatase | DIV |  | 1.327 | 1.494 |
| CPIJ011082 | heat shock protein 70 B2 | DIV, RTT | 1.293 | 3.434 | 3.217 |
| CPIJ003516 | signal transducing adapter molecule 1 | DIV | 0.961 | 1.343 | 1.542 |
| CPIJ003106 | serine/threonine-protein kinase RIO3 | DIV |  |  | 1.588 |
| CPIJ011725 | conserved hypothetical protein | DIV, RTT | 2.611 | 2.452 | 1.437 |
| CPIJ015882 | hypothetical protein | DIV | -0.973 |  | 0.892 |
| CPIJ013070 | phenylalanyl-tRNA synthetase beta chain | DIV | 2.73 | 3.131 | 1.096 |
| CPIJ013215 | conserved hypothetical protein | DIV | 3.429 | 3.133 | 1.763 |
| CPIJ005646 | alphaA-crystallin | DIV | 1.052 | 4.245 | 3.782 |
| CPIJ005271 | trypsin 2 | DIV, PRT, MET, IMM, DIG, TRP | 2.56 | 3.426 | 1.809 |
| CPIJ017218 | lupus la ribonucleoprotein | DIV | -1.832 |  | 1.008 |
| CPIJ014022 | coronin | DIV | 0.791 | 1.659 | 1.389 |
| CPIJ002783 | AN1-type zinc finger protein 2B | DIV |  | 1.239 | 1.794 |
| CPIJ007848 | serine/threonine protein kinase | DIV |  | 1.314 | 1.46 |
| CPIJ018042 | conserved hypothetical protein | DIV, CST | 0.882 | 2.011 | 1.398 |
| CPIJ001779 | syntenin-1 | DIV |  | 1.168 | 1.213 |
| CPIJ014120 | endoU protein | DIV |  |  | 1.154 |
| CPIJ014573 | phenylalanyl-tRNA synthetase beta chain | DIV | 2.57 | 3.012 | 1.065 |
| CPIJ007982 | conserved hypothetical protein | DIV |  |  | 1.549 |
| CPIJ005661 | leucine rich protein | DIV | 1.352 | 2.951 | 1.441 |
| CPIJ016906 | conserved hypothetical protein | DIV | 0.963 | 1.618 | 1.466 |
| CPIJ015901 | conserved hypothetical protein | DIV | 1.061 | 1.738 | 1.445 |
| CPIJ015439 | conserved hypothetical protein | DIV |  | 2.127 | 1.518 |
| CPIJ009383 | serine/threonine-protein kinase rio3 | DIV |  |  | 1.154 |
| CPIJ006917 | NFkappaB essential modulator | DIV, IMM | 1.044 | 1.351 | 1.419 |
| CPIJ000791 | conserved hypothetical protein | DIV |  | 0.794 | 1.463 |
| CPIJ007154 | conserved hypothetical protein | DIV | 0.993 | 1.898 | 1.474 |
| CPIJ011975 | PRA1 family protein 2 | DIV | -0.931 |  | 1.17 |
| CPIJ007754 | rac serine/threonine kinase | DIV | 1.464 | 1.716 | 1.589 |
| CPIJ013330 | eukaryotic peptide chain release factor GTP-binding subunit | DIV, RTT |  | 1.206 | 1.328 |
| CPIJ014453 | conserved hypothetical protein | DIV, CSR | 1.145 | 1.547 | 1.054 |
| CPIJ017109 | phosphatidic acid phosphatase | DIV |  | 1.061 | 1.186 |
| CPIJ003504 | ribonuclease P protein subunit p30 | DIV, RTT | -0.814 | 0.916 |  |
| CPIJ018373 | pra1 protein | DIV |  |  | 1.309 |
| CPIJ011451 | conserved hypothetical protein | DIV |  | 0.835 | 1.173 |
| CPIJ013695 | Bub1 | DIV |  | 1.022 | 0.864 |
| CPIJ007984 | f-box/lrr protein | DIV | 2.318 | 2.732 | 1.69 |
| CPIJ015320 | nicotinamide mononucleotide adenylyltransferase 1 | DIV |  | 1.31 | 1.31 |
| CPIJ001408 | conserved hypothetical protein | DIV |  | 1.1 | 0.93 |
| CPIJ006064 | conserved hypothetical protein | DIV |  | 0.895 | 1.087 |
| CPIJ013880 | heat shock protein 67B2 | DIV |  | 1.01 | 1.388 |
| CPIJ002992 | UDP-glucose 6-dehydrogenase | DIV |  | 0.858 | 1.13 |
| CPIJ016814 | conserved hypothetical protein | DIV |  | 2.164 | 1.537 |
| CPIJ008919 | tyrosine phosphatase n9 | DIV | 1.168 | 1.698 | 1.324 |
| CPIJ003165 | proteasome activator complex subunit 3 | DIV |  |  | 1.103 |
| CPIJ016374 | conserved hypothetical protein | DIV |  |  | 0.905 |
| CPIJ010173 | testis-specific protein pbs13 | DIV | 1.526 | 1.929 | 1.697 |
| CPIJ017546 | HIG1 domain family member 1A | DIV | -1.325 |  | 1.129 |
| CPIJ003754 | conserved hypothetical protein | DIV |  | 0.895 | 1.174 |
| CPIJ005745 | c3f | DIV | 1.53 | 1.191 | 1.045 |
| CPIJ017385 | pom1 | DIV |  | 0.793 | 1.014 |
| CPIJ019153 | conserved hypothetical protein | DIV | -1.146 |  | 1.104 |
| CPIJ009263 | skeletrophin | DIV |  | 1.272 | 1.06 |
| CPIJ013232 | phsophatase-2a | DIV |  |  | 1.052 |
| CPIJ011184 | deoxyribonuclease I | DIV | -1.236 | -1.005 |  |
| CPIJ015730 | apolipoprotein D | DIV |  | 1.17 | 0.977 |
| CPIJ002003 | conserved hypothetical protein | DIV | 0.819 | 1.258 | 1.017 |
| CPIJ019593 | conserved hypothetical protein | DIV | 2.863 | 2.123 |  |
| CPIJ002100 | autophagy-specific gene 12 | DIV |  |  | 1.043 |
| CPIJ002758 | cellular tumor antigen p53 | DIV |  |  | 1.172 |
| CPIJ015482 | conserved hypothetical protein | DIV | 0.938 | 0.914 | 1.667 |
| CPIJ008043 | folylpolyglutamate synthase, mitochondrial | DIV |  | 0.91 | 1.137 |
| CPIJ012025 | U4/U6 small nuclear ribonucleoprotein Prp4 | DIV |  |  | 1.047 |
| CPIJ002931 | ubiquitin protein ligase | DIV |  | 1.257 | 1.15 |
| CPIJ012074 | three prime repair exonuclease 1 | DIV |  | 1.283 | 1.102 |
| CPIJ001761 | Ofd1 protein | DIV |  |  | 1.07 |
| CPIJ003793 | BTB/POZ domain-containing protein 9 | DIV | 1.111 | 1.392 | 1.178 |
| CPIJ006231 | rolling pebbles | DIV |  | 1.186 | 1.092 |
| CPIJ000668 | conserved hypothetical protein | DIV |  | 0.976 | 1.002 |
| CPIJ016766 | conserved hypothetical protein | DIV | 1.368 | 1.57 | 1.167 |
| CPIJ019476 | vacuolar protein sorting-associating protein 4A | DIV |  | 0.936 | 1.063 |
| CPIJ004017 | cyclin l | DIV | 0.969 | 1.297 | 1.156 |
| CPIJ004908 | sarm1 | DIV |  | 1.311 | 1.29 |
| CPIJ010561 | peptidyl-prolyl cis-trans isomerase H | DIV |  |  | 0.968 |
| CPIJ011572 | serine/threonine-protein kinase rio3 | DIV |  |  | 1.148 |
| CPIJ007823 | glial maturation factor | DIV |  | 1.133 | 1.026 |
| CPIJ015727 | apolipoprotein D | DIV, TRP |  | -1.517 |  |
| CPIJ000757 | zinc finger protein 674 | DIV |  | 0.989 | 1.107 |
| CPIJ014561 | conserved hypothetical protein | DIV |  | 1.081 | 1.151 |
| CPIJ018644 | conserved hypothetical protein | DIV |  | 0.98 | 1.15 |
| CPIJ018539 | wd-repeat protein | DIV | -1.291 |  |  |
| CPIJ002084 | grb2-associated binder | DIV |  | 0.805 | 1.071 |
| CPIJ010247 | raw | DIV |  | 0.818 | 0.988 |
| CPIJ012839 | 1-acylglycerol-3-phosphate acyltransferase | DIV | 1.572 | 1.385 | 1.001 |
| CPIJ017615 | apolipoprotein D | DIV |  | 0.94 |  |
| CPIJ008588 | mRNA turnover protein 4 | DIV | -1.737 |  |  |
| CPIJ010489 | conserved hypothetical protein | DIV |  | 1.158 | 1.185 |
| CPIJ016581 | presenilin enhancer | DIV |  | 0.865 | 0.897 |
| CPIJ009329 | conserved hypothetical protein | DIV | -1.124 | -1.331 |  |
| CPIJ009996 | sprint | DIV |  | 0.788 | 1.225 |
| CPIJ003300 | serine/threonine protein kinase | DIV |  | 0.937 | 1.18 |
| CPIJ009089 | ras-related protein Rab-7 | DIV | 0.902 | 1.111 | 0.852 |
| CPIJ013497 | eukaryotic translation initiation factor 1b | DIV |  | 0.785 | 1.157 |
| CPIJ001480 | conserved hypothetical protein | DIV |  | 1.177 | 0.873 |
| CPIJ005772 | trithorax protein ash2 | DIV | 1.804 | 1.439 | 0.901 |
| CPIJ017502 | ubiquitin protein | DIV |  |  | 1.356 |
| CPIJ005048 | conserved hypothetical protein | DIV | 0.87 | 1.06 | 1.011 |
| CPIJ010037 | vacuolar protein sorting 13D | DIV |  | 0.957 | 1.069 |
| CPIJ015135 | penguin | DIV |  | 0.925 | 0.883 |
| CPIJ008259 | hypothetical protein | DIV | 0.994 | 1.144 | 0.899 |
| CPIJ003603 | conserved hypothetical protein | DIV |  | 1.288 | 1.088 |
| CPIJ013313 | apolipoprotein D | DIV |  | 1.028 | 0.824 |
| CPIJ001502 | conserved hypothetical protein | DIV |  |  | 0.864 |
| CPIJ010679 | valyl-tRNA synthetase | DIV |  | 1.216 | 1.075 |
| CPIJ017302 | conserved hypothetical protein | DIV | -0.872 |  | 1.029 |
| CPIJ009112 | plasminogen | DIV, PRT, MET, IMM, DIG, TRP | 1.357 | 1.029 | 1.095 |
| CPIJ011215 | NULL | DIV |  | 0.914 | 1.401 |
| CPIJ006282 | serine/threonine protein kinase | DIV |  | 0.85 | 1.123 |
| CPIJ016497 | thrombospondin-4 | DIV |  |  | 1.134 |
| CPIJ017801 | wd-repeat protein | DIV | -1.215 |  |  |
| CPIJ000203 | conserved hypothetical protein | DIV |  |  | 1.049 |
| CPIJ013498 | sui1 | DIV |  | 0.78 | 1.073 |
| CPIJ018160 | lipid storage droplets surface binding protein 2 | DIV | -0.963 |  | 0.897 |
| CPIJ013967 | transmembrane protein 49 | DIV |  |  | 1.223 |
| CPIJ010815 | vacuolar protein sorting-associated protein | DIV, TRP |  | 0.832 | 1.055 |
| CPIJ017844 | Tetratricopeptide repeat protein | DIV, RSM | -1.519 | -1.492 | 1.089 |
| CPIJ012735 | translation initiation factor eIF-2B subunit epsilon | DIV |  |  | 1.134 |
| CPIJ005630 | conserved hypothetical protein | DIV | 1.38 | 1.734 |  |
| CPIJ009002 | purine biosynthesis protein 6, pur6 | DIV |  |  | 0.899 |
| CPIJ003163 | conserved hypothetical protein | DIV |  |  | 0.965 |
| CPIJ011365 | E3 ubiquitin-protein ligase mib1 | DIV | 2.729 | 1.864 | 1.186 |
| CPIJ007981 | conserved hypothetical protein | DIV |  |  | 0.971 |
| CPIJ015942 | c-AMP dependent protein kinase typeI-beta regulatory subunit | DIV |  | 1.186 | 1.152 |
| CPIJ002340 | conserved hypothetical protein | DIV | 0.965 | 1.075 | 0.974 |
| CPIJ001560 | calcium-binding protein | DIV |  | -0.964 |  |
| CPIJ002173 | conserved hypothetical protein | DIV |  | 1.579 | 1.078 |
| CPIJ002643 | CDNA sequence | DIV | -1.038 |  | 0.933 |
| CPIJ009633 | conserved hypothetical protein | DIV | 1.277 | 1.354 | 1.179 |
| CPIJ015726 | apolipoprotein D | DIV, TRP |  | -1.418 |  |
| CPIJ011659 | conserved hypothetical protein | DIV | 0.833 | 1.018 | 1.018 |
| CPIJ007769 | testis development protein prtd | DIV, RTT | 0.824 | 0.844 | 0.848 |
| CPIJ012225 | cell growth-regulating nucleolar protein | DIV | -1.317 |  |  |
| CPIJ012099 | GTP-binding protein YPTC4 | DIV |  |  | 0.895 |
| CPIJ014171 | conserved hypothetical protein | DIV |  | 1.596 | 1.19 |
| CPIJ000135 | conserved hypothetical protein | DIV |  |  | 0.802 |
| CPIJ004272 | ubiquitin-conjugating enzyme rad6 | DIV |  |  | 0.929 |
| CPIJ004013 | slender lobes | DIV |  | 1.003 | 0.995 |
| CPIJ012513 | tetratricopeptide repeat protein 4 | DIV | -1.049 |  |  |
| CPIJ000285 | RING finger protein 185 | DIV |  | 0.858 | 1.189 |
| CPIJ000504 | sensitized chromosome inheritance modifier 19 | DIV | -0.893 |  | 0.859 |
| CPIJ000420 | mothers against dpp | DIV |  | 1.057 |  |
| CPIJ002707 | zinc finger protein | DIV | 1.065 | 1.588 | 0.988 |
| CPIJ009268 | hook | DIV |  |  | 0.76 |
| CPIJ014406 | hypothetical protein | DIV |  | 1.194 |  |
| CPIJ000291 | conserved hypothetical protein | DIV | 1.826 | 1.86 | 0.803 |
| CPIJ019535 | imaginal discs arrested | DIV | 1.186 | 1.223 | 0.884 |
| CPIJ004340 | AMPK-beta subunit | DIV |  |  | 0.836 |
| CPIJ002498 | lysosomal thioesterase PPT2 | DIV | 1.262 | 1.123 | 1.046 |
| CPIJ011583 | hypothetical protein | DIV |  | -1.018 |  |
| CPIJ003339 | fermitin 1 | DIV |  | 0.955 | 1.149 |
| CPIJ000181 | schnurri | DIV | 1.211 | 1.464 | 1.155 |
| CPIJ003774 | tyrosine aminotransferase | DIV |  |  | 1.23 |
| CPIJ017100 | spartin | DIV |  | 0.92 | 1.399 |
| CPIJ003008 | charged multivesicular body protein 1b | DIV |  |  | 0.847 |
| CPIJ000921 | menin | DIV | 0.947 | 1.231 | 0.926 |
| CPIJ005021 | vacuolar protein sorting-associated protein 18 | DIV |  |  | 0.791 |
| CPIJ016439 | CCR4-NOT transcription complex subunit 7 | DIV |  |  | 0.83 |
| CPIJ013723 | dimethylaniline monooxygenase | DIV | 0.844 | 1.485 | 1.161 |
| CPIJ014446 | conserved hypothetical protein | DIV |  |  | 1.033 |
| CPIJ015947 | NULL | DIV | 1.109 | 1.06 | 0.822 |
| CPIJ010915 | arginine n-methyltransferase | DIV | -0.803 |  |  |
| CPIJ000895 | conserved hypothetical protein | DIV | 1.073 | 1.267 | 1.339 |
| CPIJ002978 | aminotransferase | DIV | 1.264 | 1.688 | 1.227 |
| CPIJ003241 | conserved hypothetical protein | DIV |  | 0.759 | 0.961 |
| CPIJ001423 | ubiquitin-activating enzyme E1 | DIV |  |  | 0.858 |
| CPIJ005230 | pmp22 peroxisomal membrane protein | DIV |  |  | 0.842 |
| CPIJ019794 | prov protein | DIV, RSM | -1.126 | -1.077 | 0.849 |
| CPIJ005006 | arsenite inducible RNA associated protein aip-1 | DIV |  |  | 1.102 |
| CPIJ000084 | jun | DIV, RTT | 0.981 | 1.449 | 1.042 |
| CPIJ007658 | werner syndrome helicase | DIV | 1.442 | 1.819 | 1.063 |
| CPIJ010534 | endoribonuclease Dicer | DIV |  |  | 0.877 |
| CPIJ017390 | cytosolic Fe-S cluster assembling factor CFD1 | DIV | -0.947 |  | 1.13 |
| CPIJ009140 | conserved hypothetical protein | DIV |  |  | 0.964 |
| CPIJ008646 | conserved hypothetical protein | DIV |  | 0.77 |  |
| CPIJ008396 | RAP1B | DIV |  |  | 0.79 |
| CPIJ000288 | serine/threonine-protein kinase 3 | DIV |  | 1.145 | 0.997 |
| CPIJ003558 | deoxyhypusine hydroxylase | DIV |  |  | 0.758 |
| CPIJ013434 | serologically defined colon cancer antigen 1 | DIV |  |  | 0.755 |
| CPIJ008671 | zinc-finger protein ZPR1 | DIV |  |  | 0.841 |
| CPIJ016258 | probable ER retained protein | DIV | -1.415 | -1.699 |  |
| CPIJ013889 | conserved hypothetical protein | DIV | 0.805 | 0.915 |  |
| CPIJ005586 | histidine triad protein member | DIV, RTT |  |  | 0.79 |
| CPIJ007348 | nuclear movement protein nudC | DIV | -0.839 |  | 0.795 |
| CPIJ009031 | GXIVsPLA2 | DIV |  |  | 0.789 |
| CPIJ006276 | F-box/WD repeat protein 5 | DIV | 0.866 | 0.807 | 0.755 |
| CPIJ004172 | threonyl-tRNA synthetase | DIV |  | 0.79 | 1.025 |
| CPIJ005169 | ras-related protein Rab-10 | DIV |  |  | 0.862 |
| CPIJ017754 | conserved hypothetical protein | DIV |  |  | 0.98 |
| CPIJ001482 | pak-interacting exchange factor, beta-pix/cool-1 | DIV |  | 0.849 |  |
| CPIJ011532 | conserved hypothetical protein | DIV |  |  | 0.933 |
| CPIJ005628 | conserved hypothetical protein | DIV |  |  | 0.884 |
| CPIJ013822 | vacuolar protein sorting-associated protein 33A | DIV |  |  | 0.935 |
| CPIJ007520 | methionine-R-sulfoxide reductase | DIV |  |  | 0.956 |
| CPIJ000595 | conserved hypothetical protein | DIV | 1.238 | 1.412 | 1.01 |
| CPIJ001500 | WD repeat protein 12 | DIV | -0.921 |  |  |
| CPIJ018645 | conserved hypothetical protein | DIV |  | 0.848 | 0.798 |
| CPIJ015424 | charged multivesicular body protein 2a | DIV |  | 0.902 | 0.878 |
| CPIJ018658 | ubiquitin-conjugating enzyme E2 G2 | DIV |  |  | 0.77 |
| CPIJ013314 | apolipoprotein D | DIV |  | 1.032 |  |
| CPIJ006931 | conserved hypothetical protein | DIV | -1.134 |  |  |
| CPIJ000357 | hsp70 binding protein | DIV | -1.015 |  | 0.936 |
| CPIJ008391 | crooked neck | DIV |  |  | 0.816 |
| CPIJ005038 | conserved hypothetical protein | DIV |  |  | 0.886 |
| CPIJ001248 | DNA-binding protein smubp-2 | DIV |  | 0.939 |  |
| CPIJ015883 | f-box/lrr protein, drome | DIV |  | 1.197 | 0.877 |
| CPIJ001191 | HIT zinc finger family protein | DIV |  | 0.852 |  |
| CPIJ005730 | conserved hypothetical protein | DIV |  |  | 0.765 |
| CPIJ006485 | rho guanine dissociation factor | DIV |  |  | 0.807 |
| CPIJ011187 | deoxyribonuclease I | DIV |  |  | 0.765 |
| CPIJ001250 | potentail helicase MOV-10 | DIV |  | 1.385 |  |
| CPIJ003352 | zinc finger | DIV |  |  | 0.815 |
| CPIJ008854 | maltose phosphorylase | DIV |  |  | 0.827 |
| CPIJ006783 | DNA-binding protein D-ETS-4 | DIV |  | 0.82 |  |
| CPIJ003288 | conserved hypothetical protein | DIV, RTT |  |  | 0.871 |
| CPIJ016151 | conserved hypothetical protein | DIV |  |  | 0.758 |
| CPIJ001920 | conserved hypothetical protein | DIV |  | 1.232 | 0.798 |
| CPIJ001488 | conserved hypothetical protein | DIV |  | 1.146 | 1.028 |
| CPIJ003223 | conserved hypothetical protein | DIV |  | 0.911 | 1.059 |
| CPIJ007359 | purine nucleoside phosphorylase | DIV | 0.897 | 0.884 |  |
| CPIJ019805 | conserved hypothetical protein | DIV, RTT |  | 0.886 | 0.936 |
| CPIJ008670 | wd-repeat protein | DIV | 1.052 | 1.025 |  |
| CPIJ003559 | tubulin-specific chaperone A | DIV | -1.052 |  |  |
| CPIJ001938 | BTG1 protein | DIV | 1.906 | 1.784 | 0.851 |
| CPIJ002913 | conserved hypothetical protein | DIV | 0.761 | 1.177 | 1.174 |
| CPIJ016941 | conserved hypothetical protein | DIV | 1.19 | 1.217 | 1.023 |
| CPIJ011373 | conserved hypothetical protein | DIV |  | 0.814 | 0.784 |
| CPIJ000080 | UBX domain-containing protein 1 | DIV |  |  | 0.777 |
| CPIJ007150 | conserved hypothetical protein | DIV |  |  | 0.812 |
| CPIJ008699 | limd1 | DIV |  | 1.052 |  |
| CPIJ006689 | conserved hypothetical protein | DIV | 0.813 | 0.997 | 0.815 |
| CPIJ008123 | malate dehydrogenase | DIV |  | -0.752 |  |
| CPIJ005272 | trypsin 3A1 | DIV, PRT, IMM, MET, DIG, TRP |  |  | -0.846 |
| CPIJ015893 | disulfide isomerase | DIV | -1.297 | -2.119 | -0.936 |
| CPIJ003149 | dj-1 protein | DIV |  | -1.199 | -0.843 |
| CPIJ019318 | conserved hypothetical protein | DIV | -1.25 | -1.695 | -0.954 |
| CPIJ005086 | glycogen synthase | DIV |  | -0.935 | -0.939 |
| CPIJ012768 | malate dehydrogenase | DIV |  | -0.825 | -0.916 |
| CPIJ011328 | GTP:AMP phosphotransferase mitochondrial | DIV |  | -1.203 | -1.193 |
| CPIJ003081 | conserved hypothetical protein | DIV |  |  | -0.838 |
| CPIJ013253 | ATP-binding cassette sub-family D member 1 | DIV, TRP |  | -1.406 |  |
| CPIJ013637 | zinc finger CCHC-type and RNA-binding motif-containing protein 1 | DIV |  | -1.625 | -1.001 |
| CPIJ006941 | g-protein coupled receptor | DIV |  |  | -0.937 |
| CPIJ019606 | asparagine synthetase | DIV | -0.812 | -0.794 |  |
| CPIJ007651 | conserved hypothetical protein | DIV | -0.908 | -1.318 |  |
| CPIJ018492 | conserved hypothetical protein | DIV |  |  | -0.783 |
| CPIJ020072 | conserved hypothetical protein | DIV |  |  | -0.792 |
| CPIJ011080 | cell division control protein 2 cognate | DIV |  | -0.947 |  |
| CPIJ017124 | proline synthetase co-transcribed protein-like protein | DIV |  | -1.411 |  |
| CPIJ010984 | tetraspanin | DIV |  |  | -0.768 |
| CPIJ017079 | conserved hypothetical protein | DIV | -0.85 | -1.289 | -0.853 |
| CPIJ014326 | conserved hypothetical protein | DIV |  | -1.266 | -0.984 |
| CPIJ017394 | peptidyl-tRNA hydrolase 2, mitochondrial | DIV |  | -1.341 | -0.936 |
| CPIJ013926 | conserved hypothetical protein | DIV |  |  | -0.857 |
| CPIJ016430 | pyruvate dehydrogenase | DIV |  | -0.973 | -0.799 |
| CPIJ018887 | conserved hypothetical protein | DIV |  |  | -0.997 |
| CPIJ011350 | conserved hypothetical protein | DIV | -1.535 | -1.973 | -1.03 |
| CPIJ011826 | conserved hypothetical protein | DIV |  | -1.456 | -0.811 |
| CPIJ019133 | quinone oxidoreductase | DIV | -0.778 | -1.236 | -1.058 |
| CPIJ013721 | dimethylaniline monooxygenase 5 | DIV, TRP |  | -1.711 | -0.789 |
| CPIJ011746 | R2D2 | DIV |  |  | -1.543 |
| CPIJ006434 | dihydroxyacetone kinase | DIV | 1.064 |  | -0.829 |
| CPIJ016467 | malate dehydrogenase, mitochondrial | DIV |  | -0.908 | -0.786 |
| CPIJ001291 | fatty acid hydroxylase | DIV |  |  | -0.985 |
| CPIJ014500 | PA domain-containing protein | DIV |  | -0.985 | -1 |
| CPIJ000306 | conserved hypothetical protein | DIV |  | -1.031 | -0.856 |
| CPIJ007013 | histidine triad nucleotide-binding protein 1 | DIV |  |  | -0.802 |
| CPIJ000098 | electron transfer flavoprotein-ubiquinone oxidoreductase | DIV, RSM | -0.79 | -1.848 | -0.936 |
| CPIJ006946 | proteasome subunit alpha type 2 | DIV |  | -1.725 | -1.085 |
| CPIJ002344 | 5-formyltetrahydrofolate cyclo-ligase | DIV |  | -1.02 | -1.02 |
| CPIJ003146 | rhythmically expressed gene 2 protein | DIV | -0.999 | -1.038 |  |
| CPIJ013198 | upstream transcription factor | DIV |  |  | -0.838 |
| CPIJ018032 | vitamin K-dependent protein C | DIV, PRT, DIG | -0.986 | -2.029 |  |
| CPIJ008767 | conserved hypothetical protein | DIV |  |  | -0.962 |
| CPIJ016992 | conserved hypothetical protein | DIV | -1.722 | -2.057 | -1.087 |
| CPIJ005519 | enolase | DIV | -1.473 | -1.721 | -0.793 |
| CPIJ006247 | midline fasciclin | DIV |  | -1.446 | -1.026 |
| CPIJ017178 | myoinositol oxygenase | DIV | 2.708 | 1.204 |  |
| CPIJ019428 | trypsin 2 | DIV, PRT, MET, IMM, DIG, TRP |  | -0.981 | -1.541 |
| CPIJ017475 | conserved hypothetical protein | DIV |  |  | -1.127 |
| CPIJ016930 | conserved hypothetical protein | DIV, RTT |  | -0.819 | -0.879 |
| CPIJ016529 | beadex | DIV |  |  | -0.756 |
| CPIJ004923 | conserved hypothetical protein | DIV |  | -0.896 | -1.472 |
| CPIJ005296 | selenoprotein T | DIV |  | -0.891 | -0.936 |
| CPIJ011091 | translin associated factor x | DIV |  | -1.019 | -1.006 |
| CPIJ000948 | enolase | DIV | -1.599 | -2.058 | -0.774 |
| CPIJ006361 | equilibrative nucleoside transporter | DIV, TRP | -1.081 | -1.56 | -1.276 |
| CPIJ019397 | proline synthetase associated protein | DIV |  | -1.229 |  |
| CPIJ012506 | p31A | DIV |  | -1.06 | -1.244 |
| CPIJ014182 | conserved hypothetical protein | DIV | 0.851 |  |  |
| CPIJ001740 | mannose-P-dolichol utilization defect 1 protein | DIV | -0.814 | -1.742 | -0.962 |
| CPIJ018034 | brachyurin | DIV, PRT, MET, IMM, DIG, TRP | -0.804 | -1.643 | -0.972 |
| CPIJ011556 | conserved hypothetical protein | DIV |  |  | -0.901 |
| CPIJ002389 | transcription elongation factor B polypeptide 2 | DIV |  | -1.352 | -0.904 |
| CPIJ001898 | nucleoside diphosphate-linked moiety X motif 19 | DIV |  | -1.181 | -0.909 |
| CPIJ005584 | synaptic glycoprotein SC2 | DIV |  |  | -0.858 |
| CPIJ002248 | Y-box binding protein | DIV |  |  | -1.222 |
| CPIJ006837 | axotactin | DIV |  | -1.558 | -0.923 |
| CPIJ004284 | sulfide quinone reductase | DIV | -1.329 | -2.401 | -1.286 |
| CPIJ011712 | conserved hypothetical protein | DIV |  | -1.059 | -0.915 |
| CPIJ002071 | conserved hypothetical protein | DIV |  |  | -1.262 |
| CPIJ017974 | nitric oxide synthase | DIV |  |  | -1.005 |
| CPIJ012855 | ribokinase | DIV |  | -0.844 | -0.915 |
| CPIJ010972 | zinc finger protein | DIV | 2.026 | 1.237 | -0.774 |
| CPIJ019170 | conserved hypothetical protein | DIV |  | -1.352 | -0.954 |
| CPIJ010302 | trypsin | DIV, PRT, MET, IMM, DIG, TRP |  | -0.957 | -1.75 |
| CPIJ010384 | LYR motif-containing protein 2 | DIV |  | -1.189 | -1.366 |
| CPIJ005059 | isochorismatase domain-containing protein 2, mitochondrial | DIV |  | -1.098 | -0.826 |
| CPIJ010310 | conserved hypothetical protein | DIV | 0.989 |  |  |
| CPIJ016991 | conserved hypothetical protein | DIV | -1.616 | -1.923 | -1.132 |
| CPIJ006290 | ATPase | DIV, TRP |  |  | -1.232 |
| CPIJ013852 | tripeptidyl-peptidase 2 | DIV |  | -0.814 |  |
| CPIJ004883 | argininosuccinate lyase | DIV |  | -1.05 |  |
| CPIJ013632 | conserved hypothetical protein | DIV |  |  | -0.974 |
| CPIJ001433 | conserved hypothetical protein | DIV |  |  | -1.063 |
| CPIJ013731 | conserved hypothetical protein | DIV | -1.126 | -1.882 | -1.353 |
| CPIJ004310 | enolase | DIV | -1.697 | -2.002 | -1.057 |
| CPIJ010375 | conserved hypothetical protein | DIV | -1.34 | -1.809 | -1.154 |
| CPIJ011702 | enolase | DIV | -1.595 | -1.836 | -0.962 |
| CPIJ005673 | conserved hypothetical protein | DIV | -1.048 | -1.485 |  |
| CPIJ008668 | conserved hypothetical protein | DIV |  |  | -0.898 |
| CPIJ007291 | alkaline phosphatase | DIV |  |  | -1.037 |
| CPIJ007064 | conserved hypothetical protein | DIV, MET | -0.79 | -1.663 | -1.135 |
| CPIJ006028 | tyrosine protein kinase with ig domain | DIV |  |  | -0.818 |
| CPIJ004341 | nidogen | DIV |  | -1.183 | -1.205 |
| CPIJ017234 | semaphorin | DIV |  |  | -0.925 |
| CPIJ016192 | conserved hypothetical protein | DIV |  |  | -0.751 |
| CPIJ015130 | beta-1,3-galactosyltransferase | DIV | -1.743 | -2.19 | -1.129 |
| CPIJ018065 | trifunctional enzyme beta subunit | DIV |  | -1.704 | -1.317 |
| CPIJ011409 | estradiol 17-beta-dehydrogenase 2 | DIV |  | -1.07 | -0.851 |
| CPIJ011483 | PFTAIRE-interacting factor 1A | DIV |  |  | -1.421 |
| CPIJ010249 | retinoid X receptor alpha | DIV |  |  | -1.119 |
| CPIJ015662 | ecto ADP-ribosylhydrolase | DIV | -1.133 | -2.246 | -1.372 |
| CPIJ018288 | membrane protein | DIV |  | -1.018 | -1.057 |
| CPIJ017111 | conserved hypothetical protein | DIV | -1.696 | -1.93 | -1.259 |
| CPIJ015110 | synaptic vesicle protein | DIV, TRP |  | -1.144 | -0.927 |
| CPIJ009404 | trehalose-6-phosphate synthase | DIV | 0.924 | -1.299 | -1.164 |
| CPIJ012845 | G12 | DIV |  |  | -1.251 |
| CPIJ018720 | conserved hypothetical protein | DIV |  |  | -1.15 |
| CPIJ018652 | translocon-associated protein, gamma subunit | DIV |  |  | -1.031 |
| CPIJ001070 | zinc finger protein 509 | DIV |  | -1.35 | -0.941 |
| CPIJ019824 | signal peptidase complex subunit 3 | DIV |  | -1.152 | -1.398 |
| CPIJ006613 | aspartate aminotransferase | DIV |  | -0.808 | -0.984 |
| CPIJ010244 | conserved hypothetical protein | DIV |  | -1.315 | -1.332 |
| CPIJ009613 | serrate protein | DIV |  |  | -1.26 |
| CPIJ019793 | conserved hypothetical protein | DIV |  | -1.28 | -1.081 |
| CPIJ002908 | conserved hypothetical protein | DIV |  | -1.378 | -1.228 |
| CPIJ007901 | peritrophin-1 | DIV |  |  | -1.717 |
| CPIJ010030 | phosphatidylethanolamine-binding protein 2 | DIV |  |  | -1.051 |
| CPIJ016038 | nuclear receptor nhr-48 | DIV |  |  | -0.96 |
| CPIJ010861 | salivary adenosine deaminase | DIV |  | -1.071 | -1.273 |
| CPIJ005657 | phospholipid scramblase 1 | DIV | -1.086 | -1.751 | -1.581 |
| CPIJ009567 | ornithine decarboxylase | DIV |  | -1.359 | -0.781 |
| CPIJ013476 | chitooligosaccharidolytic beta-N-acetylglucosaminidase | DIV |  | -0.762 | -1.302 |
| CPIJ007213 | conserved hypothetical protein | DIV, IMM |  | -1.328 | -1.239 |
| CPIJ007238 | adenylate cyclase | DIV |  |  | -0.842 |
| CPIJ007545 | 1,2-dihydroxy-3-keto-5-methylthiopentene dioxygenase | DIV |  | -0.916 | -1.08 |
| CPIJ001360 | conserved hypothetical protein | DIV |  |  | -0.937 |
| CPIJ019517 | lactosylceramide 4-alpha-galactosyltransferase | DIV |  | -1.018 | -1.396 |
| CPIJ003610 | conserved hypothetical protein | DIV | -1.35 | -1.804 | -1.168 |
| CPIJ005815 | heparin sulfate O-sulfotransferase | DIV |  | -1.089 | -1.194 |
| CPIJ000840 | conserved hypothetical protein | DIV | -0.953 | -0.909 | -0.982 |
| CPIJ002430 | cytidine deaminase | DIV | -1.641 | -2.807 | -1.538 |
| CPIJ007694 | quick-to-court | DIV |  |  | -1.048 |
| CPIJ001318 | d-lactate dehydrognease 2 | DIV |  | -1.437 | -1.025 |
| CPIJ013826 | conserved hypothetical protein | DIV |  | -1.414 | -1.23 |
| CPIJ011574 | rhodopsin | DIV |  | 0.831 |  |
| CPIJ015282 | dopamine N acetyltransferase | DIV | -1.686 | -2.246 | -1.181 |
| CPIJ000855 | conserved hypothetical protein | DIV, RSM |  | -0.998 | -1.109 |
| CPIJ011197 | threonine dehydratase/deaminase | DIV |  | -0.862 | -0.996 |
| CPIJ005907 | conserved hypothetical protein | DIV | -1.104 | -0.907 | -1.415 |
| CPIJ005432 | NAD-dependent methanol dehydrogenase | DIV |  | -1.313 | -1.14 |
| CPIJ004614 | solute carrier family 23 member 2 | DIV, TRP |  | -1.041 | -1.544 |
| CPIJ002047 | dystrobrevin | DIV | -0.983 | -1.111 | -1.376 |
| CPIJ007716 | G protein-coupled receptor | DIV |  |  | -1.256 |
| CPIJ001798 | conserved hypothetical protein | DIV |  | -1.071 | -0.978 |
| CPIJ014674 | conserved hypothetical protein | DIV, TRP |  | -1.214 | -1.243 |
| CPIJ016311 | decaprenyl-diphosphate synthase subunit 2 | DIV |  | -0.794 | -1.035 |
| CPIJ019087 | carnitine O-acetyltransferase | DIV |  | -1.071 | -1.216 |
| CPIJ018126 | conserved hypothetical protein | DIV | 1.07 |  | -1.308 |
| CPIJ014185 | conserved hypothetical protein | DIV | 0.752 |  |  |
| CPIJ008203 | transketolase | DIV |  | -1.893 | -1.484 |
| CPIJ019292 | NULL | DIV |  | -0.889 | -1.467 |
| CPIJ017117 | Juvenile hormone-inducible protein | DIV |  | -1.47 | -1.301 |
| CPIJ005515 | conserved hypothetical protein | DIV | -1.71 | -2.107 | -1.534 |
| CPIJ018996 | conserved hypothetical protein | DIV |  |  | -0.882 |
| CPIJ007938 | asparagine synthetase B | DIV | -1.602 | -1.493 | -0.819 |
| CPIJ015513 | conserved hypothetical protein | DIV |  | -0.89 | -1.234 |
| CPIJ018724 | conserved hypothetical protein | DIV |  | -1.004 | -1.213 |
| CPIJ014256 | alanine aminotransferase | DIV |  | -1.034 | -1.312 |
| CPIJ006079 | tryptase gamma | DIV, PRT, MET, IMM, DIG, TRP |  |  | -1.054 |
| CPIJ010028 | OV-16 antigen | DIV |  | -0.794 | -1.37 |
| CPIJ003397 | aldo-keto reductase | DIV | -0.907 | -1.911 | -1.199 |
| CPIJ017506 | alanine aminotransferase | DIV |  | -1.011 | -1.331 |
| CPIJ011573 | opsin-1 | DIV |  | 1.342 |  |
| CPIJ012884 | otopetrin | DIV |  |  | -1.673 |
| CPIJ013882 | heat shock protein 67B2 | DIV | -0.862 | -1.896 | -1.453 |
| CPIJ010446 | Juvenile hormone-inducible protein | DIV |  | -1.412 | -1.703 |
| CPIJ015729 | apolipoprotein D | DIV, TRP |  |  | -1.173 |
| CPIJ008107 | conserved hypothetical protein | DIV |  | -1.638 | -1.699 |
| CPIJ010281 | lactoylglutathione lyase | DIV |  |  | -1.528 |
| CPIJ007905 | conserved hypothetical protein | DIV |  |  | -2.064 |
| CPIJ006094 | rfx5 | DIV |  | -0.807 | -0.897 |
| CPIJ005159 | conserved hypothetical protein | DIV |  | -0.811 | -1.5 |
| CPIJ004835 | catrin | DIV |  | -1.529 | -1.596 |
| CPIJ015979 | conserved hypothetical protein | DIV |  |  | -1.626 |
| CPIJ003138 | UNC93A protein | DIV |  | -0.808 | -1.166 |
| CPIJ012844 | G12 | DIV |  | -0.956 | -1.199 |
| CPIJ005558 | conserved hypothetical protein | DIV | -1.858 | -2.223 | -1.264 |
| CPIJ018369 | MSF1 protein | DIV |  | -1.215 | -1.578 |
| CPIJ000295 | aarF domain containing kinase 5 | DIV |  | -2.365 | -1.659 |
| CPIJ004401 | ornithine aminotransferase, mitochondrial | DIV | -3.136 | -2.896 | -1.33 |
| CPIJ006543 | urokinase-type plasminogen activator | DIV, PRT, MET, DIG |  | -1.584 | -1.802 |
| CPIJ011113 | ptpla domain protein | DIV |  | -1.492 | -1.468 |
| CPIJ000182 | N-acetyl neuraminate lyase | DIV | 0.858 | -1.144 | -1.644 |
| CPIJ004691 | conserved hypothetical protein | DIV, CST |  | -1.515 | -1.413 |
| CPIJ007178 | conserved hypothetical protein | DIV, PRT |  | -1.312 | -1.564 |
| CPIJ001089 | lactosylceramide 4-alpha-galactosyltransferase | DIV |  | -0.812 | -1.078 |
| CPIJ015728 | conserved hypothetical protein | DIV |  | -1.671 | -1.547 |
| CPIJ003342 | ceramide kinase | DIV |  |  | -1.507 |
| CPIJ008352 | conserved hypothetical protein | DIV | -0.82 | -1.811 | -1.662 |
| CPIJ014102 | synaptic vesicle protein | DIV, TRP |  | -1.235 | -0.957 |
| CPIJ016452 | acyl-coa dehydrogenase | DIV, MET |  | -1.071 | -1.63 |
| CPIJ014673 | conserved hypothetical protein | DIV, TRP |  | -1.324 | -1.671 |
| CPIJ006472 | myo-inositol-1 phosphate synthase | DIV |  | -0.907 | -1.792 |
| CPIJ004019 | saccharopine dehydrogenase domain-containing protein | DIV | -2.782 | -3.965 | -2.314 |
| CPIJ010885 | rhythmically expressed gene 2 protein | DIV |  | -2.004 | -1.719 |
| CPIJ017659 | conserved hypothetical protein | DIV |  | -0.802 | -1.7 |
| CPIJ012034 | L-allo-threonine aldolase | DIV |  |  | -1.641 |
| CPIJ001774 | synaptic vesicle protein | DIV |  | -1.571 | -1.531 |
| CPIJ001964 | Juvenile hormone-inducible protein | DIV |  | -1.974 | -2.038 |
| CPIJ002402 | conserved hypothetical protein | DIV |  | -0.851 | -1.597 |
| CPIJ019364 | apolipoprotein D | DIV, TRP |  | -1.036 | -1.852 |
| CPIJ004020 | saccharopine dehydrogenase domain-containing protein | DIV | -0.884 | -1.885 | -1.857 |
| CPIJ007525 | conserved hypothetical protein | DIV |  | -1.964 | -1.768 |
| CPIJ007516 | thiamine transporter 1 | DIV | -1.226 | -1.52 | -1.981 |
| CPIJ002256 | spermine synthase | DIV | -1.274 | -2.48 | -1.653 |
| CPIJ015808 | beta-hexosaminidase | DIV |  | -1.083 | -1.815 |
| CPIJ016662 | leucine aminopeptidase 1 | DIV |  | -1.832 | -1.943 |
| CPIJ009984 | methylmalonate-semialdehyde dehydrogenase, mitochondrial | DIV, MET |  | -1.654 | -1.602 |
| CPIJ010151 | dihydropyrimidinase | DIV | -1.284 | -1.658 | -1.645 |
| CPIJ011198 | threonine dehydratase/deaminase | DIV |  | -1.22 | -1.512 |
| CPIJ011542 | synaptic vesicle protein | DIV, TRP | -0.888 | -1.511 | -1.32 |
| CPIJ002338 | conserved hypothetical protein | DIV |  | -2.063 | -1.829 |
| CPIJ014057 | conserved hypothetical protein | DIV |  | -2.453 | -1.603 |
| CPIJ007954 | pter-prov protein | DIV |  | -0.859 | -1.493 |
| CPIJ011388 | diazepam binding inhibitor | DIV | -0.811 | -1.964 | -1.774 |
| CPIJ006409 | alanine-glyoxylate aminotransferase | DIV | 0.775 |  | -0.816 |
| CPIJ008529 | lactase-phlorizin hydrolase | DIV |  |  | -1.913 |
| CPIJ004455 | sulfite reductase | DIV | -1.06 | -1.747 | -1.687 |
| CPIJ013881 | conserved hypothetical protein | DIV | -1.286 | -2.048 | -1.843 |
| CPIJ007907 | conserved hypothetical protein | DIV |  |  | -2.513 |
| CPIJ006676 | conserved hypothetical protein | DIV |  |  | -1.535 |
| CPIJ009459 | conserved hypothetical protein | DIV |  | -1.552 | -2.411 |
| CPIJ003391 | aldehyde reductase 1 | DIV, RSM |  | -2.069 | -1.959 |
| CPIJ018121 | membrane-bound alkaline phosphatase | DIV | -0.769 | -2.636 | -2.149 |
| CPIJ006031 | conserved hypothetical protein | DIV | -1.072 | -1.656 | -1.766 |
| CPIJ002322 | conserved hypothetical protein | DIV | -2.046 | -1.96 | -2.549 |
| CPIJ011185 | deoxyribonuclease I | DIV |  |  | -2.255 |
| CPIJ003393 | aldose reductase | DIV, RSM | -1.59 | -2.601 | -1.982 |
| CPIJ014226 | cellular retinaldehyde-binding protein | DIV |  |  | -1.855 |
| CPIJ015060 | conserved hypothetical protein | DIV | -1.77 | -2.263 | -2.036 |
| CPIJ020105 | apolipoprotein D | DIV, TRP |  | -1.074 | -2.142 |
| CPIJ007313 | myotubularin | DIV |  | -1.605 | -1.968 |
| CPIJ003840 | aliphatic nitrilase | DIV |  | -2.084 | -1.971 |
| CPIJ017624 | glyoxylase | DIV | -0.886 | -1.954 | -2.101 |
| CPIJ001264 | alkaline phosphatase | DIV | -1.905 | -2.658 | -2.274 |
| CPIJ013905 | serine-type enodpeptidase | DIV, IMM |  |  | -1.827 |
| CPIJ000277 | conserved hypothetical protein | DIV | -1.328 | -2.041 | -2.238 |
| CPIJ006076 | hypodermin-B | DIV, PRT, MET, IMM, DIG, TRP | 1.398 |  | -2.109 |
| CPIJ003006 | conserved hypothetical protein | DIV |  |  | -2.362 |
| CPIJ007125 | regucalcin | DIV |  | -1.821 | -1.788 |
| CPIJ013600 | mandelate racemase | DIV |  | -2.284 | -2.198 |
| CPIJ003338 | beta-galactosidase | DIV |  | -1.43 | -2.543 |
| CPIJ018757 | gh regulated tbc protein-1 | DIV |  | -0.887 | -2.066 |
| CPIJ013737 | conserved hypothetical protein | DIV | -1.076 | -2.194 | -2.346 |
| CPIJ018991 | serine-type enodpeptidase | DIV, PRT, IMM, MET, DIG, TRP |  |  | -1.908 |
| CPIJ006542 | chymotrypsin-2 | DIV, PRT, MET, IMM, DIG, TRP |  | -2.216 | -2.465 |
| CPIJ001265 | alkaline phosphatase | DIV | -2.128 | -1.845 | -2.186 |
| CPIJ004173 | Juvenile hormone-inducible protein | DIV |  | -1.803 | -1.889 |
| CPIJ009744 | conserved hypothetical protein | DIV | -1.165 | -2.347 | -2.591 |
| CPIJ010181 | conserved hypothetical protein | DIV |  | -2.156 | -2.718 |
| CPIJ001576 | peroxisomal multifunctional enzyme type 2 | DIV, RSM |  | -2.038 | -2.599 |
| CPIJ004088 | guanylyl cyclase receptor | DIV | -1.271 | -2.327 | -2.939 |
| CPIJ007628 | Juvenile hormone-inducible protein | DIV | -3.006 | -3.889 | -2.727 |
| CPIJ014558 | nitrilase and fragile histidine triad fusion protein NitFhit | DIV, MET |  | -0.788 | -2.126 |
| CPIJ007230 | regucalcin | DIV |  | -1.483 | -2.459 |
| CPIJ013577 | selenium-binding protein 2 | DIV | -1.303 | -3.304 | -2.651 |
| CPIJ008531 | lactase-phlorizin hydrolase | DIV | -0.891 | -2.444 | -3.052 |
| CPIJ002835 | serine-pyruvate aminotransferase | DIV | -2.265 | -2.852 | -3.553 |
| CPIJ012990 | allantoicase | DIV | -1.398 | -2.917 | -3.082 |
| CPIJ006077 | trypsin theta | DIV, PRT, MET, IMM, DIG, TRP |  | -1.382 | -2.733 |
| CPIJ008530 | lactase-phlorizin hydrolase | DIV |  | -1.838 | -3.335 |
| CPIJ015241 | alkaline phosphatase | DIV | -1.809 | -3.601 | -3.781 |
| CPIJ005975 | conserved hypothetical protein | DIV |  | -1.585 | -2.711 |
| CPIJ008109 | conserved hypothetical protein | DIV |  | -1.894 | -3.446 |
| CPIJ004400 | ornithine aminotransferase, mitochondrial | DIV | -3.48 | -3.516 | -3.131 |
| CPIJ003807 | allantoinase | DIV | -2.263 | -3.305 | -3.922 |
| CPIJ006617 | conserved hypothetical protein | DIV | -2.913 | -3.786 | -4.282 |
| CPIJ001746 | conserved hypothetical protein | DIV, TRP |  | -2.923 | -4.133 |
| CPIJ008571 | UDP-glucuronosyltransferase | DIV | 3.077 | 2.918 | 2.211 |
| CPIJ017857 | ATPase WRNIP1 | DIV | 1.204 | 1.082 | 0.814 |
| CPIJ015931 | regulator of g protein signaling | DIV | 1.717 | 1.398 | 0.757 |
| CPIJ019184 | conserved hypothetical protein | DIV | 1.046 | 1.231 | 0.848 |
| CPIJ001300 | zinc finger protein 780B | DIV | 1.131 | 1.143 | 0.915 |
| CPIJ003586 | proteasome subunit alpha type 3 | DIV | -1.088 | -1.716 | -0.841 |
| CPIJ004454 | conserved hypothetical protein | DIV | -1.163 | -0.875 | 0.754 |
| CPIJ007659 | conserved hypothetical protein | DIV | 1.495 | 1.523 | 0.817 |
| CPIJ007747 | T01G9.2 | DIV | 1.711 | 1.826 | 0.962 |
| CPIJ010893 | proteasome subunit alpha type 4 | DIV | -1.017 | -1.952 | -0.851 |
| CPIJ016927 | conserved hypothetical protein | DIV | 1.443 | 1.33 | 0.79 |
| CPIJ011177 | GPI mannosyltransferase 1 | DIV | -0.914 | -1.788 | -0.962 |
| CPIJ013950 | conserved hypothetical protein | DIV | 0.771 | 1.203 | 1.145 |
| CPIJ014532 | 2-oxoisovalerate dehydrogenase subunit beta, mitochondrial | DIV | -0.895 | -1.509 | -0.855 |
| CPIJ015587 | oligosaccharyl transferase | DIV | -1.005 | -1.438 | -0.793 |
| CPIJ005468 | adenosine diphosphatase | DIV | -1.224 | -0.835 | -0.782 |
| CPIJ004924 | LRR Toll | DIV | -0.992 | -1.096 | -1.307 |
| CPIJ005258 | conserved hypothetical protein | DIV | -1.097 | -0.816 | -0.792 |
| CPIJ008106 | conserved hypothetical protein | DIV | 2.362 | 0.778 | -0.931 |
| CPIJ008512 | conserved hypothetical protein | DIV | -0.913 | -1.373 | -0.873 |
| CPIJ009050 | lkb1 interacting protein | DIV | 0.92 | 0.903 | 0.861 |
| CPIJ011711 | conserved hypothetical protein | DIV | -0.817 | -1.022 | -0.948 |
| CPIJ016908 | conserved hypothetical protein | DIV | -0.954 | -0.832 | -0.874 |
| CPIJ017966 | conserved hypothetical protein | DIV | 0.881 | 0.771 | 0.841 |
| CPIJ019557 | conserved hypothetical protein | DIV | -0.756 | -0.807 | -0.781 |
| CPIJ002525 | XPA-binding protein 1 | DIV |  | 0.875 | 0.846 |
| CPIJ013382 | serine/threonine-protein kinase RIO2 | DIV |  | 1.086 | 0.881 |
| CPIJ008105 | conserved hypothetical protein | DIV |  | -0.928 | -0.789 |
| CPIJ001707 | proteasome subunit alpha type 6 | DIV |  | -1.4 | -0.766 |
| CPIJ007734 | conserved hypothetical protein | DIV |  | -1.187 | -0.856 |
| CPIJ010031 | carnitine O-palmitoyltransferase 2, mitochondrial | DIV |  | -1.059 | -0.772 |
| CPIJ016903 | signal peptidase complex subunit 3 | DIV |  | -1.391 | -0.862 |
| CPIJ001379 | conserved hypothetical protein | DIV |  | 1.195 | 0.921 |
| CPIJ005222 | alanine-glyoxylate aminotransferase | DIV |  | -1.278 | -0.828 |
| CPIJ008108 | conserved hypothetical protein | DIV |  | -1.039 | -0.937 |
| CPIJ011190 | deoxyribonuclease I | DIV |  | 1.441 | 1.121 |
| CPIJ013562 | alanine-glyoxylate aminotransferase | DIV |  | -1.577 | -1.027 |
| CPIJ015040 | conserved hypothetical protein | DIV |  | -0.834 | -0.779 |
| CPIJ015418 | conserved hypothetical protein | DIV |  | -1.065 | -0.776 |
| CPIJ018588 | conserved hypothetical protein | DIV | 1.14 |  | 0.782 |
| CPIJ015255 | conserved hypothetical protein | DIV | 0.97 |  | 0.88 |
| CPIJ004208 | sulfakinin | DIV | 1.259 |  | 0.757 |
| CPIJ005777 | synaptic vesicle protein | DIV | -0.873 |  | 1.394 |
| CPIJ007311 | conserved hypothetical protein | DIV | 1.305 |  | -0.828 |
| CPIJ010452 | H/ACA ribonucleoprotein complex subunit 3 | DIV | -1.553 |  | 0.79 |
| CPIJ000509 | adenosine kinase | DIV | -0.884 |  | 0.805 |
| CPIJ005892 | conserved hypothetical protein | DIV | 1.024 |  | 0.886 |
| CPIJ009929 | conserved hypothetical protein | DIV | 1.002 |  | -0.809 |
| CPIJ010832 | conserved hypothetical protein | DIV | 0.883 |  | 1.114 |
| CPIJ019874 | salivary cysteine-rich peptide | DIV |  |  | -1.439 |
| CPIJ014423 | conserved hypothetical protein | DIV |  |  | 0.933 |
| CPIJ014194 | conserved hypothetical protein | DIV |  |  | 0.978 |
| CPIJ004593 | conserved hypothetical protein | DIV |  |  | 1.012 |
| CPIJ009299 | four and a half lim domains | DIV |  |  | -0.767 |
| CPIJ009370 | acylphosphatase | DIV |  |  | -0.793 |
| CPIJ001579 | UDP-glucose 4-epimerase | DIV |  |  | -0.86 |
| CPIJ000309 | sam/hd domain protein | DIV |  |  | 1.105 |
| CPIJ000980 | diaphanous | DIV |  |  | 0.785 |
| CPIJ001349 | conserved hypothetical protein | DIV |  |  | -0.765 |
| CPIJ001495 | rab5 | DIV |  |  | 0.786 |
| CPIJ002885 | conserved hypothetical protein | DIV |  |  | 0.769 |
| CPIJ003879 | lipid storage droplets surface-binding protein 1 | DIV |  |  | -0.857 |
| CPIJ003916 | conserved hypothetical protein | DIV |  |  | 0.817 |
| CPIJ004606 | phd finger domain | DIV |  |  | 0.883 |
| CPIJ005219 | disulfide isomerase | DIV |  |  | -0.796 |
| CPIJ005820 | geranylgeranyl transferase type-2 alpha subunit | DIV |  |  | 0.844 |
| CPIJ006389 | abl interactor 2 | DIV |  |  | 0.768 |
| CPIJ006885 | porcupine | DIV |  |  | 0.761 |
| CPIJ006920 | conserved hypothetical protein | DIV |  |  | 0.971 |
| CPIJ007246 | heme oxygenase 1 | DIV |  |  | 0.933 |
| CPIJ007880 | conserved hypothetical protein | DIV |  |  | -0.853 |
| CPIJ008185 | phosphatidylinositol-phosphatidylcholine transfer protein | DIV |  |  | 0.872 |
| CPIJ008649 | OV-16 antigen | DIV |  |  | -0.755 |
| CPIJ008856 | conserved hypothetical protein | DIV |  |  | 0.859 |
| CPIJ009356 | dynamin-associated protein | DIV |  |  | 0.79 |
| CPIJ009731 | glucosidase 2 subunit beta | DIV |  |  | -0.756 |
| CPIJ010601 | N-terminal acetyltransferase A complex catalytic subunit ARD1 | DIV |  |  | 0.824 |
| CPIJ011334 | nuclear lamin L1 alpha | DIV |  |  | 0.781 |
| CPIJ011910 | conserved hypothetical protein | DIV |  |  | -0.791 |
| CPIJ013401 | mical | DIV |  |  | 0.781 |
| CPIJ016477 | hypothetical protein | DIV |  |  | -0.879 |
| CPIJ016574 | geranylgeranyl transferase type-1 subunit beta | DIV |  |  | -0.888 |
| CPIJ017176 | hypothetical protein | DIV |  |  | 0.788 |
| CPIJ019901 | myo inositol monophosphatase | DIV |  |  | -0.822 |
| CPIJ004547 | lariat debranching enzyme | DIV | 1.155 | 0.92 |  |
| CPIJ009611 | synaptic vesicle glycoprotein 2B | DIV | 2.459 | 2.152 |  |
| CPIJ010510 | WD repeat protein 59 | DIV | 1.58 | 1.457 |  |
| CPIJ010990 | pacifastin light chain | DIV | -1.821 | -2.34 |  |
| CPIJ011003 | cytosolic sulfotransferase 2 | DIV | -1.693 | -1.426 |  |
| CPIJ011605 | conserved hypothetical protein | DIV | 1.743 | 1.875 |  |
| CPIJ014577 | phosphoglycerate mutase 2 | DIV | -1.872 | -2.316 |  |
| CPIJ003765 | fanconi anemia group J protein | DIV | 0.803 | 0.873 |  |
| CPIJ007528 | anterior fat body protein | DIV | -1.736 | -2.312 |  |
| CPIJ001551 | RING finger protein 10 | DIV | 1.662 | 1.41 |  |
| CPIJ003351 | 26S proteasome non-ATPase regulatory subunit 6 | DIV | -1.064 | -1.171 |  |
| CPIJ008660 | ets domain-containing protein | DIV | 1.586 | 1.205 |  |
| CPIJ018673 | succinyl-CoA ligase subunit alpha | DIV | -1.196 | -1.598 |  |
| CPIJ002025 | conserved hypothetical protein | DIV | 2.698 | 1.702 |  |
| CPIJ004301 | endoplasmic oxidoreductin-1 | DIV | -1.08 | -1.761 |  |
| CPIJ005860 | conserved hypothetical protein | DIV | 0.832 | 0.859 |  |
| CPIJ011525 | conserved hypothetical protein | DIV | 0.925 | 0.922 |  |
| CPIJ006405 | sushi | DIV | 0.89 | 1.537 |  |
| CPIJ008180 | conserved hypothetical protein | DIV | -0.886 | -1.049 |  |
| CPIJ013217 | glutamate semialdehyde dehydrogenase | DIV | 1.125 | 1.298 |  |
| CPIJ015956 | glycine N-methyltransferase | DIV | -0.891 | -1.283 |  |
| CPIJ000116 | conserved hypothetical protein | DIV | -1.335 | -0.942 |  |
| CPIJ000640 | Tsc1 | DIV | 0.942 | 0.986 |  |
| CPIJ000897 | proteasome subunit alpha type 1 | DIV | -1.445 | -1.732 |  |
| CPIJ001373 | NAD-dependent deacetylase sirtuin-4 | DIV | -0.935 | -1.06 |  |
| CPIJ001505 | conserved hypothetical protein | DIV | -1.66 | -1.814 |  |
| CPIJ002043 | translocon-associated protein, delta subunit | DIV | -1.798 | -1.665 |  |
| CPIJ002169 | adenine phosphoribosyltransferase | DIV | -1.13 | -1.227 |  |
| CPIJ002306 | GTPase-activating protein ZNF289 | DIV | -1.07 | -0.925 |  |
| CPIJ002850 | cgi-51-prov protein | DIV | -0.923 | -1.202 |  |
| CPIJ003122 | zinc finger protein | DIV | 0.944 | 0.953 |  |
| CPIJ003512 | defender against cell death 1 | DIV | -1.237 | -1.363 |  |
| CPIJ003659 | peptidyl-tRNA hydrolase 2, mitochondrial | DIV | -1.247 | -0.869 |  |
| CPIJ004201 | mevalonate kinase | DIV | -1.107 | -1.351 |  |
| CPIJ004582 | mitochondrial ribosomal protein S17 | DIV | -1.259 | -1.486 |  |
| CPIJ004671 | ATP synthase mitochondrial F1 complex assembly factor 2 | DIV | -0.933 | -1.188 |  |
| CPIJ004679 | bile acid beta-glucosidase | DIV | -2.056 | -1.589 |  |
| CPIJ004773 | partner of sld5 | DIV | -2.147 | -1.575 |  |
| CPIJ004778 | ER protein reticulon | DIV | -2.107 | -2.051 |  |
| CPIJ005136 | 26S protease regulatory subunit 6B | DIV | -1.013 | -0.911 |  |
| CPIJ005137 | Sec61 protein complex gamma subunit | DIV | -1.355 | -1.321 |  |
| CPIJ005149 | ubiquitin-conjugating enzyme E2 i | DIV | -0.981 | -1.099 |  |
| CPIJ005313 | DNA replication complex GINS protein PSF2 | DIV | -1.395 | -0.966 |  |
| CPIJ005483 | conserved hypothetical protein | DIV | -1.091 | -1.106 |  |
| CPIJ005501 | mmr1/hsr1 GTP binding protein | DIV | 0.946 | 1.062 |  |
| CPIJ005611 | tumor suppressor candidate 3 | DIV | -0.86 | -1.491 |  |
| CPIJ006096 | membrin | DIV | -1.106 | -0.868 |  |
| CPIJ006246 | conserved hypothetical protein | DIV | -1.333 | -1.406 |  |
| CPIJ006531 | NSFL1 cofactor p47 | DIV | -0.811 | -0.877 |  |
| CPIJ006881 | basic FGF-repressed Zic-binding protein | DIV | -1.146 | -1.293 |  |
| CPIJ007051 | immature colon carcinoma | DIV | -0.792 | -0.795 |  |
| CPIJ007098 | ubiquitin-fold modifier 1 | DIV | -1.121 | -1.142 |  |
| CPIJ007310 | conserved hypothetical protein | DIV | 1.291 | 1.205 |  |
| CPIJ007327 | disulfide-isomerase A6 | DIV | -1.362 | -1.351 |  |
| CPIJ007464 | conserved hypothetical protein | DIV | -0.776 | -1.166 |  |
| CPIJ007514 | ATPase family AAA domain-containing protein 3 | DIV | -1.768 | -2.706 |  |
| CPIJ007517 | kinase C-binding protein 1 | DIV | 1.083 | 1.297 |  |
| CPIJ007692 | 26S proteasome non-ATPase regulatory subunit 14 | DIV | -0.89 | -1.146 |  |
| CPIJ007693 | conserved hypothetical protein | DIV | -1.415 | -1.797 |  |
| CPIJ008191 | grpE | DIV | -1.385 | -1.642 |  |
| CPIJ008278 | huntingtin-interacting protein HYPK | DIV | -0.951 | -1.009 |  |
| CPIJ008679 | oligosaccharyl transferase | DIV | -1.029 | -1.674 |  |
| CPIJ008701 | conserved hypothetical protein | DIV | -1.43 | -1.488 |  |
| CPIJ008889 | 60 kDa heat shock protein, mitochondrial | DIV | -1.889 | -1.456 |  |
| CPIJ009046 | proliferating cell nuclear antigen | DIV | -1.249 | -0.853 |  |
| CPIJ009164 | calnexin | DIV | -0.89 | -0.778 |  |
| CPIJ009330 | conserved hypothetical protein | DIV | -1.918 | -2.26 |  |
| CPIJ009416 | ubiquitin-activating enzyme E1 domain-containing protein 1 | DIV | -1.163 | -1.237 |  |
| CPIJ009862 | conserved hypothetical protein | DIV | 1.272 | 1.188 |  |
| CPIJ009935 | mannosyl-oligosaccharide alpha-1,2-mannosidase | DIV | -0.906 | -0.934 |  |
| CPIJ009942 | density-regulated protein | DIV | -1.168 | -1.18 |  |
| CPIJ010114 | proteasome subunit alpha type | DIV | -0.899 | -1.115 |  |
| CPIJ010318 | conserved hypothetical protein | DIV | -1.613 | -1.118 |  |
| CPIJ011052 | conserved hypothetical protein | DIV | -1.104 | -0.982 |  |
| CPIJ011205 | 26S proteasome non-ATPase regulatory subunit 8 | DIV | -0.867 | -0.845 |  |
| CPIJ011727 | translocon-associated protein subunit gamma | DIV | -1.435 | -2 |  |
| CPIJ011908 | traf2 and nck interacting kinase, tnik | DIV | 1.1 | 0.937 |  |
| CPIJ011915 | multisynthetase complex, auxiliary protein, p38 | DIV | -1.276 | -0.763 |  |
| CPIJ012186 | hypothetical protein | DIV | -1.243 | -1.627 |  |
| CPIJ012218 | rho GTPase | DIV | 1.981 | 1.719 |  |
| CPIJ012633 | conserved hypothetical protein | DIV | 1.005 | 1.119 |  |
| CPIJ012670 | nucleotide exchange factor SIL1 | DIV | -1.086 | -0.999 |  |
| CPIJ013335 | ctg4a | DIV | -1.116 | -1.558 |  |
| CPIJ013982 | conserved hypothetical protein | DIV | 0.849 | 0.927 |  |
| CPIJ014098 | s-adenosyl-methyl transferase mraw | DIV | -1.312 | -1.093 |  |
| CPIJ014133 | saposin | DIV | 1.106 | 1.024 |  |
| CPIJ014505 | conserved hypothetical protein | DIV | -0.996 | -1.076 |  |
| CPIJ015322 | beta-1,4-galactosyltransferase 7 | DIV | -0.993 | -1.316 |  |
| CPIJ015372 | rhomboid protein 1, mitochondrial | DIV | -1.032 | -0.824 |  |
| CPIJ015673 | conserved hypothetical protein | DIV | -1.199 | -1.354 |  |
| CPIJ015971 | 26S proteasome non-ATPase regulatory subunit 11 | DIV | -0.887 | -1.037 |  |
| CPIJ017381 | piwi | DIV | 0.966 | 0.79 |  |
| CPIJ017596 | synaptic vesicle glycoprotein 2A | DIV | -1.696 | -2.893 |  |
| CPIJ017989 | truncated ER mannose-binding lectin | DIV | -1.089 | -1.523 |  |
| CPIJ018234 | translocon-associated protein subunit alpha | DIV | -1.313 | -1.516 |  |
| CPIJ018512 | cell division cycle and apoptosis regulator protein 1 | DIV | -0.961 | -1.199 |  |
| CPIJ000597 | conserved hypothetical protein | DIV | 0.927 | 1.079 |  |
| CPIJ000772 | zinc finger protein 189 | DIV | 0.791 | 0.826 |  |
| CPIJ001661 | conserved hypothetical protein | DIV | 0.855 | 1.136 |  |
| CPIJ001699 | dolichyl pyrophosphate Glc1Man9GlcNAc2 alpha-1,3-glucosyltransferase | DIV | -1.14 | -1.29 |  |
| CPIJ002086 | acylglycerol kinase, mitochondrial | DIV | -0.753 | -0.921 |  |
| CPIJ002834 | translocation associated membrane protein | DIV | -0.933 | -1.614 |  |
| CPIJ003312 | conserved hypothetical protein | DIV | 1.011 | 0.877 |  |
| CPIJ003331 | conserved hypothetical protein | DIV | -0.931 | -0.83 |  |
| CPIJ003735 | conserved hypothetical protein | DIV | -0.774 | -1.182 |  |
| CPIJ003753 | methionyl-tRNA formyltransferase | DIV | -0.894 | -1.268 |  |
| CPIJ004033 | headcase | DIV | 1.268 | 0.778 |  |
| CPIJ004814 | proteasome maturation protein | DIV | -1.104 | -1.278 |  |
| CPIJ004838 | 26S proteasome non-ATPase regulatory subunit 3 | DIV | -0.898 | -0.9 |  |
| CPIJ005338 | PRUNE protein | DIV | -0.99 | -1.413 |  |
| CPIJ006457 | phenylalanyl-tRNA synthetase, mitochondrial | DIV | -0.804 | -1.44 |  |
| CPIJ006564 | ubiquinone biosynthesis protein | DIV | -0.928 | -1.483 |  |
| CPIJ006877 | dcapl | DIV | 0.832 | 0.882 |  |
| CPIJ006880 | conserved hypothetical protein | DIV | -0.926 | -0.849 |  |
| CPIJ007179 | proteasome subunit alpha type 7-1 | DIV | -0.933 | -1.38 |  |
| CPIJ007912 | tuberous sclerosis complex 2 | DIV | 0.753 | 0.808 |  |
| CPIJ007951 | leucyl-tRNA synthetase | DIV | -0.768 | -1.061 |  |
| CPIJ008289 | pyruvate kinase | DIV | -0.853 | -1.337 |  |
| CPIJ010319 | Juvenile hormone-inducible protein | DIV | -0.913 | -1.57 |  |
| CPIJ010816 | mesoderm development candidate 2 | DIV | -0.871 | -0.946 |  |
| CPIJ011261 | cell cycle control protein cwf8 | DIV | -1.091 | -0.917 |  |
| CPIJ013357 | conserved hypothetical protein | DIV | -1.32 | -0.838 |  |
| CPIJ013481 | translation machinery-associated protein 20 | DIV | -0.804 | -1.11 |  |
| CPIJ013530 | conserved hypothetical protein | DIV | -1.027 | -1.241 |  |
| CPIJ013722 | dimethylaniline monooxygenase 1 | DIV | 0.806 | 0.811 |  |
| CPIJ014896 | conserved hypothetical protein | DIV | -0.996 | -1.238 |  |
| CPIJ017247 | microsomal signal peptidase 18 kDa subunit | DIV | -0.771 | -1.386 |  |
| CPIJ017298 | monocyte to macrophage differentiation factor 2 | DIV | -0.89 | -1.028 |  |
| CPIJ017927 | basic FGF-repressed Zic-binding protein | DIV | -1.163 | -1.248 |  |
| CPIJ018147 | kazal domain-containing peptide | DIV | 0.898 | 1.46 |  |
| CPIJ019831 | conserved hypothetical protein | DIV | -1.01 | -1.601 |  |
| CPIJ019881 | ubiquinone biosynthesis protein coq-4 | DIV | -0.821 | -1.155 |  |
| CPIJ002390 | dihydrofolate reductase | DIV | -1.116 | -0.804 |  |
| CPIJ003198 | neuropathy target esterase/swiss cheese | DIV | 1.178 | 0.962 |  |
| CPIJ004776 | glycogen phosphorylase | DIV | -1.413 | -1.633 |  |
| CPIJ004872 | phosphatase 2C beta | DIV | -1.224 | -0.854 |  |
| CPIJ008199 | conserved hypothetical protein | DIV | -0.845 | -0.973 |  |
| CPIJ011690 | Psf3 | DIV | -1.264 | -1.062 |  |
| CPIJ013962 | sumo-1-activating enzyme E1a | DIV | -1.564 | -1.396 |  |
| CPIJ014565 | conserved hypothetical protein | DIV | -1.009 | -1.358 |  |
| CPIJ015055 | 26S proteasome non-ATPase regulatory subunit 7 | DIV | -1.258 | -0.92 |  |
| CPIJ015192 | conserved hypothetical protein | DIV | 0.871 | 0.888 |  |
| CPIJ016043 | autocrine motility factor receptor | DIV | 0.763 | 0.794 |  |
| CPIJ016357 | conserved hypothetical protein | DIV | -1.059 | -1.194 |  |
| CPIJ018591 | calcyclin-binding protein | DIV | -0.857 | -1.251 |  |
| CPIJ019405 | p15-2a protein | DIV | -1.413 | -0.992 |  |
| CPIJ000027 | semaphorin | DIV | 0.963 | 0.832 |  |
| CPIJ000414 | conserved hypothetical protein | DIV | 2.246 | 0.992 |  |
| CPIJ000885 | conserved hypothetical protein | DIV | -0.763 | -0.772 |  |
| CPIJ000970 | conserved hypothetical protein | DIV | -0.937 | 1.293 |  |
| CPIJ001278 | conserved hypothetical protein | DIV | 0.781 | 0.778 |  |
| CPIJ001396 | conserved hypothetical protein | DIV | 0.758 | 1.074 |  |
| CPIJ001479 | conserved hypothetical protein | DIV | -0.959 | -0.904 |  |
| CPIJ001578 | conserved hypothetical protein | DIV | -0.758 | -0.814 |  |
| CPIJ002052 | WD repeat protein 61 | DIV | -1.059 | -1.214 |  |
| CPIJ003570 | conserved hypothetical protein | DIV | -0.942 | -0.788 |  |
| CPIJ003653 | myoneurin | DIV | 0.942 | 0.918 |  |
| CPIJ003819 | rabenosyn-5 | DIV | 1.036 | 0.899 |  |
| CPIJ003835 | conserved hypothetical protein | DIV | -0.772 | -1.086 |  |
| CPIJ004589 | neuronal calcium sensor | DIV | 1.199 | 1.506 |  |
| CPIJ005047 | zinc finger protein 517 | DIV | 0.775 | 1.009 |  |
| CPIJ005329 | peroxisomal carnitine O-octanoyltransferase | DIV | -0.991 | -0.91 |  |
| CPIJ005530 | conserved hypothetical protein | DIV | 0.86 | 1.763 |  |
| CPIJ005717 | conserved hypothetical protein | DIV | 1.194 | 0.796 |  |
| CPIJ005718 | dual specificity protein phosphatase 7 | DIV | 1.347 | 0.789 |  |
| CPIJ006379 | neuronal pas domain protein | DIV | -0.915 | -0.982 |  |
| CPIJ006574 | conserved hypothetical protein | DIV | 1.059 | 0.938 |  |
| CPIJ006667 | glutamate receptor | DIV | 0.856 | 1.059 |  |
| CPIJ006933 | conserved hypothetical protein | DIV | -0.903 | -0.985 |  |
| CPIJ007480 | transmembrane emp24 domain-containing protein 9 | DIV | -0.774 | -0.772 |  |
| CPIJ008573 | conserved hypothetical protein | DIV | -0.934 | -1.14 |  |
| CPIJ008998 | clk2 | DIV | 0.755 | 0.837 |  |
| CPIJ009091 | conserved hypothetical protein | DIV | 0.869 | 0.886 |  |
| CPIJ009670 | anon-35F/36A | DIV | -0.811 | -0.768 |  |
| CPIJ009730 | calmodulin | DIV | 1.006 | 0.882 |  |
| CPIJ009913 | conserved hypothetical protein | DIV | 0.928 | 0.849 |  |
| CPIJ010176 | conserved hypothetical protein | DIV | -1.066 | -1.187 |  |
| CPIJ010416 | prefoldin subunit 1 | DIV | -0.852 | -0.791 |  |
| CPIJ010685 | anaphase-promoting complex subunit 10 | DIV | -0.841 | -0.797 |  |
| CPIJ010734 | sex-determining protein fem-1 | DIV | 1.679 | 0.999 |  |
| CPIJ010939 | conserved hypothetical protein | DIV | 0.897 | 0.896 |  |
| CPIJ011462 | translation machinery-associated protein 20 | DIV | -0.851 | -1.145 |  |
| CPIJ011517 | Tes39 | DIV | -1.075 | -0.772 |  |
| CPIJ011920 | conserved hypothetical protein | DIV | 0.836 | 1.112 |  |
| CPIJ012012 | conserved hypothetical protein | DIV | -0.962 | 0.771 |  |
| CPIJ012357 | multicopper oxidase | DIV | -1.29 | -0.964 |  |
| CPIJ012564 | helix-loop-helix transcription factor | DIV | 0.861 | 0.782 |  |
| CPIJ012749 | conserved hypothetical protein | DIV | 0.809 | 1.108 |  |
| CPIJ012964 | autophagy protein 9 | DIV | 1.16 | 1.08 |  |
| CPIJ013126 | cysteine-rich hydrophobic domain 2 | DIV | 0.852 | 1.133 |  |
| CPIJ013344 | 26S protease regulatory subunit 7 | DIV | -0.855 | -0.994 |  |
| CPIJ013651 | conserved hypothetical protein | DIV | 0.776 | 1.197 |  |
| CPIJ014867 | parkin | DIV | 0.859 | 0.804 |  |
| CPIJ014870 | pre-mRNA cleavage factor im, 25kD subunit | DIV | -1.006 | -0.963 |  |
| CPIJ015441 | fas apoptotic inhibitory molecule 2 | DIV | -0.978 | -1.071 |  |
| CPIJ016934 | neuronal calcium sensor | DIV | 0.909 | 0.771 |  |
| CPIJ017223 | zinc finger protein 542 | DIV | 1.761 | 1 |  |
| CPIJ018309 | tyrosine-protein kinase | DIV | 0.799 | 1.333 |  |
| CPIJ018758 | conserved hypothetical protein | DIV | 0.818 | 1.013 |  |
| CPIJ019010 | FAM116B | DIV | 0.893 | 0.876 |  |
| CPIJ019675 | conserved hypothetical protein | DIV | 1.023 | 1.169 |  |
| CPIJ008365 | RAS | DIV |  | 1.054 |  |
| CPIJ000469 | aq_284 | DIV |  | 0.794 |  |
| CPIJ001254 | nucleolar GTP-binding protein 2 | DIV |  | 1.231 |  |
| CPIJ001621 | molybdenum cofactor synthesis protein 3 | DIV |  | 0.757 |  |
| CPIJ001916 | arginine N-methyltransferase skb1 | DIV |  | 0.797 |  |
| CPIJ004969 | pescadillo | DIV |  | 1.224 |  |
| CPIJ005364 | nucleolar GTP-binding protein 2 | DIV |  | 0.801 |  |
| CPIJ006677 | conserved hypothetical protein | DIV |  | 1.968 |  |
| CPIJ010514 | bystin | DIV |  | 0.944 |  |
| CPIJ000773 | conserved hypothetical protein | DIV |  | -0.851 |  |
| CPIJ010913 | pita | DIV |  | 0.879 |  |
| CPIJ005762 | zinc finger protein 593 | DIV |  | 0.891 |  |
| CPIJ013548 | syntaxin binding protein-1,2,3 | DIV |  | -0.818 |  |
| CPIJ009396 | inhibitor of growth protein 3 | DIV |  | 0.839 |  |
| CPIJ018015 | prefoldin | DIV |  | -0.866 |  |
| CPIJ000275 | poly polymerase 16 | DIV |  | -1.471 |  |
| CPIJ000693 | ubiquinone/menaquinone biosynthesis methyltransferase ubiE | DIV |  | -0.898 |  |
| CPIJ000944 | deoxyribonuclease I | DIV |  | -1.389 |  |
| CPIJ001550 | mystery 45A | DIV |  | 0.933 |  |
| CPIJ002330 | prefoldin | DIV |  | -1.238 |  |
| CPIJ002829 | arsenite-resistance protein | DIV |  | -1.14 |  |
| CPIJ003009 | conserved hypothetical protein | DIV |  | 1.096 |  |
| CPIJ003356 | cell cycle progression | DIV |  | -1.18 |  |
| CPIJ003385 | alkaline nuclease | DIV |  | 1.347 |  |
| CPIJ003390 | trichohyalin | DIV |  | 0.947 |  |
| CPIJ003988 | prostaglandin E synthase 2 | DIV |  | -0.912 |  |
| CPIJ004552 | conserved hypothetical protein | DIV |  | -0.945 |  |
| CPIJ004991 | peptidyl-prolyl cis-trans isomerase | DIV |  | -1.142 |  |
| CPIJ005020 | conserved hypothetical protein | DIV |  | -1.206 |  |
| CPIJ005767 | protoporphyrinogen oxidase | DIV |  | -0.848 |  |
| CPIJ005783 | signal peptidase complex subunit 1 | DIV |  | -1.025 |  |
| CPIJ005915 | aspartyl-tRNA synthetase, mitochondrial | DIV |  | -1.219 |  |
| CPIJ006492 | apolipoprotein D | DIV |  | 0.948 |  |
| CPIJ007172 | conserved hypothetical protein | DIV |  | 0.906 |  |
| CPIJ007484 | UDP-glucose:glycoprotein glucosyltransferase | DIV |  | -1.016 |  |
| CPIJ008201 | kynurenine-oxoglutarate transaminase 1 | DIV |  | -1.252 |  |
| CPIJ008212 | phosphatase-1 | DIV |  | 0.877 |  |
| CPIJ008535 | conserved hypothetical protein | DIV |  | 0.884 |  |
| CPIJ009240 | ganglioside-induced differentiation-associated protein 1 | DIV |  | -0.762 |  |
| CPIJ009747 | GPI mannosyltransferase 1 | DIV |  | -0.936 |  |
| CPIJ010292 | vacuolar protein sorting-associated protein 25 | DIV |  | -0.907 |  |
| CPIJ010513 | secretory carrier-associated membrane protein | DIV |  | 1.123 |  |
| CPIJ011345 | phosphatidylinositolglycan class N | DIV |  | -0.85 |  |
| CPIJ012528 | derlin-2.1 | DIV |  | -0.76 |  |
| CPIJ013079 | conserved hypothetical protein | DIV |  | 1.029 |  |
| CPIJ013499 | zinc finger and SCAN domain-containing protein 21 | DIV |  | 0.763 |  |
| CPIJ014467 | conserved hypothetical protein | DIV |  | -0.923 |  |
| CPIJ015361 | conserved hypothetical protein | DIV |  | -1.161 |  |
| CPIJ015524 | NULL | DIV |  | -0.97 |  |
| CPIJ015560 | nudix hydrolase 3 | DIV |  | -0.798 |  |
| CPIJ015981 | hormone-sensitive lipase | DIV |  | -0.824 |  |
| CPIJ016407 | 26S protease regulatory subunit 6A | DIV |  | -1.12 |  |
| CPIJ016541 | conserved hypothetical protein | DIV |  | -1.093 |  |
| CPIJ016635 | liprin alpha | DIV |  | -0.782 |  |
| CPIJ016640 | inositol monophosphatase | DIV |  | -0.958 |  |
| CPIJ017566 | synaptic vesicle protein | DIV |  | -1.488 |  |
| CPIJ018524 | prostaglandin E synthase 2 | DIV |  | -1.002 |  |
| CPIJ019493 | fibroblast growth factor receptor 1 | DIV |  | -1.25 |  |
| CPIJ018340 | ezrindixin-moesin-binding phosphoprotein 50 | DIV |  | 0.798 |  |
| CPIJ000019 | ANON-66Db protein | DIV |  | -0.877 |  |
| CPIJ000289 | conserved hypothetical protein | DIV |  | 0.868 |  |
| CPIJ000506 | conserved hypothetical protein | DIV |  | -0.776 |  |
| CPIJ000824 | conserved hypothetical protein | DIV |  | 0.919 |  |
| CPIJ001043 | palmitoyl-protein thioesterase 1 | DIV |  | 0.868 |  |
| CPIJ001155 | cell division protein kinase 2 | DIV |  | -0.884 |  |
| CPIJ001271 | conserved hypothetical protein | DIV |  | 0.916 |  |
| CPIJ001273 | conserved hypothetical protein | DIV |  | 1.026 |  |
| CPIJ001302 | ryanodine receptor | DIV |  | 0.796 |  |
| CPIJ001397 | synaptotagmin | DIV |  | 0.911 |  |
| CPIJ001501 | conserved hypothetical protein | DIV |  | 0.921 |  |
| CPIJ001573 | conserved hypothetical protein | DIV |  | 0.756 |  |
| CPIJ002238 | hypothetical protein | DIV |  | 0.918 |  |
| CPIJ002301 | trf-proximal protein | DIV |  | -1.207 |  |
| CPIJ002452 | ubiquitin thioesterase OTUB1 | DIV |  | -0.81 |  |
| CPIJ002648 | tyrosine phosphatase, non-receptor type nt6 | DIV |  | 0.797 |  |
| CPIJ002702 | adenylosuccinate lyase | DIV |  | -0.925 |  |
| CPIJ002906 | conserved hypothetical protein | DIV |  | -1.078 |  |
| CPIJ002949 | mago nashi | DIV |  | -0.839 |  |
| CPIJ003089 | SCO1, mitochondrial | DIV |  | -0.882 |  |
| CPIJ003124 | olygosaccharyltransferase alpha subunit | DIV |  | -0.848 |  |
| CPIJ003337 | beta-galactosidase | DIV |  | -0.773 |  |
| CPIJ003406 | nucleoside diphosphate kinase | DIV |  | -0.766 |  |
| CPIJ003419 | conserved hypothetical protein | DIV |  | 0.848 |  |
| CPIJ003725 | adenylosuccinate synthetase | DIV |  | 1.239 |  |
| CPIJ003805 | cyclic-AMP response element binding protein | DIV |  | 0.753 |  |
| CPIJ003896 | exosome component 10 | DIV |  | 0.791 |  |
| CPIJ004008 | fimbrin/plastin | DIV |  | 0.798 |  |
| CPIJ004047 | glutamyl-tRNA | DIV |  | -0.814 |  |
| CPIJ004098 | charged multivesicular body protein 3 | DIV |  | 0.751 |  |
| CPIJ004354 | conserved hypothetical protein | DIV |  | -0.886 |  |
| CPIJ004399 | conserved hypothetical protein | DIV |  | -0.817 |  |
| CPIJ004529 | ubiquitin-conjugating enzyme E2-25kD | DIV |  | -0.859 |  |
| CPIJ004538 | conserved hypothetical protein | DIV |  | 0.77 |  |
| CPIJ004594 | conserved hypothetical protein | DIV |  | 0.995 |  |
| CPIJ004833 | smad | DIV |  | 0.996 |  |
| CPIJ005084 | coiled-coil domain-containing protein 124 | DIV |  | -0.754 |  |
| CPIJ005104 | conserved hypothetical protein | DIV |  | 0.917 |  |
| CPIJ005144 | plakophilin-4 | DIV |  | 0.858 |  |
| CPIJ005235 | Juvenile hormone-inducible protein | DIV |  | -0.786 |  |
| CPIJ005261 | COP9 signalosome complex subunit 8 | DIV |  | -0.84 |  |
| CPIJ005472 | leukocyte receptor cluster member 4 protein | DIV |  | -1.009 |  |
| CPIJ005597 | conserved hypothetical protein | DIV |  | -1.436 |  |
| CPIJ005708 | conserved hypothetical protein | DIV |  | 0.844 |  |
| CPIJ005752 | 77 kDa echinoderm microtubule-associated protein | DIV |  | 0.789 |  |
| CPIJ005771 | bolA | DIV |  | -0.815 |  |
| CPIJ006192 | conserved hypothetical protein | DIV |  | 0.84 |  |
| CPIJ006259 | conserved hypothetical protein | DIV |  | -0.781 |  |
| CPIJ006378 | trachealess | DIV |  | -0.809 |  |
| CPIJ006391 | plexin-A2 | DIV |  | 0.799 |  |
| CPIJ006932 | ebna2 binding protein P100 | DIV |  | -0.753 |  |
| CPIJ007131 | mrp | DIV |  | -1.099 |  |
| CPIJ007264 | conserved hypothetical protein | DIV |  | -0.815 |  |
| CPIJ007458 | tyrosine-protein kinase src64b | DIV |  | 0.95 |  |
| CPIJ007533 | conserved hypothetical protein | DIV |  | 0.884 |  |
| CPIJ007700 | ubiquitin-activating enzyme E1 | DIV |  | -0.787 |  |
| CPIJ007713 | G protein-coupled receptor | DIV |  | 1.11 |  |
| CPIJ007733 | conserved hypothetical protein | DIV |  | 0.925 |  |
| CPIJ008316 | puff-specific protein Bx42 | DIV |  | 0.781 |  |
| CPIJ008383 | conserved hypothetical protein | DIV |  | -0.771 |  |
| CPIJ008392 | N-acetyltransferase 5 | DIV |  | -0.784 |  |
| CPIJ008481 | ADP ribosylation factor | DIV |  | 0.993 |  |
| CPIJ008621 | conserved hypothetical protein | DIV |  | 1.105 |  |
| CPIJ008872 | conserved hypothetical protein | DIV |  | 0.782 |  |
| CPIJ009217 | roundabout 1 | DIV |  | -0.81 |  |
| CPIJ009460 | conserved hypothetical protein | DIV |  | -0.758 |  |
| CPIJ009571 | fructose-bisphosphate aldolase | DIV |  | -0.759 |  |
| CPIJ009653 | conserved hypothetical protein | DIV |  | -0.825 |  |
| CPIJ009699 | conserved hypothetical protein | DIV |  | -0.764 |  |
| CPIJ009805 | conserved hypothetical protein | DIV |  | -0.936 |  |
| CPIJ009927 | chaperone protein | DIV |  | -1.142 |  |
| CPIJ009931 | 26S protease regulatory subunit 8 | DIV |  | -0.865 |  |
| CPIJ010166 | conserved hypothetical protein | DIV |  | -1.039 |  |
| CPIJ010194 | ras-related protein | DIV |  | 0.949 |  |
| CPIJ010239 | phosphatase 2c | DIV |  | 0.782 |  |
| CPIJ010440 | hypothetical protein | DIV |  | -0.906 |  |
| CPIJ010447 | conserved hypothetical protein | DIV |  | 1.099 |  |
| CPIJ010462 | thyroid receptor interacting protein | DIV |  | 0.857 |  |
| CPIJ010831 | fatty acyl-CoA reductase 1 | DIV |  | -1.297 |  |
| CPIJ010860 | cat eye syndrome critical region protein 1 | DIV |  | -0.787 |  |
| CPIJ011090 | transmembrane protein 63A | DIV |  | 1.048 |  |
| CPIJ011494 | ADP-ribose pyrophosphatase, mitochondrial | DIV |  | -0.816 |  |
| CPIJ011552 | 26S protease regulatory subunit 4 | DIV |  | -0.956 |  |
| CPIJ011557 | nucleoporin, p88 | DIV |  | -0.781 |  |
| CPIJ011559 | calcitonin receptor | DIV |  | 0.798 |  |
| CPIJ011562 | 14.5 kDa salivary peptide | DIV |  | 0.816 |  |
| CPIJ011625 | conserved hypothetical protein | DIV |  | -0.864 |  |
| CPIJ011954 | claudin 12 | DIV |  | -1.225 |  |
| CPIJ011990 | conserved hypothetical protein | DIV |  | 0.906 |  |
| CPIJ012072 | lymnokinin receptor | DIV |  | -0.826 |  |
| CPIJ012178 | ovary C/EBPg transcription factor | DIV |  | 0.813 |  |
| CPIJ012460 | conserved hypothetical protein | DIV |  | -0.77 |  |
| CPIJ012499 | conserved hypothetical protein | DIV |  | 0.856 |  |
| CPIJ012657 | wd-repeat protein | DIV |  | 0.908 |  |
| CPIJ012836 | leucine-rich transmembrane protein | DIV |  | 0.841 |  |
| CPIJ012930 | conserved hypothetical protein | DIV |  | 0.879 |  |
| CPIJ013328 | cysteine N-palmitoyltransferase Rasp | DIV |  | -0.866 |  |
| CPIJ013329 | peptidyl-prolyl cis-trans isomerase NIMA-interacting 4 | DIV |  | -1.018 |  |
| CPIJ013529 | dihydrofolate reductase | DIV |  | -0.759 |  |
| CPIJ013574 | conserved hypothetical protein | DIV |  | -0.898 |  |
| CPIJ013652 | conserved hypothetical protein | DIV |  | 0.771 |  |
| CPIJ013704 | conserved hypothetical protein | DIV |  | 0.931 |  |
| CPIJ013989 | synaptic ras GTPase activating protein | DIV |  | 0.998 |  |
| CPIJ014041 | conserved hypothetical protein | DIV |  | -0.765 |  |
| CPIJ014417 | dihydrofolate reductase | DIV |  | -0.806 |  |
| CPIJ014556 | conserved hypothetical protein | DIV |  | -0.759 |  |
| CPIJ014824 | conserved hypothetical protein | DIV |  | 0.774 |  |
| CPIJ015121 | autophagy-specific gene 2 | DIV |  | 1.358 |  |
| CPIJ015188 | conserved hypothetical protein | DIV |  | 0.886 |  |
| CPIJ015718 | arginase | DIV |  | -1.811 |  |
| CPIJ015768 | pre-mRNA-processing factor 17 | DIV |  | 0.781 |  |
| CPIJ015789 | uridine phosphorylase | DIV |  | 0.929 |  |
| CPIJ016079 | zinc finger protein | DIV |  | 1.06 |  |
| CPIJ016524 | conserved hypothetical protein | DIV |  | 0.758 |  |
| CPIJ017505 | tyrosine sulfotransferase | DIV |  | -0.803 |  |
| CPIJ017882 | conserved hypothetical protein | DIV |  | -1.03 |  |
| CPIJ018130 | proteasome regulatory subu | DIV |  | -0.841 |  |
| CPIJ018298 | tryrosine phosphatase | DIV |  | 1.048 |  |
| CPIJ018592 | nucleoporin | DIV |  | 0.987 |  |
| CPIJ018922 | GTP-binding protein | DIV |  | 0.787 |  |
| CPIJ019106 | guanine-nucleotide exchange factor c3g | DIV |  | 0.862 |  |
| CPIJ019386 | conserved hypothetical protein | DIV |  | 0.843 |  |
| CPIJ019408 | conserved hypothetical protein | DIV |  | 0.889 |  |
| CPIJ019469 | disulfide-isomerase A5 | DIV |  | -0.863 |  |
| CPIJ019708 | Juvenile hormone-inducible protein | DIV |  | -0.867 |  |
| CPIJ019712 | checkpoint protein HUS1 | DIV |  | -1.095 |  |
| CPIJ020113 | leucine-rich transmembrane protein | DIV |  | 1.037 |  |
| CPIJ000760 | conserved hypothetical protein | DIV | 0.776 |  |  |
| CPIJ004440 | conserved hypothetical protein | DIV | -1.312 |  |  |
| CPIJ006752 | conserved hypothetical protein | DIV | 1.181 |  |  |
| CPIJ003302 | RNA-binding protein 34 | DIV | -0.838 |  |  |
| CPIJ006911 | conserved hypothetical protein | DIV | 0.797 |  |  |
| CPIJ000119 | kelch domain-containing protein 4 | DIV | -1.567 |  |  |
| CPIJ008245 | conserved hypothetical protein | DIV | 0.755 |  |  |
| CPIJ014186 | conserved hypothetical protein | DIV | 0.824 |  |  |
| CPIJ007681 | frizzled | DIV | 1.338 |  |  |
| CPIJ008292 | conserved hypothetical protein | DIV | 0.785 |  |  |
| CPIJ011204 | DNA-repair protein XRCC1 | DIV | 0.76 |  |  |
| CPIJ004068 | suppressor of T-cell receptor signaling 1 | DIV | -1.05 |  |  |
| CPIJ012882 | argininosuccinate synthase | DIV | -0.892 |  |  |
| CPIJ004239 | ADP-ribosylation factor 1 | DIV | -0.892 |  |  |
| CPIJ008583 | DnaJ domain containing protein | DIV | -1.121 |  |  |
| CPIJ009364 | anamorsin | DIV | -1.02 |  |  |
| CPIJ011320 | Ufm1-conjugating enzyme 1 | DIV | -0.855 |  |  |
| CPIJ012308 | tetratricopeptide repeat protein 35 | DIV | -0.843 |  |  |
| CPIJ018084 | uty-prov protein | DIV | -0.994 |  |  |
| CPIJ001188 | conserved hypothetical protein | DIV | 1.019 |  |  |
| CPIJ003296 | transport protein SEC13 | DIV | -0.869 |  |  |
| CPIJ003757 | tyrosine phosphatase mitochondrial 1 | DIV | -0.785 |  |  |
| CPIJ005490 | prolactin regulatory element-binding protein | DIV | -0.869 |  |  |
| CPIJ006599 | mannosyl-oligosaccharide alpha-1,2-mannosidase | DIV | -0.94 |  |  |
| CPIJ017393 | conserved hypothetical protein | DIV | -0.757 |  |  |
| CPIJ019681 | conserved hypothetical protein | DIV | 0.797 |  |  |
| CPIJ000795 | H/ACA ribonucleoprotein complex subunit 4 | DIV | -1.92 |  |  |
| CPIJ003437 | conserved hypothetical protein | DIV | 1.225 |  |  |
| CPIJ003580 | cle7 | DIV | -1.008 |  |  |
| CPIJ004309 | nucleolar essential protein 1 | DIV | -1.6 |  |  |
| CPIJ005255 | conserved hypothetical protein | DIV | 1.217 |  |  |
| CPIJ005347 | 26S proteasome non-ATPase regulatory subunit 13 | DIV | -0.877 |  |  |
| CPIJ005675 | NBP2b protein | DIV | -1.34 |  |  |
| CPIJ006466 | H/ACA ribonucleoprotein complex subunit 4 | DIV | -1.212 |  |  |
| CPIJ007306 | conserved hypothetical protein | DIV | 0.769 |  |  |
| CPIJ008258 | alsin | DIV | 0.939 |  |  |
| CPIJ010589 | conserved hypothetical protein | DIV | -1.074 |  |  |
| CPIJ012872 | conserved hypothetical protein | DIV | 0.758 |  |  |
| CPIJ012888 | rRNA processing protein Ebp2 | DIV | -0.971 |  |  |
| CPIJ013086 | BRCA2 and CDKN1A-interacting protein | DIV | -1.137 |  |  |
| CPIJ013094 | phosphoglycerate mutase | DIV | 2.58 |  |  |
| CPIJ015473 | max binding protein | DIV | 0.999 |  |  |
| CPIJ018353 | 26S proteasome non-ATPase regulatory subunit 7 | DIV | -0.815 |  |  |
| CPIJ018833 | H/ACA ribonucleoprotein complex subunit 3 | DIV | -1.07 |  |  |
| CPIJ000388 | ubiquitin conjugating enzyme 7 interacting protein | DIV | 0.787 |  |  |
| CPIJ000413 | conserved hypothetical protein | DIV | 1.238 |  |  |
| CPIJ000497 | conserved hypothetical protein | DIV | 0.752 |  |  |
| CPIJ000510 | conserved hypothetical protein | DIV | 0.89 |  |  |
| CPIJ000635 | zinc finger protein CKR1 | DIV | 0.77 |  |  |
| CPIJ000667 | nucleoporin | DIV | -0.809 |  |  |
| CPIJ000697 | conserved hypothetical protein | DIV | 0.847 |  |  |
| CPIJ000827 | elbow | DIV | 0.951 |  |  |
| CPIJ000856 | conserved hypothetical protein | DIV | -0.76 |  |  |
| CPIJ001189 | conserved hypothetical protein | DIV | 0.935 |  |  |
| CPIJ001284 | conserved hypothetical protein | DIV | -0.876 |  |  |
| CPIJ001929 | GABA-A receptor interacting factor-1 | DIV | 0.775 |  |  |
| CPIJ002411 | BCDIN3 domain containing | DIV | -0.772 |  |  |
| CPIJ002812 | guanyl-nucleotide exchange factor | DIV | 0.785 |  |  |
| CPIJ003587 | toys are us | DIV | -0.796 |  |  |
| CPIJ003682 | hypoxia-inducible factor | DIV | -0.853 |  |  |
| CPIJ003811 | conserved hypothetical protein | DIV | -0.846 |  |  |
| CPIJ003887 | conserved hypothetical protein | DIV | -0.933 |  |  |
| CPIJ003986 | multiprotein bridging factor | DIV | -0.962 |  |  |
| CPIJ003997 | nuclear pore complex protein Nup107 | DIV | -1.167 |  |  |
| CPIJ004151 | nuclear hormone receptor FTZ-F1 beta | DIV | 0.764 |  |  |
| CPIJ004251 | myelin proteolipid | DIV | 1.015 |  |  |
| CPIJ004356 | conserved hypothetical protein | DIV | 1.362 |  |  |
| CPIJ004358 | blastoderm specific protein 25d | DIV | -0.847 |  |  |
| CPIJ004522 | 26S proteasome non-ATPase regulatory subunit 4 | DIV | -0.785 |  |  |
| CPIJ005016 | origin recognition complex subunit 6 | DIV | -1.099 |  |  |
| CPIJ005106 | conserved hypothetical protein | DIV | 1.051 |  |  |
| CPIJ005437 | conserved hypothetical protein | DIV | -0.947 |  |  |
| CPIJ005439 | nucleoporin | DIV | -0.858 |  |  |
| CPIJ005500 | zinc finger protein 182 | DIV | 1.116 |  |  |
| CPIJ005720 | adenosine kinase 2 | DIV | -0.777 |  |  |
| CPIJ006208 | conserved hypothetical protein | DIV | -0.77 |  |  |
| CPIJ006488 | Nup75 | DIV | -0.913 |  |  |
| CPIJ006581 | conserved hypothetical protein | DIV | 1.262 |  |  |
| CPIJ006879 | tyrosine-protein kinase pr2 | DIV | 0.916 |  |  |
| CPIJ006936 | conserved hypothetical protein | DIV | 0.896 |  |  |
| CPIJ007345 | glutamate synthase | DIV | -1.144 |  |  |
| CPIJ007421 | conserved hypothetical protein | DIV | 0.781 |  |  |
| CPIJ007427 | conserved hypothetical protein | DIV | 0.963 |  |  |
| CPIJ007504 | bumetanide-sensitive sodium- | DIV | -1.397 |  |  |
| CPIJ007511 | conserved hypothetical protein | DIV | -1.003 |  |  |
| CPIJ007560 | phosphatase and actin regulator | DIV | 0.783 |  |  |
| CPIJ007866 | conserved hypothetical protein | DIV | 0.86 |  |  |
| CPIJ008256 | threonine dehydrogenase | DIV | -0.876 |  |  |
| CPIJ008288 | HD domain-containing protein 3 | DIV | -0.846 |  |  |
| CPIJ008466 | conserved hypothetical protein | DIV | 1.073 |  |  |
| CPIJ008927 | conserved hypothetical protein | DIV | -0.765 |  |  |
| CPIJ009239 | lingerer | DIV | -0.763 |  |  |
| CPIJ009253 | cle7 | DIV | -0.926 |  |  |
| CPIJ009262 | d-3-phosphoglycerate dehydrogenase | DIV | -1.15 |  |  |
| CPIJ009620 | serine/threonine-protein phosphatase 5 | DIV | -0.836 |  |  |
| CPIJ009907 | nucleoporin | DIV | -0.857 |  |  |
| CPIJ009933 | adenylate kinase isoenzyme 6 | DIV | -0.899 |  |  |
| CPIJ010040 | CaSm | DIV | -0.77 |  |  |
| CPIJ011079 | discoidin domain receptor | DIV | 0.863 |  |  |
| CPIJ011444 | tRNA methyltransferase | DIV | -0.845 |  |  |
| CPIJ011585 | glutamate-cysteine ligase | DIV | -1.232 |  |  |
| CPIJ011687 | inosine-5'-monophosphate dehydrogenase | DIV | -1.075 |  |  |
| CPIJ011708 | vacuolar protein sorting | DIV | 0.771 |  |  |
| CPIJ011793 | conserved hypothetical protein | DIV | -0.776 |  |  |
| CPIJ012944 | FAD-NAD binding oxidoreductase | DIV | 1.229 |  |  |
| CPIJ013686 | signal transduction protein lnk-realted | DIV | 0.975 |  |  |
| CPIJ014018 | knolle | DIV | -0.963 |  |  |
| CPIJ014050 | predicted protein | DIV | -1.15 |  |  |
| CPIJ014179 | conserved hypothetical protein | DIV | -1.027 |  |  |
| CPIJ014382 | fibronectin type-III domain-containing protein 3a | DIV | 0.811 |  |  |
| CPIJ014536 | coronin | DIV | 0.846 |  |  |
| CPIJ014720 | ets DNA-binding protein pokkuri | DIV | 1.104 |  |  |
| CPIJ014736 | nucleoporin | DIV | -0.804 |  |  |
| CPIJ014872 | nucleoporin | DIV | -0.853 |  |  |
| CPIJ015212 | aspartyl-tRNA synthetase | DIV | -0.896 |  |  |
| CPIJ015812 | calcium-binding protein E63-1 | DIV | 1.086 |  |  |
| CPIJ016165 | ubiquilin-1 | DIV | -0.935 |  |  |
| CPIJ016253 | transport and Golgi organization | DIV | -0.865 |  |  |
| CPIJ016789 | zinc finger protein | DIV | -0.853 |  |  |
| CPIJ017222 | gastrula zinc finger protein | DIV | 1.023 |  |  |
| CPIJ017478 | conserved hypothetical protein | DIV | 1.065 |  |  |
| CPIJ018157 | exosome complex exonuclease RRP46 | DIV | -0.825 |  |  |
| CPIJ018607 | conserved hypothetical protein | DIV | 1.122 |  |  |
| CPIJ018620 | conserved hypothetical protein | DIV | 0.782 |  |  |
| CPIJ018946 | conserved hypothetical protein | DIV | -0.774 |  |  |
| CPIJ019322 | SET | DIV | -0.771 |  |  |
| CPIJ019832 | Cdk5 and Abl enzyme substrate 1 | DIV | 0.794 |  |  |
| CPIJ008520 | major sperm protein | DIV, CST |  |  | -0.783 |
| CPIJ003700 | supervillin | DIV, CST | 0.765 | 0.834 |  |
| CPIJ000677 | cytoplasmic dynein intermediate chain, | DIV, CST | 0.848 |  |  |
| CPIJ012156 | trypsin-1 | DIV, DIG, PRT | 0.761 | 1.146 |  |
| CPIJ003637 | ecsit | DIV, IMM |  |  | 0.806 |
| CPIJ010401 | phosphatase 1 regulatory subunit 7 | DIV, IMM |  |  | 0.837 |
| CPIJ006993 | lysine 6-oxidase | DIV, IMM |  | -0.929 |  |
| CPIJ013534 | yellow | DIV, IMM |  | -1 |  |
| CPIJ011427 | conserved hypothetical protein | DIV, IMM | 0.83 |  |  |
| CPIJ011834 | venom allergen 3 | DIV, MET | -2.941 | -3.189 |  |
| CPIJ003309 | succinate semialdehyde dehydrogenase, mitochondrial | DIV, MET |  | -1.052 |  |
| CPIJ008591 | 6-phosphofructokinase | DIV, MET |  | -0.757 |  |
| CPIJ012293 | venom allergen 5 | DIV, MET | 1.563 |  |  |
| CPIJ007385 | serine-type enodpeptidase | DIV, MET, PRT, IMM, DIG | -3.048 | -3.381 | -1.202 |
| CPIJ010899 | conserved hypothetical protein | DIV, PRT |  | 0.972 |  |
| CPIJ013739 | conserved hypothetical protein | DIV, PRT |  | -1.025 |  |
| CPIJ000835 | chymotrypsin-2 | DIV, PRT, DIG, MET |  | 0.799 | -0.962 |
| CPIJ007384 | azurocidin | DIV, PRT, IMM, MET, DIG, TRP | -1.786 | -2.294 | -1.451 |
| CPIJ006075 | trypsin beta | DIV, PRT, MET, DIG | 0.961 | 0.841 |  |
| CPIJ007080 | trypsin-1 | DIV, PRT, MET, IMM, DIG, TRP |  |  | 0.762 |
| CPIJ009607 | malic enzyme | DIV, RSM | 1.74 | 1.596 | 0.77 |
| CPIJ012341 | malic enzyme | DIV, RSM | -0.901 | -1.239 |  |
| CPIJ008100 | conserved hypothetical protein | DIV, RSM | -1.893 | -1.634 |  |
| CPIJ008825 | conserved hypothetical protein | DIV, RSM | -1.102 | -1.221 |  |
| CPIJ015373 | S-formylglutathione hydrolase | DIV, RSM | -0.89 | -1.082 |  |
| CPIJ000873 | thioredoxin domain containing 14 | DIV, RSM |  | -0.969 |  |
| CPIJ018127 | conserved hypothetical protein | DIV, RTT | 0.761 | 0.998 | 0.787 |
| CPIJ018107 | zinc finger protein | DIV, RTT | 0.813 | 0.78 |  |
| CPIJ003748 | nucleolar protein 10 | DIV, RTT |  | 0.947 |  |
| CPIJ019677 | RNA-binding protein pno1 | DIV, RTT |  | 0.865 |  |
| CPIJ018619 | Srp54 | DIV, RTT |  | 0.951 |  |
| CPIJ001487 | polyubiquitin | DIV, RTT |  | -0.779 |  |
| CPIJ015891 | ischemia/reperfusion inducible protein | DIV, RTT |  | 1.131 |  |
| CPIJ005033 | nucleolar protein NHP2 | DIV, RTT | -1.836 |  |  |
| CPIJ009807 | bhlhzip transcription factor bigmax | DIV, RTT | 1.129 |  |  |
| CPIJ020318 | conserved hypothetical protein | DIV, RTT | 0.829 |  |  |
| CPIJ005653 | developmentally regulated RNA-binding protein | DIV, RTT | -0.825 |  |  |
| CPIJ010569 | conserved hypothetical protein | DIV, RTT | -0.754 |  |  |
| CPIJ015549 | ribosomal biogenesis protein Gar2 | DIV, RTT | -0.791 |  |  |
| CPIJ000779 | equilibrative nucleoside transporter | DIV, TRP | 1.29 | 0.931 |  |
| CPIJ005417 | synaptic vesicle glycoprotein 2C | DIV, TRP | 1.206 |  |  |
| CPIJ005333 | transport protein sec23 | DIV, TRP | -0.754 |  |  |
| CPIJ008314 | sideroflexin 1,2,3 | DIV, TRP | -0.877 |  |  |
| CPIJ009191 | retinal degeneration b beta | DIV, TRP | -0.758 |  |  |
| CPIJ000150 | conserved hypothetical protein | UNK | -2.196 | -2.267 | -1.864 |
| CPIJ001035 | conserved hypothetical protein | UNK | -2.414 | -3.633 | -2.771 |
| CPIJ001281 | conserved hypothetical protein | UNK | 1.456 | 2.074 | 1.056 |
| CPIJ002468 | embryonic polarity dorsal | UNK | 1.502 | 1.395 | 0.958 |
| CPIJ003381 | SERAC1 | UNK | 2.979 | 4.609 | 3.727 |
| CPIJ003787 | conserved hypothetical protein | UNK | 2.754 | 2.715 | 1.683 |
| CPIJ004602 | serine 3-dehydrogenase | UNK | -2.749 | -3.333 | -2.873 |
| CPIJ004733 | predicted protein | UNK | -2.856 | -2.802 | -2.177 |
| CPIJ007283 | conserved hypothetical protein | UNK | 1.073 | 1.568 | 0.993 |
| CPIJ007760 | hypothetical protein | UNK | 1.223 | 1.6 | 1.629 |
| CPIJ007966 | conserved hypothetical protein | UNK | 1.086 | 1 | 0.98 |
| CPIJ009371 | conserved hypothetical protein | UNK | -2.692 | -3.614 | -3.116 |
| CPIJ010315 | Juvenile hormone-inducible protein | UNK | -3.058 | -4.095 | -3.314 |
| CPIJ010320 | conserved hypothetical protein | UNK | -1.702 | -2.632 | -2.18 |
| CPIJ010828 | conserved hypothetical protein | UNK | -2.351 | -2.013 | -1.233 |
| CPIJ011376 | hypothetical protein | UNK | 1.663 | 1.943 | 1.176 |
| CPIJ012036 | aminopeptidase N | UNK | -1.046 | -1.726 | -2.12 |
| CPIJ012069 | sucrose transport protein | UNK | -2.191 | -2.569 | -2.939 |
| CPIJ014224 | conserved hypothetical protein | UNK | -3.684 | -5.054 | -4.904 |
| CPIJ015534 | ER lumen protein retaining receptor | UNK | 2.204 | 2.18 | 1.829 |
| CPIJ016920 | NADP-dependent L-serine/L-allo-threonine dehydrogenase ydfG | UNK | -2.139 | -2.67 | -2.809 |
| CPIJ017188 | conserved hypothetical protein | UNK | -2.094 | -2.103 | -0.916 |
| CPIJ018567 | hypothetical protein | UNK | 1.992 | 2.458 | 2.92 |
| CPIJ019628 | conserved hypothetical protein | UNK | 0.918 | 1.538 | 0.992 |
| CPIJ020028 | multicopper oxidase | UNK | -1.944 | -1.486 | -1.282 |
| CPIJ020309 | hypothetical protein | UNK | 0.996 | 1.353 | 0.884 |
| CPIJ002628 | sensory appendage protein | UNK | 1.88 | 1.666 | 1.808 |
| CPIJ003366 | conserved hypothetical protein | UNK | 0.952 | 2.085 | 1.824 |
| CPIJ004283 | conserved hypothetical protein | UNK | -1.112 | -2.831 | -2.328 |
| CPIJ004815 | uricase | UNK | -2.317 | -2.457 | -2.492 |
| CPIJ008859 | conserved hypothetical protein | UNK | -2.262 | -3.331 | -2.312 |
| CPIJ011512 | conserved hypothetical protein | UNK | -0.931 | -0.784 | -1.264 |
| CPIJ012802 | conserved hypothetical protein | UNK | 1.364 | 1.556 | 1.26 |
| CPIJ012907 | luciferin 4-monooxygenase | UNK | -0.839 | -3.579 | -3.295 |
| CPIJ015296 | retinol-binding protein | UNK | -1.166 | -2.498 | -2.059 |
| CPIJ018629 | glutathione-s-transferase theta, gst | UNK | -1.523 | -2.665 | -2.455 |
| CPIJ018314 | conserved hypothetical protein | UNK | -1.157 | -1.192 | -1.735 |
| CPIJ000494 | conserved hypothetical protein | UNK | 2.218 | 2.464 | 1.149 |
| CPIJ000495 | conserved hypothetical protein | UNK | 1.981 | 3.25 | 2.016 |
| CPIJ004603 | oxidoreductase | UNK | -1.131 | -2.124 | -2.422 |
| CPIJ009726 | conserved hypothetical protein | UNK | 1.223 | 2.493 | 1.999 |
| CPIJ010324 | conserved hypothetical protein | UNK | -1.435 | -2.513 | -1.673 |
| CPIJ004346 | conserved hypothetical protein | UNK | 1.108 | 1.606 | 1.575 |
| CPIJ006693 | conserved hypothetical protein | UNK | -1.337 | -1.996 | -1.57 |
| CPIJ012374 | conserved hypothetical protein | UNK | 1.112 | 1.753 | 1.236 |
| CPIJ015669 | AMP dependent ligase | UNK | -1.737 | -2.148 | -1.843 |
| CPIJ020237 | conserved hypothetical protein | UNK | 1.088 | 1.834 | 1.951 |
| CPIJ002675 | glutathione S-transferase 1 | UNK | -1.38 | -2.479 | -2.372 |
| CPIJ012507 | conserved hypothetical protein | UNK | 1.326 | 1.36 | 0.756 |
| CPIJ000061 | conserved hypothetical protein | UNK | -2.145 | -2.28 | -1.133 |
| CPIJ002777 | conserved hypothetical protein | UNK | 1.209 | 1.625 | 1.592 |
| CPIJ005157 | hypothetical protein | UNK | 2.519 | 2.206 | 1.882 |
| CPIJ007206 | conserved hypothetical protein | UNK | 1.21 | 1.122 | 0.862 |
| CPIJ008976 | 4-nitrophenylphosphatase | UNK | -2.972 | -1.466 | 0.828 |
| CPIJ010311 | conserved hypothetical protein | UNK | -1.691 | -2.654 | -0.95 |
| CPIJ010490 | conserved hypothetical protein | UNK | 0.793 | 1.22 | 1.012 |
| CPIJ010903 | conserved hypothetical protein | UNK | -1.373 | -1.738 | -1.22 |
| CPIJ011964 | conserved hypothetical protein | UNK | 0.97 | 1.299 | 1.153 |
| CPIJ013009 | c3f | UNK | 1.397 | 1.199 | 1.065 |
| CPIJ017829 | predicted protein | UNK | -1.489 | -1.628 | 0.94 |
| CPIJ000531 | hypothetical protein | UNK | 2.498 | 4.289 | 4.062 |
| CPIJ001052 | aminopeptidase 2, mitochondrial | UNK | -1.604 | -3.234 | -2.874 |
| CPIJ001996 | conserved hypothetical protein | UNK | 1.215 | 1.614 | 1.153 |
| CPIJ014496 | conserved hypothetical protein | UNK | 2.768 | 3.142 | 3.573 |
| CPIJ019227 | pancreatic triacylglycerol lipase | UNK | -0.854 | 1.547 | 1.712 |
| CPIJ006120 | microfibril-associated glycoprotein 4 | UNK | 1.83 | 2.072 | 2.7 |
| CPIJ011414 | conserved hypothetical protein | UNK | 1.142 | 1.297 | 1.369 |
| CPIJ011663 | conserved hypothetical protein | UNK | 1.577 | 1.608 | 1.508 |
| CPIJ017460 | luciferin 4-monooxygenase | UNK | 0.844 | 2.041 | 2.006 |
| CPIJ018717 | conserved hypothetical protein | UNK | 1.591 | 2.526 | 3.106 |
| CPIJ000496 | conserved hypothetical protein | UNK | 2.097 | 2.172 | 1.379 |
| CPIJ000559 | conserved hypothetical protein | UNK | -0.847 | -2.127 | -1.638 |
| CPIJ002091 | conserved hypothetical protein | UNK | 1.01 | 1.671 | 1.399 |
| CPIJ003708 | conserved hypothetical protein | UNK | 1.43 | 1.675 | 1.023 |
| CPIJ005087 | cell wall cysteine-rich protein | UNK | -1.758 | -1.488 | 1.239 |
| CPIJ007196 | conserved hypothetical protein | UNK | -1.155 | -0.888 | 1.214 |
| CPIJ008097 | conserved hypothetical protein | UNK | -1.657 | -2.128 | -0.802 |
| CPIJ008582 | hypothetical protein | UNK | -1.907 | -1.668 | -1.149 |
| CPIJ008809 | hypothetical protein | UNK | -3.234 | -1.51 | -1.634 |
| CPIJ009505 | conserved hypothetical protein | UNK | 1.389 | 1.611 | 1.102 |
| CPIJ014493 | transient receptor potential cation channel protein painless | UNK | 1.859 | 3.358 | 1.768 |
| CPIJ015243 | hypothetical protein | UNK | -1.249 | 1.636 | 4.297 |
| CPIJ015920 | conserved hypothetical protein | UNK | -1.358 | -2.807 | -1.329 |
| CPIJ016195 | hypothetical protein | UNK | -3.589 | -1.942 | -1.77 |
| CPIJ016491 | 4-coumarate-CoA ligase 1 | UNK | -1.843 | -2.528 | -1.218 |
| CPIJ016767 | conserved hypothetical protein | UNK | 1.357 | 1.42 | 0.901 |
| CPIJ017319 | hypothetical protein | UNK | 0.906 | 1.825 | 1.727 |
| CPIJ001424 | conserved hypothetical protein | UNK | 1.087 | 2.46 | 1.656 |
| CPIJ001940 | hypothetical protein | UNK | 1.391 | 3.014 | 0.898 |
| CPIJ004598 | Acetoin | UNK | -1.66 | -1.799 | -1.038 |
| CPIJ005302 | conserved hypothetical protein | UNK | 0.775 | 1.655 | 1.178 |
| CPIJ005369 | conserved hypothetical protein | UNK | -0.789 | -1.691 | -1.098 |
| CPIJ007645 | conserved hypothetical protein | UNK | 1.809 | 3.942 | 3.691 |
| CPIJ011592 | hypothetical protein | UNK | -1.107 | -1.731 | -1.621 |
| CPIJ011770 | hypothetical protein | UNK | 1.506 | 2.407 | 2.582 |
| CPIJ012254 | conserved hypothetical protein | UNK | 2.142 | 3.021 | 1.288 |
| CPIJ014162 | BTB/POZ domain containing protein | UNK | -1.162 | -1.087 | -1.178 |
| CPIJ016398 | GTP-binding protein | UNK | 1.139 | 1.566 | 1.078 |
| CPIJ016492 | long-chain-fatty-acid-CoA ligase 1 | UNK | -0.953 | -1.621 | -0.789 |
| CPIJ016555 | conserved hypothetical protein | UNK | 1.208 | 2.468 | 1.786 |
| CPIJ017295 | NULL | UNK | 1.093 | 2.653 | 1.839 |
| CPIJ017746 | conserved hypothetical protein | UNK | -1.24 | -1.91 | -1.647 |
| CPIJ018625 | glutathione-s-transferase theta, gst | UNK | -1.517 | -1.705 | -1.244 |
| CPIJ018923 | conserved hypothetical protein | UNK | 1.189 | 2.184 | 1.592 |
| CPIJ019789 | hypothetical protein | UNK | 1.317 | 1.348 | 0.965 |
| CPIJ019996 | conserved hypothetical protein | UNK | -1.321 | -1.354 | -0.929 |
| CPIJ005269 | conserved hypothetical protein | UNK | 2.218 | 1.585 | 0.944 |
| CPIJ009976 | conserved hypothetical protein | UNK | 0.89 | 0.771 | 0.842 |
| CPIJ011318 | conserved hypothetical protein | UNK | 2.532 | 4.618 | 1.399 |
| CPIJ014495 | transient receptor potential cation channel protein painless | UNK | 1.389 | 1.521 | 1.373 |
| CPIJ000211 | cysteine-rich secretory protein-2 | UNK | 1.629 | 2.391 | 1.986 |
| CPIJ000343 | valacyclovir hydrolase | UNK | 1.075 | 1.26 | 1.345 |
| CPIJ000466 | conserved hypothetical protein | UNK | 1.614 | 1.205 | 0.921 |
| CPIJ000490 | conserved hypothetical protein | UNK | 1.027 | 1.434 | 0.899 |
| CPIJ000492 | conserved hypothetical protein | UNK | 0.837 | 1.059 | 0.867 |
| CPIJ000500 | conserved hypothetical protein | UNK | 2.049 | 3.691 | 2.999 |
| CPIJ000676 | conserved hypothetical protein | UNK | 1.206 | 1.688 | 1.855 |
| CPIJ000942 | Misexpression suppressor of ras | UNK | 0.818 | 1.337 | 1.26 |
| CPIJ001262 | alkaline phosphatase | UNK | -1.991 | -1.203 | -1.685 |
| CPIJ001667 | conserved hypothetical protein | UNK | 0.858 | 1.029 | 1.163 |
| CPIJ002272 | conserved hypothetical protein | UNK | 0.791 | 1.704 | 1.232 |
| CPIJ002572 | conserved hypothetical protein | UNK | 0.833 | 1.905 | 1.791 |
| CPIJ002612 | sensory appendage protein | UNK | 2.333 | 3.461 | 3.637 |
| CPIJ002673 | conserved hypothetical protein | UNK | 1.539 | 1.487 | 1.902 |
| CPIJ002695 | conserved hypothetical protein | UNK | 0.823 | 0.799 | 1.58 |
| CPIJ003180 | WS beta-transducin repeats protein | UNK | 0.996 | 1.418 | 2.027 |
| CPIJ003536 | conserved hypothetical protein | UNK | 1.223 | 2.845 | 2.819 |
| CPIJ003663 | conserved hypothetical protein | UNK | 0.833 | 0.907 | 1.194 |
| CPIJ003938 | conserved hypothetical protein | UNK | 1.704 | 1.84 | 1.512 |
| CPIJ004065 | conserved hypothetical protein | UNK | 2.06 | 2.103 | 1.67 |
| CPIJ004943 | hypothetical protein | UNK | -0.814 | -1.949 | -2.61 |
| CPIJ005300 | sugar transporter | UNK | -0.895 | -1.883 | -2.012 |
| CPIJ005325 | hypothetical protein | UNK | 0.896 | -0.819 | -1.687 |
| CPIJ005544 | nuclear pore complex protein nup93 | UNK | 1.022 | 1.843 | 0.819 |
| CPIJ006519 | hypothetical protein | UNK | 1.771 | 3.92 | 3.106 |
| CPIJ007305 | conserved hypothetical protein | UNK | 2.288 | 3.49 | 3.398 |
| CPIJ007369 | conserved hypothetical protein | UNK | 0.794 | 1.624 | 1.518 |
| CPIJ007584 | conserved hypothetical protein | UNK | 0.803 | 1.82 | 1.699 |
| CPIJ009630 | transcription factor IIIA | UNK | 1.036 | 1.074 | 1.02 |
| CPIJ010077 | enhancer of polycomb | UNK | 0.818 | 1.647 | 1.583 |
| CPIJ010400 | conserved hypothetical protein | UNK | 0.896 | 1.383 | 1.088 |
| CPIJ011133 | predicted protein | UNK | 1.239 | 1.197 | 1.216 |
| CPIJ011664 | conserved hypothetical protein | UNK | 1.327 | 1.37 | 0.946 |
| CPIJ012076 | conserved hypothetical protein | UNK | 0.807 | 1.655 | 1.953 |
| CPIJ012298 | conserved hypothetical protein | UNK | -1.098 | -1.139 | -1.416 |
| CPIJ014044 | conserved hypothetical protein | UNK | 1.018 | 1.514 | 1.204 |
| CPIJ014088 | conserved hypothetical protein | UNK | 1.277 | 2.187 | 1.991 |
| CPIJ014161 | conserved hypothetical protein | UNK | -1.372 | -1.425 | -1.055 |
| CPIJ014903 | zinc metalloprotease | UNK | 0.808 | 1.055 | 1.046 |
| CPIJ015367 | conserved hypothetical protein | UNK | 1.246 | 1.339 | 1.299 |
| CPIJ015898 | conserved hypothetical protein | UNK | 0.796 | 1.555 | 0.873 |
| CPIJ016094 | conserved hypothetical protein | UNK | 1.098 | 1.687 | 1.567 |
| CPIJ016280 | conserved hypothetical protein | UNK | -0.937 | -1.028 | -0.869 |
| CPIJ016399 | conserved hypothetical protein | UNK | 0.795 | 2.481 | 1.829 |
| CPIJ016792 | hypothetical protein | UNK | 0.76 | 1.658 | 2.424 |
| CPIJ017722 | gamma glutamyl transpeptidase | UNK | -1.446 | -1.415 | -1.569 |
| CPIJ018014 | cell division protein ftsj | UNK | 1.981 | 0.906 | 0.998 |
| CPIJ019637 | conserved hypothetical protein | UNK | 0.892 | 1.493 | 1.424 |
| CPIJ000050 | carboxylesterase | UNK |  | -2.592 | -2.589 |
| CPIJ000341 | hypothetical protein | UNK |  | -1.015 | -1.615 |
| CPIJ004836 | conserved hypothetical protein | UNK |  | 1.762 | 1.692 |
| CPIJ005090 | conserved hypothetical protein | UNK |  | -1.548 | -1.697 |
| CPIJ007445 | hypothetical protein | UNK |  | -1.582 | -2.483 |
| CPIJ007446 | hypothetical protein | UNK |  | -1.03 | -1.669 |
| CPIJ010801 | carboxypeptidase B | UNK |  | -1.683 | -2.227 |
| CPIJ018445 | downstream neighbor of Son | UNK |  | 1.184 | 1.328 |
| CPIJ004816 | conserved hypothetical protein | UNK |  | -1.841 | -2.725 |
| CPIJ019182 | ribonuclease UK114 | UNK |  | -0.777 | -1.126 |
| CPIJ001350 | conserved hypothetical protein | UNK |  | -1.26 | -2.513 |
| CPIJ002632 | conserved hypothetical protein | UNK |  | 0.867 | 3.148 |
| CPIJ004230 | lipase | UNK |  | -0.924 | -1.447 |
| CPIJ006537 | larval serum protein 1 beta chain | UNK |  | -2.262 | -3.433 |
| CPIJ007443 | hypothetical protein | UNK |  | -0.824 | -1.881 |
| CPIJ007447 | hypothetical protein | UNK |  | -0.907 | -1.852 |
| CPIJ007830 | conserved hypothetical protein | UNK |  | -1.808 | -2.199 |
| CPIJ008527 | glycoside hydrolase | UNK |  | -2.076 | -3.491 |
| CPIJ018825 | larval serum protein 1 beta chain | UNK |  | -2.008 | -2.906 |
| CPIJ002692 | adipocyte plasma membrane-associated protein | UNK |  | -1.143 | -1.228 |
| CPIJ000438 | serine protease | UNK |  | -3.958 | -2.993 |
| CPIJ007652 | synaptic vesicle protein | UNK |  | -1.9 | -1.715 |
| CPIJ011977 | helicase conserved C-terminal domain containing protein | UNK |  | 2.192 | 2.052 |
| CPIJ017116 | conserved hypothetical protein | UNK |  | -1.656 | -1.203 |
| CPIJ001049 | protease m1 zinc metalloprotease | UNK |  | -2.867 | -4.536 |
| CPIJ004227 | lipase | UNK |  | -2.102 | -3.223 |
| CPIJ008857 | conserved hypothetical protein | UNK |  | -2.145 | -3.494 |
| CPIJ008858 | conserved hypothetical protein | UNK |  | -1.86 | -2.864 |
| CPIJ009003 | conserved hypothetical protein | UNK |  | -2.653 | -3.07 |
| CPIJ011256 | hypothetical protein | UNK |  | -0.856 | 1.313 |
| CPIJ011682 | conserved hypothetical protein | UNK |  | -2.204 | -2.531 |
| CPIJ011987 | conserved hypothetical protein | UNK |  | -0.899 | -1.36 |
| CPIJ013872 | conserved hypothetical protein | UNK |  | 1.989 | 2.119 |
| CPIJ014047 | inosine-uridine preferring nucleoside hydrolase | UNK |  | -0.784 | -1.765 |
| CPIJ014817 | Juvenile hormone-inducible protein | UNK |  | -1.624 | -2.213 |
| CPIJ017083 | conserved hypothetical protein | UNK |  | 0.908 | 1.592 |
| CPIJ017717 | conserved hypothetical protein | UNK |  | -1.714 | -2.081 |
| CPIJ019238 | hypothetical protein | UNK |  | -0.779 | 1.172 |
| CPIJ003010 | conserved hypothetical protein | UNK |  | 1.471 | 0.817 |
| CPIJ006083 | conserved hypothetical protein | UNK |  | -1.688 | -1.212 |
| CPIJ010696 | conserved hypothetical protein | UNK |  | 0.953 | 0.802 |
| CPIJ005012 | conserved hypothetical protein | UNK |  | 1.869 | 3.101 |
| CPIJ002149 | phenylalanine hydroxylase | UNK |  | 1.77 | 2.123 |
| CPIJ007365 | conserved hypothetical protein | UNK |  | 1.419 | 1.48 |
| CPIJ008273 | transcription factor IIIB 90 kDa subunit | UNK |  | 0.89 | 0.936 |
| CPIJ008528 | glycoside hydrolase | UNK |  | -1.674 | -1.746 |
| CPIJ010565 | hemolymph protein-like protein | UNK |  | 0.871 | 0.821 |
| CPIJ016283 | conserved hypothetical protein | UNK |  | 1.049 | 0.912 |
| CPIJ019360 | predicted protein | UNK |  | 0.907 | 0.759 |
| CPIJ015242 | hypothetical protein | UNK |  | 1.332 | 2.761 |
| CPIJ000564 | conserved hypothetical protein | UNK |  | -1.741 | -1.373 |
| CPIJ001773 | synaptic vesicle protein | UNK |  | -1.811 | -1.649 |
| CPIJ002103 | conserved hypothetical protein | UNK |  | 2.672 | 2.031 |
| CPIJ005174 | hypothetical protein | UNK |  | 1.475 | 1.538 |
| CPIJ008016 | conserved hypothetical protein | UNK |  | 1.493 | 1.23 |
| CPIJ008519 | conserved hypothetical protein | UNK |  | -1.258 | -1.181 |
| CPIJ011924 | conserved hypothetical protein | UNK |  | -1.036 | -1.118 |
| CPIJ018631 | glutathione-s-transferase theta, gst | UNK |  | -1.939 | -0.778 |
| CPIJ007582 | Vanin-like protein 1 | UNK |  | 1.456 | 1.783 |
| CPIJ017593 | pantetheinase | UNK |  | 1.055 | 1.133 |
| CPIJ000136 | conserved hypothetical protein | UNK |  | 1.282 | 1.194 |
| CPIJ000348 | pregnancy zone protein | UNK |  | 1.614 | 1.431 |
| CPIJ000455 | ribonuclease UK114 | UNK |  | -1.247 | -1.541 |
| CPIJ000456 | ribonuclease UK114 | UNK |  | -1.876 | -2.579 |
| CPIJ000488 | conserved hypothetical protein | UNK |  | 1.191 | 1.127 |
| CPIJ000536 | secreted protein | UNK |  | 1.408 | 1.539 |
| CPIJ001036 | conserved hypothetical protein | UNK |  | -1.583 | -2.271 |
| CPIJ001076 | hypothetical protein | UNK |  | -0.976 | -0.85 |
| CPIJ001776 | synaptic vesicle protein | UNK |  | -1.217 | -1.087 |
| CPIJ001811 | conserved hypothetical protein | UNK |  | 1.12 | 1.565 |
| CPIJ001974 | conserved hypothetical protein | UNK |  | -0.967 | -0.909 |
| CPIJ002145 | prominin protein | UNK |  | 2.196 | 2.138 |
| CPIJ002275 | conserved hypothetical protein | UNK |  | 0.895 | 1.059 |
| CPIJ002367 | conserved hypothetical protein | UNK |  | 0.946 | 0.923 |
| CPIJ002513 | conserved hypothetical protein | UNK |  | -1.193 | -1.567 |
| CPIJ002674 | glutathione S-transferase 1 | UNK |  | -1.435 | -1.546 |
| CPIJ002803 | conserved hypothetical protein | UNK |  | 1.101 | 1.323 |
| CPIJ002805 | hypothetical protein | UNK |  | 0.797 | 1.131 |
| CPIJ003065 | hypothetical protein | UNK |  | 1.015 | 1.134 |
| CPIJ004043 | clip-domain serine protease | UNK |  | 0.926 | 1.698 |
| CPIJ004223 | pancreatic triacylglycerol lipase | UNK |  | -1.957 | -2.808 |
| CPIJ004235 | conserved hypothetical protein | UNK |  | -1.678 | -1.542 |
| CPIJ004344 | conserved hypothetical protein | UNK |  | 1.008 | 1.154 |
| CPIJ004563 | conserved hypothetical protein | UNK |  | -0.772 | -1.007 |
| CPIJ004707 | vasodilator-stimulated phosphoprotein | UNK |  | 0.833 | 1.002 |
| CPIJ004946 | leucine-rich repeat-containing protein 15 | UNK |  | -0.834 | -1.047 |
| CPIJ006229 | phospholipid-transporting ATPase 1 | UNK |  | 0.855 | 0.85 |
| CPIJ006306 | ion channel nompc | UNK |  | -1.739 | -2.075 |
| CPIJ006348 | conserved hypothetical protein | UNK |  | -0.865 | -0.867 |
| CPIJ006459 | long-chain-fatty-acid-CoA ligase | UNK |  | 0.952 | 1.259 |
| CPIJ006674 | malate dehydrogenase | UNK |  | -0.873 | -0.937 |
| CPIJ006746 | Vps16B | UNK |  | 0.974 | 0.994 |
| CPIJ006840 | CD109 antigen | UNK |  | -0.946 | -0.993 |
| CPIJ006965 | conserved hypothetical protein | UNK |  | -0.837 | -0.946 |
| CPIJ006973 | archease | UNK |  | 0.849 | 1.889 |
| CPIJ007539 | hypothetical protein | UNK |  | -1.116 | -1.361 |
| CPIJ007540 | conserved hypothetical protein | UNK |  | 1.19 | 1.014 |
| CPIJ007941 | hypothetical protein | UNK |  | -0.977 | -0.848 |
| CPIJ008319 | conserved hypothetical protein | UNK |  | 0.998 | 1.058 |
| CPIJ008860 | conserved hypothetical protein | UNK |  | -1.032 | -1.277 |
| CPIJ008875 | prolylcarboxypeptidase | UNK |  | -1.451 | -2.468 |
| CPIJ008882 | conserved hypothetical protein | UNK |  | -0.806 | -1.499 |
| CPIJ009488 | conserved hypothetical protein | UNK |  | 0.801 | 1.155 |
| CPIJ009535 | conserved hypothetical protein | UNK |  | -0.783 | -1.206 |
| CPIJ009636 | conserved hypothetical protein | UNK |  | 1.422 | 0.917 |
| CPIJ009903 | hypothetical protein | UNK |  | -0.952 | -1.574 |
| CPIJ010015 | conserved hypothetical protein | UNK |  | 0.888 | 1.077 |
| CPIJ010269 | hypothetical protein | UNK |  | 1.173 | 2.489 |
| CPIJ010307 | conserved hypothetical protein | UNK |  | -0.771 | -1.024 |
| CPIJ010316 | conserved hypothetical protein | UNK |  | -0.999 | -0.792 |
| CPIJ010444 | hypothetical protein | UNK |  | 1.642 | 1.051 |
| CPIJ010794 | nmda receptor glutamate-binding chain | UNK |  | 0.847 | 0.951 |
| CPIJ011111 | conserved hypothetical protein | UNK |  | -0.92 | -1.119 |
| CPIJ011344 | conserved hypothetical protein | UNK |  | -0.909 | -0.882 |
| CPIJ011505 | conserved hypothetical protein | UNK |  | -1.161 | -1.45 |
| CPIJ011771 | conserved hypothetical protein | UNK |  | 1.739 | 1.43 |
| CPIJ011926 | conserved hypothetical protein | UNK |  | 1.293 | 0.925 |
| CPIJ012119 | conserved hypothetical protein | UNK |  | -1.29 | -1.385 |
| CPIJ012166 | conserved hypothetical protein | UNK |  | 1.071 | 1.212 |
| CPIJ012672 | vacuolar protein sorting-associated protein | UNK |  | 1.149 | 1.21 |
| CPIJ012917 | conserved hypothetical protein | UNK |  | 1.109 | 1.45 |
| CPIJ013047 | predicted protein | UNK |  | 1.353 | 1.823 |
| CPIJ013635 | adipocyte plasma membrane-associated protein | UNK |  | -1.304 | -1.386 |
| CPIJ014064 | conserved hypothetical protein | UNK |  | 0.9 | 0.784 |
| CPIJ014108 | conserved hypothetical protein | UNK |  | -0.847 | -0.92 |
| CPIJ014353 | glutamate transporter | UNK |  | -0.85 | -1.048 |
| CPIJ014598 | conserved hypothetical protein | UNK |  | -0.889 | -1.132 |
| CPIJ014659 | conserved hypothetical protein | UNK |  | -1.155 | -1.901 |
| CPIJ014786 | conserved hypothetical protein | UNK |  | 1.194 | 0.913 |
| CPIJ015287 | conserved hypothetical protein | UNK |  | 1.112 | 1.778 |
| CPIJ015550 | conserved hypothetical protein | UNK |  | -0.842 | -0.966 |
| CPIJ015743 | conserved hypothetical protein | UNK |  | -0.977 | -1.724 |
| CPIJ015918 | conserved hypothetical protein | UNK |  | -1.604 | -1.107 |
| CPIJ015919 | NULL | UNK |  | -1.166 | -1.449 |
| CPIJ015944 | predicted protein | UNK |  | 1.127 | 0.86 |
| CPIJ016343 | conserved hypothetical protein | UNK |  | 1.881 | 1.76 |
| CPIJ016805 | conserved hypothetical protein | UNK |  | -0.753 | -1.494 |
| CPIJ016875 | conserved hypothetical protein | UNK |  | 1.448 | 1.648 |
| CPIJ017076 | conserved hypothetical protein | UNK |  | -1.758 | -1.92 |
| CPIJ017197 | conserved hypothetical protein | UNK |  | 0.919 | 1.344 |
| CPIJ017751 | conserved hypothetical protein | UNK |  | 1.184 | 0.815 |
| CPIJ018100 | conserved hypothetical protein | UNK |  | -0.8 | -1.601 |
| CPIJ018193 | hypothetical protein | UNK |  | 1.211 | 1.679 |
| CPIJ018586 | conserved hypothetical protein | UNK |  | 0.915 | 0.986 |
| CPIJ019271 | conserved hypothetical protein | UNK |  | -1.338 | -1.651 |
| CPIJ019343 | nuclear matrix protein | UNK |  | -0.83 | -1.036 |
| CPIJ019926 | hypothetical protein | UNK |  | -1.562 | -2.025 |
| CPIJ019929 | conserved hypothetical protein | UNK |  | -0.982 | -1.047 |
| CPIJ020038 | conserved hypothetical protein | UNK |  | 0.871 | 0.833 |
| CPIJ020076 | archease | UNK |  | 1.044 | 1.686 |
| CPIJ020145 | glycerol kinase | UNK |  | -1.3 | -1.662 |
| CPIJ020266 | conserved hypothetical protein | UNK |  | 1.206 | 0.96 |
| CPIJ020312 | conserved hypothetical protein | UNK |  | 1.34 | 1.032 |
| CPIJ008246 | conserved hypothetical protein | UNK | -1.646 |  | 0.977 |
| CPIJ008797 | conserved hypothetical protein | UNK | 1.341 |  | -0.847 |
| CPIJ002072 | conserved hypothetical protein | UNK | -1.431 |  | 2.265 |
| CPIJ002447 | helicase conserved C-terminal domain containing protein | UNK | 0.876 |  | 0.97 |
| CPIJ003534 | conserved hypothetical protein | UNK | -0.904 |  | 1.83 |
| CPIJ009902 | predicted protein | UNK | -0.992 |  | -2.157 |
| CPIJ011188 | conserved hypothetical protein | UNK | -0.841 |  | 1.431 |
| CPIJ000560 | conserved hypothetical protein | UNK | 1.772 |  | -2.114 |
| CPIJ012929 | conserved hypothetical protein | UNK | -1.087 |  | 2.047 |
| CPIJ017461 | aldo-keto reductase | UNK | 0.896 |  | -1.377 |
| CPIJ003955 | predicted protein | UNK | 1.153 |  | -1.791 |
| CPIJ002849 | conserved hypothetical protein | UNK | 1.006 |  | 1.412 |
| CPIJ010154 | conserved hypothetical protein | UNK | 0.965 |  | -0.846 |
| CPIJ014763 | conserved hypothetical protein | UNK | 0.771 |  | 0.814 |
| CPIJ012007 | conserved hypothetical protein | UNK |  |  | 0.999 |
| CPIJ016224 | anionic trypsin-2 | UNK |  |  | 0.898 |
| CPIJ019825 | conserved hypothetical protein | UNK |  |  | -0.978 |
| CPIJ000437 | serine protease | UNK |  |  | -2.201 |
| CPIJ002137 | serine protease1/2 | UNK |  |  | -2.724 |
| CPIJ006478 | 3-hydroxyacyl-coa dehyrogenase | UNK |  |  | -1.018 |
| CPIJ015403 | serine protease | UNK |  |  | -2.451 |
| CPIJ011255 | hypothetical protein | UNK |  |  | 1.335 |
| CPIJ019006 | conserved hypothetical protein | UNK |  |  | -0.848 |
| CPIJ002233 | sphingomyelin synthetase | UNK |  |  | 1.27 |
| CPIJ013521 | conserved hypothetical protein | UNK |  |  | 1.259 |
| CPIJ013871 | hypothetical protein | UNK |  |  | 1.04 |
| CPIJ016778 | hydroxyacyl-coenzyme A dehydrogenase, mitochondrial | UNK |  |  | -0.919 |
| CPIJ019149 | hypothetical protein | UNK |  |  | -1.598 |
| CPIJ000767 | hypothetical protein | UNK |  |  | 0.795 |
| CPIJ004876 | conserved hypothetical protein | UNK |  |  | -1.283 |
| CPIJ002836 | conserved hypothetical protein | UNK |  |  | -2.08 |
| CPIJ002741 | conserved hypothetical protein | UNK |  |  | -0.953 |
| CPIJ004852 | conserved hypothetical protein | UNK |  |  | -1.245 |
| CPIJ004226 | pancreatic triacylglycerol lipase | UNK |  |  | -0.942 |
| CPIJ009026 | conserved hypothetical protein | UNK |  |  | -0.866 |
| CPIJ000074 | conserved hypothetical protein | UNK |  |  | -0.816 |
| CPIJ000204 | conserved hypothetical protein | UNK |  |  | 1.087 |
| CPIJ000319 | conserved hypothetical protein | UNK |  |  | -1.15 |
| CPIJ000657 | hypothetical protein | UNK |  |  | -0.941 |
| CPIJ000766 | conserved hypothetical protein | UNK |  |  | -1.508 |
| CPIJ000973 | conserved hypothetical protein | UNK |  |  | -1.436 |
| CPIJ000975 | prolyl 4-hydroxylase subunit alpha-1 | UNK |  |  | -1.526 |
| CPIJ001274 | hypothetical protein | UNK |  |  | 0.873 |
| CPIJ001391 | conserved hypothetical protein | UNK |  |  | 0.769 |
| CPIJ001395 | translocase of outer membrane 34 | UNK |  |  | 1.095 |
| CPIJ001902 | hypothetical protein | UNK |  |  | 1.441 |
| CPIJ002013 | hypothetical protein | UNK |  |  | -0.76 |
| CPIJ002681 | glutathione S-transferase | UNK |  |  | -0.775 |
| CPIJ002902 | paramyosin | UNK |  |  | -0.999 |
| CPIJ002986 | arrowhead | UNK |  |  | -0.875 |
| CPIJ003001 | conserved hypothetical protein | UNK |  |  | 0.799 |
| CPIJ003143 | conserved hypothetical protein | UNK |  |  | 0.854 |
| CPIJ003176 | conserved hypothetical protein | UNK |  |  | 0.975 |
| CPIJ004225 | pancreatic triacylglycerol lipase | UNK |  |  | -0.783 |
| CPIJ004520 | conserved hypothetical protein | UNK |  |  | 0.933 |
| CPIJ004549 | conserved hypothetical protein | UNK |  |  | 0.84 |
| CPIJ005420 | PPARgamma constitutive coactivator 1 | UNK |  |  | 1.377 |
| CPIJ005492 | conserved hypothetical protein | UNK |  |  | -1.108 |
| CPIJ005693 | conserved hypothetical protein | UNK |  |  | 0.985 |
| CPIJ006212 | conserved hypothetical protein | UNK |  |  | -0.921 |
| CPIJ006483 | conserved hypothetical protein | UNK |  |  | 0.764 |
| CPIJ006710 | conserved hypothetical protein | UNK |  |  | 1.115 |
| CPIJ006800 | conserved hypothetical protein | UNK |  |  | -1.15 |
| CPIJ007197 | conserved hypothetical protein | UNK |  |  | 1.288 |
| CPIJ007662 | conserved hypothetical protein | UNK |  |  | 0.791 |
| CPIJ007873 | conserved hypothetical protein | UNK |  |  | -1.121 |
| CPIJ007908 | conserved hypothetical protein | UNK |  |  | -1.09 |
| CPIJ008220 | conserved hypothetical protein | UNK |  |  | 0.776 |
| CPIJ008417 | glutamate receptor, ionotropic ampa, subunit 1, 2, 3 | UNK |  |  | -1.145 |
| CPIJ008499 | conserved hypothetical protein | UNK |  |  | -1.35 |
| CPIJ009024 | conserved hypothetical protein | UNK |  |  | 1.016 |
| CPIJ009107 | conserved hypothetical protein | UNK |  |  | -1.993 |
| CPIJ009213 | conserved hypothetical protein | UNK |  |  | -0.941 |
| CPIJ009715 | conserved hypothetical protein | UNK |  |  | -1.562 |
| CPIJ009716 | conserved hypothetical protein | UNK |  |  | -0.803 |
| CPIJ009924 | conserved hypothetical protein | UNK |  |  | -1.065 |
| CPIJ009978 | AMP dependent coa ligase | UNK |  |  | -1.601 |
| CPIJ010234 | conserved hypothetical protein | UNK |  |  | 0.797 |
| CPIJ010270 | conserved hypothetical protein | UNK |  |  | 1.11 |
| CPIJ010506 | serine protease inhibitor, serpin | UNK |  |  | 0.875 |
| CPIJ010741 | pyridoxal phosphate phosphatase | UNK |  |  | 0.959 |
| CPIJ011623 | conserved hypothetical protein | UNK |  |  | -1.172 |
| CPIJ011717 | g-protein coupled receptor | UNK |  |  | -1.048 |
| CPIJ011916 | conserved hypothetical protein | UNK |  |  | 0.778 |
| CPIJ012026 | conserved hypothetical protein | UNK |  |  | 0.861 |
| CPIJ012163 | conserved hypothetical protein | UNK |  |  | -0.807 |
| CPIJ012620 | ybiN | UNK |  |  | 1.229 |
| CPIJ012700 | CHKov1 | UNK |  |  | -1.117 |
| CPIJ012785 | conserved hypothetical protein | UNK |  |  | 0.813 |
| CPIJ013084 | brain chitinase and chia | UNK |  |  | 0.899 |
| CPIJ013195 | conserved hypothetical protein | UNK |  |  | -0.991 |
| CPIJ014337 | conserved hypothetical protein | UNK |  |  | 1.357 |
| CPIJ014462 | conserved hypothetical protein | UNK |  |  | 0.887 |
| CPIJ014949 | conserved hypothetical protein | UNK |  |  | -0.964 |
| CPIJ015108 | conserved hypothetical protein | UNK |  |  | 0.95 |
| CPIJ015227 | acetyl-coA acetyl transferase II | UNK |  |  | 1.241 |
| CPIJ015618 | acetyl-CoA acetyltransferase 2 | UNK |  |  | 1.222 |
| CPIJ016380 | galactokinase | UNK |  |  | -0.759 |
| CPIJ016420 | hypothetical protein | UNK |  |  | -0.795 |
| CPIJ016485 | conserved hypothetical protein | UNK |  |  | -0.896 |
| CPIJ016696 | g-protein coupled receptor | UNK |  |  | -1.075 |
| CPIJ016761 | pol-like protein | UNK |  |  | -0.934 |
| CPIJ017397 | conserved hypothetical protein | UNK |  |  | -1.174 |
| CPIJ017604 | hypothetical protein | UNK |  |  | 1.025 |
| CPIJ018232 | cholinesterase | UNK |  |  | -0.912 |
| CPIJ018233 | carboxylesterase | UNK |  |  | -1.596 |
| CPIJ018313 | conserved hypothetical protein | UNK |  |  | -1.117 |
| CPIJ018416 | brahma-associated protein 111kD | UNK |  |  | -0.865 |
| CPIJ018465 | conserved hypothetical protein | UNK |  |  | -1.327 |
| CPIJ018755 | hypothetical protein | UNK |  |  | -0.845 |
| CPIJ018945 | predicted protein | UNK |  |  | -0.876 |
| CPIJ019827 | conserved hypothetical protein | UNK |  |  | 1.181 |
| CPIJ019918 | conserved hypothetical protein | UNK |  |  | -0.867 |
| CPIJ020092 | conserved hypothetical protein | UNK |  |  | -1.238 |
| CPIJ020308 | conserved hypothetical protein | UNK |  |  | -0.866 |
| CPIJ000881 | conserved hypothetical protein | UNK | -2.286 | -2.647 |  |
| CPIJ003983 | conserved hypothetical protein | UNK | 2.259 | 0.987 |  |
| CPIJ009044 | conserved hypothetical protein | UNK | -1.534 | -1.431 |  |
| CPIJ017118 | conserved hypothetical protein | UNK | -1.381 | -0.891 |  |
| CPIJ017811 | HPS | UNK | 1.899 | 1.185 |  |
| CPIJ014758 | conserved hypothetical protein | UNK | 1.183 | 1.26 |  |
| CPIJ003464 | conserved hypothetical protein | UNK | -1.179 | -0.751 |  |
| CPIJ006140 | conserved hypothetical protein | UNK | -1.061 | -0.976 |  |
| CPIJ006479 | 3-hydroxyacyl-coa dehyrogenase | UNK | -0.752 | -1.21 |  |
| CPIJ005496 | hypothetical protein | UNK | -3.531 | -3.384 |  |
| CPIJ005497 | hypothetical protein | UNK | -3.388 | -3.437 |  |
| CPIJ005498 | hypothetical protein | UNK | -3.434 | -3.406 |  |
| CPIJ007033 | lipase | UNK | -3.371 | -3.568 |  |
| CPIJ012382 | hypothetical protein | UNK | 0.935 | 0.985 |  |
| CPIJ012498 | conserved hypothetical protein | UNK | 1.02 | 1.194 |  |
| CPIJ016923 | fixR | UNK | -1.082 | -1.3 |  |
| CPIJ018749 | hypothetical protein | UNK | -4.387 | -4.439 |  |
| CPIJ003958 | hypothetical protein | UNK | 0.912 | 1.055 |  |
| CPIJ019233 | cerebral protein 1 | UNK |  | 0.865 |  |
| CPIJ014197 | conserved hypothetical protein | UNK |  | -0.751 |  |
| CPIJ005903 | conserved hypothetical protein | UNK |  | 0.872 |  |
| CPIJ006906 | conserved hypothetical protein | UNK |  | -1.365 |  |
| CPIJ018933 | tyrosine-protein kinase receptor Tie-1 | UNK |  | -2.418 |  |
| CPIJ019816 | conserved hypothetical protein | UNK |  | -1.233 |  |
| CPIJ000022 | conserved hypothetical protein | UNK |  | -0.935 |  |
| CPIJ000281 | conserved hypothetical protein | UNK |  | -0.892 |  |
| CPIJ000316 | conserved hypothetical protein | UNK |  | -1.115 |  |
| CPIJ001037 | conserved hypothetical protein | UNK |  | -1.032 |  |
| CPIJ001351 | conserved hypothetical protein | UNK |  | -0.815 |  |
| CPIJ001425 | conserved hypothetical protein | UNK |  | 0.981 |  |
| CPIJ002657 | proclotting enzyme | UNK |  | 0.87 |  |
| CPIJ002771 | cystatin-like protein | UNK |  | 1.168 |  |
| CPIJ003373 | predicted protein | UNK |  | -0.793 |  |
| CPIJ010600 | conserved hypothetical protein | UNK |  | 0.765 |  |
| CPIJ014001 | superoxide dismutase, Mn | UNK |  | -0.758 |  |
| CPIJ015941 | arylalkylamine N-acetyltransferase | UNK |  | 1.122 |  |
| CPIJ016964 | conserved hypothetical protein | UNK |  | 0.76 |  |
| CPIJ017687 | conserved hypothetical protein | UNK |  | -0.86 |  |
| CPIJ017843 | hypothetical protein | UNK |  | 0.982 |  |
| CPIJ018521 | conserved hypothetical protein | UNK |  | -0.791 |  |
| CPIJ018744 | ankyrin 2,3/unc44 | UNK |  | -0.761 |  |
| CPIJ019089 | conserved hypothetical protein | UNK |  | 0.779 |  |
| CPIJ020274 | conserved hypothetical protein | UNK |  | -0.855 |  |
| CPIJ010309 | conserved hypothetical protein | UNK | 0.991 |  |  |
| CPIJ012698 | conserved hypothetical protein | UNK | 0.94 |  |  |
| CPIJ003489 | hypothetical protein | UNK | -0.838 |  |  |
| CPIJ004015 | predicted protein | UNK | 0.957 |  |  |
| CPIJ012956 | conserved hypothetical protein | UNK | -1.007 |  |  |
| CPIJ001427 | conserved hypothetical protein | UNK | 1.144 |  |  |
| CPIJ009838 | conserved hypothetical protein | UNK | -0.778 |  |  |
| CPIJ010655 | conserved hypothetical protein | UNK | -0.856 |  |  |
| CPIJ017666 | conserved hypothetical protein | UNK | -0.826 |  |  |
| CPIJ017910 | conserved hypothetical protein | UNK | 1.329 |  |  |
| CPIJ020172 | conserved hypothetical protein | UNK | -0.751 |  |  |
| CPIJ007468 | conserved hypothetical protein | UNK | -1.184 |  |  |
| CPIJ019390 | conserved hypothetical protein | UNK | 1.047 |  |  |
| CPIJ001548 | conserved hypothetical protein | UNK | 0.879 |  |  |
| CPIJ002410 | conserved hypothetical protein | UNK | -0.753 |  |  |
| CPIJ003386 | predicted protein | UNK | 0.939 |  |  |
| CPIJ013619 | conserved hypothetical protein | UNK | -0.954 |  |  |
| CPIJ013643 | conserved hypothetical protein | UNK | -0.762 |  |  |
| CPIJ019819 | conserved hypothetical protein | UNK | -0.756 |  |  |
| CPIJ001282 | conserved hypothetical protein | UNK | 1.289 | 1.878 | 1.053 |
| CPIJ002679 | glutathione S-transferase theta-2 | UNK | -3.598 | -2.541 | -1.082 |
| CPIJ008302 | conserved hypothetical protein | UNK | 1.041 | 1.083 | 0.752 |
| CPIJ019683 | conserved hypothetical protein | UNK | -1.248 | -1.831 | -1.195 |
| CPIJ018194 | conserved hypothetical protein | UNK | 0.897 | 1.152 | 0.802 |
| CPIJ018116 | conserved hypothetical protein | UNK | -1.122 | -1.359 | -0.788 |
| CPIJ009229 | conserved hypothetical protein | UNK | 1.416 | 0.932 | 0.811 |
| CPIJ003963 | conserved hypothetical protein | UNK | 0.915 | 0.814 | 0.766 |
| CPIJ001345 | conserved hypothetical protein | UNK | 1.001 | 1.165 | 0.765 |
| CPIJ005200 | conserved hypothetical protein | UNK | -1.468 | -0.882 | 0.882 |
| CPIJ006221 | conserved hypothetical protein | UNK | -1.339 | -1.711 | -0.801 |
| CPIJ009740 | LIM protein pin-2 | UNK | 1.181 | 1.406 | 0.831 |
| CPIJ011496 | hypothetical protein | UNK | -2.944 | -3.251 | -0.76 |
| CPIJ015536 | outer mitochondrial translocase subunit | UNK | -1.006 | -1.432 | -0.811 |
| CPIJ018834 | conserved hypothetical protein | UNK | -1.232 | -1.894 | -0.768 |
| CPIJ010758 | conserved hypothetical protein | UNK | -3.551 | -3.921 | -1.917 |
| CPIJ010761 | conserved hypothetical protein | UNK | -2.482 | -2.609 | -0.906 |
| CPIJ019565 | glucose-6-phosphate isomerase | UNK | -0.799 | -1.674 | -0.774 |
| CPIJ001177 | transcription factor | UNK | 1.065 | 1.266 | 0.985 |
| CPIJ003907 | conserved hypothetical protein | UNK | 1.973 | 2.087 | 1.38 |
| CPIJ012948 | conserved hypothetical protein | UNK | 1.392 | 1.607 | 0.97 |
| CPIJ013194 | conserved hypothetical protein | UNK | 1.501 | 1.579 | 1.108 |
| CPIJ002274 | conserved hypothetical protein | UNK | 1.251 | 1.439 | 1.047 |
| CPIJ002957 | sugar transporter | UNK | 0.944 | 0.859 | 0.771 |
| CPIJ006112 | conserved hypothetical protein | UNK | 0.925 | 1.96 | 1.352 |
| CPIJ012786 | predicted protein | UNK | -1.295 | -1.508 | -1.452 |
| CPIJ012899 | secreted protein | UNK | -0.956 | -1.196 | -0.893 |
| CPIJ010625 | hypothetical protein | UNK |  | 1.765 | 1.278 |
| CPIJ012889 | nonsense-mediated mRNA decay protein | UNK |  | 1.289 | 0.853 |
| CPIJ017875 | hypothetical protein | UNK |  | 0.966 | 0.804 |
| CPIJ012798 | conserved hypothetical protein | UNK |  | -1.658 | -0.778 |
| CPIJ017337 | conserved hypothetical protein | UNK |  | -1.387 | -0.824 |
| CPIJ000016 | conserved hypothetical protein | UNK |  | -1.55 | -0.874 |
| CPIJ001186 | conserved hypothetical protein | UNK |  | 0.837 | 1.035 |
| CPIJ003604 | conserved hypothetical protein | UNK |  | 1.095 | 0.761 |
| CPIJ005585 | conserved hypothetical protein | UNK |  | 1.129 | 1.073 |
| CPIJ005861 | conserved hypothetical protein | UNK |  | 0.822 | 0.774 |
| CPIJ007955 | conserved hypothetical protein | UNK |  | 0.833 | 0.839 |
| CPIJ008952 | conserved hypothetical protein | UNK |  | 1.255 | 0.924 |
| CPIJ010313 | conserved hypothetical protein | UNK |  | -0.78 | -0.806 |
| CPIJ010622 | conserved hypothetical protein | UNK |  | 1.162 | 0.876 |
| CPIJ010709 | molybdopterin cofactor sulfurase | UNK |  | -1.097 | -0.892 |
| CPIJ011089 | translin associated factor x | UNK |  | -1.012 | -0.84 |
| CPIJ011207 | conserved hypothetical protein | UNK |  | 1.006 | 0.909 |
| CPIJ012307 | galactose-specific C-type lectin | UNK |  | -1.356 | -0.947 |
| CPIJ013405 | suppressor of ty | UNK |  | -0.862 | -0.83 |
| CPIJ014239 | conserved hypothetical protein | UNK |  | 0.988 | 1.217 |
| CPIJ019682 | conserved hypothetical protein | UNK |  | -0.978 | -0.804 |
| CPIJ013511 | hypothetical protein | UNK | 1.777 |  | -0.862 |
| CPIJ039733 | NULL | UNK | 2.333 |  | 1.03 |
| CPIJ002833 | conserved hypothetical protein | UNK | -1.718 |  | -1.466 |
| CPIJ015112 | E3 ubiquitin-protein ligase nedd-4 | UNK | 0.933 |  | 0.795 |
| CPIJ005505 | hypothetical protein | UNK | -0.755 |  | -0.912 |
| CPIJ016518 | conserved hypothetical protein | UNK | 0.911 |  | 0.913 |
| CPIJ016891 | predicted protein | UNK | -1.114 |  | 0.792 |
| CPIJ007777 | conserved hypothetical protein | UNK |  |  | -1.102 |
| CPIJ010738 | FAM96A | UNK |  |  | -1.108 |
| CPIJ016655 | conserved hypothetical protein | UNK |  |  | 0.948 |
| CPIJ017967 | ovochymase-2 | UNK |  |  | 0.915 |
| CPIJ015193 | zinc finger protein 10 | UNK |  |  | 0.92 |
| CPIJ002461 | hect E3 ubiquitin ligase | UNK |  |  | -0.929 |
| CPIJ005964 | Na+/K+ ATPase alpha subunit | UNK |  |  | -0.765 |
| CPIJ000052 | conserved hypothetical protein | UNK |  |  | 0.768 |
| CPIJ000121 | conserved hypothetical protein | UNK |  |  | 0.82 |
| CPIJ000692 | conserved hypothetical protein | UNK |  |  | 0.991 |
| CPIJ001715 | conserved hypothetical protein | UNK |  |  | -1.101 |
| CPIJ001865 | predicted protein | UNK |  |  | 0.884 |
| CPIJ001901 | predicted protein | UNK |  |  | 0.847 |
| CPIJ001935 | conserved hypothetical protein | UNK |  |  | 0.762 |
| CPIJ002234 | conserved hypothetical protein | UNK |  |  | 0.98 |
| CPIJ003144 | conserved hypothetical protein | UNK |  |  | 0.796 |
| CPIJ003575 | ionotropic glutamate receptor-invertebrate | UNK |  |  | 0.801 |
| CPIJ004570 | conserved hypothetical protein | UNK |  |  | 0.879 |
| CPIJ005391 | conserved hypothetical protein | UNK |  |  | -0.881 |
| CPIJ006371 | conserved hypothetical protein | UNK |  |  | 0.76 |
| CPIJ007946 | conserved hypothetical protein | UNK |  |  | 0.817 |
| CPIJ008318 | conserved hypothetical protein | UNK |  |  | 0.816 |
| CPIJ008501 | conserved hypothetical protein | UNK |  |  | -1.094 |
| CPIJ009395 | speckle-type poz protein | UNK |  |  | 0.764 |
| CPIJ009398 | conserved hypothetical protein | UNK |  |  | -0.82 |
| CPIJ011828 | predicted protein | UNK |  |  | 0.761 |
| CPIJ011900 | predicted protein | UNK |  |  | 0.971 |
| CPIJ012669 | conserved hypothetical protein | UNK |  |  | 0.756 |
| CPIJ013696 | hypothetical protein | UNK |  |  | 0.761 |
| CPIJ014801 | conserved hypothetical protein | UNK |  |  | 0.973 |
| CPIJ015321 | conserved hypothetical protein | UNK |  |  | 0.87 |
| CPIJ018079 | conserved hypothetical protein | UNK |  |  | -0.822 |
| CPIJ018903 | conserved hypothetical protein | UNK |  |  | 0.771 |
| CPIJ019578 | hypothetical protein | UNK |  |  | -0.91 |
| CPIJ004809 | hypothetical protein | UNK | 1.789 | 1.225 |  |
| CPIJ007855 | zinc finger protein 667 | UNK | 1.205 | 1.022 |  |
| CPIJ008500 | conserved hypothetical protein | UNK | 1.664 | 1.783 |  |
| CPIJ009945 | conserved hypothetical protein | UNK | 2.259 | 1.337 |  |
| CPIJ015917 | conserved hypothetical protein | UNK | 1.489 | 0.903 |  |
| CPIJ012697 | conserved hypothetical protein | UNK | -1.392 | -2.099 |  |
| CPIJ012916 | conserved hypothetical protein | UNK | 1.049 | 1.253 |  |
| CPIJ014715 | zinc finger protein | UNK | 1.159 | 0.808 |  |
| CPIJ014787 | conserved hypothetical protein | UNK | 1.336 | 1.283 |  |
| CPIJ014901 | conserved hypothetical protein | UNK | -1.201 | -1.803 |  |
| CPIJ015672 | inhibitor of nuclear factor kappa B kinase beta subunit | UNK | 1.122 | 1.155 |  |
| CPIJ019282 | NudC domain containing 1 | UNK | 0.932 | 0.993 |  |
| CPIJ003964 | conserved hypothetical protein | UNK | 1.732 | 1.377 |  |
| CPIJ008435 | conserved hypothetical protein | UNK | -0.934 | -1.455 |  |
| CPIJ015226 | conserved hypothetical protein | UNK | 1.193 | 1.012 |  |
| CPIJ018565 | peptide methionine sulfoxide reductase | UNK | -0.875 | 0.919 |  |
| CPIJ003465 | conserved hypothetical protein | UNK | -1.611 | -0.941 |  |
| CPIJ003467 | conserved hypothetical protein | UNK | -1.276 | -1.027 |  |
| CPIJ003542 | conserved hypothetical protein | UNK | 0.804 | 1.12 |  |
| CPIJ014587 | conserved hypothetical protein | UNK | 1.355 | 0.942 |  |
| CPIJ018297 | hypothetical protein | UNK | 1.153 | 0.769 |  |
| CPIJ000572 | steroid dehydrogenase | UNK | -1.219 | -1.586 |  |
| CPIJ001128 | valacyclovir hydrolase | UNK | -2.179 | -1.854 |  |
| CPIJ001912 | conserved hypothetical protein | UNK | -0.978 | -1.353 |  |
| CPIJ002000 | conserved hypothetical protein | UNK | 1.008 | 0.853 |  |
| CPIJ002068 | conserved hypothetical protein | UNK | -1.535 | -1.344 |  |
| CPIJ005390 | conserved hypothetical protein | UNK | -1.271 | -1.902 |  |
| CPIJ006280 | conserved hypothetical protein | UNK | -0.925 | -1.014 |  |
| CPIJ007073 | l | UNK | -1.667 | -1.724 |  |
| CPIJ007074 | l | UNK | -1.776 | -1.753 |  |
| CPIJ007748 | conserved hypothetical protein | UNK | -1.119 | -1.21 |  |
| CPIJ008211 | conserved hypothetical protein | UNK | -1.775 | -2.059 |  |
| CPIJ008404 | conserved hypothetical protein | UNK | -1.269 | -1.32 |  |
| CPIJ008932 | conserved hypothetical protein | UNK | 1.198 | 1.206 |  |
| CPIJ009045 | conserved hypothetical protein | UNK | -1.767 | -1.479 |  |
| CPIJ009054 | coiled-coil domain-containing protein 47 | UNK | -1.605 | -1.556 |  |
| CPIJ009427 | conserved hypothetical protein | UNK | 1.03 | 1.176 |  |
| CPIJ009790 | conserved hypothetical protein | UNK | -1.65 | -1.15 |  |
| CPIJ010149 | conserved hypothetical protein | UNK | -1.548 | -1.574 |  |
| CPIJ010308 | conserved hypothetical protein | UNK | -1.675 | -1.764 |  |
| CPIJ010381 | conserved hypothetical protein | UNK | -0.936 | -1.375 |  |
| CPIJ010451 | conserved hypothetical protein | UNK | -1.02 | -1.35 |  |
| CPIJ010457 | conserved hypothetical protein | UNK | -1.15 | -2.012 |  |
| CPIJ011176 | hypothetical protein | UNK | -1.641 | -1.292 |  |
| CPIJ011248 | multiple inositol polyphosphate phosphatase 1 | UNK | 1.295 | 0.751 |  |
| CPIJ011275 | hypothetical protein | UNK | 1.143 | 1.093 |  |
| CPIJ012005 | hypothetical protein | UNK | -1.642 | -1.694 |  |
| CPIJ012029 | conserved hypothetical protein | UNK | 1.437 | 1.39 |  |
| CPIJ012271 | WD and tetratricopeptide repeats protein 1 | UNK | 1.691 | 1.553 |  |
| CPIJ012857 | conserved hypothetical protein | UNK | -1.295 | -1.703 |  |
| CPIJ013088 | conserved hypothetical protein | UNK | -1.273 | -1.741 |  |
| CPIJ013089 | predicted protein | UNK | -0.971 | -1.466 |  |
| CPIJ014272 | conserved hypothetical protein | UNK | -1.596 | -1.577 |  |
| CPIJ014590 | conserved hypothetical protein | UNK | -0.79 | -0.915 |  |
| CPIJ015051 | conserved hypothetical protein | UNK | 1.317 | 1.224 |  |
| CPIJ015928 | conserved hypothetical protein | UNK | 0.953 | 1.045 |  |
| CPIJ016158 | proteasome subunit beta type 3 | UNK | -1.436 | -1.727 |  |
| CPIJ016179 | conserved hypothetical protein | UNK | -1.288 | -1.621 |  |
| CPIJ016620 | conserved hypothetical protein | UNK | -1.037 | -1.732 |  |
| CPIJ016672 | conserved hypothetical protein | UNK | -1.068 | -0.864 |  |
| CPIJ017106 | conserved hypothetical protein | UNK | -1.336 | -1.16 |  |
| CPIJ017471 | hypothetical protein | UNK | -1.969 | -1.542 |  |
| CPIJ017597 | synaptic vesicle protein | UNK | -2.395 | -3.01 |  |
| CPIJ017903 | conserved hypothetical protein | UNK | -1.044 | -1.161 |  |
| CPIJ019894 | conserved hypothetical protein | UNK | -0.852 | -0.918 |  |
| CPIJ020055 | conserved hypothetical protein | UNK | -1.01 | -1.939 |  |
| CPIJ020231 | conserved hypothetical protein | UNK | -1.064 | -1.186 |  |
| CPIJ000575 | oryzain gamma chain | UNK | 1.143 | 1.482 |  |
| CPIJ001120 | conserved hypothetical protein | UNK | 0.998 | 1.059 |  |
| CPIJ002840 | E3 ubiquitin-ligase protein COP1 | UNK | -0.885 | -0.779 |  |
| CPIJ002917 | hypothetical protein | UNK | -0.934 | -1.285 |  |
| CPIJ003716 | conserved hypothetical protein | UNK | -0.902 | -1.128 |  |
| CPIJ003851 | conserved hypothetical protein | UNK | 0.841 | 0.799 |  |
| CPIJ004645 | conserved hypothetical protein | UNK | 1.164 | 1.024 |  |
| CPIJ005045 | conserved hypothetical protein | UNK | -0.923 | -0.979 |  |
| CPIJ006025 | conserved hypothetical protein | UNK | 1.224 | 0.796 |  |
| CPIJ006953 | nuclear protein localization 4 | UNK | -0.814 | -1.103 |  |
| CPIJ007274 | conserved hypothetical protein | UNK | -0.849 | -1.305 |  |
| CPIJ007785 | conserved hypothetical protein | UNK | -0.797 | -1.087 |  |
| CPIJ009344 | conserved hypothetical protein | UNK | -0.994 | -1.181 |  |
| CPIJ010330 | importin beta-3 | UNK | 0.783 | 1.049 |  |
| CPIJ010680 | conserved hypothetical protein | UNK | -0.785 | -1.371 |  |
| CPIJ010839 | L-seryl-tRNA | UNK | -0.97 | -0.872 |  |
| CPIJ010896 | conserved hypothetical protein | UNK | -0.842 | -0.962 |  |
| CPIJ012287 | hypothetical protein | UNK | -1.072 | -1.666 |  |
| CPIJ012312 | conserved hypothetical protein | UNK | 0.769 | 1.143 |  |
| CPIJ012788 | zinc finger protein | UNK | 0.885 | 0.971 |  |
| CPIJ013358 | conserved hypothetical protein | UNK | -0.815 | -0.855 |  |
| CPIJ013366 | conserved hypothetical protein | UNK | -0.828 | -1.548 |  |
| CPIJ013855 | conserved hypothetical protein | UNK | 0.76 | 0.797 |  |
| CPIJ014313 | conserved hypothetical protein | UNK | -0.962 | -0.832 |  |
| CPIJ014569 | conserved hypothetical protein | UNK | -0.855 | -1.363 |  |
| CPIJ014585 | MYG1 | UNK | -0.84 | -1.124 |  |
| CPIJ014751 | conserved hypothetical protein | UNK | 0.93 | 0.83 |  |
| CPIJ015106 | tonin | UNK | -0.914 | -0.981 |  |
| CPIJ015622 | dynein light chain 1, cytoplasmic-like protein | UNK | -0.922 | -1.172 |  |
| CPIJ016731 | conserved hypothetical protein | UNK | -0.931 | -1.252 |  |
| CPIJ018609 | mitochondrial import inner membrane translocase subunit tim44 | UNK | -1.003 | -1.224 |  |
| CPIJ007401 | Autophagy-specific protein | UNK | 1.1 | 1.021 |  |
| CPIJ008508 | predicted protein | UNK | -1.12 | -0.964 |  |
| CPIJ010038 | conserved hypothetical protein | UNK | -1.389 | -1.293 |  |
| CPIJ011174 | conserved hypothetical protein | UNK | -1.202 | -1.028 |  |
| CPIJ013216 | conserved hypothetical protein | UNK | 0.974 | 0.752 |  |
| CPIJ017532 | conserved hypothetical protein | UNK | -0.993 | -0.796 |  |
| CPIJ017828 | predicted protein | UNK | -1.841 | -2.111 |  |
| CPIJ000491 | conserved hypothetical protein | UNK | -1.221 | -1.083 |  |
| CPIJ000533 | conserved hypothetical protein | UNK | 0.864 | 0.847 |  |
| CPIJ000646 | conserved hypothetical protein | UNK | -1.039 | -0.841 |  |
| CPIJ000748 | conserved hypothetical protein | UNK | 1.193 | 1.454 |  |
| CPIJ001067 | E3 ubiquitin-protein ligase FANCL | UNK | -1.065 | -1.332 |  |
| CPIJ001706 | small nuclear ribonucleoprotein sm d2 | UNK | -1.222 | -0.913 |  |
| CPIJ001939 | hypothetical protein | UNK | 0.783 | 0.792 |  |
| CPIJ002830 | arsenite-resistance protein | UNK | -0.813 | -1.015 |  |
| CPIJ002935 | conserved hypothetical protein | UNK | -0.793 | 1.026 |  |
| CPIJ003466 | conserved hypothetical protein | UNK | -0.926 | -0.768 |  |
| CPIJ003561 | sorting nexin | UNK | 0.921 | 0.842 |  |
| CPIJ003884 | Mgat2 | UNK | -0.793 | -0.909 |  |
| CPIJ004472 | conserved hypothetical protein | UNK | -0.8 | -1.09 |  |
| CPIJ005590 | amidase | UNK | 1.038 | 0.816 |  |
| CPIJ005770 | conserved hypothetical protein | UNK | -0.752 | -1.042 |  |
| CPIJ006098 | conserved hypothetical protein | UNK | 0.797 | 0.809 |  |
| CPIJ006516 | conserved hypothetical protein | UNK | 0.885 | 0.77 |  |
| CPIJ008048 | peroxisomal N1-acetyl-spermine/spermidine oxidase | UNK | -0.83 | -1.125 |  |
| CPIJ008933 | conserved hypothetical protein | UNK | -0.963 | -1.043 |  |
| CPIJ009074 | conserved hypothetical protein | UNK | -0.961 | -1.417 |  |
| CPIJ009934 | conserved hypothetical protein | UNK | 1.067 | 0.821 |  |
| CPIJ010523 | U520 | UNK | 1.624 | 2.208 |  |
| CPIJ010554 | hypothetical protein | UNK | -0.959 | -1.458 |  |
| CPIJ010681 | conserved hypothetical protein | UNK | 1.393 | 0.981 |  |
| CPIJ011962 | multidrug resistance-associated protein 14 | UNK | -1.232 | -1.18 |  |
| CPIJ015414 | hypothetical protein | UNK | -0.77 | -0.796 |  |
| CPIJ015532 | ER lumen protein retaining receptor | UNK | -0.77 | -0.936 |  |
| CPIJ017748 | galectin | UNK | -0.96 | -0.769 |  |
| CPIJ018081 | conserved hypothetical protein | UNK | 0.779 | 0.755 |  |
| CPIJ020123 | smad nuclear-interacting protein 1 | UNK | -0.9 | -0.855 |  |
| CPIJ012739 | RNA-binding protein NOB1 | UNK |  | 1.456 |  |
| CPIJ014360 | brix domain-containing protein 1 | UNK |  | 0.878 |  |
| CPIJ015049 | hypothetical protein | UNK |  | 0.783 |  |
| CPIJ015114 | conserved hypothetical protein | UNK |  | 1.035 |  |
| CPIJ006974 | conserved hypothetical protein | UNK |  | 0.882 |  |
| CPIJ000489 | conserved hypothetical protein | UNK |  | 2.425 |  |
| CPIJ002237 | hypothetical protein | UNK |  | -0.813 |  |
| CPIJ004429 | conserved hypothetical protein | UNK |  | 1.164 |  |
| CPIJ005204 | peptide methionine sulfoxide reductase msrA | UNK |  | 0.808 |  |
| CPIJ013870 | hypothetical protein | UNK |  | 0.853 |  |
| CPIJ014232 | conserved hypothetical protein | UNK |  | 1.226 |  |
| CPIJ007570 | conserved hypothetical protein | UNK |  | 1.109 |  |
| CPIJ008523 | serine-type endopeptidase | UNK |  | -0.793 |  |
| CPIJ011624 | hypothetical protein | UNK |  | 1.848 |  |
| CPIJ003556 | conserved hypothetical protein | UNK |  | -0.923 |  |
| CPIJ010321 | conserved hypothetical protein | UNK |  | -2.322 |  |
| CPIJ014710 | conserved hypothetical protein | UNK |  | 0.937 |  |
| CPIJ001135 | chromobox protein | UNK |  | -0.837 |  |
| CPIJ001603 | truncated ER mannose-binding lectin | UNK |  | -0.867 |  |
| CPIJ002418 | conserved hypothetical protein | UNK |  | -1.302 |  |
| CPIJ003328 | conserved hypothetical protein | UNK |  | -0.855 |  |
| CPIJ003358 | cell cycle progression | UNK |  | -1.046 |  |
| CPIJ003404 | conserved hypothetical protein | UNK |  | -1.139 |  |
| CPIJ003777 | conserved hypothetical protein | UNK |  | -0.969 |  |
| CPIJ004527 | conserved hypothetical protein | UNK |  | -1.389 |  |
| CPIJ005596 | conserved hypothetical protein | UNK |  | -1.016 |  |
| CPIJ006850 | galactosyltransferase | UNK |  | -1.299 |  |
| CPIJ007465 | conserved hypothetical protein | UNK |  | -1.004 |  |
| CPIJ008328 | conserved hypothetical protein | UNK |  | 0.882 |  |
| CPIJ008801 | truncated ER mannose-binding lectin | UNK |  | -0.851 |  |
| CPIJ008902 | conserved hypothetical protein | UNK |  | 0.931 |  |
| CPIJ009918 | conserved hypothetical protein | UNK |  | -0.938 |  |
| CPIJ010623 | hypothetical protein | UNK |  | 0.937 |  |
| CPIJ011010 | apyrase | UNK |  | -1.023 |  |
| CPIJ011093 | conserved hypothetical protein | UNK |  | 0.964 |  |
| CPIJ011677 | conserved hypothetical protein | UNK |  | -1.431 |  |
| CPIJ011944 | hypothetical protein | UNK |  | 0.957 |  |
| CPIJ012115 | conserved hypothetical protein | UNK |  | -0.855 |  |
| CPIJ012554 | conserved hypothetical protein | UNK |  | -1.144 |  |
| CPIJ012931 | conserved hypothetical protein | UNK |  | -0.953 |  |
| CPIJ013212 | conserved hypothetical protein | UNK |  | 0.974 |  |
| CPIJ014196 | conserved hypothetical protein | UNK |  | -2.406 |  |
| CPIJ014724 | conserved hypothetical protein | UNK |  | -1.021 |  |
| CPIJ014885 | NMD protein | UNK |  | 1.015 |  |
| CPIJ015265 | zinc finger protein 345 | UNK |  | 0.752 |  |
| CPIJ015391 | serine/threonine-protein kinase rio2 | UNK |  | 0.96 |  |
| CPIJ016020 | hydroxyacylglutathione hydrolase | UNK |  | -0.999 |  |
| CPIJ016274 | conserved hypothetical protein | UNK |  | -0.794 |  |
| CPIJ016884 | conserved hypothetical protein | UNK |  | -1.202 |  |
| CPIJ017372 | conserved hypothetical protein | UNK |  | -0.917 |  |
| CPIJ019777 | conserved hypothetical protein | UNK |  | 1.273 |  |
| CPIJ020079 | conserved hypothetical protein | UNK |  | -1.195 |  |
| CPIJ008846 | basic helix-loop-helix zip transcription factor | UNK |  | 0.856 |  |
| CPIJ000163 | conserved hypothetical protein | UNK |  | 0.804 |  |
| CPIJ000180 | conserved hypothetical protein | UNK |  | 0.774 |  |
| CPIJ000229 | conserved hypothetical protein | UNK |  | 0.801 |  |
| CPIJ000336 | conserved hypothetical protein | UNK |  | -1.124 |  |
| CPIJ000390 | conserved hypothetical protein | UNK |  | 0.926 |  |
| CPIJ000818 | conserved hypothetical protein | UNK |  | -0.946 |  |
| CPIJ000956 | conserved hypothetical protein | UNK |  | 0.912 |  |
| CPIJ001085 | low molecular weight protein-tyrosine-phosphatase | UNK |  | 0.934 |  |
| CPIJ001133 | cyclin-dependent kinase regulatory subunit | UNK |  | -0.841 |  |
| CPIJ001144 | conserved hypothetical protein | UNK |  | -0.812 |  |
| CPIJ001614 | ATP-binding cassette transporter | UNK |  | 0.795 |  |
| CPIJ001763 | Ofd1 protein | UNK |  | 0.995 |  |
| CPIJ001957 | conserved hypothetical protein | UNK |  | 0.794 |  |
| CPIJ002163 | hypothetical protein | UNK |  | 0.898 |  |
| CPIJ002239 | actin-binding protein | UNK |  | 0.781 |  |
| CPIJ002307 | conserved hypothetical protein | UNK |  | -0.766 |  |
| CPIJ002308 | conserved hypothetical protein | UNK |  | -0.843 |  |
| CPIJ002309 | conserved hypothetical protein | UNK |  | -0.795 |  |
| CPIJ002312 | conserved hypothetical protein | UNK |  | 0.922 |  |
| CPIJ002770 | cystatin-like protein | UNK |  | 1.135 |  |
| CPIJ003120 | hypothetical protein | UNK |  | -0.831 |  |
| CPIJ003566 | conserved hypothetical protein | UNK |  | -1.129 |  |
| CPIJ004758 | hypothetical protein | UNK |  | 0.907 |  |
| CPIJ005270 | conserved hypothetical protein | UNK |  | 0.813 |  |
| CPIJ006512 | hypothetical protein | UNK |  | -0.886 |  |
| CPIJ006914 | conserved hypothetical protein | UNK |  | -1.296 |  |
| CPIJ007184 | seryl-tRNA synthetase | UNK |  | -0.762 |  |
| CPIJ007562 | dihydrofolate reductase | UNK |  | -0.794 |  |
| CPIJ007939 | conserved hypothetical protein | UNK |  | -0.892 |  |
| CPIJ008050 | predicted protein | UNK |  | 0.79 |  |
| CPIJ008255 | conserved hypothetical protein | UNK |  | 0.808 |  |
| CPIJ008498 | conserved hypothetical protein | UNK |  | 0.994 |  |
| CPIJ008587 | conserved hypothetical protein | UNK |  | -1.073 |  |
| CPIJ008990 | conserved hypothetical protein | UNK |  | -0.847 |  |
| CPIJ009723 | HSPB1-associated protein 1 | UNK |  | -1.007 |  |
| CPIJ009936 | hypothetical protein | UNK |  | -1.014 |  |
| CPIJ010342 | conserved hypothetical protein | UNK |  | -0.837 |  |
| CPIJ010808 | conserved hypothetical protein | UNK |  | 0.892 |  |
| CPIJ011274 | conserved hypothetical protein | UNK |  | -0.777 |  |
| CPIJ011576 | rhodopsin | UNK |  | 0.756 |  |
| CPIJ011649 | conserved hypothetical protein | UNK |  | -0.842 |  |
| CPIJ011875 | endo beta N-acetyl glucosaminidase | UNK |  | -1.092 |  |
| CPIJ011988 | conserved hypothetical protein | UNK |  | 0.919 |  |
| CPIJ012231 | conserved hypothetical protein | UNK |  | -0.79 |  |
| CPIJ012562 | 26S proteasome non-ATPase regulatory subunit 10 | UNK |  | -0.829 |  |
| CPIJ013363 | conserved hypothetical protein | UNK |  | -1.268 |  |
| CPIJ014036 | conserved hypothetical protein | UNK |  | -0.93 |  |
| CPIJ014420 | conserved hypothetical protein | UNK |  | 1.828 |  |
| CPIJ014520 | conserved hypothetical protein | UNK |  | 0.821 |  |
| CPIJ014529 | conserved hypothetical protein | UNK |  | 0.857 |  |
| CPIJ014684 | conserved hypothetical protein | UNK |  | 0.83 |  |
| CPIJ014838 | conserved hypothetical protein | UNK |  | -0.891 |  |
| CPIJ014999 | conserved hypothetical protein | UNK |  | 0.795 |  |
| CPIJ015058 | conserved hypothetical protein | UNK |  | 0.767 |  |
| CPIJ015142 | conserved hypothetical protein | UNK |  | 0.942 |  |
| CPIJ015194 | zinc finger protein 175 | UNK |  | 0.786 |  |
| CPIJ015250 | hypothetical protein | UNK |  | -1.265 |  |
| CPIJ015370 | conserved hypothetical protein | UNK |  | 0.821 |  |
| CPIJ015382 | charged multivesicular body protein 5 | UNK |  | 1.016 |  |
| CPIJ015643 | ybiN | UNK |  | 0.753 |  |
| CPIJ015793 | conserved hypothetical protein | UNK |  | 0.925 |  |
| CPIJ016047 | charged multivesicular body protein 5 | UNK |  | 0.856 |  |
| CPIJ016120 | conserved hypothetical protein | UNK |  | -0.82 |  |
| CPIJ016364 | conserved hypothetical protein | UNK |  | 0.91 |  |
| CPIJ016660 | conserved hypothetical protein | UNK |  | -0.832 |  |
| CPIJ016796 | transcription factor | UNK |  | 0.92 |  |
| CPIJ016947 | conserved hypothetical protein | UNK |  | 1.24 |  |
| CPIJ017127 | prolyl 4-hydroxylase subunit alpha-2 | UNK |  | 0.952 |  |
| CPIJ017459 | 2-succinylbenzoate-CoA ligase | UNK |  | -1.064 |  |
| CPIJ017572 | focal adhesion kinase | UNK |  | 0.858 |  |
| CPIJ017628 | EF-hand domain-containing family member C2 | UNK |  | -0.843 |  |
| CPIJ017675 | predicted protein | UNK |  | 1.021 |  |
| CPIJ018016 | glyoxylase 3 | UNK |  | -0.834 |  |
| CPIJ018355 | smad | UNK |  | 1.011 |  |
| CPIJ018503 | conserved hypothetical protein | UNK |  | -1.025 |  |
| CPIJ018522 | conserved hypothetical protein | UNK |  | -0.868 |  |
| CPIJ018614 | malate dehydrogenase | UNK |  | -0.972 |  |
| CPIJ018875 | zinc finger protein 40 | UNK |  | 0.885 |  |
| CPIJ018895 | myosin iii | UNK |  | 0.753 |  |
| CPIJ019121 | pentatricopeptide repeat domain 3 | UNK |  | -0.806 |  |
| CPIJ019862 | hypothetical protein | UNK |  | -1.093 |  |
| CPIJ019919 | Cnot1 protein | UNK |  | 0.823 |  |
| CPIJ020052 | conserved hypothetical protein | UNK |  | -0.786 |  |
| CPIJ020183 | folate carrier protein | UNK |  | 0.845 |  |
| CPIJ000698 | conserved hypothetical protein | UNK | 0.994 |  |  |
| CPIJ000972 | DNA-directed RNA polymerase, mitochondrial | UNK | 0.827 |  |  |
| CPIJ005322 | conserved hypothetical protein | UNK | 0.884 |  |  |
| CPIJ008984 | conserved hypothetical protein | UNK | 1.488 |  |  |
| CPIJ013510 | conserved hypothetical protein | UNK | 1.698 |  |  |
| CPIJ017191 | conserved hypothetical protein | UNK | 0.779 |  |  |
| CPIJ003962 | conserved hypothetical protein | UNK | -0.891 |  |  |
| CPIJ010146 | conserved hypothetical protein | UNK | -0.833 |  |  |
| CPIJ006169 | WD repeat protein 74 | UNK | -0.783 |  |  |
| CPIJ006576 | conserved hypothetical protein | UNK | 0.872 |  |  |
| CPIJ019205 | hypothetical protein | UNK | -0.753 |  |  |
| CPIJ013933 | conserved hypothetical protein | UNK | 0.774 |  |  |
| CPIJ020234 | zinc finger protein 232 | UNK | 0.769 |  |  |
| CPIJ008662 | conserved hypothetical protein | UNK | -1.194 |  |  |
| CPIJ001795 | conserved hypothetical protein | UNK | 1.001 |  |  |
| CPIJ003471 | conserved hypothetical protein | UNK | -1.533 |  |  |
| CPIJ006175 | down syndrome cell adhesion molecule | UNK | 0.799 |  |  |
| CPIJ001346 | conserved hypothetical protein | UNK | 1.022 |  |  |
| CPIJ004556 | conserved hypothetical protein | UNK | 1.531 |  |  |
| CPIJ004565 | wd-repeat protein | UNK | 1.033 |  |  |
| CPIJ008308 | conserved hypothetical protein | UNK | 1.546 |  |  |
| CPIJ011773 | conserved hypothetical protein | UNK | -1.025 |  |  |
| CPIJ013927 | conserved hypothetical protein | UNK | 0.779 |  |  |
| CPIJ014180 | conserved hypothetical protein | UNK | 1.005 |  |  |
| CPIJ015442 | conserved hypothetical protein | UNK | 0.852 |  |  |
| CPIJ017068 | conserved hypothetical protein | UNK | -0.893 |  |  |
| CPIJ018561 | conserved hypothetical protein | UNK | -1.409 |  |  |
| CPIJ019590 | predicted protein | UNK | 1.315 |  |  |
| CPIJ006684 | predicted protein | UNK | 0.805 |  |  |
| CPIJ009433 | conserved hypothetical protein | UNK | -0.927 |  |  |
| CPIJ012920 | F-box only protein 9 | UNK | 0.846 |  |  |
| CPIJ002123 | Rsl1d1 protein | UNK | -0.942 |  |  |
| CPIJ004654 | conserved hypothetical protein | UNK | -1.962 |  |  |
| CPIJ005306 | restin | UNK | 1.448 |  |  |
| CPIJ006776 | hematopoietic signal peptide-containing membrane domain-containing 1 | UNK | -0.96 |  |  |
| CPIJ007684 | conserved hypothetical protein | UNK | 1.012 |  |  |
| CPIJ008888 | conserved hypothetical protein | UNK | -1.47 |  |  |
| CPIJ009799 | conserved hypothetical protein | UNK | 0.945 |  |  |
| CPIJ011530 | hypothetical protein | UNK | -1.788 |  |  |
| CPIJ011909 | conserved hypothetical protein | UNK | -0.951 |  |  |
| CPIJ012556 | hypothetical protein | UNK | -0.958 |  |  |
| CPIJ014363 | Rrp15 protein | UNK | -1.14 |  |  |
| CPIJ015440 | conserved hypothetical protein | UNK | -0.792 |  |  |
| CPIJ017389 | zinc transporter | UNK | 1.118 |  |  |
| CPIJ018975 | short-chain dehydrogenase | UNK | 0.864 |  |  |
| CPIJ019563 | ribose-phosphate pyrophosphokinase 1,2 | UNK | 0.754 |  |  |
| CPIJ000252 | conserved hypothetical protein | UNK | -0.772 |  |  |
| CPIJ000464 | conserved hypothetical protein | UNK | 0.846 |  |  |
| CPIJ001930 | conserved hypothetical protein | UNK | -1.062 |  |  |
| CPIJ002565 | conserved hypothetical protein | UNK | 0.799 |  |  |
| CPIJ002583 | Ampd2 protein | UNK | 0.763 |  |  |
| CPIJ003320 | conserved hypothetical protein | UNK | 0.953 |  |  |
| CPIJ003363 | conserved hypothetical protein | UNK | 0.812 |  |  |
| CPIJ003438 | conserved hypothetical protein | UNK | 0.98 |  |  |
| CPIJ003551 | conserved hypothetical protein | UNK | 0.773 |  |  |
| CPIJ003645 | segmentation protein cap'n'collar | UNK | 0.774 |  |  |
| CPIJ003713 | Ptcd2 protein | UNK | -0.822 |  |  |
| CPIJ003830 | conserved hypothetical protein | UNK | -0.758 |  |  |
| CPIJ004812 | apoptosis inhibitor | UNK | -0.947 |  |  |
| CPIJ005228 | exosome complex exonuclease RRP43 | UNK | -0.933 |  |  |
| CPIJ005441 | hypothetical protein | UNK | -0.804 |  |  |
| CPIJ005814 | conserved hypothetical protein | UNK | -0.912 |  |  |
| CPIJ006341 | hypothetical protein | UNK | -0.805 |  |  |
| CPIJ006527 | anion exchange protein 2, slc4a2 | UNK | -0.779 |  |  |
| CPIJ006757 | conserved hypothetical protein | UNK | 0.938 |  |  |
| CPIJ007011 | THUMP domain-containing protein 1 | UNK | -0.8 |  |  |
| CPIJ008196 | conserved hypothetical protein | UNK | -0.778 |  |  |
| CPIJ008798 | conserved hypothetical protein | UNK | 0.895 |  |  |
| CPIJ009116 | conserved hypothetical protein | UNK | -0.79 |  |  |
| CPIJ009143 | phosphoribosylglycinamide formyltransferase | UNK | -0.852 |  |  |
| CPIJ009725 | conserved hypothetical protein | UNK | -0.926 |  |  |
| CPIJ010348 | prov protein | UNK | 0.783 |  |  |
| CPIJ010564 | conserved hypothetical protein | UNK | -0.948 |  |  |
| CPIJ010751 | conserved hypothetical protein | UNK | 0.968 |  |  |
| CPIJ010755 | conserved hypothetical protein | UNK | 0.81 |  |  |
| CPIJ010935 | GTP-binding nuclear protein Ran | UNK | -0.769 |  |  |
| CPIJ011608 | predicted protein | UNK | 0.773 |  |  |
| CPIJ011806 | superoxide dismutase, Mn | UNK | -0.764 |  |  |
| CPIJ013066 | conserved hypothetical protein | UNK | -0.755 |  |  |
| CPIJ013164 | conserved hypothetical protein | UNK | 0.789 |  |  |
| CPIJ013350 | conserved hypothetical protein | UNK | -0.828 |  |  |
| CPIJ013495 | conserved hypothetical protein | UNK | 0.765 |  |  |
| CPIJ013568 | exosome complex exonuclease RRP43 | UNK | -0.979 |  |  |
| CPIJ013581 | conserved hypothetical protein | UNK | -0.77 |  |  |
| CPIJ014076 | ran | UNK | -0.751 |  |  |
| CPIJ014352 | conserved hypothetical protein | UNK | -0.791 |  |  |
| CPIJ014894 | conserved hypothetical protein | UNK | -0.79 |  |  |
| CPIJ015085 | hect E3 ubiquitin ligase | UNK | 0.914 |  |  |
| CPIJ015149 | conserved hypothetical protein | UNK | 0.85 |  |  |
| CPIJ015312 | conserved hypothetical protein | UNK | 0.908 |  |  |
| CPIJ015579 | conserved hypothetical protein | UNK | 0.787 |  |  |
| CPIJ015601 | conserved hypothetical protein | UNK | 0.846 |  |  |
| CPIJ015990 | 26S proteasome regulatory subunit 7, psd7 | UNK | 0.877 |  |  |
| CPIJ016263 | conserved hypothetical protein | UNK | 0.796 |  |  |
| CPIJ017439 | conserved hypothetical protein | UNK | 1.022 |  |  |
| CPIJ017496 | roundabout | UNK | 1.68 |  |  |
| CPIJ018498 | hypothetical protein | UNK | -1.062 |  |  |
| CPIJ018921 | conserved hypothetical protein | UNK | 0.81 |  |  |
| CPIJ019000 | conserved hypothetical protein | UNK | -0.799 |  |  |
| CPIJ019391 | conserved hypothetical protein | UNK | 0.899 |  |  |
| CPIJ020272 | conserved hypothetical protein | UNK | -0.986 |  |  |
