# Supplementary material for: Molecular analysis of Culex quinquefasciatus larvae responses to Lysinibacillus sphaericus Bin toxin
Source: PLoS One. 2017 Apr 13;12(4):e0175473. doi: 10.1371/journal.pone.0175473 (PMC5391067; doi:10.1371/journal.pone.0175473)
Supplement: S3 Table — The fold change in gene expression levels relative to levels in the non-treated C. quinquefasciatus larval gut (control) is shown. p-values are for a Student’s t-test comparing fold change in gene expression upon Bin intoxicated cells. *, p<0.05; SEM, standard error of the mean. (DOCX) [file pone.0175473.s003.docx]

**Table S3:** Averaged data from four biological replicate Real-time qPCR assays of caspase-1, caspase-3, and cytochrome c gene expression after 6, 12, or 18 h of Bin exposure. The fold change in gene expression levels relative to levels in the non-treated *C. quinquefasciatus* larval gut (control) is shown. p-values are for a Student’s *t*-test comparing fold change in gene expression upon Bin intoxicated cells. *, p<0.05; SEM, standard error of the mean.

|  | **6 h of Bin exposure** | | | **12 h of Bin exposure** | | | **18 h of Bin exposure** | | |
| --- | --- | --- | --- | --- | --- | --- | --- | --- | --- |
|  | **Fold change** | **SEM** | **p-value** | **Fold change** | **SEM** | **p-value** | **Fold change** | **SEM** | **p-value** |
| **Caspase 1**  **(CPIJ008093)** | 1.037 | 0.426 | > 0.999 | 0.332 | 0.079 | 0.9604 | 1.656 | 0.706 | 0.9624 |
| **Caspase 3**  **(CPIJ009057)** | 3.464 | 1.983 | 0.3494 | 7.176 | 2.138 | 0.0032* | 8.422 | 2.068 | 0.0005* |
| **Cytochrome c**  **(CPIJ014995)** | 1.262 | 1.133 | 0.9974 | 0.067 | 0.019 | 0.9035 | 0.418 | 0.108 | 0.9730 |
